# Preeclamptic Women Have Disrupted Placental microRNA Expression at the Time of Preeclampsia Diagnosis: Meta-Analysis

**DOI:** 10.3389/fbioe.2021.782845

**Published:** 2021-12-24

**Authors:** Andja Cirkovic, Dejana Stanisavljevic, Jelena Milin-Lazovic, Nina Rajovic, Vedrana Pavlovic, Ognjen Milicevic, Marko Savic, Jelena Kostic Peric, Natasa Aleksic, Nikola Milic, Tamara Stanisavljevic, Zeljko Mikovic, Vesna Garovic, Natasa Milic

**Affiliations:** ^1^ Institute for Medical Statistics and Informatics, Faculty of Medicine, University of Belgrade, Belgrade, Serbia; ^2^ Institute of Molecular Genetics and Genetic Engineering, University of Belgrade, Belgrade, Serbia; ^3^ Center for Molecular Biology, University of Vienna, Vienna, Austria; ^4^ Faculty of Medicine, University of Belgrade, Belgrade, Serbia; ^5^ Clinic for Gynecology and Obstetrics Narodni Front, Belgrade, Serbia; ^6^ Division of Nephrology and Hypertension, Mayo Clinic, Rochester, MN, United States

**Keywords:** epigenetics, miRNA, preeclampsia, pathophysiology, meta-analysis

## Abstract

**Introduction:** Preeclampsia (PE) is a pregnancy-associated, multi-organ, life-threatening disease that appears after the 20th week of gestation. The aim of this study was to perform a systematic review and meta-analysis to determine whether women with PE have disrupted miRNA expression compared to women who do not have PE.

**Methods:** We conducted a systematic review and meta-analysis of studies that reported miRNAs expression levels in placenta or peripheral blood of pregnant women with vs. without PE. Studies published before October 29, 2021 were identified through PubMed, EMBASE and Web of Science. Two reviewers used predefined forms and protocols to evaluate independently the eligibility of studies based on titles and abstracts and to perform full-text screening, data abstraction and quality assessment. Standardized mean difference (SMD) was used as a measure of effect size.

**Results:** 229 publications were included in the systematic review and 53 in the meta-analysis. The expression levels in placenta were significantly higher in women with PE compared to women without PE for miRNA-16 (SMD = 1.51,95%CI = 0.55–2.46), miRNA-20b (SMD = 0.89, 95%CI = 0.33–1.45), miRNA-23a (SMD = 2.02, 95%CI = 1.25–2.78), miRNA-29b (SMD = 1.37, 95%CI = 0.36–2.37), miRNA-155 (SMD = 2.99, 95%CI = 0.83–5.14) and miRNA-210 (SMD = 1.63, 95%CI = 0.69–2.58), and significantly lower for miRNA-376c (SMD = –4.86, 95%CI = –9.51 to –0.20). An increased level of miRNK-155 expression was found in peripheral blood of women with PE (SMD = 2.06, 95%CI = 0.35–3.76), while the expression level of miRNA-16 was significantly lower in peripheral blood of PE women (SMD = –0.47, 95%CI = –0.91 to –0.03). The functional roles of the presented miRNAs include control of trophoblast proliferation, migration, invasion, apoptosis, differentiation, cellular metabolism and angiogenesis.

**Conclusion:** miRNAs play an important role in the pathophysiology of PE. The identification of differentially expressed miRNAs in maternal blood creates an opportunity to define an easily accessible biomarker of PE.

## Introduction

Preeclampsia (PE) has been shown to affect 1–7.5% of all pregnancies, making it one of the leading causes of maternal and fetal morbidity and mortality worldwide (Abalos et al., 2013; Witcher, 2018; Garovic et al., 2020). PE is a multi-factorial, multi-systemic pregnancy specific condition found typically after 20 weeks of gestation or early post-delivery (American College of Obstetricians and Gynecologists, 2019). Although clinical symptoms appear relatively late in pregnancy, PE pathology begins early, making the identification of potential biomarkers during the first trimester a possible strategy for identifying predictors of PE (McElrath et al., 2020). Several potential biomarkers already have been evaluated: C reactive protein (CRP), cytokines (IL-6, IL-8, TNF-α), microparticle proteins (C1RL, GP1BA, VTNC, and ZA2G), oxidative stress markers (malondialdehyde - MDA), and genetic factors (PAI-1 4G/5G polymorphism) (Black and Horowitz, 2018; Giannakou et al., 2018; Taravati and Tohidi, 2018; McElrath et al., 2020). There are few known biomarkers, however, that can accurately predict the risk for PE. The use of combinations of several biomarkers previously has been proposed as a diagnostic or predictive parameter, such as the ratio of soluble fms-like tyrosine kinase-1 to placental growth factor ratio (sFlt-1/PlGF) (Lecarpentier and Tsatsaris, 2016). A study by Garovic et al. reported podocyturia, defined as the presence of podocin-positive cells in urine sampled ≤24 h of delivery, as a 100% sensitive and specific diagnostic marker for PE (Garovic et al., 2007).

Significant progress has been made in the past decade in the assessment of epigenetic mechanisms that might be involved in the pathophysiology of PE, and which aim to identify potential diagnostic and/or predictive epigenetic markers of PE. More specifically, short non-coding microRNAs (miRNAs) are involved in post-transcriptional gene expression and play a role in numerous diseases, modulating regulatory pathways that control development, differentiation, and organ function. MiRNAs are single-stranded RNA molecules consisting of 19–24 nucleotides, and their mode of action is primarily by degrading targeted mRNA transcripts or inhibiting translation of mRNA into a protein product (Hombach and Kretz, 2016). It is also known that miRNA molecules are involved in the physiological regulation of major processes of placentation (Mouillet et al., 2015). It might therefore be anticipated that dysfunction of miRNA expression could be important for the development of PE. Studies recently published explored a possible causal relationship between miRNA expression and PE (Youssef and Marei, 2019; Hemmatzadeh et al., 2020). It has been demonstrated that expression levels of miRNAs in different tissues play a role in physiological pregnancy as regulators of trophoblast proliferation, migration, invasion, apoptosis, differentiation, cellular metabolism and placental angiogenesis (Hayder et al., 2018). The placenta is one of the main sources of miRNAs, but they also can be found in the circulation (Mouillet et al., 2015). Placental miRNA-210 expression has been the most studied in PE and other pregnancy related complications, and increased levels have been demonstrated (Muralimanoharan et al., 2012; Awamleh and Han, 2020). Results from evaluations of other frequently analyzed miRNAs, such as miRNA-155, -223, -126, -183, -182, -281b, -154, -139-5p, -29b, -181a, -15b (Mayor-Lynn et al., 2011; Yang et al., 2011; Zhao et al., 2013; Sheikh et al., 2016; Hemmatzadeh et al., 2020), suggest that miRNA expression differs according to the severity of PE (Jairajpuri et al., 2017), and also differs throughout the course of normal pregnancy (Cai et al., 2017). While some research has been done to investigate the association between miRNA expression levels and PE, there is still a lack of evidence to support the common use of miRNAs as functional biomarkers related to PE. The aim of this study was to perform a systematic review and meta-analysis to determine whether women with PE have disrupted miRNA expressions compared to women without PE.

## Materials and Methods

This systematic review was performed in accordance with the Preferred Reporting Items for Systematic Reviews and Meta-Analyses (PRISMA) and MOOSE guidelines (Stroup et al., 2000; Liberati et al., 2009).

### Study Selection

Publications were screened for inclusion in the systematic review in two phases, and all disagreements were resolved by discussion at each stage with inclusion of a third reviewer. We included studies that compared miRNA expression levels between women with and without PE. Studies were eligible for inclusion if the miRNA expression levels were measured in both groups. Studies were excluded if they: 1) investigated other outcomes, 2) did not make comparisons between PE and control groups, 3) examined other populations (animal, cell lines), 4) assessed other epigenetic markers, 5) were abstracts, or 6) were not original articles.

### Database Search

Two biostatisticians with expertise in conducting systematic reviews and meta-analyses (NM, AC) developed the search strategy. A systematic review of peer-reviewed publications was performed through searches of PubMed, Web of Science (WoS) and embase electronic databases until October 29, 2021. Search queries differed according to the database. Key words for the PubMed search were: preeclampsia and (epigenetic or epigenetics or miRNA or microRNA or DNA methylation or DNA methylation or long non coding RNA); for Wos: TS = *eclampsia and TS= (epigenetic* or microRNA or DNA methylation or gene imprinting or long non coding RNA), and for embase: preeclampsia and (epigenetics or microRNA or DNA methylation or genome imprinting or long untranslated RNA). Only publications in English were considered. In addition, reference lists of articles identified through electronic retrieval were manually searched, as well as relevant reviews and editorials. Experts in the field were contacted to identify other potentially relevant articles.

Authors of relevant articles were contacted to obtain missing data. Studies with combined data of gestational hypertension and/or chronic hypertension in pregnancy and PE were only eligible if data for the subset of women who developed preeclampsia were available.

### Article Screening and Selection

Two reviewers (AC, JML) independently evaluated the eligibility of all titles and abstracts. Studies were included in the full text screening if either reviewer identified the study as being potentially eligible, or if the abstract and title did not include sufficient information. Studies were eligible for full text screening if they included comparisons of miRNA expression levels between women with and without PE. Preeclampsia included more severe, less severe, and not specified forms. The same reviewers independently performed full text screening to select articles for inclusion according to the criteria listed under Inclusion and Exclusion Criteria. Disagreements were resolved by consensus (AC, JML) or arbitration (NM, DS).

### Data Abstraction and Quality Assessment

Two reviewers independently abstracted the following data: author(s), country of research, year of publication, study design, sample size, study population, maternal age, preeclampsia definitions, disease severity (more severe, less severe or not-specified PE), inclusion and exclusion criteria used in the original articles, sample type and time of sampling, matching, evaluated miRNAs, method for miRNA expression quantification, miRNA expression value, housekeeping gene for internal control, conclusion in original article. Each reviewer independently evaluated the quality of selected manuscripts using an adapted version of the Newcastle-Ottawa tool for observational studies (Wells et al., 2014). Reviewers used a standardized previously defined miRNA protocol when selecting and abstracting data. All detailed information about quality assessment, data extraction, variables, miRNA expression quantification methods and housekeeping gene for internal normalization are available at https://osf.io/g42ze/.

### Statistical Analysis

The primary outcome was expression levels of miRNAs, presented as means with standard deviation. GetData Graph Digitizer version 2.26.0.20 was used to read miRNA values when figures presenting miRNA expression levels were available (Digitize graphs and plots, 2013). Median was used as an approximation of the arithmetic mean, and IQR/1.35 was used as an approximation of standard deviation. If standard error was used in the original article, standard deviation was calculated as sd = se*√n, and if the range was presented, standard deviation was estimated as (max-min)/4.

Methodologies for measuring miRNA expression levels varied; therefore, the standardized mean difference (SMD) was used as a measure of effect size to examine differences between the preeclampsia and non-preeclampsia groups. SMD expresses the difference between group means in units of standard deviation and was estimated by pooling individual trial results using random-effects models via the Der Simonian-Laird method. Heterogeneity was assessed using the Chi-square Q and I2 statistic. I2 presents the inconsistency between the study results and quantifies the proportion of observed dispersion that is real, i.e., due to between-study differences and not due to random error. The categorization of heterogeneity was based on the Cochrane Handbook (Higgins et al., 2019) and states that I2<30%, 30–60% or >60%, correspond to low, moderate and high heterogeneity, respectively. Forest plots were constructed for each analysis showing the SMD (box), 95% confidence interval (lines), and weight (size of box) for each trial. The overall effect size was represented by a diamond. Meta-analysis was performed for all miRNAs with available data from at least three relevant studies.

Sensitivity analyses were conducted to examine the effects of: 1) replacement of studies that measured miRNA expression levels in the chorionic plate with studies exploring the basal plate, 2) inclusion of measurements performed in more severe, less severe or not-specified PE forms only (instead of all PE forms), 3) replacement of miRNA expression levels obtained in term controls with miRNA expression levels in preterm controls, 4) inclusion of studies exploring miRNA expression levels in moderate or mild proteinuria PE groups, instead of severe proteinuria as in the PE group in the first analysis. A *p* value < 0.05 was statistically significant. Analyses were performed using Review Manager Version 5.4 (Cochrane, 2021).

## Results

### Systematic Review

**FIGURE 1:**
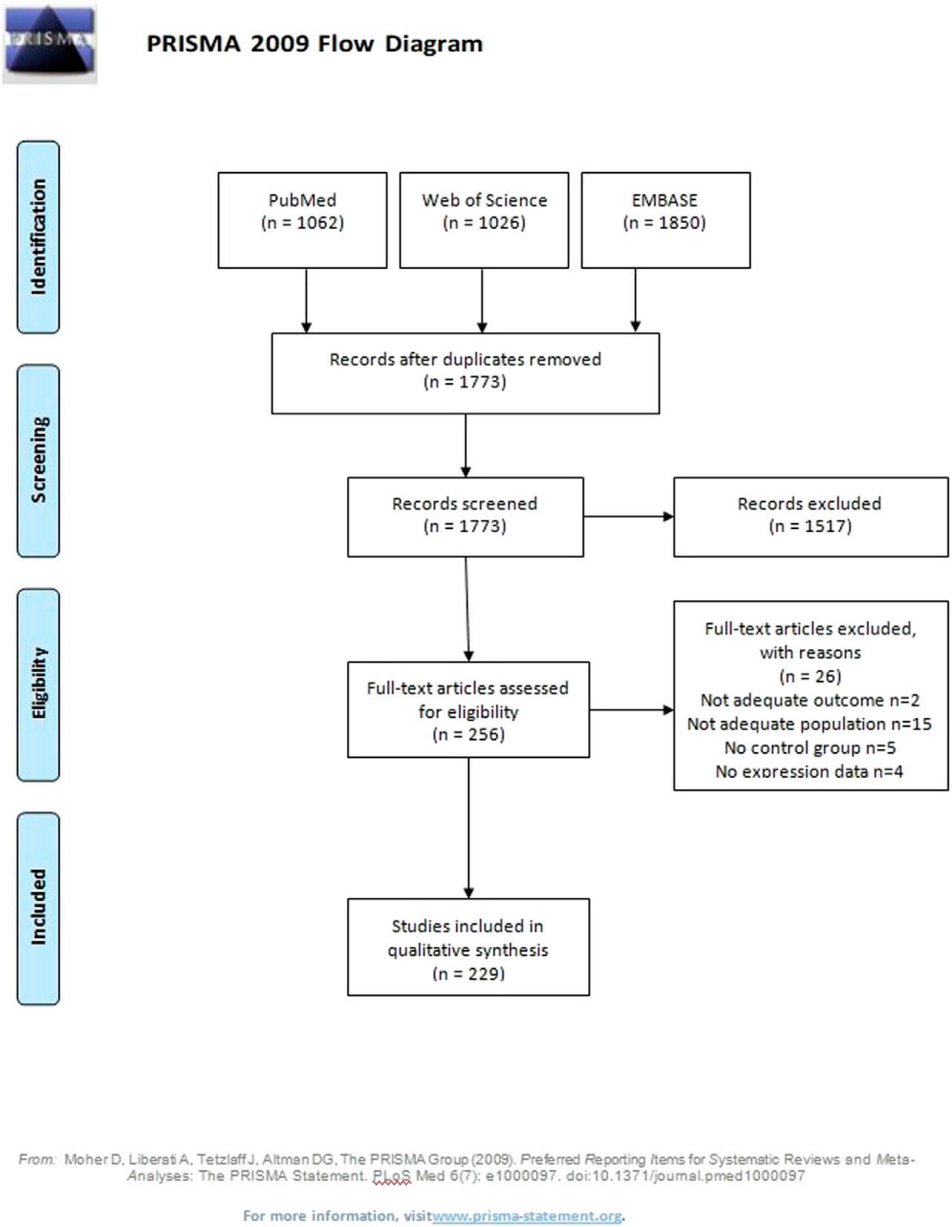
Flow diagram.

A total of 1773 potentially eligible articles were found. 1,517 articles were excluded because they were duplicates, not original articles, were without PE as the outcome, did not compare PE and control groups, examined populations other than women (animals, cell lines), did not explore miRNA expression levels, or were abstracts. Of the 256 reviewed full text articles, 229 were selected for inclusion in the systematic review. A flow diagram illustrating this selection process is presented in Figure 1.

Characteristics of all 229 publications included in the systematic review are presented in detail in Table 1. They were published between 2007 and 2021, with a total of 13043 participants; 6,459 women with and 6584 without PE. The minimum sample size of the PE group was four, and a minimum of one for the control group. The maximum sample size was 200 in PE and 321 in the control group. Four publications did not report the number of participants. 139 studies were cross-sectional, 64 were case-control, 11 were nested case-control studies, while only six were prospectively followed cohorts. Five studies included two or three sub-studies with the same or different study designs. In eighteen publications, the study design was not clearly stated. Most studies were from China (138), United States (19), and Czech Republic (8). Study groups were matched in 73 (32%) of all articles, and gestational age at the time of delivery was the most used variable for matching (in 53 of 73 publications). Maternal age at the time of delivery was used for matching in 35 publications. Other matching variables were BMI at the time of delivery, parity, race and/or ethnicity, gravidity, delivery, fetal gender, family history of PE, smoking history, additional comorbidities, systolic blood pressure at the time of inclusion, diastolic blood pressure at the time of inclusion, proteinuria at the time of inclusion, infant weight, pre-pregnancy indices, duration of storage of plasma samples, and maternal body weight at the time of delivery. Regression analysis was used to account for confounders in 14 publications. Ethnicity was reported in eight and race in eleven publications. Fetal gender was reported in 25 publications. The expression levels of miRNA were explored according to fetal gender in just three studies, and a regression model was adjusted for fetal gender in one publication. The most examined source of miRNAs was placenta, reported in 155/229 publications. Ninety-eight studies used maternal peripheral blood: plasma in 46, serum in 28, plasma exosomes in 9, mononuclear cells in 2, serum exosomes in 2, whole blood in 2, and leukocytes and buffy coat in one study each. Twelve studies analyzed miRNA expression levels in umbilical cord cell populations: mesenchymal stem cells in 4, and HUVECs, vein cells, maternal blood, exosomes, endothelial progenitor cells, serum, fetal blood, and umbilical cord tissue in one study each. Other rarely sampled tissues were myometrium, urine, maternal subcutaneous fat tissue endothelium, and placental blood vessel endothelium. Tissue was sampled at the time of delivery in 149 (65%) studies. In 60 studies, sampling was done prior to delivery and, in two studies, after delivery; 1 year after (Murphy et al., 2015), and 3–11 years after delivery (Hromadnikova et al., 2019b). Time of sampling was not reported in 34 (15%) publications. Most articles did not differentiate the type of PE (70%). Inclusion and exclusion criteria were not reported in most studies assessing miRNA in preeclamptic pregnancies. Only primiparous women were included in six studies, only non-smokers in 17, and only women without chronic hypertension in 89 publications. Detailed additional inclusion and exclusion criteria are presented in Supplementary Table S1. The presence of renal disease was the most common (50/229). The presence of diabetes mellitus (49/229) and the presence of cardiovascular disease (32/229) were reported less often. The presence of obesity was reported in six and preeclampsia in the previous gestation in five publications.

**TABLE 1:** Systematic review.

Author year* Country	Study design	Sample size		Maternal age^a^ PE vs. Controls (years)	Sample	Time of sampling	Controls/Unexposed	Matching	Inclusion criteria		
		n PE	n Controls						All primiparas	All non-smokers	No chronic hypertension
Pineles 2007* (Pineles et al., 2007) United States	Not clear (cross-sectional, case-control study)	9	9	28 (19–39) vs. 24 (18–37)	placenta	NR	pregnant women with presence of regular uterine contractions at a frequency of at least 2 contractions every 10 min that were associated with cervical changes and resulted in delivery at 37 completed weeks of gestation who delivered normal infants with birthweights appropriate for gestational age (10th–90th percentile) matched for gestational age at delivery (within 2 weeks)	Gestational age	NR	no	yes
Hu 2009* (Hu et al., 2009) China	Cross-sectional	24	26	28.1 ± 1.3 vs. 28.7 ± 1.1	placenta (chorion)	at the time of delivery	pregnant women with normal term pregnancy, without chronic hypertension, cardiovascular disease, renal disease, hepatitis, diabetes, any evidence of intrapartum infection or other pregnancy complications, such as fetal anomalies or chromosomal abnormalities	Maternal age and gestational age	no	NR	yes
Zhu 2009* (Zhu et al., 2009) China	Cross-sectional	23 Total 8 mPE 15 sPE	11	31.9 ± 3.8 (sPE); 29.5 ± 5.3 (mPE) vs. 31.8 ± 3.7	placenta (villi)	NR	normal pregnancies	Gestational age	all nulliparous	NR	yes
Zhang 2010* (Zhang et al., 2010) China	Case-control	20	20	NR	placenta (chorion)	at the time of delivery	normotensive pregnancies with gestational age matched groups	Gestational age	NR	NR	yes
Cheng 2011 (Cheng et al., 2011) China	Cross-sectional	5	5	33 ± 3 (25–40) vs. 29 ± 1 (27–33)	UC HUVECs	at the time of delivery	Healthy women	NR	NR	NR	NR
Enquobahrie 2011 (Enquobahrie et al., 2011) United States	Not clear (participants were selected from cohort and case-control studies)	20	20	32.8 ± 7.4) vs. 30.4 ± 5.6)	placenta	at the time of delivery	normotensive pregnancies uncomplicated by proteinuria matched for parity, maternal race/ethnicity, and labor status	Parity, maternal race/ethnicity and labor status	no	NR	yes
Gunel 2011 (Gunel et al., 2011) Turkey	Cross-sectional	20	20	NR	MPB (plasma)	NR	healthy pregnant women	NR	NR	NR	NR
Guo 2011 (Guo et al., 2011) China	Cross-sectional	NR	NR	NR	placenta	NR	normal pregnant women	NR	NR	NR	NR
Mayor-Lynn 2011* (Mayor-Lynn et al., 2011) United States	Cross-sectional	6	10 Total 5 term controls 5 preterm controls	23.8 (20–26) vs. 28.3 (21–38)	placenta (villi)	at the time of delivery	term controls - pregnancies who delivered normal infants at term without labor via elective Caesarean section preterm controls - presence of preterm regular uterine contractions of at least 3 contractions in 10 min that were associated with cervical changes that resulted in delivery at ≤35 completed weeks of gestation	NR	no	no	NR
Yang 2011 (Yang et al., 2011) China	Cross-sectional	4 Total 2 mPE 2 sPE	1	Individual data sPE: 28; 34 mPE: 26; 27 Control: 28 years	MPB (serum)	before delivery (during 3rd trimester)	normal pregnant women	NR	all nulliparous	NR	NR
Bai 2012*(Bai et al., 2012) China	Cross-sectional	15	17	27.5 ± 4.3 vs. 29.7 ± 2.6	placenta	at the time of delivery	normal pregnant women defined as a single gestation in a previously normotensive woman who did not suffer from high blood pressure and proteinuria during pregnancy, and delivered a healthy neonate with a weight adequate for gestational age after 37 weeks of pregnancy	Gestational age	no	NR	yes
Hromadnikova 2012 (Hromadnikova et al., 2012) Czech Republic	Not clear (retrospective, cohort)	16 + 7 who later developed PE	50	NR	MPB (plasma)	NR	normal progression of pregnancy defined as those without medical, obstetric, or surgical complications at the time of the study and who subsequently delivered full-term, singleton, healthy infants weighing >2,500 g after 37 completed weeks of gestation	Gestational age	NR	NR	NR
Ishibashi 2012 (Takizawa et al., 2012) China	Cross-sectional	8	10	Individual data for PE patients: 28, 28, 29, 31, 31, 32, 32, 36; NR for controls	placenta	NR	normal pregnancies	Gestational age	NR	NR	NR
Lazar 2012 (Lázár et al., 2012) Hungary	Not clear (prospective study)	31	28	29 (18–39) vs. 28 (20–41)	placenta	at the time of delivery	normotensive pregnant women	NR	no	NR	NR
Liu 2012*(Liu et al., 2012) China	Cross-sectional	11	16	NR	UC-MSCs placenta (decidua)	NR NR	women with normal pregnancy	NR	NR	NR	NR
Muralimanoharan 2012*(Muralimanoharan et al., 2012) United States	Cross-sectional	6	6	32.6 ± 3.6 vs. 28.6 ± 2.6	placenta (villi)	at the time of delivery	uncomplicated pregnancies	NR	NR	NR	NR
Wang 2012*(Wang et al., 2012a) United States	Cross-sectional	10	10	23 ± 1.2 vs. 23 ± 1.2	placenta	at the time of delivery	normotensive term pregnancies	NR	NR	yes	NR
Wang 2012*(Wang et al., 2012b) China	Cross-sectional	20	20	30.81 ± 0.74 vs. 30.50 ± 0.76	placenta (decidua MSCs)	at the time of delivery	maternal age and gestational age at delivery matched normotensive controls	NR	NR	NR	yes
Wu 2012*(Wu et al., 2012) China	Case-control	10	9	29.9 ± 3.1 vs. 30.4 ± 1.3	MPB (plasma)	NR	term matched normal pregnancies	Yes (no variable)	no	yes	NR
Zhang 2012 (Zhang et al., 2012) China	Case-control	30 Total 15 mPE 15 sPE	15	30.9 ± 4.1 (sPE) 31.6 ± 3.6 (mPE) vs. 29.7 ± 3.6	MPB (plasma)	NR	healthy pregnant controls who had had normal blood pressure with the absence of medical and obstetrical complications matched for age, gestational age, parity, and body mass index (BMI) at the time of blood sampling	Maternal age, gestational age, parity, and BMI at the time of sampling	NR	NR	yes
Anton* 2013 (Anton et al., 2013) United States	Case-control	40 (PE + GHTA)	33	25.5 ± 7.5 vs. 26.2 ± 6.7	MPB (serum)	before delivery (during 3rd trimester)	women without hypertension-related complications who presented for delivery at term (≥37 gestational weeks)	NR	no	NR	NR
	Nested case-control	41 (PE + GHTA)	56	31.2 ± 7.5 vs. 29.7 ± 6.6	MPB (serum)	before delivery (15–20 gw)	randomly selected from the cohort	NR	no	no	yes
Betoni 2013*(Betoni et al., 2013) United States	Case-control	16	12	26.0 ± 5.9 vs. 30.9 ± 5.8	placenta	NR	patients without PE matched for maternal age and ethnicity, as well as for type of delivery, gestational age, birth weight and sex of the child	Gestational age	no	no	yes
Choi 2013 (Choi et al., 2013) China	Cross-sectional	11	10	31.0 ± 5.3 vs. 30.7 ± 3.9	placenta	NR	normotensive pregnancies uncomplicated by proteinuria	NR	no	NR	yes
Fu, 2013*(Fu et al., 2013) China	Cross-sectional	15	22 term controls	30.8 ± 1.9 (preterm PE) 34.8 ± 2.2 (term PE) Vs. 32.0 ± 1.23 (preterm controls) 33.4 ± 0.7 (term controls)	placenta	at the time of delivery (36–40 gw)	normal pregnancies	Gestational age	NR	NR	NR
	Cross-sectional	29 Total 13 preterm PE 16 term PE	44 Total 13 preterm controls 31 term controls	29.8 ± 0.7 (preterm PE) Vs. 29.5 ± 0.8 (preterm controls) 29.2 ± 1.0 (term PE) Vs. 31.7 ± 0.6 (term controls)	MPB (plasma)	before delivery (15-18 gw and 36-40 gw)	normal pregnancies	NR	NR	NR	NR
	Cross-sectional	37 Total 16 preterm PE 11 term PE	23 preterm controls 25 term controls	NR	placenta	at the time of delivery (25-35 gw and 36–40 gw)	normal pregnancies	NR	NR	NR	NR
Guo 2013 (Guo et al., 2013) United States	Cross-sectional	16	29	NR	placenta	NR	control group	NR	no	NR	yes
Hromadnikova 2013 (Hromadnikova et al., 2013) Czech Republic	Cohort	63 Total 24 mPE 39 sPE 24 EOPE 39 LOPE	55	NR	MPB (plasma)	NR	pregnant women without complications who delivered full term, singleton, healthy infants weighting >2,500 g after 37 completed gw	Gestational age	NR	NR	no
Kumar 2013 (Kumar et al., 2013) China	Cross-sectional	8	8	NR	placenta	at the time of delivery	term gestation-matched normotensive pregnant women	Gestational age	NR	NR	yes
Li 2013 (Li et al., 2013a) China	Cross-sectional	4 mPE +4 sPE profiling study 16 mPE +22 sPE validation study	4 in profiling study 32 in validation study	34 (28–39) (sPE) 29 (23–36) (mPE) vs. 28 (26–30) in profiling study 33 (24–43) (sPE) 31 (26–39) (mPE) vs. 29 (25–36) in validation study	MPB (plasma)	before delivery	normal pregnancies, age, gestational week and gravidity matched with PE	Maternal age, gestational age, and gravidity	yes	NR	yes
Li 2013*(Li et al., 2013b) China	Cross-sectional	24	26	28.1 ± 1.3 vs. 28.7 ± 1.1	placenta (chorion)	at the time of delivery	normal term pregnancies without chronic hypertension, cardiovascular disease, renal disease, hepatitis, diabetes, any evidence of intrapartum infection or other pregnancy complications, such as fetal anomalies or chromosomal abnormalities	NR	no	NR	NR
Yan 2013 (Yan et al., 2013a) China	Case-control	12	12	31.4 ± 4.03 vs. 30.3 ± 3.67	placenta	NR	normotensive and nonproteinuric during pregnancy and delivered healthy infants of appropriate weight	Maternal age, BMI, and gestational age	NR	yes	yes
Campos 2014 (Campos et al., 2014) Brasil	Cross-sectional	19	14	26 ± 6 vs. 27 ± 6	MPB (plasma)	before delivery (at the time of clinical attendance)	healthy pregnant women	NR	no	yes	yes
Chen 2014 (Chen et al., 2014) China	Cross-sectional	20 Total 15 mPE 5 sPE	40	27 (24–34) vs. 25 (25–30)	placenta	NR	normal deliveries	NR	NR	NR	NR
Doridot 2014 (Doridot et al., 2014) France	Cross-sectional	5	8	NR	placenta	NR	Women who underwent Caesarean surgery without suffering any disease during pregnancy	NR	NR	NR	NR
Hong 2014 (Hong et al., 2014) China	Case-control	115	115	NR	placenta	at the time of delivery	gestational age matched normotensive pregnancies	Gestational age	NR	NR	yes
Lalevee 2014*(Lalevée et al., 2014) Switzerland	Not clear (prospective case–control study)	15	14	36.1 (22.6–44.5) vs. 33.3 (26.5–37.2)	placenta	at the time of delivery	controls	NR	no	NR	NR
Li 2014 (Li et al., 2014a) China	Case-control	13	26	29.58 ± 0.68 vs. 29.56 ± 0.48	Placenta (basal plate and chorionic plate)	at the time of delivery	gestation-week-matched pregnant healthy controls without renal disease, cardiovascular disease, transient hypertension in pregnancy, gestational diabetes mellitus, hepatitis. Any evidence of spontaneous abortion, intrauterine fetal death, fetal chromosomal or other pregnancy complications were excluded from this study	Gestational age	no	NR	yes
Li 2014*(Li et al., 2014b) China	Cross-sectional	19	22	27.6 ± 4.2 vs. 28.2 ± 4.5	placenta	at the time of delivery	normal pregnant women defined as previously and currently normotensive female during pregnancy who delivered a healthy neonate following 37 weeks of gestation	NR	NR	NR	yes
Luo 2014*(Luo et al., 2014) China	Case-control	15	26	29.3 ± 1.3 vs. 31.6 ± 0.9	placenta (chorionic villi) placenta (chorionic plate and basal plate)	after abortion/elective termination (7–8 gw) at the time of delivery	normal pregnant women defined as gestation in a previously normotensive woman who did not suffer from any complications during pregnancy and who delivered a healthy neonate with a weight adequate for a gestational age of more than 37 weeks of pregnancy	Gestational age	NR	NR	yes
Luque 2014 (Luque et al., 2014) Spain	Nested case-control	31	44	32.6 ± 6.6 vs. 32.3 ± 5.6	MPB (serum)	before delivery (11 + 0, 13 + 6 gw)	normotensive pregnancies without proteinuria	NR	no	no	yes
Ura 2014 (Ura et al., 2014) Italy	Not clear (retrospective study)	24	24	34.4 (33.0–36.8) vs. 33.7 (30.3–36.1)	MPB (serum)	before delivery (12–14 gw)	normal pregnancies	NR	no	no	NR
Wang 2014 (Wang et al., 2014) China	Not clear	NR	NR	NR	placenta	NR	normal pregnancies	NR	NR	NR	NR
Weedon-Fekjaer 2014 (Weedon-Fekjær et al., 2014) Norway	Cross-sectional	49 Total 23 EOPE 26 LOPE	23	NR	placenta	at the time of delivery	uncomplicated pregnancies delivered at term (37–41 gw)	NR	NR	NR	yes
Winger 2014 (Winger et al., 2014) United States	Not clear (retrospective study)	12 Total 7LOPE 5EOPE	19	43.7 ± 8.7 vs. 37.6 ± 5.1	MPB	before delivery (1st trimester)	delivery of a singleton normal karyotype baby with the following pregnancy criteria: (i) delivered at 37- to 40-weeks of gestation, (ii) birthweight of ≥6 lbs, (iii) normal maternal blood pressure throughout pregnancy or (iv) twin delivery with gestational age ≥35 weeks with birthweights of ≥5.1 lbs and (v) no other pregnancy or delivery complications	NR	NR	NR	NR
Xu 2014*(Xu et al., 2014) China	Case-control	14	33	NR	placenta (chorionic plate and basal plate)	at the time of delivery	gestational week matched normal pregnant women	Gestational age	NR	NR	NR
	Case-control	20	20	NR	MPB (plasma)	before delivery (15–19 gw) at the time of delivery (35–39 gw)	gestational week matched normal pregnant women	Gestational age	NR	NR	NR
Zhao 2014*(Zhao et al., 2014) China	Case-control	20	20	28.9 ± 1.2 vs. 29.2 ± 1.4	Placenta (decidual MSCs)	at the time of delivery	age matched normotensive controls	Maternal age	no	NR	yes
Zou 2014 (Zou et al., 2014) China	Cross-sectional	30	30	30.2 ± 5.7 vs. 30.6 ± 3.5	placenta	at the time of delivery (immediately after placental delivery)	normal pregnant women defined as not having PE or any other complications (including maternal history of hypertension and/or renal disease, maternal infection, smoking, alcoholism, chemical dependency, and fetal congenital anomalies)	NR	yes	no	NR
Akehurst 2015*(Akehurst et al., 2015) Scotland	Not clear (prospective study)	18	18	31 ± 5.3 vs. 31 ± 5.4	MPB (plasma)	before delivery (16–18 gw)	matched for age, BMI, and parity	Maternal age, BMI, and parity	no	no	NR
	Case-control	19	19	29 ± 5.4 vs. 30 ± 4.6	placenta	at the time of delivery	normotensive individuals matched for age, BMI, and parity	Maternal age, BMI, and parity	no	no	NR
	Not clear	2	9 term	NR	myometrium	at the time of delivery	normotensive women	Maternal age, BMI, and parity	NR	NR	NR
Anton 2015 (Anton et al., 2015) United States	Case-control	31 Total 18 term PE 13 preterm PE	14	28.1 ± 7.7 (total) 28.3 ± 8.2 (term PE) 27.8 ± 7.3 (preterm PE) vs. 27.0 ± 7.2	placenta	at the time of delivery	women without hypertension-related complications that presented for delivery at term (37 gestational weeks)	NR	NR	NR	NR
Chen 2015 (Chen et al., 2015) China	Cross-sectional	5	10	NR	placenta (decidua MSCs)	at the time of delivery	healthy pregnancies	NR	NR	NR	NR
Ding 2015 (Ding et al., 2015) China	Case-control	18	21	28.44 ± 0.95 vs. 30.05 ± 0.72	placenta	at the time of delivery	normal pregnancy defined as patients with no history of hypertension or proteinuria during weeks 35–40 of pregnancy who delivered healthy neonates via Caesarean section	Maternal age, gestational age	yes	NR	yes
Hromadnikova 2015a (Hromadnikova et al., 2015a) Czech Republic	Cohort	80	20	33 (30–36) vs. 30 (26.5–33)	placenta	NR	without medical, obstetrical, or surgical complications at the time of the study and who subsequently delivered full term, singleton healthy infants weighing >2,500 g after 37 completed weeks of gestation	NR	NR	NR	yes
Hromadnikova 2015b (Hromadnikova et al., 2015b) Czech Republic	Cohort	63	42	31.7 ± 5.0 vs. 30.6 ± 4.4	placenta	NR	those without medical, obstetrical, or surgical complications at the time of the study and who subsequently delivered full-term, singleton healthy infants weighing >2,500 g after 37 completed weeks of gestation	NR	NR	NR	yes
Hu 2015 (Hu et al., 2015) China	Cross-sectional	24 Total 17 7	24 Total 17 7	27.42 ± 3.89 vs. 27.11 ± 3.18	umbilical cord vein UC-MSC	NR	normal pregnancies delivered after 34 weeks	NR	NR	NR	NR
Jiang 2015 (Jiang et al., 2015) China	Cross-sectional	20	20	28.1 ± 4.8 vs. 26.3 ± 5.2	placenta	NR	previously and currently normotensive pregnant female, who delivered a healthy neonate following 37 weeks of gestation	NR	NR	NR	yes
Lasabova 2015*(Lasabová et al., 2015) Slovak Republic	Case-control	11	7	27.6 ± 4.9 vs. 26.6 ± 2.8	placenta	at the time of delivery	normotensive healthy singleton pregnancies with no history of cigarette smoking, diabetes autoimmune disease, or thrombophilia	NR	NR	NR	yes
Li 2015*(LI et al., 2015) China	Case-control	60 Total 12 - 1st trimester 20 - 2nd trimester 28 - 3rd trimester	60 Total 12 - 1st trimester 20 - 2nd trimester 28 - 3rd trimester	28.7 ± 3.6 vs. 28.1 ± 3.8	MPB (serum)	before delivery (after ≥8 h fasting)	healthy pregnant women without complications	Maternal age at delivery within 1-year-old gap and gestational age of blood sampling	NR	NR	NR
Miura 2015 (Miura et al., 2015) Japan	Case-control	20 Total 6 sEOPE 14 sLOPE	20	31.9 ± 2.9 sEOPE 30.2 ± 4.4 sLOPE vs. 32.8 ± 4.0	MPB (plasma)	before delivery (27–34 gw)	Uncomplicated gestational age matched pregnant women	Gestational	NR	NR	NR
Murphy 2015 (Murphy et al., 2015) Canada	Cohort	13 Total 7 mPE 6 sPE	17	30.4 ± 7.3 (total) 32.4 ± 6.9 (mPE) 28.0 ± 7.6 (sPE) vs. 28.2 ± 4.1	MPB (plasma)	at the time of delivery (peripartum) after delivery (1 year postpartum)	Uncomplicated pregnancies	Time	NR	NR	NR
Sun 2015 (Sun et al., 2015) China	Cross-sectional	20	20	29 ± 3.7 vs. 28.9 ± 2.5	placenta	at the time of delivery	Healthy pregnancies	NR	NR	NR	NR
Winger 2015 (Winger et al., 2015) United States	Cross-sectional	12 Total 7 LOPE 5 EOPE 5 preconceptional PEs 5 1st trimester	20 Total 11 pre-conception controls 9 Controls for 1st trimester	36.7 ± 3.5 (total) 37.7 ± 3.8 (LOPE) 35.2 ± 2.8 (EOPE) vs. 36.3 ± 4.7	MPB	before delivery (pre-conception and 1st trimester)	Healthy pregnant women in healthy pregnancies	NR	NR	NR	NR
Yang 2015 (Yang et al., 2015) China	Cross-sectional	4	1	PE patients’ individual data mPE: 27, 26 sPE: 34, 28 vs. Controls NR	MPB (plasma) placenta	before delivery at the time of delivery	Pregnant women without complications	NR	NR	NR	NR
Zhang 2015*(Zhang et al., 2015) China	Cross-sectional	3	3	29.58 ± 0.68 vs. 29.56 ± 0.48	placenta (basal plate and chorionic plate)	at the time of delivery	Normal pregnant controls without any complications	Yes (no variable)	NR	NR	NR
Hromadnikova 2016*(Hromadnikova et al., 2016) Czech Republic	Not clear (retrospective study from prospective cohort)	68 Total 32 mPE 36 sPE 24 EOPE 44 LOPE	20	33 (30–36) vs. 30 (26.5–33)	MPB (whole peripheral blood)	NR	Normal pregnancies defined as those without medical, obstetrical, or surgical complications at the time of the study and who subsequently delivered full term, singleton healthy infants weighing >2,500 g after 37 completed weeks of gestation	NR	NR	NR	no
Hu 2016 (Hu et al., 2016) China	Cross-sectional	19	23	27.42 ± 3.89 vs. 27.11 ± 3.18	placenta	NR	healthy pregnant women at term	NR	no	NR	yes
Munaut 2016*(Munaut et al., 2016) Belgium	Not clear (retrospective study from prospective cohort)	23	44	29 (19–44) vs. 30 (19–38)	MPB (serum)	before delivery	pregnant women presenting, at 24 to <37 weeks’ gestation, clinical suspicion of, but not manifesting preeclampsia/eclampsia/HELLP syndrome	NR	no	no	no
Ospina-Prieto (Ospina-Prieto et al., 2016) 2016 Germany	Cross-sectional	11	13	27.0 ± 2.8 (total) 28.0 mean EOPE 26.4 mean LOPE vs. 29.5 ± 5.8	placenta (villi)	at the time of delivery (immediately after delivery)	NR	Maternal age		yes	
Sandrim 2016a (Sandrim et al., 2016a) Brasil	Case-control	7	10	24 ± 6 vs. 28 ± 6	MPB (plasma)	at the time of delivery	healthy pregnancies matched for gestational age at sampling, maternal age, and BMI	Gestational age, maternal age, and BMI	NR	NR	NR
Sandrim 2016b (Sandrim et al., 2016b) Brasil	Case-control	19	14	26 ± 5 vs. 27 ± 6	MPB (plasma)	before delivery	healthy pregnant women	NR	no	yes	yes
	Nested case-control	8	8	NR	MPB (plasma)	before delivery (35 + 1 and 35 + 5 gw)	healthy pregnant women	NR	no	yes	NR
Vashukova 2016 (Vashukova et al., 2016) Russia	Cross-sectional	5	6	35.0 ± 2.4 vs. 29.3 ± 0.6	placenta	at the time of delivery	normal pregnancies	NR	NR	NR	yes
Wang 2016 (Wang et al., 2016a) United States	Cross-sectional	5	5	26 ± 5 (20–33) vs. 29 ± 7 (20–37)	Maternal subcutaneous fat tissue endothelial cells	at the time of delivery	normal pregnancies defined as pregnancy with blood pressure (<140/90 mm Hg), absence of proteinuria, and obstetrical and medical complications	NR	NR	yes	NR
Wang 2016 (Wang et al., 2016b) China	Case-control	34 Total 13 PE age 21–29 years 13 PE age >30 years 8 PE with complications (chronic HTA and GDM)	13	25.69 ± 1.31 vs. 29.08 ± 2.60	MPB	NR	normal pregnant women	NR	no	NR	NR
Yang 2016 (Yang et al., 2016) China	Cross-sectional	17	40	28.85 ± 2.02 vs. 28.96 ± 4.11	placenta (chorionic plate, basal plate) MPB (plasma)	at the time of delivery NR	normal pregnant women	NR	no	NR	NR
Zhou 2016*(Zhou et al., 2016) China	Cross-sectional	31 Total 9 discovery set 22 validation set	29 Total 9 discovery set 20 validation set	Discovery set 32.1 ± 6.9 vs. 28.3 ± 1.4 Validation set 30.4 ± 4.7 vs. 30.5 ± 4.4	placenta (chorionic plate)	at the time of delivery	normal pregnant women	NR	NR	NR	NR
Adel 2017*(Adel et al., 2017) Egypt	Cross-sectional	35 Total 25 mPE 10 sPE	35	24 (18–40) vs. 25 (19–35)	placenta (villi)	at the time of delivery	primigravid normotensive throughout gestation with no excess albumin in urine	NR	NR	NR	NR
Azizi 2017*(Azizi et al., 2017) Iran	Case-control	59	40	27.42 ± 6.7 vs. 23.78 ± 4.15	placenta (chorion)	at the time of delivery	gestational age-matched normotensive pregnancies	Gestational age	all nulliparous	NR	yes
Fang 2017 (Fang et al., 2017) China	Cross-sectional	12	12	NR	placenta (trophoblast cells)	at the time of delivery	normal pregnancies	NR	NR	NR	NR
Gan 2017 (Gan et al., 2017) China	Case-control	20	20	28.95 ± 4.16 vs. 30.05 ± 4.22	MPB (serum) urine	before delivery before delivery	healthy pregnant women without complications were selected as the control based on similar maternal age at delivery and the similar weight at delivery	Maternal age and maternal weight at delivery	NR	NR	NR
Gao 2017 (Gao et al., 2017) China	Cross-sectional	26	18	30.8 ± 5.2 vs. 29.6 ± 4.6	MPB (plasma)placenta	before delivery (16, 20, 24, 30 gw) NR	normal pregnancies were defined as those without medical, obstetric or surgical complications at the time of the study and who subsequently delivered full term, singleton healthy infants weighing >2,500 g after 37 completed weeks of gestation	NR	NR	NR	NR
Gunel 2017 (Gunel et al., 2017) Turkey	Case-control	18	18	NR	MPB (plasma)	at the time of delivery	matched for age, gestational week, and gravidity healthy pregnancies 37-40 gw	Maternal age, gestational age, and gravidity	yes	NR	NR
Guo 2017 (Guo et al., 2017) China	Cross-sectional	29	26	32.14 ± 1.17 vs. 29.64 ± 1.00	placenta	at the time of delivery	healthy pregnant women	NR	NR	no	yes
Han 2017 (Han et al., 2017) China	Cross-sectional	40	20	30.25 ± 5.16 vs. 29.74 ± 4.16	placenta	at the time of delivery	women in normal late pregnancy	NR	NR	yes	NR
Hromadnikova 2017 (Hromadnikova et al., 2017) Czech Republic	Not clear (retrospective study)	56 Total 15 mPE 41 sPE 19 EOPE 37 LOPE	44	33 (22–43) vs. 32 (20–39)	UC blood	NR	Normal pregnancies defined as those without medical, obstetrical, or surgical complications at the time of the study and who subsequently delivered full term, singleton healthy infants weighing >2,500 g after 37 completed weeks of gestation	NR	no	NR	no
Hu 2017 (Hu et al., 2017) China	Cross-sectional	19	23	NR	placenta	at the time of delivery	healthy pregnant women at term	NR	NR	NR	NR
Huang 2017 (Zhang et al., 2017) China	Nested case-control	26	52	28.3 ± 3.8 vs. 28.1 ± 4.4	MPB (plasma)	before delivery (12–20 gw)	healthy pregnant women who had no relevant disease over the same period	gestational age and maternal age	no	no	yes
Jairajpuri 2017 (Jairajpuri et al., 2017) Kingdom of Bahrain	Cross-sectional	15	7	30 (25–38) (mPE) 34 (28–39) (sPE) vs. 29 (23–36)	MPB (plasma)	NR	no previous history of hypertension, cardiovascular disease, hepatitis, kidney disease, diabetes, and any evidence of intrapartum infection or other complications of pregnancy such as fetal anomalies or chromosomal abnormalities	Maternal age and BMI	yes	NR	no
Jiang 2017 (Jiang et al., 2017) China	Case-control	19	19	31.3 ± 5.8 vs. 30.9 ± 5.6	MPB (serum)	1st trimester 10-14 gw 2nd trimester 20-24 gw 3rd trimester 30-34 gw	healthy pregnant women without complications	maternal age (±1 year) at delivery and gestational age	NR	NR	yes
Jin 2017*(Jin et al., 2017) China	Cross-sectional	15	15	NR	Placenta MPB	NR NR	normal pregnancies	NR	NR	NR	NR
Korkes 2017*(Korkes et al., 2017) United States	Cross-sectional	11	11	31.6 ± 1.63 vs. 34.36 ± 1.5	placenta	NR	normal pregnancies	NR	NR	NR	yes
Li 2017a*(Li et al., 2017a) China	Cross-sectional	NR	NR	NR	placenta	at the time of delivery	Normal pregnancy without preeclampsia or any other complications	NR	NR	NR	NR
Li 2017b (Li et al., 2017b) China	Case-control	32 Total 24 (UC tissue) 8 (UC-MSCs)	30 Total 24 (UC tissue) 6 (UC-MSCs)	29.5 ± 0.9 vs. 28.9 ± 0.5 (UC tissue) 29.6 ± 0.2 vs. 28.7 ± 0.9 (UC-MCSs)	UC tissue UC-MSCs	at the time of delivery	healthy pregnancies who underwent Caesarean section	NR	no	NR	yes
Lu 2017 (Lu et al., 2017) China	Cross-sectional	84 Total 38 mPE 46 sPE	50	28.5 ± 1.6 (mPE) 29.2 ± 2.1 (sPE) vs. 28.6 ± 1.3	placenta	at the time of delivery	normal pregnancy	NR	NR	no	NR
Luo 2017 (Luo et al., 2017a) NR	Cross-sectional	16	16	NR	placenta	NR	NR	NR	NR	NR	NR
Luo 2017 (Luo et al., 2017b) China	Case-control	23	15	30.6 ± 1.0 vs. 28.1 ± 0.9	placenta	at the time of delivery	healthy women not having preeclampsia or any other complications, such as maternal history of hypertension and/or renal or cardiac disease, maternal infection, multiple pregnancies, premature rupture of membranes or fetal anomalies	NR	NR	NR	NR
Meng 2017 (Meng et al., 2017) Inner Mongolia (China)	Cross-sectional	20	10	28.9 ± 0.15 vs. 28.3 ± 0.21	placenta	at the time of delivery	normal pregnancy	NR	all nulliparous	NR	NR
Nizyaeva 2017*(Nizyaeva et al., 2017) NR	Cross-sectional	10 Total 5 EOPE 5 LOPE	8 Total 4 preterm 4 full-term	23–40 for all respondents	Placenta (syncytiotrophoblast and syncytial knots)	NR	preterm controls - women without clinical manifestations of hypertensive disorders and without inflammatory diseases (no inflammatory infiltration was confirmed by results of histological analysis) term controls - uterine scar after the previously surgery, severe myopia, and anatomically narrow pelvis	NR	NR	NR	NR
Salomon 2017 (Salomon et al., 2017) Chile	Not clear (retrospectively stratified case-control experimental design)	45 Total 15 11–14 gw 15 22–24 gw 15 32–36 gw	96 Total 32 11–14 gw 32 22–24 gw 32 32–36 gw	29 ± 1.6 (18–40) vs. 25 ± 1.2 (18–36)	MPB (plasma exosomes)	11–14 gw 22–24 gw 32–36 gw	healthy subjects without pregnancy complications or chronic medical problems, and did not differ in racial origin from PE patients	Gestational age	NR	yes	yes
Shao 2017 (Shao et al., 2017) China	Case-control	24 Total sEOPE 10 sLOPE 14	43 10 Preterm controls 33 Term controls	29.8 ± 6.5 (total) 30.3 ± 6.2 (sEOPE) 28.8 ± 5.3 (sLOPE) vs. 29.2 ± 5.6 (preterm controls) 28.6 ± 4.7 (normal pregnancy)	placenta	at the time of delivery	Term controls - gestation in a previously healthy woman who did not experience any complications during pregnancy and who delivered a healthy neonate with a weight adequate for a gestational age of longer than 37 weeks Preterm controls - unexplained preterm labor defined as labor of unknown causes earlier than 34 weeks, but without any other diagnosable pregnancy problems	Gestational age	NR	NR	yes
Singh 2017 (Singh et al., 2017) United States	Cross-sectional	4	4	NR	placenta (chorionic villi)	before delivery (11–12 gw)	healthy pregnancies who delivered at term matched for gestational age at CVS (+/- 6 days), fetal sex, parity with PE women	Gestational age (+/- 6 days), fetal sex and parity	NR	yes	yes
Truong 2017 (Truong et al., 2017) United States	Case-control	6	6	32 ± 4.3 (28 ± 33) vs. 31 ± 2.9 (29 ± 35)	MPB (plasma exosomes)	before delivery (before 20 gw)	women without chronic medical conditions or obstetric complications	NR	NR	NR	yes
Tsai 2017 (Tsai et al., 2017) Taiwan	Case-control	31	60	33.83 ± 5.77 vs. 31.33 ± 4.31	MPB (plasma) fetal cord blood (plasma) placenta	before delivery - within hours to 2 days before delivery at the time of delivery	healthy controls	Gestational age	NR	NR	yes
Wang 2017 (Wang et al., 2017) China	Cross-sectional	25	25	20–35 for all respondents	placenta	at the time of delivery	healthy controls	NR	NR	NR	yes
Wei 2017 (Wei et al., 2017) New Zealand	Cross-sectional	7	4	28.0 ± 5.78 vs. 32 ± 4.99	placenta (trophoblast debris)	at the time of delivery	normotensive term pregnancies	NR	NR	NR	yes
Xiao 2017 (Xiao et al., 2017) China	Cross-sectional	30	30	28.34 ± 4.12 vs. 28.81 ± 4.94	placenta	at the time of delivery	healthy pregnancies who underwent Caesarean section	NR	NR	NR	NR
Xu 2017 (Xu and Zhang, 2017) China	Cross-sectional	25	25	NR	placenta	NR	normal pregnancies	NR	NR	NR	NR
Yang 2017*(Yang et al., 2017) China	Cross-sectional	60	20	NR	MPB (serum) placenta	at the time of delivery	subjects who were normotensive during pregnancy and who, both previously and presently, had delivered a healthy neonate after 37 weeks of gestation	NR	NR	NR	yes
Brkic 2018 (Brkić et al., 2018) China	Cross-sectional	15	15	36.67 ± 0.27 vs. 37.56 ± 0.2	Placenta (chorionic plate and basal plate)	at the time of delivery	previously normotensive women who did not suffer from complications during pregnancy and who delivered a healthy neonate with a weight adequate for a gestational age	Gestational age	NR	NR	yes
	Case-control	9 Term PE	69 Total 13 1st trimester 9 2nd trimester 23 preterm control 24 term control	32 ± 1.17 (term PE) vs. (preterm control) 33 ± 0.76 (term control)	placenta (trophoblast cells)	at the time of delivery	1st and 2nd trimester - healthy patients undergoing elective termination of pregnancy Preterm controls – spontaneous preterm labor delivered either by Caesarean section for fetal distress or vaginal delivery Term controls - vaginal delivery or elective Caesarean sections with Appropriate for Gestation Age babies	NR	NR	NR	NR
Chi 2018 (Chi and Zhang, 2018) China	Cross-sectional	30	30	25–35 for all respondents	placenta (placental villi)	NR	age matched healthy controls	Maternal age	NR	NR	NR
Dai 2018 (Dai and Cai, 2018) China	Cross-sectional	63 Total 55 sEOPE 8 sLOPE	65	29.7 ± 4.2 vs. 30.8 ± 3.9	placenta (trophoblast cells)	NR	pregnancies free of any pregnancy complications that terminated between 34 and 40 gestational weeks	Maternal age, BMI and gestational age	no	NR	NR
Fang 2018 (Fang et al., 2018) China	Cross-sectional	50	50	29.8 ± 4.2 vs. 30.5 ± 3.2	Placenta	NR	normal pregnant women	NR	NR	NR	NR
Gao 2018 (Gao et al., 2018a) China	Cross-sectional	42	42	30.12 ± 3.98 vs. 32.36 ± 4.87	Placenta	at the time of delivery	normal pregnancy	NR	NR	NR	NR
Gao 2018 (Gao et al., 2018b) China	Cross-sectional	29	35	28.3 ± 4.2 vs. 27.2 ± 3.1	Placenta	at the time of delivery	pregnant women without PE or any other complications, such as premature rupture of membranes, fetal anomalies, maternal history of hypertension and/or renal or cardiac disease, maternal infection, or smoking	NR	NR	NR	NR
Gunel 2018 (Gunel et al., 2020) Turkey	Cross-sectional	10	10	30.7 ± 2.3 vs. 31.75 ± 3.92	MPB (plasma) placenta	just before delivery at the time of delivery	healthy women	NR	NR	NR	NR
Guo 2018 (Guo et al., 2018) China	Cross-sectional	20	20	28.6 ± 3.1 vs. 27.1 ± 2.6	MPB (plasma and serum)	at the time of delivery	healthy pregnant women	NR	NR	NR	NR
Khaliq 2018*(Khaliq et al., 2018) South Africa	Cross-sectional	28	32	Not clear	MPB (serum) placenta	NR at the time of delivery	normotensives with no obstetrical or medical complications	NR	NR	NR	yes
Kim 2018 (Kim et al., 2018) Republic of Korea	Cross-sectional	17	17	NR	MPB (serum)	NR	normal pregnant women	NR	NR	NR	NR
Li 2018*(Li et al., 2018) China	Cross-sectional	91 Total 40 mPE 51 sPE	67	29.4 ± 2.8 vs. 28.4 ± 3.5	MPB (plasma) placenta	during the treatment at the time of delivery	normal pregnant women	NR	NR	NR	yes
Liu 2018 (Liu et al., 2018) China	Cross-sectional	18	20	30.3 ± 4.6 vs. 29.5 ± 4.3	Placenta	at the time of delivery	normal pregnant women	NR	NR	NR	NR
Lou 2018 (Lou et al., 2018) China	Case-control	28	34	NR	Placenta	at the time of delivery	age matched healthy controls	Maternal age	NR	NR	NR
Lykoudi 2018 (Lykoudi et al., 2018) Greece	Cross-sectional	16 Total 11 EOPE 5 LOPE	8	35.1 (28–45) EOPE 28.4 (20–35) LOPE vs. 35.7 (35–39)	Placenta	at the time of delivery	uncomplicated term pregnancies	NR	NR	NR	NR
Martinez-Fierro 2018 (Martinez-Fierro et al., 2018) Mexico	Nested case–control study	45 in total 6 at 12 gw 10 at 16 gw 14 at 20 gw 15 at the time of diagnosis	18	23.5 ± 5.1 vs. 23.4 ± 5.8	MPB (serum)	before delivery (12th, 16th and/or 20th gw) at enrolment and PE patients at the time of diagnosis	matched healthy pregnancies without complications (normotensive controls)	NR	no	yes	yes
Motawi 2018 (Motawi et al., 2018) Egypt	Case-control	100 Total 23 EOPE 77 LOPE	100 Total 20 early pregnancy controls 80 late pregnancy controls	28.77 ± 5.72 vs. 28.06 ± 5.65	MPB (plasma exosomes)	NR	uncomplicated pregnancy: (1) gestational age at venipuncture between 20 – 42 weeks; (2) no medical, obstetrical, or surgical complications; (3) absence of labor at the time of venipuncture; and (4) delivery of a normal term (≥37 weeks) neonate whose birth weight was between the 10th and 90th percentile for gestational age. Divided into early (<20 gw) and late (>20 gw) pregnancy control groups	Maternal age	NR	NR	yes
Niu 2018* (Niu et al., 2018) China	Cross-sectional	25	20	27.9 ± 2.9 vs. 28.1 ± 3.2	Placenta	at the time of delivery	healthy pregnant women	NR	NR	yes	yes
Nizyaeva 2018*(Nizyaeva et al., 2018) Russia	Cross-sectional	22 Total 12 EOPE 10 LOPE	15 Total 10 late normal 5 early normal	NR	placenta (syncytiotrophoblast) endothelium	at the time of delivery	Late normal pregnancies defined as women with physiological course of pregnancy and full-term gestational age. Early normal pregnancies defined as women with preterm operative delivery at 26–31 gw	NR	NR	NR	NR
Shen 2018 (Shen et al., 2018) China	Case-control	10	10	29.11 ± 5.01 vs. 27.56 ± 3.21	MPB (serum exosomes)	before delivery (prior to treatment)	gestational age-matched normal pregnant women	Gestational age	NR	NR	yes
Timofeeva 2018 (Timofeeva et al., 2018) Russia	Cohort	28 Total 16 EOPE 2 moderate EOPE 14 severe EOPE 12 LOPE 11 moderate LOPE 1 severe LOPE	26 Total 16 full term 10 indicated for Caesarean	NR	Placenta MPB (plasma)	at the time of delivery	women with full term physiological pregnancy (37–40 gw) and pregnant women with an indication for an emergency Caesarean section due to the lack of prolonging the pregnancy because of cervical insufficiency, placental abruption, or premature rupture of the fetal membrane without clinical manifestations of PE	NR	NR	NR	NR
	Cohort	6 sEOPE	10	NR	MPB (plasma exosomes)	before delivery (11-13 gw, 24–26 gw and 30–32 gw)	women with physiological pregnancy	NR	NR	NR	NR
Wang 2018 (Wang and Yan, 2018) China	Cross-sectional	20	20	29.7 ± 2.4 vs. 28.6 ± 3.2	Placenta	at the time of delivery	pregnant women with normal term pregnancy (without PE or other complications)	NR	NR	NR	NR
Wang 2018 (Wang et al., 2018a) China	Cross-sectional	9	8	34.8 ± 1.4 vs. 34.3 ± 2.2	MPB (plasma)	at the time of delivery	preterm labor control defined an uniparous gestation in a previously normotensive woman who did not exhibit any gestational complication and delivered a healthy newborn of gestational age before 37 weeks of pregnancy	Gestational age	no	NR	yes
Wang 2018*(Wang et al., 2018b) China	Case-control	10	10	31.3 ± 4.84 vs. 30.5 ± 4.37	Placenta	NR	normal pregnancies	NR	NR	NR	NR
Wang 2018 (Wang et al., 2018c) Australia	Case-control	16 Total 8 EOPE 8 LOPE	48 Total 8 term controls 7 at 10–11 gw 8 at 14.3–17.8 gw 8 preterm controls	NR	Placenta	at the time of delivery	Term controls defined as uncomplicated singleton pregnancies delivering at term (38.2–40.4 weeks gestation) by elective Caesarean section in the absence of labor. Women treated with non-steroidal anti-inflammatory drugs or who had a history of infection, chorioamnionitis, PE, or who were undergoing induction of labor, were excluded from this group. Women undergoing elective terminations of pregnancy at 10–11 gw or 14.3–17.8 gw. Preterm controls defined as equivalent gestational age women who delivered preterm (at 31.6–35.1 gestational weeks) after spontaneous labor/rupture of membranes and vaginal delivery with no evidence of hypertension	Gestational age	NR	NR	NR
Winger 2018 (Winger et al., 2018) United States	Not clear (retrospective study)	4	20	30.9 ± 8.8 vs. 33.3 ± 6.5	MPB (buffy coat)	before delivery (11–13 gw)	Normal delivery defined as the delivery of a singleton, normal karyotype baby with the following pregnancy criteria: delivery at 38 ± 42 weeks gestation, baby weight within the normal range for gestational age and maternal BMI <30	NR	NR	NR	NR
Zou 2018 (Zou et al., 2018) China	Cross-sectional	15	18	NR	placenta (basal plate) placenta (chorionic plate)	NR	normal pregnant women	NR	NR	NR	NR
Awamleh 2019 (Awamleh et al., 2019) Canada	Case-control	19	20	28.6 ± 7.0 vs. 28.2 ± 5.0	placenta (villi)	at the time of delivery	gestational age- matched patients with preterm labor and no other complications before 34 weeks of gestation	Gestational age	NR	NR	yes
Biro 2019*(Biró et al., 2019) Hungary	Cross-sectional	21 Total 8 13	15 Total 8 7	33.43 ± 6.48 vs. 31.25 ± 5.80	MPB (plasma) placenta	before delivery (3rd trimester) at the time of delivery	normotensive group with the exclusion of women with history of pregnancy-related or other forms of hypertension, spontaneous abortion, preterm birth, and intrauterine growth restriction	NR	NR	NR	NR
Chen 2019*(Chen et al., 2019) China	Cross-sectional	29	27	31 ± 7 vs. 26 ± 6	Placenta	at the time of delivery	pregnant women with normal uncomplicated pregnancies (≥36 weeks of gestation)	NR	no	NR	yes
Devor 2019 (Devor et al., 2020) United States	Case-control	4	5	35.8 ± 2.8 vs. 29.2 ± 2.1	MPB (plasma exosomes)	before delivery (in each trimester)	matched healthy controls who underwent a normal spontaneous vaginal delivery	Yes (no variable)	NR	NR	NR
Dong 2019*(Dong et al., 2019) China	Case-control	40 Total 20 EOPE 20 LOPE	40 Total 20 early control 20 late control	29.10 ± 6.03 (EOPE) 29.15 ± 5.13 (LOPE) vs. 29.6 ± 4.88 (early controls) 30.05 ± 4.91 (late controls)	MPB (plasma)	before delivery (prior to any surgery) at the time of delivery (for PE patients)	Early controls defined as 20–34 gestational week normal pregnant women who underwent routine outpatient antenatal examinations and did not develop preeclampsia. Late controls defined as 34–41 gestational week normal pregnant women who underwent routine outpatient antenatal examinations and did not develop preeclampsia	Gestational age	no	yes	yes
Eghbal-Fard 2019 (Eghbal-Fard et al., 2019) Iran	Case-control	50	50	33.2 ± 5.1 vs. 31.8 ± 3.4	MPB (mononuclear cells)	before delivery	healthy gestational matched pregnant women	Gestational age	NR	NR	NR
Hocaoglu 2019*(Hocaoglu et al., 2019) Turkey	Case-control	23 Total 6 mEOPE 6 sEOPE 5 mLOPE 6 sLOPE	28	29.8 ± 5.9 (Total) vs. 28.1 ± 5.8	MPB (leukocytes)	before delivery	no obstetrical or medical complications whose gestational weeks were matched	Gestational age	no	no	yes
Hromadnikova 2019a*(Hromadnikova et al., 2019b) Czech Republic	Not clear (cohort case-control study)	101 Total 24 mPE 77 sPE	89	32 (21–44) at delivery 38 (28–52) at follow-up vs. 32 (25–43) at delivery 38 (29–50) at follow-up	MPB	after delivery (3–11 years postpartum)	normal gestation	NR	no	no	NR
Hromadnikova 2019b (Hromadnikova et al., 2019a) Czech Republic	Nested case-control	43 Total 13 mPE 30 sPE 10 EOPE 33 LOPE	102 Total 50 control 1 52 control 2	32.34 ± 0.73 Total vs. 31.88 ± 0.56 (control 1) 31.21 ± 0.56 (control 2)	MPB (plasma exosomes)	before delivery (10–13 gw)	normal pregnancies without complications delivering full term, healthy infants after 37 weeks of gestation weighting >2,500 g, were selected for equal gestational age, equal age of women at the time of sampling and equal plasma sample storage times	NR	no	NR	NR
Hu 2019 (Hu et al., 2019) China	Cross-sectional	25	25	29.24 ± 4.05 vs. 28.04 ± 3.09	Placenta	NR	normal pregnancy	NR	NR	NR	NR
Huang 2019 (Huang et al., 2019) China	Cross-sectional	20	20	29.6 (5.8) vs. 31.3 (4.6)	Placenta	at the time of delivery	normotensive pregnant women	NR	yes	NR	NR
Li 2019 (Li et al., 2019) China	Cross-sectional	10	10	27.92 ± 3.94 (23–34) vs. 28.00 ± 3.54 (22–34)	Placenta	at the time of delivery	healthy controls	NR	NR	NR	NR
Liu 2019 (Liu et al., 2019a) China	Cross-sectional	20	20	NR	Placenta	at the time of delivery	normal pregnant women	NR	NR	NR	NR
Liu 2019 (Liu et al., 2019b) China	Cross-sectional	39	42	NR	Placenta	NR	normal pregnant women	NR	NR	NR	NR
Liu 2019 (Liu et al., 2019c) China	Cross-sectional	30	30	27.07 ± 2.53 vs. 28.67 ± 2.78	Placenta	at the time of delivery	normal pregnant women	NR	NR	NR	yes
Ma 2019 (Ma et al., 2019) China	Not clear (prospective study)	89	70	27.25 vs. 26.81	MPB (serum)	before delivery (20 gw)	pregnant women with no evident anomalies detected during physical examinations	NR	NR	no	yes
Martinez-Fierro 2019 (Martinez-Fierro et al., 2019) Mexico	Nested case-control	30 Total 6 12 gw 10 16 gw 14 20 gw	18	23.5 ± 5.1 vs. 23.4 ± 5.8	MPB (serum)	before delivery (at the time of PE diagnosis, and at the 12th, 16th and/or 20th gw)	healthy pregnancies without complications matched by age, nulliparity, body mass index (BMI), and a personal and family history of PE	Maternal age, nulliparity, BMI and personal and family history of PE	no	yes	NR
Mei 2019 (Mei et al., 2019) China	Cross-sectional	20	20	NR	Placenta	at the time of delivery	normal pregnant women	NR	NR	NR	NR
Nejad 2019 (Nejad et al., 2019) Iran	Case-control	20	20	29 ± 1.1 vs. 28 ± 0.92	MPB (plasma)	NR	healthy controls matched for BMI (body mass index, 29–39 kg/m2), ethnicity (Iranian), smoking (non-smoker)	BMI (29–39 kg/m^2^), ethnicity (Iranian), smoking (non-smoker)	NR	yes	yes
Pillay 2019 (Pillay et al., 2019) South Africa	Case-control	30 Total 15 EOPE 15 LOPE	15 Preterm controls (≤33 gw) 15 Term controls (≥34 gw)	25.25 ± 5.13 (EOPE) 27.11 ± 5.23 (LOPE) vs. 28.43 ± 2.23 (≤33 gw) 26.12 ± 3.62 (>34 gw)	MPB (plasma exosomes)	before delivery (at the time of clinical diagnosis of PE)	Gestationally matched normotensive pregnant woman (blood pressure of 120 ± 10/80 ± 5 (systolic/diastolic mm Hg) with absent proteinuria as detected by a rapid urine dipstick test)	Gestational age	NR	NR	NR
Sekar 2019 (Sekar et al., 2019) India	Cross-sectional	NR	NR	NR	MPB	NR	Normotensives	NR	NR	NR	NR
Shi 2019 (Shi et al., 2019) China	Cross-sectional	15	15	29.5 ± 2.8 vs. 28.3 ± 3.7	placenta	at the time of delivery	Normal-term pregnancies without PE or any other complications	NR	NR	NR	NR
Tang 2019 (Tang et al., 2019) China	Case-control	30	30	27.8 (24.5–31.0) vs. 27.3 (25.0–28.0)	placenta	at the time of delivery	healthy pregnant women with uncomplicated pregnancies	Gestational age	no	yes	yes
Wang 2019 (Wang et al., 2019a) Taiwan	Case-control	33	55	34.02 ± 5.57 vs. 31.33 ± 4.31	MPB (plasma)	before delivery (prepartum after hospital admittance for delivery)	healthy controls	NR	no	NR	NR
Wang 2019 (Wang et al., 2019b) China	Cross-sectional	20	20	Individual data 29.00 ± 3.82 vs. 27.50 ± 3.35	Placenta MPB (serum)	at the time of delivery	normal controls	NR	NR	NR	NR
Wang 2019 (Wang et al., 2019e) China	Cross-sectional	42	39	28.9 ± 2.1 vs. 29.1 ± 1.9	Placenta MPB (serum exosomes)	NR	normal pregnancies	NR	NR	NR	NR
Wang 2019*(Wang et al., 2019c) China	Case-control	17	17	28.1 ± 0.8 vs. 29.7 ± 1.2	placenta	at the time of delivery	normotensive healthy nulliparous and nonproteinuric during pregnancy matched for age and BMI	Maternal age and BMI	NR	yes	NR
Wang 2019 (Wang et al., 2019d) China	Cross-sectional	30	30	28.2 ± 3.2 vs. 28.9 ± 3.0	placenta	at the time of delivery	healthy pregnant women	NR	NR	NR	yes
Xiaobo 2019 (Xiaobo et al., 2019) China	Cross-sectional	15 Total 10 EOPE 5 LOPE	15	30.2 ± 5.4 vs. 29.3 ± 4.7	placenta	at the time of delivery	healthy pregnant women	NR	NR	NR	yes
Xie 2019 (Xie et al., 2019) Chin	Cross-sectional	57	57	27.12 ± 4.11 vs. 26.37 ± 3.29	placenta	NR	healthy patients	NR	NR	NR	NR
Xue 2019 (Xue et al., 2019) China	Case-control	20	20	28.55 ± 0.83 vs. 27.00 ± 0.68	Placenta MPB (serum)	at the time of delivery NR	women without renal disease, cardiovascular disease, transient hypertension in pregnancy, gestational diabetes mellitus, hepatitis, any evidence of spontaneous abortion, intrauterine fetal death, fetal chromosomal or other pregnancy complications	Maternal age and gestational age	no	NR	NR
Yang 2019*(Yang et al., 2019a) China	Cross-sectional	57 Total preterm PE 12 term PE 14 31 plasma	32 Total preterm age matched control 11 term age matched control 12 9 plasma	31.57 ± 2.98 vs. 32.83 ± 3.19	Placenta MPB (plasma)	at the time of delivery	Early trimester controls – patients undergoing terminated pregnancies through dilation and curettage procedure	Maternal age	no	NR	NR
Yang 2019a (Yang and Guo, 2019) China	Cross-sectional	30	30	28.63 ± 2.24 vs. 28.83 ± 2.42	placenta	at the time of delivery	control group	NR	NR	NR	yes (essential HTA)
Yang 2019b (Yang and Meng, 2019) China	Cross-sectional	30	30	27.80 ± 2.10 vs. 28.20 ± 1.50	placenta	at the time of delivery	normal group	NR	NR	yes	yes
Yang 2019a (Yang et al., 2019b) China	Cross-sectional	40	40	30.5 ± 5.3 vs. 30.9 ± 4.6	placenta	at the time of delivery	healthy controls	NR	NR	NR	NR
Yang 2019b (Yang et al., 2019c) China	Cross-sectional	57	70	73.8 ± 3.3 vs. 67.2 ± 2.6	placenta	at the time of delivery	normal controls	NR	yes	NR	yes
Youssef 2019*(Youssef and Marei, 2019) Egypt	Cross-sectional	30 Total mPE 12 sPE 18	20	31.77 ± 3.16 vs. 29.75 ± 4.24	MPB (serum)	before delivery	healthy pregnant women without any pregnancy complications who came for delivery between 38 and 40 weeks of gestation	NR	no	NR	yes
Zhong 2019 (Zhong et al., 2019) China	Cross-sectional	3	3	NR	MPB (plasma)	before delivery	normal pregnancies	NR	NR	NR	NR
Ayoub 2019* (Ayoub et al., 2019) Egypt	Cross-sectional	80	80	30.5 (21–41) vs. 32 (19.42)	MPB (serum)	At the time of diagnosis of PE	Normal pregnancies	No	NR	Yes	Yes
Cao 2019 (Cao et al., 2019) China	Cross-sectional	25	28	29.78 ± 5.25 vs. 30.45 ± 4.62	Placenta MPB (plasma)	NR	Normal pregnancies	No	NR	NR	NR
Demirer 2019 (Demirer et al., 2020) Turkey	Not clear (prospective study)	96 total 48 EOPE 48 LOPE	23 + 3 early stage 3 late stage	30.12 ± 5.7 Total 31.0 ± 5.5 EOPE 29.4 ± 5.8 LOPE	MPB	Before delivery	Healthy pregnant women with no obstetrical or medical complications	No	No	No	Yes
Lip 2019 (Lip et al., 2020) Netherlands	Cross-sectional	10 EOPE	10	31.5 ± 5.7 vs. 28.0 ± 4.4	MPB (plasma)	At the time of PE diagnosis	Healthy pregnant women	Gestational age at sampling	NR	No	Yes
Lv 2019 (Lv et al., 2019) China	Cross-sectional	18	18	32.94 ± 4.64 vs. 31.06 ± 4.02	Placenta	At the time of delivery	Normal singleton pregnant women by Caesarean	No	NR	NR	Yes
Qian 2019 (Qian and Liu, 2019) China	Cross-sectional	16	16	29.3 ± 2.5 vs. 28.4 ± 3.1	Placenta (villi)	At the time of delivery	Normal pregnant women	No	NR	NR	NR
Xu 2019 (Xu et al., 2019) United States	Cross-sectional	6	6	29 ± 6 vs. 29 ± 7	Maternal subcutaneous adipose tissue	At the time of delivery	Normal pregnant women	No	NR	Yes	NR
Yang 2019 (Yang and Meng, 2020) China	Case-control	30	30	28.30 ± 2.07 vs. 29.00 ± 1.55	Placenta	At the time of delivery	Normal full term pregnancy	No	Yes	Yes	Yes
Yuan 2019* (Yuan et al., 2020) China	Cross-sectional	30	30	27.8 ± 2.8 vs. 26.52 ± 4.9	Placenta	At the time of delivery	Normal pregnancies	No	NR	NR	NR
Zhang 2019 (Zhang et al., 2019b) China	Cross-sectional	30	30	28.36 ± 4.78 vs. 24.34 ± 2.87	MPB (serum)	At the time of delivery	Healthy pregnancies	No	NR	NR	NR
Akgor 2020* (Akgör et al., 2021) Turkey	Cross-sectional	31	32	29.9 ± 6.66 vs. 29.47 ± 6.33	MPB (plasma)	Before delivery	Term-matched healthy pregnancies	Gestational age, BMI, additional comorbities, parities, age	No	NR	NR
Devor 2020 (Devor et al., 2020) United States	Case-control	4 LOPE	5	35.8 ± 2.8 vs. 29.2 ± 2.1	MPB (plasma)	Before delivery (1st trimester -before 13 GW 2nd trimester -13-26 GW 3rd trimester -26-40 GW)	Matched healthy controls	Mmaternal age, BMI	NR	NR	Yes
Dong 2020*(Dong et al., 2020) China	Cross-sectional	20	20	31.7 ± 3.2 vs. 29.7 ± 2.3	MPB Placenta	Before delivery At the time of delivery	Women without PE	No	NR	NR	Yes
Fan 2020* (Fan et al., 2020) China	Cross-sectional	25	25	27.92 ± 2.81 vs. 26.84 ± 2.30	Placenta	At the time of delivery	Normal pregnant women without any other complications, such as premature rupture of membranes, fetal anomalies, maternal history of hypertension and/or renal or cardiac disease, maternal infection, or smoking	No	NR	Yes	Yes
Gong 2020 (Gong et al., 2020) China	Cross-sectional	8	8	31 ± 4.3 vs. 30 ± 4.5	Placenta	At the time of delivery	Healthy pregnancies	No	NR	NR	Yes
Han 2020 (Han et al., 2021) China	Cross-sectional	60 Total 30 severe EOPE 30 mild EOPE 20 PE	30 20	31.56 ± 4.76 Severe EOPE vs. 30.34 ± 4.28 Mild EOPE 31.18 ± 4.16 vs. 30.86 ± 4.72	MPB (serum) UCB Placenta	At the time of delivery	Normal pregnancies	Gestational age, maternal age	NR	Yes	Yes
Huang 2020 (Huang et al., 2020) China	Cross-sectional	46 sPE	57	29.6 ± 3.9 vs. 28.5 ± 4.1	Placenta	At the time of delivery	pregnant women without any pregnancy complications (34–40 gestational weeks)	Gestational age, BMI, maternal age	NR	NR	Yes
Jelena 2020 (Jelena et al., 2020) Serbia	Case-control	19	17	34 (20–51) vs. 32 (22–40)	MPB (plasma)	At the time of delivery	Healthy pregnant women	No	NR	No	Yes
Kim 2020 (Kim et al., 2020) South Korea	Case-control	92	92	32.73 ± 0.54 vs. 31.49 ± 0.50	MPB (serum)	Before delivery	Normotensive pregnant women selected at random	No	NR	NR	Yes
Li W 2020 (Li et al., 2020d) China	Cross-sectional	30	30	NR	Placenta	At the time of delivery	Healthy	No	NR	NR	NR
Li T 2020* (Li et al., 2020c) China	Case-control	30 sPE	20	25.45 ± 3.03 vs. 25.27 ± 3.19	Placenta	At the time of delivery	Healthy pregnant women	No	yes	NR	Yes
Li Q 2020 (Li et al., 2020b) China	Nested case-control	15	29	31.13 ± 1.24 vs. 30.62 ± 0.72	MPB (plasma) Placenta	Before delivery (between 12 + 0 and 13 + 6 GW) At the time of delivery	Gestational age matched healthy pregnancies without any other complications during pregnancy	Gestational age	NR	NR	Yes
Li H 2020 (Li et al., 2020a) China	Cross-sectional	24	24	NR	Placenta	At the time of delivery	Healthy Pregnancies	No	NR	NR	Yes
Licini 2020 (Licini et al., 2021)li Russia	Nested case-control	13 10	18 20	33 (31; 34) 36.9 ± 5.25 vs. 30.2 ± 7.59 1st trimester 32.6 ± 4.05 3rd trimester	MPB (plasma) Placenta	Before delivery (12th GW) At the time of delivery	Healthy pregnant women (normal uterine and umbilical Doppler flow velocimetry during gestation and where the foetus was appropriate for the gestational age (newborns _10th _ 90th percentile for gender and gestational age according to Italian charts) Voluntary terminations in the 1st trimester, and healthy term prgnancies	Gestational age	NR	No	Yes
Ma 2020 (Ma et al., 2020) China	Cross-sectional	36	30	NR	Placental monocytes MPB (serum exosomes)	NR	Normal pregnant volunteers	No	NR	NR	NR
Mavreli 2020 (Mavreli et al., 2020) Greece	Case-control	17 LOPE 5 for NGS 12 for qRT-PCR	17 5 for NGS 12 for qRT-PCR	31.81 (21.2–39.50) vs. 33.19 (26.75–41.27)	MPB (plasma)	Before delivery (1st trimester)	Uncomplicated pregnancies delivered at 38–42 GW, chromosomally normal baby weighing within the normal range for gestational age, matched for maternal age, gestational age and duration of storage of plasma samples	Maternal age, gestational age, duration of storage plasma samples	NR	No	Yes
Sheng 2020 (Sheng et al., 2020) China	Case-control	200	200	31.19 ± 4.84 vs. 31.02 ± 4.26	MPB (plasma)	NR	Healthy pregnant women	No	NR	Yes	Yes
Song 2020 (Song et al., 2020) China	Cross-sectional	24	24	NR	Placenta	At the time of delivery	Healthy pregnant women	No	NR	NR	NR
Tao 2020 (Tao et al., 2020) China	Cross-sectional	35	35	28.31 ± 2.86 vs. 28.66 ± 3.0	Placenta	At the time of delivery	Normal pregnancies	No	NR	NR	NR
Wang 2020 (Wang et al., 2020) China	Cross-sectional	24	24	NR	Placenta	At the time of delivery	Healthy pregnancies	No	NR	NR	NR
Whigham 2020 (Whigham et al., 2020) Australia	Case-control	34 PE 36 GW 43 PE 28 GW 32 sEOPE 34 LOPE	196 Controls 36 GW 91 Controls 28 GW 22 gestation matched preterm 12 gestation matched term	28 GW 31 (36–34) vs. 32 (29–34.8) 36 GW 31 (28–33) vs. 31 (26.5–36.3)	MPB (whole blood) Placenta	Before delivery (28 GW) At the time of delivery	Pre-term controls - pre-term rupture of membranes, placenta praevia or antepartum haemorrhage without any evidence of infection (histopathological examination of the placentas), hypertensive disease or maternal comorbidities. Term controls – healthy pregnancies matched fo gestational age	Gestational age	NR	NR	Yes
Wu 2020 (Wu et al., 2020a) China	Cross-sectional	30	30	31.2 ± 4.8 vs. 28.6 ± 5.7	Placenta	At the time of delivery	Healthy pregnant women	No	NR	NR	Yes
Wu 2020 (Wu et al., 2020b) China	Cross-sectional	64 Total 26 mPE 28 sPE	35	NR	Placenta	At the time of delivery	Healthy pregnant women	No	NR	NR	NR
Xueya 2020 (Xueya et al., 2020) China	Cross-sectional	18	20	32.5 ± 1.25 vs. 32.1 ± 0.75	UCB (exosomes) MPB (plasma exosomes) Placenta	After childbirth After PE diagnosis At the time of delivery	Healthy donors	No	NR	NR	NR
Yang 2020 (Yang et al., 2021) China	Cross-sectional	20	20	NR	Placenta UCMSC	At the time of delivery	Normotensive pregnant women	No	NR	NR	Yes
Zhao 2020 (Zhao et al., 2020) China	Case-control	30	30	NR	Placenta	At the time of delivery	Normal pregnancies	No	NR	NR	NR
Zheng W 2020 (Zheng et al., 2020) China	Cross-sectional	30 sPE	20	28.2 ± 2.1 vs. 27.3 ± 1.9	Placenta MPB (serum)	At the time of delivery	Healthy pregnant women	No	NR	NR	Yes
Zhou 2020 (Zhou et al., 2020) China	Cross-sectional	32	28	32 ± 4.6 vs. 33 ± 3.9	MPB (serum) Placenta	At the time of delivery	Normal pregnant women	Maternal age, gestational age, pre-pregnancy indices	NR	NR	NR
Zhu 2020 (Zhu et al., 2020) China	Cross-sectional	30	30	NR	Placenta	At the time of delivery	Normal full term pregnancies	No	Yes	NR	NR
Ali 2021 (Ali et al., 2021) Pakistan	Cross-sectional	27	27	26 (23–30) vs. 25 (22–28)	MPB (serum)	At the time of delivery	Healthy pregnant women with normal blood pressure (BP) and comparable age in the final trimester (28–40 weeks)	Maternal age, gestational age	NR	Yes	Yes
Brodowski 2021 (Brodowski et al., 2021) Germany	Cross-sectional	12 (6 UCB +6 MPB samples)	9 (6 UCB +6 MPB samples)	UCB ECFC 31.5 ± 3.7 vs. 32.8 ± 5.2 MPB ECFC30.8 ± 5.5 vs. 31.7 ± 7.4	UCB (endothelial colony forming cells) MPB (endothelial colony forming cells)	Before delivery	Healthy uncomplicated pregnancies	Gestational age at delivery, BMI, and maternal age	No	NR	Yes
Cai 2021 (Cai et al., 2021) China	Cross-sectional	40	40	29.95 ± 2.67 vs. 28.00 ± 3.20	Placenta	At the time of delivery	Normal pregnancies defined as blood pressure or urine protein in the normal range within 35–40 weeks of pregnancy, followed by Caesarean delivery of healthy infants	No	NR	NR	Yes
Chu 2021 (Chu et al., 2021) China	Cross-sectional	18	28	28 ± 8 vs. 29 ± 6	Placenta	At the time of delivery After selective pregnancy termination (1st and 2nd trimester controls)	Normal term pregnancies 1st trimester (6–8 GW) controls 2nd trimester (18–21 GW) controls	No	No	NR	NR
Hayder 2021 (Hayder et al., 2021) Canada	Case-control	18 Total 14 Pre-term PE 4 Term PE	30 Total 13 Pre-term 17 Term	Pre-term 30.27 ± 0.36 vs. 29.83 ± 0.51 Term 37.25 ± 0.25 vs. 38.32 ± 0.14	Placenta	At the time of delivery	Pre-term controls 26–36 GW Term controls 37–40 GW	No	NR	NR	NR
Jairajpuri 2021 (Jairajpuri et al., 2021) Bahrain	Case-control	30 15 mPE 15 sPE	15	32 (29–35) mPE 33 (29–37) sPE vs. 30 (25–35)	MPB (plasma)	At the time of delivery	Healthy controls with no previous history of hypertension, cardiovascular disease, hepatitis, kidney disease, diabetes, and any evidence of intrapartum infection or other complications of pregnancy such as fetal anomalies or chromosomal abnormalities in the third trimester	No	NR	Yes	Yes
Liu 2021 (Liu et al., 2021) China	Cross-sectional	30 EOPE	30	30.77 ± 5.75 vs. 32.10 ± 4.96	Placenta	At the time of delivery	Helathy pregnancies who had chosen Caesarean section because of abnormal fetal position, pelvic stenosis, or social factorsetc.	No	No	NR	NR
Kamali Simsek, 2021 (Kamali Simsek et al., 2021) Turkey	Cross-sectional	7	7	31.3 ± 5.02 vs. 28.2 ± 4.7	Placenta (hDMSC)	At the time of delivery	Healthy pregnant women	Gestational age	NR	NR	NR
Kolkova 2021 (Kolkova et al., 2021) Slovakia	Case-control	27 Total 13 mPE 11 sPE 7 EOPE 17 LOPE	32 (29 used for miRNA analysis)	27 (21–50) vs. 30 (25–37)	MPB (plasma)	Before delivery	Normal pregnancies with no pregnancy complications, such as artificial insemination, threatened abortion, premature rupture of membranes and/or premature birth, placenta praevia, and foetal macrosomia	No	NR	NR	Yes
Liao 2021 (Liao et al., 2021) China	Case-control	70 EOPE 33 sEOPE 37 mEOPE	35	28.6 ± 2.2 sEOPE 27.9 ± 3.1 mEOPE vs. 28.2 ± 2.9	MPB (serum)	Before delivery	Normal pregnant women	No	NR	NR	Yes
Luizon 2021 (Luizon et al., 2021) Brasil	Nested case-control	5 sPE	5	29.8 ± 2.0 vs. 28.8 ± 2.6	MPB (plasma)	Before delivery	Healthy pregnancies	No	NR	NR	Yes
Mao 2021 (Mao et al., 2021) China	Case-control	24	21	32.21 ± 4.51 Vs. 34.23 ± 3.29	Placenta	At the time of delivery	Normal pregnancies	Maternal age, maternal weight, systolic blood pressure mmHg, diastolic blood pressure mmHg, proteinuria g/day, body weight of infant g, Gestational age	NR	Yes	NR
Martinez-Fierro 2021 (Martinez-Fierro and Garza-Veloz, 2021) Mexico	Nested case-control	16	18	23.5 ± 5.1 vs. 23.4 ± 5.8	MPB (serum)	Before delivery (12, 16, 20 GW) At the time of PE diagnosis	Healthy pregnancies without complications	No	No	NR	Yes
Peng 2021 (Peng et al., 2021) China	Cross-sectional	30	30	30.2 ± 5.1 vs. 30.5 ± 4.8	Placenta	At the time of delivery	Normal pregnant women	No	NR	NR	Yes
Witvrouwen 2021* (Witvrouwen et al., 2021) Belgium	Cross-sectional	24 EOPE	30	28.5 (26.7–30.9) vs. 29.2 (27.4–32.5)	MPB (plasma)	At the time of PE diagnosis (22–36 GW)	Healthy pregnancies free from medication and did not have a history of PE, (pregnancy-induced) hypertension, cardiovascular disease or other chronic conditions	No	No	No	No
Xu 2021 (Xu et al., 2021) China	Cross-sectional	35 Total 20 EOPE 15 sPE	38	30.92 ± 1.89 EOPE 31.27 ± 3.85 sPE vs. 30.67 ± 2.56	Placenta	At the time of delivery	Healthy pregnant women	No	NR	NR	NR
Yu 2021 (Yu et al., 2021) China	Case-control	40 sPE	40	NR	Placenta	At the time of delivery	Control pregnancies	No	NR	NR	Yes
Zhao X 2021a* (Zhao et al., 2021a) China	Cross-sectional	10	10	29.73 ± 4.2 vs. 28.85 ± 3.9	Placenta	At the time of delivery	normal pregnant women were: 1) healthy subjects; 2) successful pregnancy, normal blood pressure and negative proteinuria	No	NR	NR	Yes
Zhao X 2021b (Zhao et al., 2021b) China	Case-control	25	25	28.91 ± 5.42 vs. 26.73 ± 4.34	Placenta	At the time of delivery	Normal pregnant women	No	NR	NR	Yes
Zhu 2021 (Zhu and Liu, 2021) China	Cross-sectional	21	21	34.1 ± 5 vs. 33.5 ± 4	MPB (serum)	NR	Normal pregnant women defined as i) Healthy subjects; ii) delivery after 37 weeks; iii) successful pregnancy without any complications, normal blood pressure and negative proteinuria	No	NR	NR	Yes
Zolfaghari 2021 (Zolfaghari et al., 2021) Iran	Case-control	25	25	29.2 ± 4.38 vs. 28.12 ± 3.84	MPB (mononuclear cells)	Before delivery	Healthy age-matched pregnant women at 28–38 weeks of gestation with no sign of historical disorders were engaged for this study	Maternal age	NR	NR	Yes

a Expressed as mean ± sd, mean ± sd (min-max), mean (min-max), mean ± se, med (min-max), med (25–75 percentile), med (Q1; Q3), or as individual data, as stated in the original article.

GW, gestational week; mPE, mild PE; sPE, severe PE; MPB, maternal peripheral blood; mEOPE, mild early onset PE; sEOPE, severe early onset PE; mLOPE, mild late onset PE; sLOPE, severe late onset PE; BMI, body mass index; MSC, mesenchymal stem cells; UC, umbilical cord; NR, not reported; UCMSC, umbilical cord mesenchymal stem cells; UCB, umbilical cord blood; hDMSC, decidual derived mesenchymal stem cells; GHTA, gestational hypertension; Lbs, pounds; HELLP, Hemolysis, elevated Liver enzymes and Low Platelets; HTA, hypertension; GDM, gestational diabetes mellitus; CVS, chorionic villus sampling.

Disease severity was reported in 70/229 publications. Details regarding PE definitions and the diagnostic criteria used in the original articles are presented in Supplementary Tables S2, S3. qRT-PCR as the detection method with U6 as an internal control was utilized in almost all studies, and the details regarding quantification methods and housekeeping genes used are presented in Supplementary Table S4. A list of all explored miRNAs from the included publications according to PE severity (more severe, less severe, and not-specified PE) is presented in Supplementary Tables S5–S7.

### Meta-Analysis

A meta-analysis was performed for the following fourteen miRNAs: miRNA-16, miRNA-17, miRNA-17-5p, miRNA-20b, miRNA-23a, miRNA-29a-3p, miRNA-29b, miRNA-30a-3p, miRNA-155, miRNA-155-5p, miRNA-181a, miRNA-195, miRNA-210, and miRNA-376c.

**FIGURE 2:**
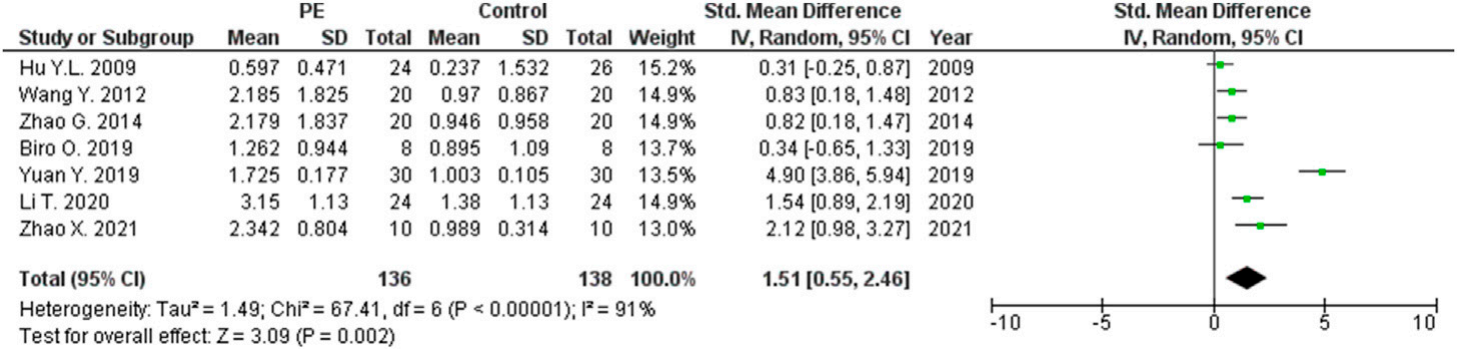
Meta-analysis of differences in expression level of miRNA-16 in placenta between women with vs. without preeclampsia.

**FIGURE 3:**
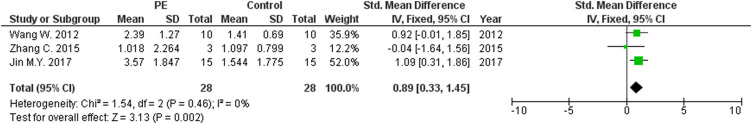
Meta-analysis of differences in expression level of miRNA-20b in placenta between women with vs. without preeclampsia.

**FIGURE 4:**
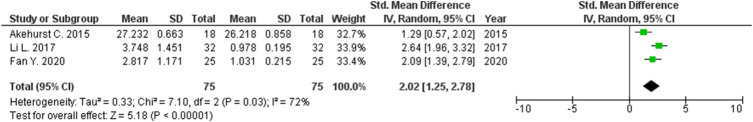
Meta-analysis of differences in expression level of miRNA-23a in placenta between women with vs. without preeclampsia.

**FIGURE 5:**
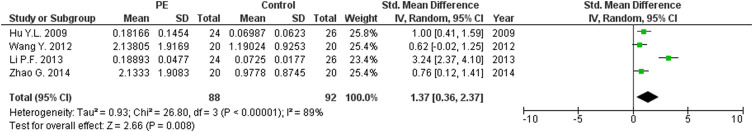
Meta-analysis of differences in expression level of miRNA-29b in placenta between women with vs. without preeclampsia.

**FIGURE 6:**
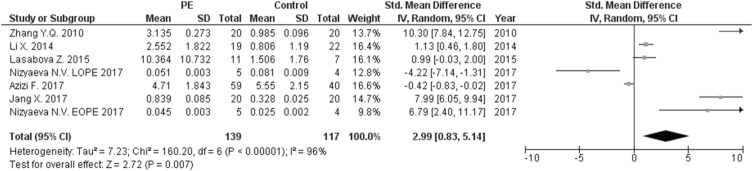
Meta-analysis of differences in expression level of miRNA-155 in placenta between women with vs. without preeclampsia.

**FIGURE 7:**
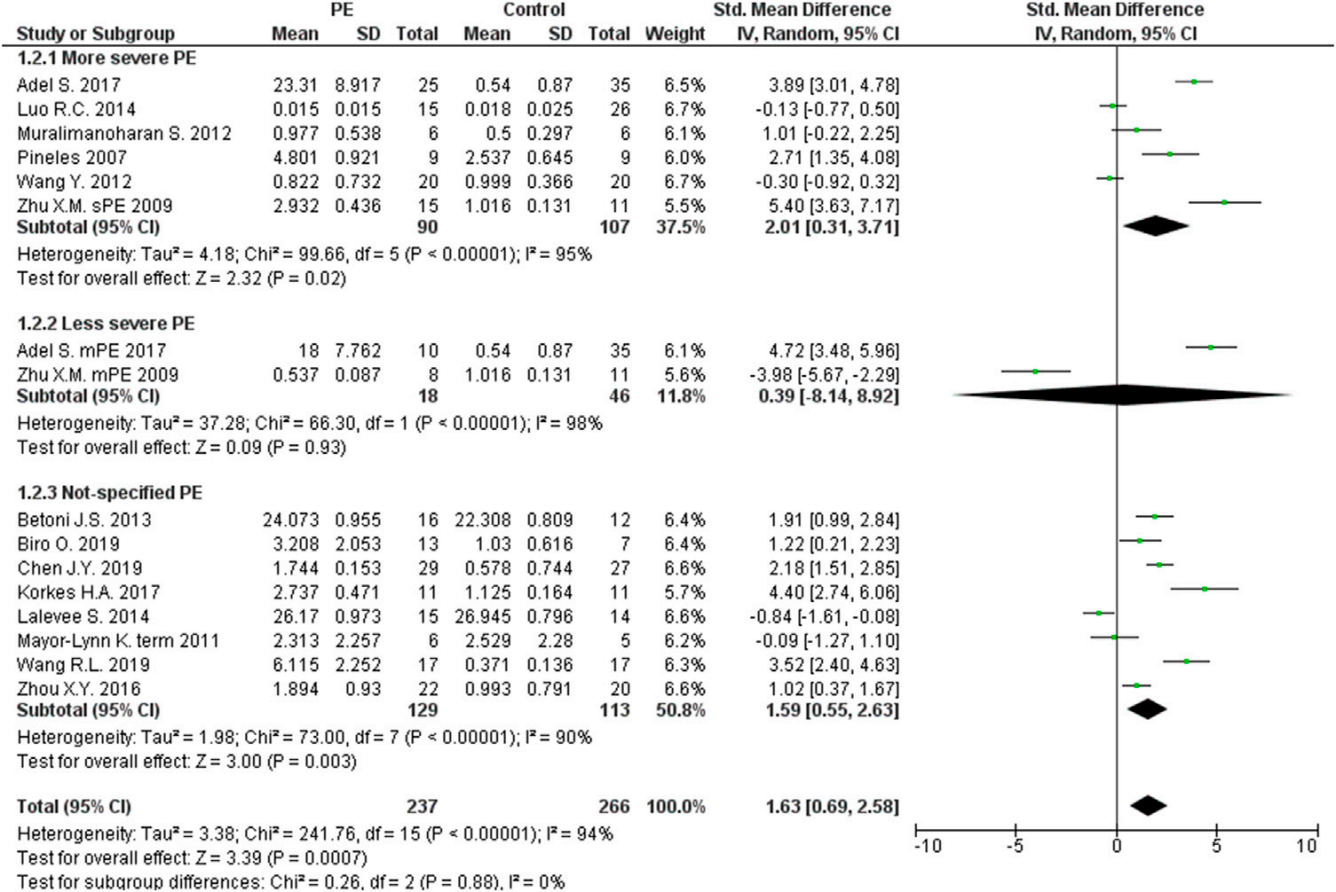
Meta-analysis of differences in expression level of miRNA-210 in placenta between women with vs. without preeclampsia.

**FIGURE 8:**
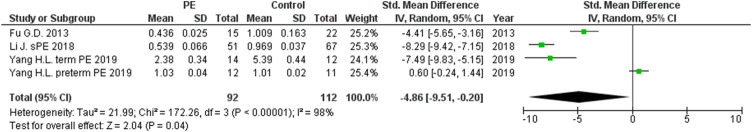
Meta-analysis of differences in expression level of miRNA-376c in placenta between women with vs. without preeclampsia.

The expression levels were significantly higher in the placentas of women with PE compared to women without PE for miRNA-16 (SMD = 1.51, 95%CI = 0.55–2.46, *p* = 0.002) (Figure 2), miRNA-20b (SMD = 0.89, 95%CI = 0.33–1.45, *p* = 0.002) (Figure 3), miRNA-23a (SMD = 2.02, 95%CI = 1.25–2.78, *p* < 0.001) (Figure 4), miRNA-29b (SMD = 1.37, 95%CI = 0.36–2.37, *p* = 0.008) (Figure 5), miRNA-155 (SMD = 2.99, 95%CI = 0.83–5.14, *p* = 0.007) (Figure 6) and miRNA-210 (SMD = 1.63, 95%CI = 0.69–2.58, *p* < 0.001) (Figure 7). Subgroup analysis showed increased levels of miRNA-210 expression in placentas of women with more severe (SMD = 2.01, 95%CI = 0.31–3.71, *p* = 0.020), but not in women with a less severe form of PE (SMD = 0.39, 95%CI = –8.14 = 8.92, *p* = 0.930), compared to women without PE (Figure 7). The expression levels in placenta were significantly lower in women with PE compared to women without PE for miRNA-376c (SMD = –4.86, 95%CI = –9.51 to –0.20, *p* = 0.040) (Figure 8).

**FIGURE 9:**
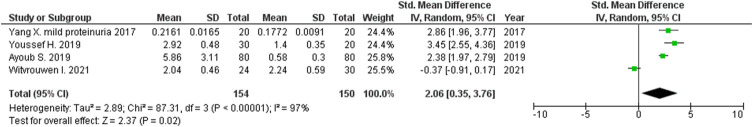
Meta-analysis of differences in expression level of miRNA-155 in peripheral blood between women with vs. without preeclampsia.

**FIGURE 10:**
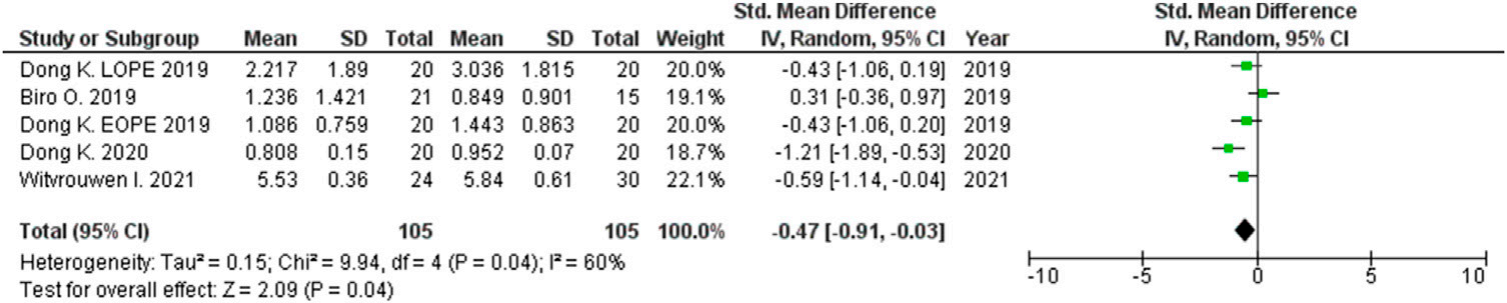
Meta-analysis of differences in expression level of miRNA-16 in peripheral blood between women with vs. without preeclampsia.

The expression level was significantly higher in the maternal peripheral blood of women with PE compared to women without PE for miRNA-155 (SMD = 2.06, 95CI = 0.35–3.76, *p* = 0.020) (Figure 9), but it was lower for miRNA-16 (SMD = –0.47, 95%CI = –0.91 to –0.03, *p* = 0.040) (Figure 10).

The functional roles of all significant miRNAs are presented in detail in Table 2. Although the roles of the evaluated miRNAs are confusing, special emphasis should be placed on the interpretation of the miRNAs known roles in controlling trophoblast proliferation, migration, invasion, apoptosis, differentiation, cellular metabolism, and angiogenesis.

**TABLE 2:** Functional roles of significant miRNAs.

miRNA	Placenta	Maternal peripheral blood	Role
**16**	↑	↓	https://www.genecards.org/cgi-bin/carddisp.pl?gene=MIR16-1
**20b**	↑	NA	https://www.genecards.org/cgi-bin/carddisp.pl?gene=MIR20B&keywords=miRNA-20b
**23a**	↑	NA	https://www.genecards.org/cgi-bin/carddisp.pl?gene=MIR23A&keywords=miRNA-23a
**29b**	↑	NA	https://www.genecards.org/cgi-bin/carddisp.pl?gene=MIR29B1&keywords=miRNA-29b
**155**	↑	↑	https://www.genecards.org/cgi-bin/carddisp.pl?gene=MIR155&keywords=miRNA-155
**210**	↑	NA	https://www.genecards.org/cgi-bin/carddisp.pl?gene=MIR210&keywords=miRNA-210
**376c**	↓	NA	https://www.genecards.org/cgi-bin/carddisp.pl?gene=MIR376C&keywords=miRNA-376c

Placental expression levels were not significantly different in women with PE compared to women without PE for miRNA-17 (SMD = 0.22, 95%CI = -1.35 to –1.79, *p* = 0.790) (Supplementary Figure S1), miRNA-30a-3p (SMD = 1.00, 95%CI = –0.50–2.50, *p* = 0.190) (Supplementary Figure S2), miRNA-181a (SMD = 0.05, 95%CI = –0.99–1.08, *p* = 0.930) (Supplementary Figure S3), and miRNA-195 (SMD = –0.16, 95%CI = –1.35–1.02, *p* = 0.780) (Supplementary Figure S4). The expression level was not significantly different in maternal peripheral blood in women with PE compared to women without PE for miRNA-17-5p (SMD = 0.08, 95%CI = –0.74–0.90, *p* = 0.850) (Supplementary Figure S5), miRNA-29a-3p (SMD = –0.29, 95%CI = –1.22–0.64, *p* = 0.540) (Supplementary Figure S6), miRNA-155-5p (SMD = –0.37, 95%CI = –1.07–0.33, *p* = 0.300) (Supplementary Figure S7), miRNA-181a (SMD = 0.22, 95%CI = –0.42–0.86, *p* = 0.500) (Supplementary Figure S8), and miRNA-210 (SMD = 0.48, 95%CI = –0.66–1.62, *p* = 0.410) (Supplementary Figure S9).

The same results were obtained when sensitivity analyses were performed to exclude studies with unspecified types of PE, to replace expression data obtained from the chorionic plate with those obtained from the basal plate, including/excluding different forms (more/less severe) of PE where possible (Supplementary Figures S10–S22).

## Discussion

We identified in this study seven differentially expressed miRNAs in the placentas of women with vs without PE. miRNA-16, miRNA-20b, miRNA-23a, miRNA-29b, miRNA-155, and miRNA-210 were significantly increased in the placentas of PE women, while the levels of miRNA-376c were significantly decreased in PE placentas. We found no differences in the expression levels of miRNA-17, miRNA-30a-3p, miRNA-181a, and miRNA-195 in placentas of PE vs. non-PE women. A meta-analysis of the miRNA expression levels in the peripheral blood of PE women compared to women without PE was performed for miRNA-16, miRNA-17-5p, miRNA-29a-3p, miRNA-155, miRNA-155-5p, miRNA-181a and miRNA-210. A significant decrease in miRNA-16 expression levels in maternal peripheral blood of PE women was found, and no differences were found for other evaluated miRNAs. A sensitivity analysis did not change the results of the primary analysis.

Placentation is thought to be the basis for normal physiological pregnancy and is required for fetal growth and development, as well as the expectation of term labor. Several sensitive, precisely dictated, vascular processes involving angiogenesis at the fetal-maternal interface and adequate cytotrophoblast invasion with spiral-artery remodeling are essential for placentation (Weedon-Fekjær et al., 2014). At the very beginning of a pregnancy in which PE will develop, the transformation of proliferative endothelium into invasive endothelium is absent, and the expected extensive invasion of cytotrophoblasts into the spiral arteries does not occur. This results in pathologic remodeling of the placental arterioles, which become narrow, with reduced flow and sclerotic changes in the arteriolar walls (Mouillet et al., 2015). Placental ischemia promotes an inflammatory state that is characterized by increased production of inflammatory cytokines by pro-inflammatory T cells, and a decrease in regulatory and anti-inflammatory cytokines (Hanna et al., 2000). Decreased levels of anti-inflammatory cytokines (IL-10, IL-4) and increased pro-inflammatory cytokines (TNF-α, IL-6) in the circulation and placental tissue support the inflammatory background of preeclampsia (Keiser et al., 2009; Spence et al., 2021). These processes lead to placental malnutrition, and subsequent development of PE. The placenta is known to be an organ in which a large number of miRNAs are expressed (Mouillet et al., 2015). Several miRNAs contribute to the processes of trophoblast proliferation, invasion, and differentiation. miRNA-125b-1-3p and miRNA-210 inhibit trophoblast proliferation and invasion, while miRNA-155 inhibits trophoblast invasion only. In contrast, miRNA-376c enhances trophoblast proliferation and invasion (Mouillet et al., 2015). Fu et al. demonstrated that miR-376c promotes trophoblast cell proliferation, survival, migration, and invasion, and postulated that inhibition of Nodal and TGF-β signaling by miR-376c is important for adequate placentation (Fu et al., 2013). Primate-specific C19MC miRNAs, which are almost exclusively expressed in placenta, were described as important factors influencing adequate trophoblast invasion and arterial remodeling (Hromadnikova et al., 2013; Mouillet et al., 2015). As knowledge of the functional importance of miRNAs in adequate placentation and the development of PE increases (Hayder et al., 2018), it becomes important to determine whether miRNA expression levels are disrupted in PE, and which specific miRNA contributes predominantly to disease pathogenesis.

Meta-analysis in this study revealed significantly higher miRNA-16 expression in the placentas of women with PE compared to those without PE. Confirmation of the possible association between altered expression of miRNA-16 and PE was first described by Hu et al. who showed that there is increased expression of miRNA-16 in the placentas of women with severe PE (Hu et al., 2009). This was followed by Vu et al. who found increased expression of miRNA-16 in the sera of women with PE compared with healthy controls (Wu et al., 2012). The pathologic significance of miRNA-16 lies in its function in regulating the cell cycle. Liu et al. have shown that miRNA-16 stops the cell cycle in G1 phase by regulating the expressions of the CCND3, CCNE1 and CDK6 genes. Based on these physiological roles, it is supposed that miRNA-16 acts as a tumor suppressor (Yan X. et al., 2013). It also is known that the target of miRNA-16 is the Vascular Endothelial Growth Factor (VEGF) gene, whose product is an extremely important protein that initiates vasculogenesis in the placenta and induces proliferation and migration of endothelial cells in blood vessels (Wang and Zhao, 2010). In a study by Wang et al., miRNA-16 was found to have the potential to inhibit proliferation, migration and angiogenesis in mesenchymal stem cells (Wang Y. et al., 2012). The significantly lower miRNA-16 expression levels in the maternal peripheral blood of women with PE compared to those without PE led epigenetic analysis in another direction. It is proposed, but not proven, that miRNA-16 plays a significant role in the progression of human cardiac cell injury in ischemic dilated cardiomyopathy through endoplasmic reticulum stress, inflammation, autophagy, and apoptosis (Calderon-Dominguez et al., 2021). Down regulation of this miRNA, known as an anti-apoptotic factor, also was registered in ischemic myocardial cells, as a reaction to hypoxia in order to protect the tissue (Zhang H. J. et al., 2019). Therefore, miRNA-16 may play a role in both ischemic cardiomyopathy and preeclampsia, which similarly represent hypoxia induced pathological states. Original research articles have reported differing results regarding miRNA-16 levels in pregnancy complications. miRNA-16 levels were elevated in fetal macrosomia, but decreased in severe preeclampsia (Wu et al., 2012; Ge et al., 2015).

Increased expressions of miRNA-20b and miRNA-29b in the placentas of women with PE compared to women without PE were also found in our study. It is well known that the target gene for both miRNA-20 and miRNA-16 is VEGF, thus affecting placental vasculogenesis (Hayder et al., 2018). miRNA-20b binds to the Ephrin Type-B Receptor 4 (EPHB4) and Ephrin Type-B Receptor 2 (EPHB2), important receptors for intercellular communication, which have functions in the regulation of cellular morphology, binding, migration, proliferation, differentiation, and survival. These processes are assumed to be involved in the miRNA-20b contribution to placental blood vessel remodeling (Pasquale, 2005; Lisabeth et al., 2013). miRNA-29b is involved in the processes of trophoblast proliferation and invasion (Harapan and Andalas, 2015). miRNA-29b contributes to preeclampsia through dysregulation of the extracellular signal-regulated protein kinase and focal adhesion kinase (ERK/FAK) signaling pathway that allows the expression of matrix metalloproteinase-2 (MMP2), which is in turn an important factor for migration and invasion of trophoblast cells. Increased expression of miRNA-29b in severe PE has been previously shown to be associated with reduced expressions of MMP2 and integrin β1(ITGβ1) (Li H. et al., 2013).

The increased miRNA-23a levels in PE placentas support previously reported results that the level of this miRNA is upregulated in conditions related to abnormal angiogenesis (Chhabra et al., 2010). The main role of miRNA-23a, as part of the miR-23a∼27a∼24–2 cluster, is to mediate blood vessel genesis. It is included, except in PE, in pathological states such as muscle atrophy, cardiac hypertrophy, and cancers (Chhabra et al., 2010). Data *in vitro*, as well as *in vivo*, indicate that miRNA-23a and miR-23b may have opposite roles, with the former regulating angiogenesis and cellular junctions, and hence inhibiting vascular permeability, while miRNA-23b promotes permeability (Li et al., 2016).

MiRNA-155 expression levels were significantly increased in the placentas and maternal peripheral blood of women with PE compared to those without PE. The increased expression of miRNA-155 and resultant lower levels of cysteine-rich protein 61 (CYR61) and cyclin D1, have been associated with the inhibition of trophoblast invasion (Zhang et al., 2010; Dai et al., 2012). It also has been previously demonstrated that a significant increase in miRNA-155 decreases endothelial nitric oxide synthase (eNOS) expression and thus contributes to development of severe PE (Li X. et al., 2014). This result is consistent with findings from previous studies (Zhang et al., 2010; Gan et al., 2017). This immunomodulatory miRNA, induced in activated T lymphocytes, B lymphocytes and macrophages (Bernstein et al., 2003), is also disrupted in maternal peripheral blood. Its increased expression level was associated with a decreased level of pro-angiogenic factor, VEGF, in an experimental rat model of PE (Cheng et al., 2011). Newly performed studies have reported significantly higher levels of miRNA-155 in the maternal peripheral blood of women with compared to women without PE (Ayoub et al., 2019; Youssef and Marei, 2019; Witvrouwen et al., 2021).

MiRNA-210 has been the most evaluated small non-coding RNA. It is known that miRNA-210 is induced under hypoxic conditions which exist prior to, as well as during the clinical manifestations of PE. Hypoxia stimulates the production of NF-kB 1 (nuclear factor kappa-B 1) and HIF-1A (hypoxia inducible factor 1 α), which induce the expression of miRNA-210 (Muralimanoharan et al., 2012). Previous research has confirmed significantly increased expression of miRNA-210 in both the placentas and sera of women with PE and suggests that miRNA-210 obtained from serum may be a useful biomarker even months before diagnosis (Anton et al., 2013). Micro RNA-210 plays a role in several processes, such as inhibition of cytotrophoblast migration and invasion, differentiation, apoptosis, inflammation, angiogenesis, as well as in the regulation of cellular metabolism. miRNA-210 partially inhibits trophoblast invasion via the ERK/MAPK signaling pathway (Anton et al., 2012). Cell metabolism is dictated by miRNA-210 in that increased expression leads to decreased mitochondrial respiration and vice versa (Hayder et al., 2018). miRNA-210 also plays a role as a suppressor of EFNA3, a member of the ephrin ligand family which is important for cell migration, and HOXA9, an important angiogenesis regulator (Zhang et al., 2012; Luo et al., 2014). Overall, inadequate trophoblast invasion and impaired cellular metabolism are confirmed factors that can lead to the development of PE. Anton et al. found that for each 5-U increase in miR-210 in sera of previously healthy women at the beginning of the second trimester, the odds of PE development later in pregnancy increased fourfold (Anton et al., 2013).

MiRNA-376c plays a role in trophoblast proliferation and differentiation (Hayder et al., 2018). We found significantly lower levels of expression in the placentas of women with PE compared to women without PE, which is consistent with the findings of other studies (Fu et al., 2013; Yang H.-l. et al., 2019). Only Yang et al. showed no significant difference in the levels of miRNA-376c expression in the placentas of women with preterm preeclampsia and gestational age matched controls without PE (Yang H.-l. et al., 2019). Fu at al. showed that a decrease in miR-376c expression results in excessive apoptosis, insufficient cell proliferation, and shallow invasion of trophoblasts in the uterus in preeclampsia (Fu et al., 2013).

In summary, our results clearly identify a subset of miRNAs that are dysregulated in preeclampsia and clearly point towards the underlying mechanisms that may be contributing to the pathophysiology of preeclampsia. Our results set the stage for several venues for future research with an overall goal to facilitate early diagnosis and optimize fetal and maternal outcomes. First, given the clinical heterogeneity of preeclampsia (severe vs. mild, late vs. early, and “placental” vs. “maternal”), adequately designed and powered studies may detect differences in miRNA and related specific underlying mechanisms responsible for specific clinical subtypes. Second, clinical studies may identify a marker (or set of markers) with either predictive or diagnostic role. Third, further discovery of signaling pathways affected by miRNA may lead to mechanism-based therapies.

Our study has several limitations. They originate from the unavailability of all/some data from the original publications, uninformative figures presented in the articles, and selection of the housekeeping gene used for internal controls. The consequences of the data unavailability are possible exclusion of relevant data and a smaller number of included studies, as well as miRNAs, in the meta-analysis that may lead to an overestimation/underestimation of the effects of miRNA expression level on PE development. The importance of adequate selection of the housekeeping gene should be emphasized to standardize miRNA evaluation methodology and to provide comparability between studies. The definition of PE is not the same in each of the included studies which may lead to inclusion of heterogeneous cases that can change the assessment of the effect. Through the systematic review, it was realized that cases and controls were rarely matched for gestational age at the time of sampling. It is necessary to highlight the importance of comparing matched groups because it is known that there are physiological changes in miRNAs expression levels throughout pregnancy. The miRNA source in plasma may be maternal, fetal, or both, yet only a small number of studies reported these data.

## Conclusion

MiRNAs play an important role in the pathophysiology of PE. The functional roles of the microRNAs found to be disrupted in preeclamptic pregnancies include control of trophoblast proliferation, migration, invasion, apoptosis, differentiation, cellular metabolism, and angiogenesis. The identification of differentially expressed miRNAs in maternal blood creates an opportunity to define an easily accessible biomarker of PE. A better understanding of the role of microRNAs in the development of PE offers great potential for developing diagnostic and therapeutic targets for PE.

## Data Availability

The original contributions presented in the study are included in the article/Supplementary Material, further inquiries can be directed to the corresponding authors.
