# Supplementary material for: Preeclamptic Women Have Disrupted Placental microRNA Expression at the Time of Preeclampsia Diagnosis: Meta-Analysis
Source: Front Bioeng Biotechnol. 2021 Dec 24;9:782845. doi: 10.3389/fbioe.2021.782845 (PMC8740308; doi:10.3389/fbioe.2021.782845)
Supplement: Supplementary file 1 [file DataSheet1.docx]

Supplementary Material

# Supplementary Tables

Table S1. Additional inclusion/exclusion criteria

| Author Year  Country | CVD | Diabetes | Renal Disease | Obesity | Previous PE | Other Exclusion Criteria | Other Inclusion Criteria |
| --- | --- | --- | --- | --- | --- | --- | --- |
| Pineles 2007  USA | not reported | not reported | not reported | not reported | not reported | premature rupture of membranes, multiple gestation, histologic chorioamnionitis, stillbirth, or fetal anomalies | not reported |
| Zhu 2009  China | not reported | not reported | not reported | not reported | not reported | not reported | delivered by elective cesarean delivery in the absence of labor |
| Zhang 2010  China | not reported | not reported | yes | not reported | not reported | collagen vascular disease, premature rupture of membrane, and other pregnancy complications such as fetal anomalies or chromosomal abnormalities | cesarean section, normal platelet counts, normal liver functions and normal fetal weight |
| Enquobahrie 2011  USA | not reported | yes | not reported | not reported | not reported | nonsingleton pregnancies | not reported |
| Gunel 2011  Turkey | not reported | not reported | not reported | not reported | not reported | an invasive procedure. | Pregnant women who carried fetuses from 26 to 40 weeks of gestation and none undergone an invasive procedure |
| Mayor-Lynn 2011  USA | not reported | yes | not reported | not reported | not reported | not reported | preterm premature ruptured membranes without labor, multiple gestations, and fetal demise in utero or fetal anomalies |
| Yang 2011  China | not reported | not reported | not reported | not reported | not reported | any other maternal complications, birthweight of <10% | delivered by elective cesarean delivery in the absence of labor |
| Bai 2012  China | not reported | not reported | not reported | not reported | not reported | during pregnancy developed renal disease, transient hypertension in pregnancy, gestational diabetes, spontaneous abortion, intrauterine fetal death, fetal chromosomal or congenital abnormalities or pregnancies conceived by fertility treatment | not reported |
| Hromadnikova 2012  Czech Republic | not reported | not reported | not reported | not reported | not reported | not reported | white women |
| Lazar 2012  Hungary | not reported | not reported | not reported | not reported | not reported | not reported | singleton pregnancies |
| Muralimanoharan 2012  USA | not reported | not reported | not reported | not reported | not reported | not reported | delivery at term by cesarean section (with no labor) |
| Wang 2012  USA | not reported | not reported | not reported | not reported | not reported | signs of infection | not reported |
| Wang 2012  China | yes | yes | yes | not reported | not reported | multiple pregnancies such as twins, fetal structural or genetic anomalies, and the presence of hemolysis, elevated liver enzymes, the HELLP syndrome, hepatic disease, or other infectious disease were criteria for exclusion | not reported |
| Wu 2012  China | not reported | not reported | not reported | not reported | not reported | not reported | between 37-40 gw, Han ethnicity |
| Zhang 2012  China | not reported | not reported | not reported | not reported | not reported | not reported | Chinese women |
| Anton 2013^*^  USA | not reported | not reported | not reported | not reported | not reported | not reported | Pregnant women, with singleton gestations, presenting for fetal aneuploidy screening |
| Betoni 2013  USA | not reported | not reported | not reported | not reported | not reported | patients under 18 years of age, multifetal pregnancy, inability or refusal to provide written informed consent, gestational hypertension, eclampsia, and HELLP syndrome | not reported |
| Fu, 2013  China | not reported | not reported | not reported | not reported | not reported | women who developed renal disease, transient hypertension in pregnancy, gestational diabetes, spontaneous abortion, intrauterine fetal death, fetal chromosomal or congenital abnormalities or pregnancies conceived by fertility treatment | unifetal gestation |
|  | not reported | not reported | not reported | not reported | not reported | women who developed renal disease, transient hypertension in pregnancy, gestational diabetes, spontaneous abortion, intrauterine fetal death, fetal chromosomal or congenital abnormalities or pregnancies conceived by fertility treatment | unifetal gestation |
| Guo 2013  USA | not reported | not reported | not reported | not reported | not reported | multiple gestations, fetuses with documented congenital anomalies, fetuses with documented chromosomal abnormalities, and fetuses with prolonged premature rupture of the membranes (greater than 4 weeks) from ref 6 | 18 and older from ref 6 |
| Hromadnikova 2013  Czech Republic | not reported | not reported | not reported | not reported | not reported | not reported | Caucasian women |
| Yan 2013  China | yes | yes | not reported | not reported | not reported | rupture of membranes, other significant preexisting metabolic disorders, prenatal maternal infection, fetal anomaly, and multifetal gestation | in the 3^rd^ trimester of pregnancy, all underwent cesarean section |
| Campos 2014  Brasil | not reported | not reported | not reported | yes | not reported | none of the women were in labour, nor with superimposed preeclampsia, twin or multiple pregnancies or any evidence of previous medical illness | Caucasian women |
| Doridot 2014  France | not reported | not reported | not reported | not reported | not reported | not reported | after Cesarean section |
| Hong 2014  China | not reported | not reported | yes | not reported | not reported | collagen vascular disease and other pregnancy complications (such as fetal anomalies or chromosomal bnormalities) | after Cesarean section |
| Li 2014  China | not reported | not reported | not reported | not reported | not reported | had renal disease, spontaneous abortion, gestational diabetes, fetal chromosomal or congenital abnormalities | not reported |
| Luo 2014  China | not reported | not reported | not reported | not reported | not reported | developed renal disease, transient hypertension during pregnancy, multiple pregnancy, gestational diabetes, spontaneous abortion, intrauterine fetal death, fetal chromosomal or congenital abnormalities or pregnancies conceived by fertility treatment | not reported |
| Luque 2014  Spain | not reported | not reported | not reported | not reported | not reported | pregnancies with aneuploidies, major fetal abnormalities, and those ending in termination, miscarriage or fetal death before 24 weeks of gestation | singleton pregnancies |
| Weedon-Fekjaer 2014  Norway | not reported | not reported | yes | not reported | not reported | not reported | singleton pregnancy delivered by elective caesarean section, fasting overnight (minimum 6 h), none were in labor, none had ruptured membranes or clinical signs of infection. |
| Xu 2014  China | not reported | not reported | not reported | not reported | not reported | not reported | All the patients accepted no special medical treatment before termination of pregnancy |
| Zhao 2014  China | Yes | Yes | Yes | not reported | not reported | Any complications of pregnancy such as multiple pregnancies including twins, fetal structural or genetic anomalies, hemolysis, elevated liver enzyme levels, the HELLP syndrome, hepatic disease, diabetes, or other infectious | after Cesarean section |
| Zou 2014  China | not reported | not reported | not reported | not reported | not reported | not reported | primiparas aged 20-36years who underwent Cesarean deliveries |
| Akehurst 2015  Scotland | not reported | not reported | not reported | no | yes | not reported | women with two or more risk factors for preeclampsia (nulliparity, age over 35 years, BMI >30 kg/m2, family history of preeclampsia in mother or sister at 16-28 gw |
| Chen 2015  China | not reported | not reported | not reported | not reported | not reported | not reported | Cesarean section |
| Ding 2015  China | not reported | not reported | yes | not reported | not reported | Gestational diabetes mellitus, placental abruption or placenta praevia, anemia or other hematologic disease, fetal distress syndrome or growth retardation | not reported |
| Hromadnikova 2015a  Czech Republic | not reported | not reported | not reported | not reported | not reported | Patients with a complicated gestation demonstrating premature rupture of membranes, in utero infections, fetal anomalies or chromosomal abnormalities, and fetal demise in utero or stillbirth | Cucasian |
| Hromadnikova 2015b  Czech Republic | not reported | not reported | not reported | not reported | not reported | premature rupture of membranes, in utero infections, fetal anomalies or chromosomal abnormalities, and fetal demise in utero or stillbirth | Caucasian |
| Jiang 2015  China | not reported | not reported | yes | not reported | not reported | Gestational diabetes mellitus, spontaneous abortion or fetal chromosomal or congenital abnormalities | not reported |
| Zhang 2015  China | not reported | not reported | not reported | not reported | not reported | women with any preexisting diseases or other pregnancy complications were excluded from this study | not reported |
| Hromadnikova 2016  Czech Republic | not reported | not reported | not reported | not reported | not reported | complicated gestation demonstrating premature rupture of membranes, in utero infections, fetal anomalies or chromosomal abnormalities, and fetal demise in utero or stillbirth | Caucasian pregnant women |
| Hu 2016  China | not reported | not reported | not reported | not reported | not reported | not reported | women who underwent elective cesarean section |
| Munaut 2016  Belgium | not reported | not reported | not reported | not reported | not reported | manifestation of PE at selection | pregnant women at 24 to <37 weeks’ gestation, clinical suspicion of, but not manifesting preeclampsia/eclampsia/HELLP syndrome |
| Ospina-Prieto 2016  Germany |  |  |  |  |  | multiple pregnnacies | not reported |
| Sandrim 2016a  Brasil | not reported | not reported | not reported | not reported | not reported | not reported | not reported |
| Sandrim 2016b  Brasil | yes | yes | yes | not reported | not reported | superimposed pre-eclampsia, haemostatic abnormalities, nonwhite, cancer, twin pregnancy, autoimmune and hepatic diseases | not reported |
| Vashukova 2016  Russia | yes | yes | yes | not reported | not reported | hepatitis, intrapartum infection or other pregnancy complications | not reported |
| Wang 2016  USA | not reported | not reported | not reported | not reported | not reported | present signs of infection | not reported |
| Zhou 2016  China | not reported | not reported | not reported | not reported | not reported | not reported | Han ethnicity |
| Adel 2017  Egypt | not reported | not reported | not reported | not reported | not reported | not reported | primigravidas |
| Azizi 2017  Iran | not reported | not reported | yes | not reported | not reported | collagen vascular disease, positive family history for PE and other pregnancy complications (such as fetalanomalies or chromosomal abnormalities) | not reported |
| Guo 2017  China | not reported | not reported | yes | not reported | not reported | Complications (not reported) | not reported |
| Han 2017  China | not reported | not reported | not reported | not reported | not reported | blood transfusion, immunotherapy and other obstetric complications | Singleton pregnancies |
| Hromadnikova 2017  Czech Republic | not reported | not reported | not reported | not reported | not reported | premature rupture of membranes, in utero infections, fetal anomalies or chromosomal abnormalities, and fetal demise in utero or stillbirth | Caucasian pregnant women |
| Hu 2017  China | not reported | not reported | not reported | not reported | not reported | not reported | elective Cesarean section |
| Huang 2017  China | yes | yes | yes (nephropathy, kidney disease) | not reported | not reported | not reported | Chinese Han women |
| Jairajpuri 2017  Kingdom of Bahrain | no | no | no | not reported | not reported | not reported | women in 3^rd^ trimester pregnancy |
| Korkes 2017  USA | not reported | yes | yes | not reported | not reported | multiple gestations | not reported |
| Li 2017b  China | not reported | not reported | yes (chronic nephritis) | not reported | not reported | Gestational diabetes mellitus, multiple gestations, the HELLP syndrome, hepatic disease, in vitro fertilization and embryo transfer (IVF-ET) or other infectious and neoplastic disease and fetal congenital defect | not reported |
| Lu 2017  China | yes | not reported | not reported | not reported | not reported | congenital diseases, hereditary diseases, autoimmune diseases, and cardiovascular diseases, pregnancy complications such as, hypertension, nephritis, heart diseases, anemia, hepatitis, intrahepatic cholestasis in gestation period, and sexually transmitted diseases, internal medicine complications and other obstetric complications such as placenta praevia, placental abruption, mother and child blood type incompatibility, gestational diabetes, premature rupture of fetal membranes, and so on, serious smoking, alcoholism, drug abuse, or history of mental illness | not reported |
| Meng 2017  Inner Mongolia (China) | not reported | not reported | not reported | not reported | not reported | other pathologies (exclude preeclampsia) or under medication were excluded | Chinese Han |
| Nizyaeva 2017  Not reported | not reported | yes | not reported | not reported | not reported | severe extragenital pathology, post-transplantation state, history of cancer, severe fetal abnormalities, and congenital malformations of the fetus. | Not reported |
| Salomon 2017  Chile | not reported | not reported | not reported | not reported | not reported | not reported | singleton gestation and none of them took multivitamins or aspirin during pregnancy, without intrauterine infection |
| Shao 2017  China | not reported | not reported | not reported | not reported | not reported | developed renal disease, cardiovascular disease, gestational diabetes mellitus, spontaneous abortion, intrauterine fetal death, fetal chromosomal or congenital abnormalities, or pregnancies that were conceived with the assistance of fertility treatments | not reported |
| Singh 2017  USA | not reported | yes | not reported | not reported | not reported | gestational diabetes, multiple gestation | not reported |
| Wei 2017  New Zeland | not reported | not reported | not reported | not reported | not reported | not reported | singleton pregnancies |
| Yang 2017  China | not reported | not reported | yes | not reported | not reported | spontaneous abortion, gestational diabetes, fetal chromosomal, or congenital abnormalities | not reported |
| Zhang 2017  China | yes | yes | yes | not reported | not reported | not reported | Chinese Han women |
| Brkic 2018  China | not reported | not reported | not reported | not reported | not reported | Patients who developed renal disease, transient hypertension during pregnancy, gestational diabetes, spontaneous abortion, intrauterine fetal death, fetal chromosomal or congenital abnormalities or pregnancies conceived by fertility treatment | pregnant women who underwent perinatal medical care |
|  | not reported | not reported | not reported | not reported | not reported | known HIV or Hepatitis infection and any classified as a missed miscarriage | not reported |
| Chi 2018  China | not reported | not reported | not reported | not reported | not reported | All patients with complications of pregnancy, including twins, fetal gene abnormalities, maternal chronic hypertension, aberrant liver enzyme levels, cardiovascular disease, renal disease, diabetes or other infectious diseases | 25-35 years previously underwent caesarean section |
| Dai 2018  China | not reported | not reported | not reported | not reported | not reported | pregnancy complicated by essential hypertension, diabetes and chronic nephrosis | not reported |
| Fang 2018  China | not reported | not reported | not reported | not reported | not reported | internal and surgical diseases, drug taken history during pregnancy, or in vitro fertilization. | Not reported |
| Khaliq 2018  South Africa | not reported | not reported | not reported | not reported | not reported | not reported | aged between 17–45 years |
| Kim 2018  Republic of Korea | not reported | not reported | not reported | not reported | not reported | not reported | not reported |
| Li 2018  China | not reported | yes | yes (chronic nephritis) | not reported | not reported | twin pregnancy or above; a history of blood transfusion or application of special drugs during pregnancy; severe infection during pregnancy; limb defects or suffering major accidents before pregnancy; not cooperative in treatment or follow‑up | no familial genetic disease and immune deficiency; patients without congenital heart disease, hepatitis; patients with healthy diet and regular rest; patients without diseases of respiratory or digestive system. |
| Liu 2018  China | yes | yes | yes (chronic nephritis) | not reported | not reported | not reported | Monocyesis, normal pregnancy at the time of sample collection and no pre‑existing clinical conditions, including hepatic or autoimmune disease. |
| Lykoudi 2018  Greece | not reported | not reported | not reported | not reported | not reported | multiple pregnancies, congenital or chromosomal abnormalities of the fetus, renal disease, chronic hypertension, cardiovascular disease or other pregnancy complications | not reported |
| Martinez-Fierro 2018  Mexico | not reported | yes (DM2) | yes (chronic renal disease) | not reported | no | gestational hypertension, gestational diabetes mellitus, hypo/hyperthyroidism, rheumatoid arthritis, and systemic lupus erythematosus | not reported |
| Motawi 2018  Egypt | not reported | not reported | yes | not reported | not reported | fetuses affected with congenital anomalies | not reported |
| Niu 2018  China | yes (cardiac diasese) | yes | yes | not reported | not reported | no maternal complications such as multiple pregnancies, fetal anomalies, premature rupture of membranes, maternal infection | Cesarean delivery singleton pregnancies |
| Shen 2018  China | yes | yes | yes (chronic nephritis) | not reported | not reported | not reported | not reported |
| Timofeeva 2018  Russia | not reported | not reported | not reported | not reported | not reported | assisted reproductive techniques to increase fertility, multifetal pregnancies, severe somatic pathology in pregnant women, fetal aneuploidy, and vaginal delivery | not reported |
| Wang 2018  China | not reported | not reported | not reported | not reported | not reported | not reported | Cesarean delivery |
| Wang 2018  China | yes | not reported | yes | not reported | not reported | transient hypertension in pregnancy, intrauterine fetal death, gestational diabetes, fetal congenital or chromosomal abnormalities or pregnancies conceived by in-vitro fertilization | not reported |
| Wang 2018  China | not reported | not reported | not reported | not reported | not reported | not reported | Cesarean delivery |
| Winger 2018  USA | not reported | not reported | not reported | yes | not reported | not reported | singleton delivery, delivered baby with no obvious birth defects, BMI <30, blood sample available from 11±13 weeks gestation |
| Awamleh 2019  Canada | not reported | yes | not reported | not reported | n | gestational diabetes, obvious chorioamnionitis, alcohol/drug use, chromosomal or genetic abnormalities, congenital anomalies, or infection | not reported |
| Biro 2019  Hungary | not reported | not reported | not reported | not reported | not reported | Pregnancies with ongoing labor, multiple gestations, and congenital malformations | not reported |
| Chen 2019  China | not reported | yes | yes | not reported | not reported | multifetal gestation, autoimmune or vascular disease, infection (HIV, syphilis or hepatitis B or C), present signs of infection, active labor, rupture of the amniotic membrane, hormonal contraception | not reported |
| Devor 2019  USA | not reported | not reported | not reported | not reported | not reported | HIV positive, hepatitis C positive, incarcerated, or who had fetuses with a major structural or chromosomal abnormality | women with singleton pregnancies older than 18 years, less than 40-year old presenting for prenatal care prior to 12 weeks |
| Dong 2019  China | not reported | not reported | not reported | not reported | not reported | pregnancy-induced diabetes or any other medical problem that would predispose them to an alteration in their immune response such as nephritis, cancer, systemic lupus erythematosus (SLE) | singleton and not in labor |
| Eghbal-Fard 2019  Iran | not reported | not reported | not reported | not reported | not reported | history hyperthyroidism and hypothyroidism, hypertension, autoimmune diseases (such as rheumatoid arthritis and lupus), intrahepatic cholestasis of pregnancy, active thromboembolic disorders, maternal or fetal infection as well as bleeding, miscarriage, and abruption. Receiving steroids or other immunosuppressive medicines | not in active labor at the time of enrollment and blood sampling |
| Hocaoglu 2019  Turkey | yes | yes | yes | not reported | not reported | under 18 years of age, twin or multiple pregnancies, hemostatic abnormalities, any other confounding pathologies (including intrahepatic cholestasis of pregnancy, autoimmune, hepatic diseases), cancer, fetal congenital anomalies, intrauterine fetal death. | Not reported |
| Hromadnikova 2019a  Czech Republic | not reported | not reported | not reported | not reported | not reported | not reported | Caucasian women with equal age |
| Hromadnikova 2019b  Czech Republic | not reported | not reported | not reported | not reported | not reported | not reported | singleton pregnancies of Caucasian descent only |
| Hu 2019  China | not reported | not reported | not reported | not reported | not reported | not reported | not reported |
| Huang 2019  China | not reported | not reported | not reported | not reported | not reported | not reported | primiparas after Cesarean section |
| Li 2019  China | not reported | not reported | not reported | not reported | not reported | not reported | not reported |
| Liu 2019  China | not reported | not reported | not reported | not reported | not reported | not reported | not reported |
| Liu 2019  China | not reported | not reported | not reported | not reported | not reported | not reported | not reported |
| Liu 2019  China | yes (heart disease) | yes | not reported | yes (kidney disease) | not reported | thyroid disease, and other complications that may lead to hypoxic changes | termination of pregnancy via cesarean section with epidural anesthesia |
| Ma 2019  China | not reported | yes | not reported | not reported | not reported | malnutrition, prenatal infections, or fetal chromosomal abnormalities, one or more malignant tumors, other complications developed during the pregnancy period | aged above 18 years with a gestational period longer than 20 weeks, with no congenital immunodeficiencies, physical disabilities, or cognitive dysfunction, willing to cooperate with treatment protocols and follow-up |
| Martinez-Fierro 2019  Mexico | not reported | not reported | not reported | not reported | no | not reported | not reported |
| Mei 2019  China | not reported | not reported | not reported | not reported | not reported | not reported | not reported |
| Nejad 2019  Iran | not reported | yes | not reported | not reported | yes | twin pregnancy and history of any other disease | not reported |
| Pillay 2019  South Africa | not reported | not reported | not reported | not reported | n | not reported | singleton pregnancies with no evidence of any infections or medical, surgical or other obstetric complications. |
| Sekar 2019  India | not reported | not reported | not reported | not reported | not reported | not reported | not reported |
| Shi 2019  China | not reported | not reported | not reported | not reported | not reported | not reported | not reported |
| Tang 2019  China | not reported | yes | yes | not reported | not reported | multifetal gestation, autoimmune disease, angiopathy, maternal or fetal infection, and fetal congenital anomaly | not reported |
| Wang 2019  Taiwan | not reported | not reported | not reported | not reported | not reported | not reported | not reported |
| Wang 2019  China | not reported | not reported | not reported | not reported | not reported | not reported | not reported |
| Wang 2019  China | not reported | not reported | not reported | not reported | not reported | not reported | not reported |
| Wang 2019  China | yes | yes | not reported | not reported | not reported | multiple pregnancies, rupture of membranes, and fetal structural or genetic anomalies, severe pre‐existing metabolic disorders | nulliparous women in the 3^rd^ trimester after Cesarean section |
| Wang 2019  China | yes | not reported | yes (kidney function damage) | not reported | not reported | multiple pregnancy, server liver function damage, psychiatric disorder and other pregnancy coexisted diseases | not reported |
| Xiaobo 2019  China | not reported | not reported | yes | not reported | not reported | twin pregnancy and acute or chronic hepatitis | not reported |
| Xie 2019  China | yes | not reported | not reported | not reported | not reported | Hypertensive emergencies, abnormal pregnancy with other complications of obstetrics | not reported |
| Xue 2019  China | not reported | not reported | not reported | not reported | not reported | not reported | not reported |
| Yang 2019  China | not reported | not reported | not reported | not reported | not reported | gestational diabetes, transient hypertension, spontaneous abortion, congenital abnormalities in the fetus, and intrauterine fetal death | unifetal gestation |
| Yang 2019a  China | not reported | not reported | yes (kidney disease) | not reported | not reported | history of drug or alcohol abuse half year prior to providing written informed consent | Cesarean section |
| Yang 2019b  China | yes (heart diseases) | yes | yes (nephropathy) | not reported | not reported | not reported | Cesarean section, Han ethnicity |
| Yang 2019a  China | not reported | not reported | not reported | not reported | not reported | not reported | Cesarean section |
| Yang 2019b  China | yes (heart diseases) | yes | yes (chronic nephritis) | not reported | not reported | autoimmune diseases, thrombophilic conditions, vaginal delivery, fetal malformation, and HELLP syndrome (hemolysis, elevated liver enzymes and low platelet count) | Cesarean section |
| Youssef 2019  Egypt | not reported | not reported | not reported | not reported | not reported | pregnancy complications or systemic diseases | not reported |
| Zhong 2019  China | not reported | not reported | not reported | not reported | not reported | not reported | not reported |
| Ayoub 2019  Egypt | not reported | yes | yes | not reported | not reported | chromosomal abnormalities, alcoholism, and fetal congenital abnormalities | All subjects were unrelated and of the same race |
| Demirer 2019  Turkey | yes | yes | yes | yes (body mass index ≥40 kg/m2) | not reported | >18 years of age, gestational hypertension, with or without  superimposed PE, twin or multiple pregnancies, hemostatic abnormalities, and any other confounding pathologies (including intrahepatic  cholestasis of pregnancy, autoimmune, renal and hepatic diseases), cancer, pregnancies complicated by neonatal chromosomal, structural anomalies, intrauterine infection, intrauterine fetal death. | Not reported |
| Dong 2019  China | not reported | yes (pregnancy induced) | yes (nephritis) | not reported | not reported | Cancer, Systemic lupus erythematosus | All pregnancies were singleton and not in labour |
| Lip, 2019  Netherlands | not reported | no | not reported | not reported | not reported | no autoimmune diseases (antiphospholipid syndrome, SLE) | not reported |
| Lv, 2019  China | yes | not reported | yes | not reported | not reported | Poor lifestyle habits, liver disease | not reported |
| Xu, 2019  USA | not reported | yes | yes | not reported | not reported | Infection, HELLP syndrome | not reported |
| Yang, 2019  China | yes | yes | yes | not reported | not reported | fetal dysplasia, premature rupture of membranes, or infection of maternity | Han ethnicity |
| Yuan, 2019  China | not reported | not reported | not reported | not reported | not reported | no other medical and surgical complications before and after pregnancy except PE | monocyesis |
| Akgor, 2020  Turkey | not reported | not reported | not reported | not reported | not reported | Multiple pregnancies, pregnancy-related diseases and any pregnancy  complications except PE, and history of additional comorbidities | gestational age from 20 to 40 weeks, singleton pregnancies, age more than 18 years old |
| Devor, 2020  USA | not reported | not reported | not reported | not reported | not reported | HIV positive, hepatitis C positive, incarcerated, or who had fetuses with a major structural or chromosomal  abnormality | singleton pregnancies older than 18 years, less than 40-year old presenting for prenatal care prior to 12 weeks |
| Dong, 2020  China | not reported | not reported | not reported | not reported | not reported | chromosomal abnormalities, anatomical variations, hormonal disorders and infectious diseases | single pregnancy, age <35 years and >18 years, no previous or ongoing treatment |
| Fan, 2020  China | yes | yes | yes | not reported | not reported | thyroid disease, or multiple pregnancies | not reported |
| Han, 2020  China | not reported | not reported | not reported | not reported | not reported | blood transfusion and immunotherapy, and other obstetric complications were excluded | singleton |
| Huang, 2020  China | not reported | yes | yes (chronic kindey disease) | not reported | not reported | Essential hypertension | normal liver and kidney function and platelet count. |
| Jelena, 2020  Serbia | not reported | yes | not reported | not reported | not reported | Multifetal pregnancy, major fetal anomaly, fetal death, any condition requiring termination of pregnancy, any maternal chronic disease and inability to give informed consent | not reported |
| Kim, 2020  South Korea | not reported | yes | not reported | not reported | not reported | elevated liver enzyme levels, twin or multiple pregnancies, or any evidence of previous medical illness, superimposed PE | not reported |
| Li T, 2020  China | not reported | not reported | not reported | not reported | not reported | malignant tumors, chemotherapy, immunotherapy and radiothe­rapy before surgery | Conscious, 29-41 weeks of gestation with normal liver and kidney function |
| Li H, 2020  China | not reported | yes | not reported | not reported | not reported | Multiple pregnancies, history of labor induction at >16 weeks of gestational age, placenta previa, placental abruption, idiopathic hrombocytopenia,  antiphospholipid antibody syndrome, and other severe complications during pregnancy |  |
| Licini, 2020  Russia | yes | yes | yes | not reported | not reported | multiparity, multiple gestation,  thyroid and immune diseases and congenital or acquired thrombophilia | 18 to 45 years with a singleton pregnancy and no genetic diseases (e.g. aneuploidy) who gave their written informed consent  to participate |
| Sheng, 2020  China | yes | yes | yes | not reported | not reported | maternal complications such as multiple pregnancies, fetal  anomalies, premature rupture of membranes, maternal infection, | Singleton pregnancies |
| Tao, 2020 | not reported | not reported | not reported | not reported | not reported | not reported | Cesarean delivery |
| Whigham, 2020 | not reported | not reported | not reported | not reported | not reported | not reported | English-speaking women aged over 18 years, carrying a well-dated singleton pregnancy with normal midtrimester morphology ultrasound |
| Wu, 2020 | not reported | not reported | no | not reported | not reported | patients with gynecological organic lesions such as uterine fibroids or ovarian cysts, etc., as detected by B-ultrasound and a gynecological examination; an adverse birth history (e.g., abortion, fetal growth restriction or stillbirth). | Cesarean section, pregnant woman as confirmed by B-ultrasound and an obstetric examination; *>* 20 weeks of pregnancy |
| Zheng W, 2020 | yes | not reported | yes | not reported | not reported | not reported | Cesarean section, singleton pregnancies and no other complications, including premature membrane rupture, and maternal infection |
| Zhou 2020 | not reported | not reported | not reported | not reported | not reported | Age over 45 years old, history of immune diseases before pregnancy, history of ovarian or uterine diseases, hormonal disorder and long‑term use of hormones or immunosuppressive drugs | Good health before pregnancy and no history of induced abortion |
| Zhu, 2020 | not reported | not reported | not reported | not reported | not reported | not reported | Cesarean delivery |
| Ali, 2021 | yes | yes | yes | not reported | yes | Multiple gestations, chronic diseases like chronic arthritis, inflammatory disease of the bowl, high blood pressure, any persistent inflammatory disease |  |
| Cai, 2021 | yes | yes | yes | not reported | not reported | hepatitis, infectious diseases during pregnancy, or other complications during pregnancy | All subjects were mononucleosis-free and had no other medical and surgical complications before and after pregnancy (except PE) |
| Jiu, 2021 | not reported | not reported | not reported | not reported | not reported | not reported | Cesarean section |
| Kolkova, 2021 | not reported | not reported | not reported | not reported | not reported | not reported | Caucasian women with singleton pregnancies |
| Liao, 2021 | yes | yes | yes | not reported | not reported | not reported | Singleton pregnancies without other maternal complications, such as multiple pregnancy, fetal abnormalities, premature rupture of membranes, maternal infection |
| Martinez-Fierro, 2021 | not reported | not reported | not reported | not reported | not reported | other pregnancy-related disorders and/or underlying medical diseases | not reported |
| Peng, 2021 | yes | yes | yes (nephropathy) | yes | not reported | hepatopathy, intra uterine fetal death, fetal chromosomal or congenital abnormalities, and pregnancy following fertility treatment | not reported |
| Witvrouwen, 2021 | not reported | yes ((gestational) diabetes) | yes (chronic kindey disease) | not reported | not reported | multiple pregnancies, fetal malformations, hypercholesterolemia, autoimmune disorders, connective tissue diseases or use of acetylsalicylic acid | not reported |
| Xu, 2021 | not reported | not reported | not reported | not reported | not reported | no other medical and surgical complications before and after pregnancy | Singleton pregnancies |
| Yu, 2021 | not reported | not reported | yes (chronic nephritis) | not reported | not reported | alcohol drinking, smoking, malignancy, severe infection, autoimmune and renal diseases | not reported |
| Zhao, 2021 | yes | yes | yes | not reported | not reported | medical and surgical complications, such as hemolysis, hepatic disease or other infectious disease | not reported |
| Zhao, 2021 | not reported | yes | yes | not reported | not reported | Infection and systemic lupus erythematosus, and no other pregnancy complications | not reported |
| Zhu, 2021 | yes | yes | yes | not reported | not reported | not reported | not reported |

Table S2. Preeclampsia definitions and/or guidelines

| **Definition** | **Guideline/Author** | **Ref No** |
| --- | --- | --- |
| Preeclampsia was diagnosed when both pregnancy-induced hypertension and proteinuria were present. Pregnancy-induced hypertension was defined as a sustained (≥2 measures 6 hours apart) blood pressure elevation (>140/90 mm Hg) >20 weeks of gestation. Proteinuria was defined as a sustained ( ≥2 measures 4 hours apart) presence of elevated protein in the urine ( >30 mg/dL or >1+ on a urine dipstick) | **ACOG 2000** | (Enquobahrie et al., 2011) |
| Preeclampsia was defined as hypertension—systolic blood pressure, ≥140 mm Hg, or diastolic blood pressure, ≥90 mmHg, on at least 2 occasions, at least 4 hours apart, and proteinuria of ≥300 mg in a 24-hour urine collection or urine dipstick measurement of ≥2+ | not reported | (Mayor-Lynn et al., 2011) |
| Preeclampsia was defined as blood pressure >140/90mm Hg in two determinations 4 hours apart or by a diastolic blood pressure >110 mm Hg that was associated with a protein level >300 mg/24 h after 20 weeks of gestation | **ACOG no year** | (Hromadnikova et al., 2012) |
| preeclampsia is defined as elevated blood pressure (≥140/90 mm Hg on two measurements ≥6 hours apart), with ≥1+ proteinuria (0-trace protein for gestational hypertension | **ACOG no year** | (Anton et al., 2013) |
| PE was defined as systolic blood pressure ≥140mmHg or diastolic blood pressure ≥90mmHg on two occasions 6hours apart after 20 weeks of gestation, and proteinuria of 300 mg/24 h on two occasions 6 hours apart | not reported | (Betoni et al., 2013) |
| Preeclampsia was diagnosed when both pregnancy-induced hypertension and proteinuria were present. Pregnancy-induced hypertension was defined as a sustained (2 measures 6 h apart) blood pressure elevation (>140/90 mmHg) >20 weeks of gestation. Proteinuria was defined as a sustained ( 2 measures 4 h apart) presence of elevated protein in the urine (>30 mg/dL or >1+ on a urine dipstick) | **ACOG 2000** | (Guo et al., 2013) |
| Preeclampsia was defined as blood pressure >140/90mmHg in two determinations 4 hours apart that was associated with proteinuria >300mg/24 h after 20 gw. | not reported | (Hromadnikova et al., 2013) |
| Pre‐eclampsia was diagnosed as systolic blood pressure >140mmHg and/or diastolic blood pressure >90mm Hg arising after 20 weeks of gestation and proteinuria of ≥1+ (300 mg/24 h) in a previously normotensive woman | Chesley LC. 1980. Hypertension in pregnancy: Definitions, familial factor, and remote prognosis. Kidney Int 18:234–240. | (Yan et al., 2013) |
| Preeclampsia was defined as increased blood pressure (≥140 mmHg systolic or ≥90 mmHg diastolic on 2 or more measurements at least 6 h apart) with significant proteinuria (≥0.3 g/24 h) in a woman after 20 weeks of gestation. | Anonymous. Report of the National High Blood Pressure Education Program Working Group on high blood pressure in pregnancy. Am J Obstet Gynecol 2000;183:S1–S22. | (Campos et al., 2014) |
| Systolic pressure > 140 mmHg, diastolic pressure> 90 mmHg, and proteinuria > 0.3 g per 24 h | not reported | (Doridot et al., 2014) |
| Pre-eclampsia was defined as gestational hypertension (systolic pressure ≥140mmHg and/or diastolic blood pressure ≥90mmHg on at least two occasions after gestational week 20, with proteinuria ( ≥0.3 g/24 h) | **ACOG 2002** | (Hong et al., 2014)  Yang 2019  Licini 2020  Mavreli 2020  Whigham 2020  Jairajpuri 2021 |
| not reported | **ISSHP** criteria no reference | (Akehurst et al., 2015) |
| not reported | **ACOG no year** | (Anton et al., 2015)  Xueya 2020  Chu 2021 |
| Preeclampsia was defined as blood pressure > 140/90 mmHg in two determinations 4 hours apart that was associated with proteinuria > 300 mg/24 h after 20 weeks of gestation | **ACOG 2002** | (Hromadnikova et al., 2015a)  (Hromadnikova et al., 2015b) |
| Preeclampsia is defined as the onset of gestational hypertension and proteinuria after 20 weeks of gestation. Hypertension was defined as two or more recordings of a diastolic blood pressure of ≥90 mmHg taken ≥4 hours apart. Proteinuria was defined as the excretion of≥300 mg of protein over 24 hours | Lasabova Z, Zigo I, Svecova I, Szabo G, Stanclova A, Skerenova M, et al. (2014). Association of specific diplotypes defined by common rs1800682 and rare rs34995925 single nucleotide polymoprhisms within the STAT1 transcription binding site of theFAS gene promoter with preeclampsia. Gen PhysiolBiophys. 33:199–204. | (Lasabová et al., 2015)  Kolkova 2021 |
| PE was defined as systolic pressure >140 mmHg or diastolic blood pressure >90 mmHg in 2 different occasions with proteinuria (>0.3 g/24 h) after the 20th gestation week | not reported | (LI et al., 2015) |
| PE was defined as systolic blood pressure ≥140 mmHg and diastolic blood pressure ≥90 mmHg with proteinuria >300mg/24 hours or 1+ on repeat dipstick at the time of presentation to clinic or 120 admission/transfer to a participating research hospital | based on PE-NET cohort diagnosticcriteria (no reference) | (Murphy et al., 2015) |
| Preeclampsia was defined as blood pressure >140/90mmHg in two determinations 4 h apart that was associated with proteinuria>300 mg/24 h after 20 weeks of gestation | **ACOG 2002** ACOG practice bulletin, Diagnosis and management of preeclampsia and eclampsia, Obstet. Gynecol. 99 (2002) 159–167. | (Hromadnikova et al., 2016)  Lip 2019  Akgor 2020 |
| Preeclampsia was defined as pregnancy induced hypertension (blood pressure ≥140/90 mmHg) and proteinuria (≥0.3 g/24 h or ≥1+ according to a routine urinalysis) in women who were normotensive before pregnancy and had no other underlying clinical problems such as renal disease | not reported | (Hu et al., 2016) |
| PE was defined as de novo hypertension after 20th week of gestation plus proteinuria | World Health Organization. Reproductive health and research. Managing complications in pregnancy and childbirth: a guide for midwives and doctors. Geneva: World Health Organization, Dept. of Reproductive Health and Research, 2003. | (Ospina-Prieto et al., 2016) |
| Pre-eclampsia was defined as increased blood pressure (≥140 mmHg systolic or ≥90 mmHg diastolic on two or more measurements at least 6 hours apart) with significant proteinuria (≥0.3 g/24 hours) in a woman after 20 weeks of gestation, in accordance with the guidelines of the NHBPEP (National High Blood Pressure Education Program Working Group on High Blood Pressure in Pregnancy). | not reported | (Sandrim et al., 2016b) |
| Diagnosis of preeclampsia was defined as follows: a sustained systolic blood pressure of ≥ 140 mm Hg or a sustained diastolic blood pressure of ≥90 mm Hg on two separate readings; proteinuria measurement of 1+ or more on dipstick; or 24-hour urine protein collection with ≥300 mg in the specimen | not reported | (Wang et al., 2016a) |
| not reported | Schroeder BM. ACOG practice bulletin on diagnosing and managing preeclampsia and eclampsia. American College of Obstetricians and Gynecologists. Am Fam Physician 2002; 66(2): 330–331. | (Wang et al., 2016b) |
| PE was defined as systolic blood pressure (SBP) ≥ 140 mmHg and/or diastolic blood pressure (DBP) ≥ 90 mmHg on 2 occasions at least 4 hours apart with proteinuria ≥ 300 mg/day from 24 h urine collection occurring after the 20th week of gestation but resolving by the 12th week postpartum | not reported | (Zhou et al., 2016) |
| PE was diagnosed as systolic blood pressure ≥160mm Hg or diastolic blood pressure ≥110mm Hg on at least 2 occasions, accompanying severe proteinuria (≥2g/24 hours) at 20 to 34 weeks of gestation | not reported | (Gan et al., 2017) |
| preeclampsia is indicated in a previously normotensive pregnant woman who after 20 weeks of gestation displays a systolic blood pressure ≥140 mmHg and/or a diastolic blood pressure ≥90 mmHg twice more than 4 h apart and presents with 300 mg proteinuria in a 24-h period (or a protein/creatinine rate of at least 0.3 or proteinuria ≥1+) or with ≥1 severe features in the absence of proteinuria | **ACOG 2013** | (Zhang et al., 2017)  Demirer 2019  Sheng 2020  Luizon 2021 |
| The diagnostic criteria for PE followed that of the American College of Obstetricians and Gynecologists, with either severe hypertension (≥160 mmHg and/or 110 mmHg) plus mild proteinuria or mild hypertension plus severe proteinuria (>2 g/24 h or >2+) | **ACOG no year** | Zheng W 2020  Zhao 2021a |
| not reported | **ACOG 2013** | (Korkes et al., 2017) |
| PE was defined as either severe hypertension or severe proteinuria in accordance with the International Society for the Study of Hypertension in Pregnancy | not reported | (Li et al., 2017a) |
| PE was defined as gestational hypertension (systolic pressure > 140 mmHg or diastolic blood pressure > 90 mmHg on two or more occasions after gestational week 20) with proteinuria (> 0.3g/day) | not reported | (Li et al., 2017b)  Ayoub2019 |
| not reported | **ACOG no year** | (Luo et al., 2017b) |
| PE was defined as new-onset hypertension [(BP) blood pressure ≥140/90mmHg on two separate occasions at least 6 h apart or BP ≥160/110mmHg)]and proteinuria (>300mg/24h) after 20 weeks of gestation in previously normotensive women | not reported | (Salomon et al., 2017) |
| PE was defined as new-onset hypertension [(BP) blood pressure ≥140/90mmHg on two separate occasions at least 6h apart or BP ≥160/110mmHg)]and proteinuria (>300mg/24h) after 20 weeks of gestation in previously normotensive women | The International Society for the Study of Hypertension in Pregnancy **ISSHP 2013** Tranquilli AL, Brown MA, Zeeman GG, Dekker G, Sibai BM. The definition of severe and early-onset preeclampsia. Statements from the International Society for the Study of Hypertension in Pregnancy (**ISSHP**). Pregnancy Hypertens. 2013; 3(1):44±7. | (Truong et al., 2017)  Jelena 2020  Brodowski 2021 |
| Preeclampsia was defined as hypertension (systolic blood pressure ≥140 mm Hg or diastolic blood pressure ≥90 mmHg on at least2 occasions taken 4 h apart) and proteinuria ( 300 mg in a 24-hour urine collection or 1 dipstick measurement of +2) occurring after 20 weeks of gestation in a previously normotensive woman | not reported | (Tsai et al., 2017) |
| The criteria used for diagnosis of pre‑eclampsia were as follows: i) Systolic blood pressure of ≥140 mm Hg or diastolic blood pressure of ≥90 mm Hg occurring 20 weeks post‑gestation in a woman whose blood pressure was previously normal; and ii) proteinuria, with excretion of ≥0.3 g in a 24‑h period | not reported | (Wang et al., 2017) |
| The diagnostic criteria for preeclampsia was new-onset of hypertension after 20 weeks of gestation (defined as systolic blood pressure ≥ 140 mmHg or diastolic blood pressure ≥ 90 mmHg on two occasions, at least 4 hours apart), accompanied with one or more of the following symptoms, new-onset proteinuria (>0.3 gram per 24 hours), new-onset thrombocytopenia (platelet count < 100,000/μl), impaired liver function, renal insufficiency, pulmonary edema and visual or cerebral disturbances | **ACOG 2013** | (Wei et al., 2017)  Devor 2020  Fan 2020 |
| Preeclampsia (PE) was diagnosed when patients had no history of preexisting or chronic hypertension, but they showed systolic blood pressure of >140 mm Hg or diastolic blood pressure of >90 mm Hg on at least 2 occasions, accompanied by significant proteinuria (>2 g per 24 h in 2 samples collected at >4 h intervals) after 20 weeks of gestation | not reported | (Brkić et al., 2018) |
| diagnosis of PE, systolic blood pressure >140 mmHg and diastolic blood pressure >90 mmHg | not reported | (Chi and Zhang, 2018) |
| PE was defined as a sustained blood pressure of ≥ 140/90mmHg with proteinuria of at least 300 mg after 20 weeks of pregnancy | Tranquilli A, Dekker G, Magee L, Roberts J, Sibai B, Steyn W; et al. The classification, diagnosis and anagement of the hypertensive disorders of pregnancy: a revised statement from the ISSHP. Pregnancy Hypertension. 2014;4(2):97–104. | (Khaliq et al., 2018)  Witvrouwen 2021 |
| Preeclampsia was defined by clinical diagnosis with systolic blood pressure >140 mmHg or diastolic blood pressure ≥90 mmHg and proteinuria ≥0.3 g/24 h | not reported | (Kim et al., 2018) |
| not reported | **ACOG 2013** | (Li et al., 2018) |
| PE was defined as hypertension (blood pressure ≥140/90 mmHg) in two determinations, 4 hours apart associated with proteinuria (> 300 mg/24 h or ≥1+, according to a routine urinalysis) after 20 weeks of gestation in previously normotensive women. In the absence of proteinuria, PE was characterized based on the new modified guidelines recommended by the task force on Hypertension in pregnancy. | C.M. Brown, V.D. Garovic, Mechanisms and management of hypertension in pregnant women, Current 317 hypertension reports 13(5) (2011) 338-46.  **ACOG 2013**. American College of, Gynecologists, P. Task Force on Hypertension in, Hypertension in pregnancy. Report of the American College of Obstetricians and Gynecologists' Task Force on Hypertension in Pregnancy, Obstetrics and gynecology 122(5) (2013) 1122-31. | (Lykoudi et al., 2018) |
| Preeclampsia was defined as the presence of hypertension (systolic blood pressure (BP) ≥ 140 mmHg or diastolic BP ≥ 90 mmHg on at least two occasions, 4 hours to 1 week apart) and proteinuria (≥ 300 mg in a 24-hour urine collection or one dipstick measurement ≥ 2+). | not reported | (Motawi et al., 2018) |
| not reported | Cunningham FG, Leveno KJ, Bloom SL, Spong CY, Dashe JS,Hoffman BL, CaseyBM and Sheffield JS: Hypertensive disorders.In: Williams Obstetrics. 24th edition. McGraw‑Hill Education,New York, NY, pp729‑731, 2014 | (Shen et al., 2018) |
| Preeclampsia was defined according to the guidelines of the International Society for the Study of Hypertension in Pregnancy | Brown MA, Lindheimer MD, de Swiet M, Van Assche A, Moutquin JM. The classification and diagnosisof the hypertensive disorders of pregnancy: statement from the international society for the study ofhypertension in pregnancy (ISSHP). Hypertens Pregnancy 2001;20:IX±XIV. | (Winger et al., 2018)  Martinez-Fierro 2021 |
| PE was defined as blood pressure greater than 140/90mmHg with proteinuria >300mg/24 h or at least 1 g/L (++) on dipstick testing | not reported | (Biró et al., 2019) |
| PE was defined as systolic or diastolic blood pressure higher than 140/90mmHg and proteinuria above 0.3 g/24 hr, according to internationally accepted criteria | Roberts, J. M., August, P. A., Bakris, G., Barton, J. R., Bernstein, I. M.,Druzin, M.,Ngaiza, K. (2013). Hypertension in pregnancy. Report ofthe American College of Obstetricians and Gynecologists’ task force on hypertension in pregnancy. American College of Obstetricians and Gynecologists, 122(5), 1122–1131. | (Eghbal-Fard et al., 2019) |
| PE was diagnosed after 20 weeks gestation, systolic pressure ≥140 mm Hg and/or diastolic pressure ≥90 mm Hg at rest on 2 occasions at least 4 to 6 hours apart, and urinary protein ≥0.3 g/24 hours | not reported | (Huang et al., 2019) |
| not reported | Zhou R, Tardivel A, Thorens B, Choi I, Tschopp J. Thioredoxininteractingprotein links oxidative stress to inflammasome activation.Nat Immunol. 2010;11:136–40. | (Liu et al., 2019c) |
| not reported | Davison JM and Lindheimer MD. **ISSHP**, an association saving the lives of pregnant women and their babies: (the international society for the study of hypertensions in pregnancy (ISSHP): its history). Pregnancy Hypertens 2017; 7: 2-28. | (Ma et al., 2019) |
| not reported | M.A. Brown, M.D. Lindheimer, M. de Swiet, A. Van Assche, J.M. Moutquin, The classification and diagnosis of the hypertensive disorders of pregnancy: statement from the International Society for the Study of Hypertension in Pregnancy (ISSHP), Hypertens. Pregnancy 20 (1) (2001) IX–XIV. | (Martinez-Fierro et al., 2019)  Huang 2020 |
| preeclampsia is defined as increased blood pressure (≥140 mmHg systolic, or ≥90 mmHg diastolic on 2 or occasions at least 4 h apart) in addition to any of following features: significant proteinuria (≥0.3 g/L in 24 or ≥1+), thrombocytopenia (low blood platelet count, platelet count≤105/micro liter), renal insufficiency(serum creatinine concentrations≥97.24 μmol/L), and impaired liver function (doubling of blood transaminases concentration) after 20 weeks of gestation in a woman with a previously normal blood pressure | **ACOG 2013** | (Nejad et al., 2019)  Wu 2020 |
| not reported | ISSHP no reference | (Shi et al., 2019) |
| Preeclampsia was defined as hypertension (systolic blood pressure ≥140 mm Hg or/and diastolic blood pressure ≥90 mmHg) and proteinuria (≥300 mg in a 24-h urine collection or 1 dipstick measurement of ≥2+) occurring after 20 weeks of gestation. | not reported | (Wang et al., 2019a) |
| Pre‐eclampsia was clinically described as hypertension (systolic blood pressure ≥140mmHg and/or diastolic blood pressure ≥90mmHg) after 20 weeks in previously normotensive women, with proteinuria (>300mg/24 hr), one or more other organ system dysfunction or fetus growth restriction | 1) Knight, M., Nair, M., Tuffnell, D., Shakespeare, J., Kenyon, S. K. J. (Eds).(2017). Saving Lives, Improving Mothers’ Care‐Lessons learned toinform maternity care from the UK and Ireland Con dential Enquiries into Maternal Deaths and Morbidity, on behalf of M‐U 2013–15.  2 ) Say, L., Chou, D., Gemmill, A., Tunçalp, Ö., Moller, A.‐B., Daniels, J. Alkema, L. (2014). Global causes of maternal death: A WHO systematic analysis. The Lancet Global Health, 2, e323–e333.  3) Tranquilli, A. L., Dekker, G., Magee, L., Roberts, J., Sibai, B. M., Steyn, W., Brown, M. A. (2014). The classification, diagnosis and management ofthe hypertensive disorders of pregnancy: A revised statement from the ISSHP. Pregnancy Hypertension, 4, 97–104 | (Wang et al., 2019c) |
| PE was defined as increased systolic blood pressure (≥160 mmHg) and/or increased diastolic blood pressure (≥ 110 mmHg) observed after 20 weeks gestation; low platelet counts (< 100×109/L); impaired liver function (serum transaminase level over 2 fold chance than normal value); impaired kidney function(Scr>97.2 μmol/L or over 2 fold-chance than normal value); pulmonary edema; newly occurred brain and visual impairment | not reported | (Wang et al., 2019d) |
| not reported | ACOG 2002 A.C.O.P. Bulletins-Obstetrics, ACOG practice bulletin. Diagnosis and management of preeclampsia and eclampsia. Number 33, January 2002, Obstet. Gynecol. 99 (1) (2002) 159–167. | (Xiaobo (赵肖波) et al., 2019) |
| Systolic blood pressure ≥140 mmHg or diastolic blood pressure ≥90 mmHg at 20 weeks post‑gestation in a pregnant woman whose blood pressure was previously normal, 24 h urine protein ≥0.3 g | Redman CW and Sargent IL : Latest advances in understanding preeclampsia. Science 308: 1592‑1594, 2005. | (Xie et al., 2019) |
| high blood pressure and a high protein content in the urine | not reported | (Yang and Guo, 2019) |
| not reported | ACOG 2002 | (Yang and Meng, 2019) |
| not reported | ISSHP no reference | (Youssef and Marei, 2019) |
| not reported | Di Lorenzo G, Ceccarello M, CecottiV,Ronfani L, Monasta L, VecchiBrumatti L, Montico M and D'Ottavio G: First trimester maternal serum PIGF, free β‑hcg, PAPP‑A, PP‑13, uterine artery doppler and maternal history for the prediction of preeclampsia. Placenta 33: 495‑501, 2012. | (Zhong et al., 2019) |
| Preeclampsia was diagnosed by the elevation of blood pressure above  140/90 mmHg measured at four-hour intervals and proteinuria above  0.3 g/day after 20 gestational weeks | Roberts JM, Pearson G, Cutler J, Lindheimer M; NHLBI Working Group on Research on Hypertension During Pregnancy. Summary of the NHLBI Working Group on Research on Hypertension During Pregnancy. Hypertension. 2003 Mar;41(3):437-45. | Dong 2019 |
| PE is defined as a blood pressure of more than 140/90 mmHg or mild hypertension with more than 3g protein/24 hour urine | Not reported | Lv 2019  Li H, 2020 |
| PE patients were diagnosed by systolic/diastolic blood pressure (> 140/90 mm Hg) and proteinuria | Not reported | Qian 2019 |
| PE was defined as sustained systolic blood pressure of  ≥ustained systolic blood pressure ofic/diastolic blood pressure  on two separate readings; proteinuria measurement of 1+ or more  on dipstick, or 24-hour urine protein collection with ia measure  the specimen | Not reported | Xu 2019 |
| Not reported | Niu, J. M., Lei, Q., Lü, L. J., Wen, J. Y., Lin, X. H., Duan, D. M., … Yi, J. (2013).  Evaluation of the diagnostic criteria of gestational metabolic syndrome and analysis of the risk factors. Zhonghua fu chan ke za  zhi, 48(2), 92–97. | Yuan 2019 |
| PE was defined as systolic blood pressure ≥140 mmHg and/or diastolic blood pressure ≥90 mmHg after 20 weeks of gestation, whereas women with previous normal blood pressure had proteinuria ≥1+ (300 mg/24 h) after preg­nancy | Chaiworapongsa T, Chaemsaithong P, Yeo L and Romero R: Pre‑eclampsia part 1: Current understanding of its pathophys­iology. Nat Rev Nephrol 10: 466‑480, 2014. | Dong 2020 |
| PE was defined as systolic blood pressure ≥140 mmHg and/or diastolic blood pressure ≥90 mmHg on at least two occasions 4 h apart but no more than 7 days apart, which developed after 20 weeks of gestation in previously normotensive women; proteinuria was defined as ≥300 mg protein in a 24 h urine specimen | ISSHP 2018 | Kim 2020  Li Q, 2020 |
| PE were recruited during their first pregnancy with systolic blood pressure hich developed after = 0.133 kPa), diastolic b lood p ressure egnancy with systolic blood pressure hich developed after 20 weeks of gestation in previousl+) and the above | Not reported | Yang 2020 |
| New onset of systolic blood pressure (SBP) >140mmHg or  diastolic blood pressure (DBP) >90mmHg at >20 weeks of gestation accompanied by 24hrs proteinuria >300mg (>1+ on dipstick), in at least two random urine samples collected 4-6hrs apart | Uzan J, Carbonnel M, Piconne O, Asmar R, Ayoubi JM. Preeclampsia: pathophysiology, diagnosis, and management. Vasc Health Risk Manag. 2011; 7:467-74. | Ali, 2021 |
| Not reported | Gou WL, Yang HX. Hypertensive disorderS complicating pregnancy. Obstet Gynecol. 2019;234(7):11149–55. | Cai 2021 |
| PE was defined as patients who had no history of preexisting or  chronic hypertension, but exhibited systolic pressure >140 mmHg or diastolic pressure > 90 mmHg at least 2 occasions, concurrent with proteinuria (> 2 g per 24 h in 2 samples obtained at > 4 h intervals) after 20 weeks of gestation | Brkic J, Dunk C, O’Brien J, Fu G, Nadeem L, Wang YL, et al. MicroRNA-218-5p promotes endovascular trophoblast differentiation and spiral artery remodeling. Mol Ther. 2018;26(9):2189–205. | Peng, 2021 |
| Not reported | Niu JM, Lei Q, Lu LJ, et al. Evaluation of the diagnostic criteria of gestational metabolic syndrome and analysis of the risk factors.  Zhonghua Fu Chan Ke Za Zhi. 2013;48:92-97. | Xu 2021 |
| PE showed blood pressure over 140/90 mmHg after 20 gestation weeks and proteinuria higher than 0.3 g/24 h or at least ‘++’ by semi quantitative dipstick testing in the absence of urinary tract infection | Heydarlou, H., et al., 2019. Investigation of follicular helper t cells, as a novel player, in preeclampsia. J. Cell. Biochem. 120, 3845–3852. | Zolfaghari, 2021 |

Table S3. Diagnostic criteria for less and more severe preeclampsia

| **Author** | **Less severe PE** | **Criteria** | **More severe PE** | **Criteria** |
| --- | --- | --- | --- | --- |
| Y. L. Hu 2009 | / | / | sLOPE | Severe PE was defined by the presence of ≥1 of the following: 1) severe gestational hypertension (systolic pressure >160 mm Hg or diastolic blood pressure >110mmHg on ≥2 occasions after gestational week 20); or 2) severe proteinuria (≥5 g protein in a 24-h urine specimen) |
| X. M. Zhu 2009 | mPE | Mild PE was defined as maternal systolic blood pressure of ≥140 mm Hg and/or diastolic blood pressure of ≥90mmHg on 2 occasions separated by 6 hours and significant proteinuria ( ≥300 mg of protein in a 24-hour urine specimen or ≥1+ by dipstick) after 20 weeks of gestation | sPE | Severe PE was defined as either severe hypertension(systolic blood pressure of ≥160mmHgand/or diastolic blood pressure of ≥110mm Hg on at least 2 occasions 6 hours apart) plus mild proteinuria or mild hypertension plus severe proteinuria ( >2 g/24 hr or 2 by dipstick). |
| L. Guo 2011 | mPE | not reported | sPE | not reported |
| Q. Yang 2011 | mPE | not reported | sPE | not reported |
| L. Liu 2012 | / | / | sLOPE | not reported |
| L. Wu 2012 | / | / | sLOPE | Severe PE was defined as either severe hypertension (either a systolic blood pressure of 160 mmHg or higher or a diastolic blood pressure of 110 mmHg or higher on two occasions at least 6 h apart while the patient was on bed rest) or severe proteinuria (either urinary excretion of 5 g protein or higher in a 24 h urine specimen or 3C protein or greater in two random urine samples collected at least 4 h apart), or both, were present after 20 weeks of gestation |
| Y. Zhang 2012 | mPE | mPE was defined as maternal systolic blood pressure 140 mm Hg and/or diastolic blood pressure 90 mm Hg on two occasions, separated by 6 hrs, and proteinuria 300 mg in a 24-hr period after 20-week gestation | sPE | sPE was considered as either sever hypertension (maternal blood pressure>160/110 mm Hg on two separate readings at least 6 hrs apart) or severe proteinuria (>2 g protein in a 24-h period). |
| I. Hromadnikova 2013 | LOPE mPE | not reported | EOPE sPE | Severe preeclampsia was diagnosed by the presence of one or more of the findings according to the guidelines of ACOG Committee |
| H. L. Li 2013 | mPE | mPE was defined as maternal systolic blood pressure of ⩾140mmHg and/or diastolic blood pressure of ⩾90mmHg on 2 occasions separated by 6 hours and significant proteinuria (⩾300mg of protein in a 24-hour urine specimen or ⩾1+ by dipstick) after 20 weeks of gestation | sPE | sPE was defined as either severe hypertension (systolic blood pressure of ⩾160mmHg and/or diastolic blood pressure of ⩾110mmHg on at least 2 occasions 6 hours apart) plus mild proteinuria or mild hypertension plus severe proteinuria (⩾2 g/24hr or ⩾2+ by dipstick) |
| P. F. Li 2013 | / | / | sLOPE | Severe PE was defined by the presence of more than one of the following: (i) severe gestational hypertension (systolic pressure>160mmHg or diastolic blood pressure>110mmHg on two or more occasions after gestational week 20), (ii) severe proteinuria (5 g of protein in a 24 h urine specimen), (iii) oliguria <500 ml in 24 h, (iv) cerebral or visual disturbances, (v) pulmonary edema or cyanosis, (vi) epigastric or right upper-quadrant pain, (vii) impaired liver function, (viii) thrombocytopenia or (ix) fetal growth restriction |
| Y. S. Chen 2014 | mPE | mPE was defined as systolic pressure ≥140 mmHg or diastolic pressure ≥90 mmHg; 24 h proteinuria ≥300mg | sPE | sPE was defines as systolic pressure ≥160 mmHg or diastolic pressure ≥100 mmHg; 24 h proteinuria ≥2g |
| A. Luque 2014 | / | / | EOPE | not reported |
| M. S. Weedon-Fekjaer 2014 | LOPE | after 34 weeks | EOPE sPE | before 34 gw Severe preeclampsia was defined by the American College of Obstetricians and Gynecologist's criteria |
| E. E. Winger 2014 | LOPE | ≥ 34 gw | EOPE | < 34 gw |
| G. Zhao 2014 | / | / | sLOPE | not reported |
| I. Hromadnikova 2015 | LOPE mPE | PE after 34 weeks of gestation not reported | EOPE sPE | PE before 34 weeks of gestation sPE was defined as presence of one or more of the following findings: 1) a systolic blood pressure > 160 mmHg or a diastolic blood pressure > 110 mmHg, 2) proteinuria greater than 5g of protein in a 24-hour sample, 3) very low urine output (less than 500 ml in 24 h), 4) signs of respiratory problems (pulmonary oedema or cyanosis), 5) impairment of liver function, 6) signs of central nervous system problems (severe headache, visual disturbances),7) pain in the epigastric area or right upper quadrant, 8) thrombocytopenia, and 9) the presence of severe fetal growth restriction |
| I. Hromadnikova 2015 | LOPE mPE | PE after 34 weeks of gestation not reported | EOPE sPE | PE before 34 weeks of gestation sPE was defined as presence of one or more of the following findings: 1) a systolic blood pressure >160 mmHg or a diastolic blood pressure > 110 mmHg, 2) proteinuria greater than 5g of protein in a 24-hour sample, 3) very low urine output (less than 500 ml in 24 h), 4) signs of respiratory problems (pulmonary edema or cyanosis), 5) impairment of liver function, 6) signs of central nervous system problems (severe headache, visual disturbances),7) pain in the epigastric area or right upper quadrant, 8) thrombocytopenia, and 9) the presence of severe fetal growth restriction |
| K. Miura 2015 | LOPE | Women in whom the onset of PE was after 32weeks of gestation | EOPE sPE | Women in whom the onset of PE was before 32weeks of gestation were defined as having the early onset type  sPE was defined as the presence of one or more of the following criteria: severe gestational hypertension (SBP >160mmHg or DBP >110mmHg on more than two occasions after 20weeks of gestation) or ≥2 g protein in a 24-h urine specimen. |
| M. S. Murphy 2015 | LOPE mPE | >34 weeks gestation not reported | EOPE sPE | <34 weeks gestation Severe PE diagnosis criteria were met if in addition to the criteria for PE, an individual presented with any one of the following: two stable measurements of systolic blood pressure 160 mmHg or diastolic blood pressure 110 mmHg a minimum of at least six hours apart, proteinuria of 5g/24 hours or 3+ pm repeat dipstick, oliguria ≤500mL/24 hours, cerebral or visual disturbances, epigastric pain, thrombocytopenia <150,0009/L, AST >46 U/L, ALT >40 U/L, serum creatinine >106 umol/L, pulmonary cyanosis or intrauterine growth restricted baby. |
| S. Yang 2015 | mPE | not reported | sPE | not reported |
| I. Hromadnikova 2016 | LOPE mPE | >34 weeks gestation | EOPE sPE | <34 weeks gestation Severe preeclampsia was diagnosed by the presence of one or more of the following findings: 1) a systolic blood pressure >160 mm Hg or a diastolic blood pressure >110 mm Hg, 2) proteinuria greater than 5 g of protein in a 24-hour sample, 3) very low urine output (less than 500 mL in 24 h), 4) signs of respiratory problems (pulmonary edema or cyanosis), 5) impairment of liver function, 6) signs of central nervous system problems (severe headache, visual disturbances), 7) pain in the epigastric area or right upper quadrant, 8) thrombocytopenia, and 9) the presence of severe fetal growth restriction |
| T. X. Hu 2016 | / | / | EOPE sPE | not reported blood pressure ≥160/110 mmHg and proteinuria ≥2.0 g/24 h |
| S. Ospina-Prieto 2016 | LOPE | at or after 34 gw | EOPE | before 34 gw |
| V. C. Sandrim 2016 | mPE | not reported | sPE | Severe pre-eclampsia was defined as ≥160 mmHg systolic or ≥110 mmHg diastolic, on two or more measurements at least 6 hours apart; and/or proteinuria ≥2 g/24 hour |
| W. W. Yang 2016 | / | / | sEOPE | sEOPE was defined as de-novo occurrence of systolic blood pressure (SBP) ≥160 mmHg or diastolic blood pressure (DBP) ≥110 mm Hg on at least two occasions, accompanying severe proteinuria (≥3+ or ≥2 g/24h) at 20±34 weeks of gestation |
| S. Adel 2017 | mPE | systolic blood pressure (140/90–160/110mmHg) as measured twice, using an appropriate cuff, 6 h apart and according to proteinuria in a 24-hour protein excretion ≥300 mg/d or ≥1+by dipstick on two random urine samples collected at least 6 h apart. | sPE | systolic blood pressure ≥160/110mmHgas measured twice, using an appropriate cuff, 6 h apart and according to proteinuria in a 24-hour protein excretion ≥5 g/d or ≥3+by dipstick on two random urine samples collected at least 6 h apart. |
| S. Gao 2017 | / | / | EOPE | delivery before 35 weeks of gestation |
| T. Gunel 2017 | / | / | sLOPE | sLOPE was defined as either severe hypertension (systolic blood pressure of >160mmHg and/or diastolic blood 15 pressure of >110mmHg on at least 2 measurements 6 hours apart) severe proteinuria, or both,16 presenting after 20 weeks of gestation |
| L. Han 2017 | mPE | not reported | sPE | not reported |
| I. Hromadnikova 2017 | LOPE mPE | after 34 weeks not reported | EOPE sPE | before 34 gw presence of one or more of the following findings: 1) a systolic blood pressure > 160 mmHg or a diastolic blood pressure > 110 mmHg, 2) proteinuria greater than 5g of protein in a 24-hour sample, 3) very low urine output (less than 500 ml in 24 h), 4) signs of respiratory problems (pulmonary edema or cyanosis), 5) impairment of liver function, 6) signs of central nervous system problems (severe headache, visual disturbances), 7) pain in the epigastric area or right upper quadrant, 8) thrombocytopenia, and 9) the presence of severe fetal growth restriction |
| D. S. Jairajpuri 2017 | mPE | Mild PE patients had SBP ≥ 140mmHg, DBP ≥90 mmHg on 2 occasions separated by 6 hours, and proteinuria (≥300 mg of protein in a 24-hour urine specimen or ≥1+ by dipstick) | sPE | severe PE was confirmed if one of more of these conditions were met: persistent SBP ≥160mmHg and/or DBP ≥110mmHg on least at 2 occasions 6 hours apart; proteinuria (≥2g/24 hour or≥2+ by dipstick); and serum creatinine ≥106 mmol/L (except patients whose serum creatinine was increased before the test), low platelet count (<105/mL); increased lactate dehydrogenase; elevated liver enzyme function (ALT, AST), and persistent upper abdominal pain |
| T. M. Lu 2017 | mPE | not reported | sPE | not reported |
| H. X. Meng 2017 | / | / | EOPE | not reported |
| N. V. Nizyaeva 2017 | LOPE | / | EOPE | / |
| X. Shao 2017 | / | / | sEOPE sLOPE | Severe preeclampsia was defined as a pregnancy having no history of preexisting or chronic hypertension but showing systolic blood pressure of ≥160 mm Hg or diastolic blood pressure of ≥110 mm Hg on at least 2 occasions, accompanied by significant proteinuria (≥2 g/24 hours or 3+ by dipstick in 2 random samples collected at >4-hour intervals) or problems in multiple organs (such as pulmonary edema, seizures, oliguria, abnormal liver enzymes associated with persistent epigastric or right upper quadrant pain, or persistent and severe central nervous system symptoms) after the 20^th^week of gestation |
| X. Yang 2017 | mLOPE | Late onset PE (≥32 weeks) was defined as those patients who displayed gestational hypertension and proteinuria without a history of hypertension, presenting with systolic blood pressure≥140 mm Hg and/or diastolic blood pressure ≥90 mm Hg. | sLOPE | not reported |
| T. Gunel 2018 | / | / | sLOPE | not reported |
| M. L. Martinez-Fierro 2018 | LOPE mPE | more than 34 + 1 gw | EOPE sPE | before 34 gw PE was considered as severe ifthe patient had a blood pressure≥ 160 mmHg systolic or ≥ 110 mmHg diastolic on two occasions at least 6 h apart while the patient was at bed rest, and a proteinuria of 5 g or more in a 24-h urine specimen, or 3 + or greater in two random urine samples collected at least 4 h apart |
| T. M. K. Motawi 2018 | LOPE | ≥ 37 gw term | EOPE | < 37gw preterm |
| N. V. Nizyaeva 2018 | LOPE | after 34 weeks | EOPE | before 34 gw |
| A. V. Timofeeva 2018 | mEOPE mLOPE | after 34 weeks | sEOPE sLOPE | 24-34gw Severe PE was diagnosed by the presence of one or more of the following clinical manifestations: 1) systolic blood pressure above 160 mm Hg or diastolic pressure above 110mmHg, 2) proteinuria above 5 g/l, 3) very low levels of daily diuresis (less than 500 ml in 24 h), 4) respiratory disorders (pulmonary oedema or cyanosis), 5) liver dysfunction, 6) central nervous system malfunction (severe headache or visual disturbances), 7) pain in the epigastric region or right upper quadrant, 8) thrombocytopenia and 9) the presence of severe intrauterine growth retardation |
| H. Wang 2018 | not reported | not reported | sEOPE | not reported |
| Y. Wang 2018 | LOPE | at or after 34 gw | EOPE | before 34 gw |
| Z. Awamleh2019 | / | / | EOPE | prior to 34 gw |
| K. Dong 2019 | LOPE | after 34 weeks | EOPE | before 34 gw |
| M. Hocaoglu 2019 | mEOPE mLOPE | PE occurring at 34 weeks or later | sEOPE sLOPE | PE occurring at less than 34 weeks |
| I. Hromadnikova 2019 | mPE | not reported | sPE | not reported |
| M. L. Martinez-Fierro 2019 | LOPE mPE | later than 34+1 gw | EOPE sPE | present before 34 WG Severe PE was considered as that in which the patient had a blood pressure ≥160mm Hg systolic or≥110mm Hg diastolic on two occasions at least 6 h apart while the patient was at bed rest, and a proteinuria of 5 g or more in a 24-h urine specimen, or 3+ or greater in two random urine samples collected at least 4 h apart |
| P. Pillay 2019 | LOPE | New onset hypertension (diastolic blood pressure of >90 mm Hg and systolic blood pressure of >140 mm Hg) and proteinuria (≥300 mg) at ≥34 weeks gestational age. | EOPE | New onset hypertension (diastolic blood pressure of ≥90 mmHg and systolic blood pressure of ≥140 mm Hg) and proteinuria (≥300 mg) at ≤33 weeks. |
| Z. Xiaobo 2019 | LOPE | after 34 weeks | EOPE | before 34 weeks of gestation |
| H. M. G. Youssef 2019 | mPE | Mild PE was defined as maternal systolic blood pressure of ≥140 mmHg and/or diastolic blood pressure of ≥90 mmHg, with significant proteinuria (≥1+ by dipstick) at 20 to 34 weeks of gestation | sPE | Severe Pe was defined as blood pressure values>160 mmHg systolic or 110 mmHg diastolic and the amount of proteinuria is considered not useful to define the severity, and if thrombocytopenia increase in liver or kidney functions, persistent epigastric pain, pulmonary edema, cerebral or visual disturbances are present, the patients are categorized as severe PE subgroup even with mild hypertension |
| Ayoub 2019 | mPE | Not reported | sPE | Not reported |
| Demirer 2019 | LOPE | Preeclampsia occurring at less than 34 weeks of gestation | EOPE | PE that occurred at 34 weeks |
| Dong 2019 | LOPE | Not reported | EOPE | Not reported |
| Lip 2019 | / | / | EOPE | Women delivered before 34 GW |
| Devor 2020 | LOPE | PE diagnosed after 34 GW | / | / |
| Han 2020 | Mild EOPE | systolic blood pressure ≥140 mmHg and/or diastolic blood pressure ≥90 mmHg, urine protein ≥0.3 g/24 h or urine protein ≥.(+) before 34 weeks of pregnancy | Severe EOPE | hypertension and proteinuria after 20 weeks of pregnancy, accompanied by at least one of the following clinical signs: 1. Systolic blood pressure ≥160–180 mmHg or diastolic blood pressure ≥110 mmHg; 2. urinary protein ≥5 g/24 h or random urine protein ≥r(+++); 3. central nervous system dysfunction, mental state changes and severe headache and cerebrovascular accident; 4. blurred vision, spotted hemorrhage; 5. liver dysfunction transaminase increased at least twice; 6. renal dysfunction with oliguria; 7. thrombocytopenia; 8. Subhepatic hemorrhage or liver rupture; 9. coagulopathy; and 10. microvascular hemolysis  combining fetal growth restriction, oligohydramnios and placental abruption. |
| Huang 2020 | / | / | sPE | According to the International Association for Pregnancy and Hypertension Research (ISSHP) criteria, the patient’s PE status is considered severe |
| Jelena 2020 | mPE  LOPE | Not reported  At or after 34 GW | /  EOPE | /  Before 34 GW |
| Mavreli 2020 | LOPE | >34 GW | / | / |
| Whigham 2020 | LOPE | Not reported | sEOPE | Not reported |
| Wu G, 2020 | mPE | Not reported | sPE | Not reported |
| Ali, 2021 | LOPE | PE ranged from 28 to 32 weeks | EOPE | PE ranged from 32.1 to 40 weeks |
| Jairajpuri, 2021 | mPE | Mild PE patients had systolic blood pressure ≥140 mm Hg, diastolic blood pressure ≥90 mm Hg on 2 occasions separated by 6 h, and proteinuria (≥300 mg of protein in a 24-h urine specimen or ≥1+ by dipstick) | sPE | severe PE was confirmed if one or more of these conditions were met: persistent systolic blood pressure ≥160 mm Hg and/or diastolic blood pressure ≥110 mm Hg on least at 2 occasions 6 h apart; proteinuria (≥2 g/24 h or ≥2+ by dipstick); and serum creatinine ≥106 mmol/l (except patients whose serum creatinine was increased before the test), low platelet count (*<*105/ml); increased lactate dehydrogenase; elevated liver enzyme function (ALT, AST), and persistent upper abdominal pain |
| Kolkova, 2021 | LOPE  mPE | late-onset PE (after 34 weeks of pregnancy)  mPE criteria not reported | EOPE  sPE | early (before 34 weeks of pregnancy)  Severe PE was defined as severe gestational hypertension (systolic blood pressure ≥160 mmHg or diastolic ≥110 mmHg on two occasions at least four hours apart), based on ACOG criteria |
| Liao, 2021 | / | / | mEOPE  sEOPE | The diagnostic criteria for EOPE were: (1) first  detected hypertension after 20-week gestation (systolic blood pressure ≥140 mm Hg or diastolic blood pressure ≥140 mm Hg); (2) urinary protein > 0.3 g/24 h or random urinary protein > 1+; (3) new-onset hypertension or one or more of adverse characteristics [including thrombocytopenia (<100000/lL), renal injury (creatinine > 1.1 mg/dL), pulmonary edema, liver dysfunction, cerebral or visual impairment. Patients showing systolic blood pressure ≥160 mm Hg or diastolic blood pressure ≥110 mm Hg accompanied with mild proteinuria, or mild hypertension with severe proteinuria (>2 g/24 h or random  urinary protein > 2+) were included in the severe EOPE group, and the rest were included in the mild EOPE group |
| Luizon, 2021 | mPE | Not reported | sPE | Not reported |
| Witvrouwen, 2021 | / | / | EOPE | Gestational age <34 weeks |
| Xu, 2021 | / | / | EOPE  sPE | Not reported |
| Yu, 2021 | / | / | sPE | Diagnosis of severe PE was based on the definition in Williams Obstetrics (23^rd^ Edition) |

Table S4. Quantification methods and housekeeping gene

| Ref No | First method (which) | Validation | Validation population | Housekeeping  gene |
| --- | --- | --- | --- | --- |
| (Pineles et al., 2007) | TaqMan MicroRNA Assays Human Panel–Early Access Kit (Applied Biosystems, Foster City, CA), with 2-step qRT-PCR assay | Not reported |  | 5S rRNA for qRT-PCR |
| (Hu et al., 2009) | miRNA microarray chip | yes (TaqMan MicroRNA Assays with qRT-PCR) | same | U6 |
| (Zhu et al., 2009) | miRNA microarray chip (Exiqon) | yes (qRT-PCR) | same | U6 |
| (Zhang et al., 2010) | Sybr green qRT-PCR (Toyobo co.,) | Cell migration assay, Sybr green RT-PCR |  | U6 |
| (Cheng et al., 2011) | Northern blot | Differential expression profile of microRNAs | same | U6 |
| (Enquobahrie et al., 2011) | miRNA microarray chip | pregnancies vs normal pregnancies | Not reported | has-miRNA-525-5p |
| (Gunel et al., 2011) | High-specificity TaqMan qRT-PCR | Not reported |  | Not reported |
| (Guo et al., 2011) | Small RNA library preparation and sequencing (SOLiDTM sequencing platform) | Gene Ontology enrichment analysis |  | Not reported |
| (Mayor-Lynn et al., 2011) | miRNA microarray chip | yes (qRT-PCR) | not clear | U6 and 18S rRNA |
| (Yang et al., 2011) | next-generation sequencing (ABI SOLID sequencing) | Not reported |  | Not reported |
| (Bai et al., 2012) | qRT-PCR | qRT-PCR, Western blot, Luciferase Dual Assay |  | U6 |
| (Hromadnikova et al., 2012) | TaqMan MicroRNA Assay | Not reported |  | Not reported |
| (Takizawa et al., 2012) | HTS (high-throughput sequencing analysis), TaqMan Human MicroRNA Arrays version 2.0, RT-PCR | yes (TaqMan Human MicroRNA Array,Luciferase Assay,ELISA) | same | Not reported |
| (Lázár et al., 2012) | TaqMan Human MicroRNA Arrays version 2.0, RT-PCR | Not reported |  | snoRNA202 |
| (Liu et al., 2012) | RT-PCR | Western Blotting and ELISA, Transient Transfection and Reporter Assay |  | U6 |
| (Muralimanoharan et al., 2012) | Not reported | Not reported |  | 18S rRNA |
| (Wang et al., 2012a) | miRNA microarray analysis | yes (qRT-PCR,,transfection of miRNA precursors or antagomirs into endothelial and BeWo cells) | same | U18 |
| (Wang et al., 2012b) | Agilent human miRNA microarray,qRT-PCR | yes (qRT-PCR, miRNA gene network analysis) | same | U6 |
| (Wu et al., 2012) | Agilent miRNA microarray chip | yes (Real-time quantitative stem-loop RT-PCR) | same | cel-miR-39, cel-miR-54, and cel-miR-238 |
| (Zhang et al., 2012) | SYBR-Green qRT-PCR | Plasmid constructs and luciferase activity assays, Western blot, Transwell migration and matrigel invasion assay, Chromatin immunoprecipitation assay, Evaluation of NF-B p50 activity |  | U6 |
| (Anton et al., 2013) | qRT-PCR | EVT Cell Treatments for Invasion Assays |  | U6 |
|  | qRT-PCR | Not reported |  | U6 |
| (Betoni et al., 2013) | miRNA microarray | yes (qRT-PCR) | partially independent | U6 |
| (Choi et al., 2013) | miRNA microarray | yes (qRT-PCR, MicroRNA predicted target genes and functional analysis) | same | U6 |
| (Fu et al., 2013) | qRT-PCR | MicroRNA-376c Promoted Trophoblast Outgrowth and Invasion, MicroRNA-376c Targets ALK5 and ALK7, MicroRNA-376c Impaired TGF-β/Nodal Signaling in Trophoblast Cells |  | U6 |
|  | qRT-PCR | Not reported |  | U6 |
|  | qRT-PCR | Not reported |  | U6 |
| (Guo et al., 2013) | Illumina array, qRT-PCR | yes (qRT-PCR) | different | Not reported |
| (Hromadnikova et al., 2013) | TaqMan MicroRNA Assay | yes (qRT-PCR) | same | Cel-miR-39 |
| (Kumar et al., 2013) | TaqMan MicroRNA Assay | qRT-PCR,Luciferase Assay, Adenoviral overexpression and RNA interference, Chromatin immunoprecipitation assay |  | Not reported |
| (Li et al., 2013a) | SOLiD Sequencing | yes (qRT-PCR) | different | Not reported |
| (Li et al., 2013b) | TaqMan MicroRNA Assay | miRNA target prediction and GO, Transfection experiments, Cell proliferation, cell cycle and apoptosis assays, ELISA, Cell invasion assay, Plasmid construct and luciferase analysis |  | U6 |
| (Yan et al., 2013) | qRT-PCR | qRT‐PCR FOR miR‐126 and target genes, Western Immunoblotting, Cell Proliferation, Colony forming and Cell Diffrenetiation Assay,Migration Assay, *In vivo* vasculogenesis, Immunohystochemical analysis |  | U6 |
| (Campos et al., 2014) | TaqMan miRNA qRT-PCR assays | Not reported |  | Cel-miR-39 |
| (Chen et al., 2014) | qRT-PCR | Western Blotting,Cell transfection |  | U6 |
| (Doridot et al., 2014) | SYBR green qRT-PCR preceded by a polyadenylation step | Plasmid construction, Cell culture, hypoxia, and transfection, Luciferase assay |  | 5S rRNA and SDHA |
| (Hong et al., 2014) | qRT-PCR | Cell culture and transfection, qRT-PCR, Western Blotting |  | U6 |
| (Lalevée et al., 2014) | TaqMan qRT-PCR | UTR cloning and dual luciferase assay, Transient transfection siRNA and mimics,small RNA libraries for high-throughput sequencing, Western Blotting, Immunofluorescence in BeWo cells |  | U6 |
| (Li et al., 2014a) | qRT-PCR | In vitro tranwell insert invasion assay, Western blotting, Luciferase assays |  | U6 |
| (Li et al., 2014b) | qRT-PCR | Western blotting, Plasmids and transfection, Cell survival and proliferation assay, Transwell invasion and migration assay |  | U6 |
| (Luo et al., 2014) | qRT-PCR | Direct Target of miR-210 in Human Trophoblast Cells, Cell Invasion in HTR8/SV neo Cells |  | U6 |
| (Luque et al., 2014) | OpenArray Real-Time qPCR Analysis Software | yes (real-time quantitative stem-loop RT-PCR) | same | Cel-miR-54 |
| (Ura et al., 2014) | TaqMan MicroRNA Assay | yes (qRT-PCR) | same | Cel-miR-39 |
| (Wang et al., 2014) | In situ hybridization | yes (qRT-PCR, In situ hybridization, Cell Culture and Transfection, Proliferation Assay, Cell Migration and Invasion Assay, Western blotting, Luciferase assay, Immunofluorescent staining) | same | Not reported |
| (Weedon-Fekjær et al., 2014) | qRT-PCR, High-throughput sequencing | qRT-PCR, miRNA target prediction |  | U18 |
| (Winger et al., 2014) | qRT-PCR, MicroArray analysis | qRT-PCR |  | U48 |
| (Xu et al., 2014) | mammalian miRNA chip array | yes (SYBR and TaqMan qRT-PCR, In Situ Hybridization for miRNAs, Cell Culture and Transient Transfection, Transwell Invasion Assay, Western Blotting, Luciferase Assays) |  | U6 |
|  | TaqMan miRNA micrarray | Not reported |  | U6 |
| (Zhao et al., 2014) | human miRNA microarray kit | SYBR green qPCR, miRNA microarray analysis, miRNA-Gene-network and miRNA-Gene-ontology (GO) network analysis, Luciferase assays |  |  |
| (Zou et al., 2014) | qRT-PCR | Western blotting, Luciferase assay, Treatment of HTR-8/SVneo cells with sodium 4-phenylbutyrate |  | U6 |
| (Akehurst et al., 2015) | miRNA microarray | yes (Taqman and Open Array qRT-PCR) |  | Cel-miR-39 |
|  | Open Array | yes (qRT-PCR) |  | Cel-miR-39 |
|  | Open Array | yes (qRT-PCR) |  | Cel-miR-39 |
| (Anton et al., 2015) | qRT-PCR | Wester blotting, Matrigel Invasion Assay, Angiogenic Growth Factor ELISAs, Lactate Dehydrogenase (LDH) Cytotoxicity Assay |  | U6 |
| (Chen et al., 2015) | stem-loop RT-PCR | Transient transfection, Cell viability analysis, Cell cycle and apoptosis analysis, Proliferation analysis |  | Not reported |
| (Ding et al., 2015) | PrimeScript RT Master Mix and SYBR PrimeScript miRNA RT-PCR Kits, respectively (Takara Biotechnology, Dalian, China) | Yes (qRT-PCR) | same | U6 |
| (Hromadnikova et al., 2015a) | Taqman qRT-PCR | microRNA-gene-Disease ontology interactions |  | RNU58A and U54 |
| (Hromadnikova et al., 2015b) | Taqman qRT-PCR | C19MC microRNA target prediction |  | Cel-miR-39 |
| (Hu et al., 2015) | qRT-PCR | Transient transfection, Western blotting,ELISA, qRT-PCR, Luciferase assay |  | U6 |
| (Jiang et al., 2015) | microarray analysis | yes (qRT-PCR, MiRanda and TargetScan, Western blotting, Plasmids, small interfering (si)RNA and transfection, Luciferase assay, Cell survival and proliferation assay, Transwell invasion and migration assays) | same | U6 |
| (Lasabová et al., 2015) | TaqMan MicroRNA Assay | Not reported |  | RNU44 |
| (LI et al., 2015) | RT‑qPCR | Not reported |  | Cel-miR-39-1 |
| (Miura et al., 2015) | TaqMan MicroRNA Assays | yes (absolute quantification qRT-PCR) | same | U6 |
| (Murphy et al., 2015) | Custom miScript miRNA PCR Arrays | yes (qRT-PCR) | same | Cel-miR-39 |
| (Sun et al., 2015) | qRT-PCR | Cell proliferation/MTT assay, Scratch wound assay,Transwell assay, Western blotting, Dual luciferase reporter assay |  | U6 |
| (Winger et al., 2015) | qRT-PCR | MicroRNA associations with immunological assays, Selection of a seven-microRNA panel, MicroRNA cluster association with metabolic pathways, Immunotesting |  | RNU48 |
| (Yang et al., 2015) | SOLiD sequencing platform | Not reported |  | Not reported |
| (Zhang et al., 2015b) | human miRNA chip assay | yes (qRT-PCR) | same | U6 |
| (Hromadnikova et al., 2016) | TaqMan MicroRNA Assay | yes (qRT-PCR) | same | RNU38B, RNU58A |
| (Hu et al., 2016) | qRT-PCR | Placental explants and cell culture, Western blotting, qRT-PCR, Lactate dehydrogenase (LDH), VEGF, PlGF and placental lactogen (hPL) measurement, RNA interferences, Migration assay of human umbilical vein endothelial cells and tube-like structure formation assay, Assessment of VEGF mRNA stability, Verification of the targets of miRs |  | U6 |
| (Munaut et al., 2016) | qRT-PCR | Not reported |  | U6 and cel-miR-39 |
| (Ospina-Prieto et al., 2016) | qRT-PCR | Cell viability and proliferation assay, Cell invasion assay, Nanoparticle tracking analysis, Co-incubation of nontransfected trophoblast and immune cells with isolated Evs |  | RNU48 |
| (Sandrim et al., 2016a) | SYBR Green qRT-PCR | Not reported |  | U6 |
| (Sandrim et al., 2016b) | miRNA microarray | yes (qRT-PCR) | different | Cel-miR-39 |
|  | qRT-PCR | no |  | miR-423 |
| (Vashukova et al., 2016) | miRNA sequencing | Not reported |  | Not reported |
| (Wang et al., 2016a) | in situ hybridization (ISH) | Immunohistochemistry, Endothelial isolation and culture, Pre-miR-203 transfection, Protein expression, Endothelial IL-6, IL-8, and ICAM production, Myeloperoxidase assay for neutrophil-endothelial adhesion, Construction of SOCS-3/ZsGreen1 GFP vector, SOX3-gene transfer, Endothelial surface molecule ICAM and VCAM expression |  | U6 |
| (Wang et al., 2016b) | high-troughput miRNA sequencing | yes (qRT-PCR) | different | U6 |
| (Yang et al., 2016) | qRT-PCR | Western blotting, Immunohistochemistry, ELISA, Luciferase assay |  | U6 and miR-39 |
| (Zhou et al., 2016) | High-troughput miRNA sequencing | yes (qRT-PCR, Migration and invasion assays) | not clear | U6 |
| (Adel et al., 2017) | qRT-PCR | Not reported |  | U6 |
| (Azizi et al., 2017) | qRT-PCR | Not reported |  | U6 |
| (Fang et al., 2017) | qRT-PCR | Transfection |  | U6 |
| (Gan et al., 2017) | SYBR Green qRT-PCR | Not reported |  | U6 |
| (Gao et al., 2017) | miRNA microarray | yes (qRT-PCR, Luciferase reporter gene activity assay, Transfection of miR-300 mimics and inhibitor, Western blotting) | same | U6 |
| (Gunel et al., 2017) | miRNA microarray | yes (stem-loop qRT-PCR) | same | Cel-miR-39 |
| (Guo et al., 2017) | qRT-PCR | HE staining and immunohistochemistry, Cell viability measurements, Apoptosis assay, Luciferase activity assay, Western blotting, Transfection |  | U6 |
| (Han et al., 2017) | SYBR Green qRT-PCR | MTT Detection, Cell apoptosis testing, Western blotting |  | GAPDH |
| (Hromadnikova et al., 2017) | qRT-PCR | Not reported |  | RNU44 and U75 |
| (Hu et al., 2017) | qRT-PCR | Immunohistochemistry, Western Blotting, Real-time H2S production measurement, RNA interferences, Assessment of sFlt-1 mRNA stability, Verification of the targets of miRs |  | U6 |
| (Zhang et al., 2017) | Stem-loop real-time RT-PCR | Target prediction software, Dual-luciferase assays, Proliferation assay, Transwell invasion assay, In vitro angiogenesis assay |  | Not reported |
| (Jairajpuri et al., 2017) | MiRNA microarray analysis | yes (SYBR Green qRT-PCR) | same | U6 |
| (Jiang et al., 2017) | qRT-PCR,In situ hybridization | Transwell migration and matrigel invasion assay, Western blotting, Generation of luciferase reporter assay |  | U6 |
| (Jin et al., 2017) | MiRNA microarray analysis | yes (qRT-PCR, Transfection, *In vitro* migration and invasion assay, Western Blotting, Luciferase assay) | different | U6 and GAPDH |
| (Korkes et al., 2017) | qRT-PCR | Western blotting |  | Not reported |
| (Li et al., 2017a) | qRT-PCR | MTT sssay, Apoptosis assay, Caspase-3 assay, Luciferase reporter assay, Western blotting |  | U6 |
| (Li et al., 2017b) | qRT-PCR | Cell proliferation, cell cycle and apoptosis assays, Western blotting, HTR-8/Svneo invasion assay, MSC and HTR-8/Svneo migration assay, β-galactosidase activity assay, Dual-luciferase assays |  | U6 |
| (Lu et al., 2017) | qRT-PCR | Immunohistochemistry, Luciferase Reporter Gene Assay, Cell Transfection, Scratch and Transwell Assays, Western Blotting |  | β actin and U6 |
| (Luo et al., 2017a) | microarray analysis | Functional enrichment analyses of the DEMIs and DEGs, Integration of the protein-protein interaction (PPI) network |  | Not reported |
| (Luo et al., 2017b) | qRT-PCR | Cell culture and transfection, Apoptosis assay, qRT-PCR, Western blotting, Transwell assays, Proliferation assay |  | GAPDH |
| (Meng et al., 2017) | qRT-PCR | Cell culture and transfection, Cell viability assay, Cell survival assay, In vitro transwell insert invasion assay, Western blotting, qRT-PCR, Luciferase assays |  | U6 |
| (Nizyaeva et al., 2017) | *In situ* hybridization | Not reported |  | U6 |
| (Salomon et al., 2017) | spectrophotometer, NGS | Not reported |  | Not reported |
| (Shao et al., 2017) | qRT-PCR | Dual-Luciferase Activity Assay, De Novo E2 Synthesis in JEG-3 Cells, In Situ Hybridization |  | U6 |
| (Singh et al., 2017) | Affymetrix Expression Console (TAC) | yes (qRT-PCR) | different | U6-2 |
| (Truong et al., 2017) | next generation sequencing (NGS) | Nanoparticle Tracking Analysis (NTA), Fluorescence NTA, Western blotting, Endothelial cell migration |  | Not reported |
| (Tsai et al., 2017) | qRT-PCR, TaqMan MicroRNA Assay | Not reported |  | U6 |
| (Wang et al., 2017) | qRT-PCR | Transfection, Western blotting, Cell migration, MTT assay, Cell invasion assay |  | U6 |
| (Wei et al., 2017) | Illumina HiSeq 2500 System Rapid with 1 × 100 base single end sequenci–g – RNA deep sequencing | yes (qRT-PCR) | same | Not reported |
| (Xiao et al., 2017) | qRT-PCR | Cell transfection, Knockdown and overexpression of PTEN in HTR8/Svneo cells, Western blotting, Cell proliferation, EdU staining, Cell apoptosis,Wound healing and transwell assays, Dual luciferase reporter assay |  | U6 |
| (Xu and Zhang, 2017) | qRT-PCR | Western blotting, ELISA, Transwell assa, MTT assay |  | U6 |
| (Yang et al., 2017) | qRT-PCR | Not reported |  | GAPDH |
| (Brkić et al., 2018) | qRT-PCR | Not reported |  | U6 |
|  | qRT-PCR | Not reported |  | U6 |
| (Chi and Zhang, 2018) | qRT-PCR | MiR‐145 target gene predictions, Western blotting, MTT assay for HTR‐8/Svneo cell proliferation |  | U6 |
| (Dai and Cai, 2018) | in situ hybridization then qRT-PCR in situ PE vs Controls, but qRT-PCR no comparison between groups | Transfection, In situ hybridization assay, Western blotting, Transwell assay |  | U6 |
| (Fang et al., 2018) | qRT-PCR | Cell Culture and Transfection Experiments, Western Blot Assay, Wound Healing Test, Transwell Assay |  | U6 |
| (Gao et al., 2018a) | qRT-PCR | Cell Culture and Transfection, CCK8 Assay for Cell Proliferation, Cell Cycle Assay, Apoptosis Assay, Western blotting |  | GAPDH |
| (Gao et al., 2018b) | qRT-PCR | MiRNAs, small interference RNAs (siRNAs), plasmids and transfection, Transwell invasion assay, Wound healing assay, Western blotting |  | U6 |
| (Gunel et al., 2020) | Not reported | yes (qRT-PCR) | Not reported | Cel-miR-39 |
| (Guo et al., 2018) | qRT-PCR | Western blotting, CCK-8 assay, Cell apoptotic rate detection, Transwell migration and invasion assays |  | U6 |
| (Khaliq et al., 2018) | qRT-PCR | Western blot analysis |  | U6 and cel-miR-39 |
| (Kim et al., 2018) | qRT-PCR | Not reported |  | SNORD-95 |
| (Li et al., 2018) | qRT-PCR | Western blotting |  | U6 |
| (Liu et al., 2018) | qRT-PCR | Hematoxylin and eosin (H&E) staining, Immunohistochemistry, Cell culture and transfection, Cell proliferation, Western blotting |  | U6 |
| (Lou et al., 2018) | qRT-PCR | Transfection, Cell Counting Kit-8 (CCK-8) Assay, Transwell Assay, Western Blotting |  | U6 |
| (Lykoudi et al., 2018) | miRNA microarray | yes (qRT-PCR) | same | RNU44 |
| (Martinez-Fierro et al., 2018) | TaqMan Human MicroRNA Array | yes (qRT-PCR) | same | Not reported |
| (Motawi et al., 2018) | qRT-PCR | Not reported |  | U6 |
| (Niu et al., 2018) | qRT-PCR | MicroRNA prediction of target genes and functional and bioinformatics analysis, Forced and repressed expression of miR-30a-3p, Western blotting, Apoptosis assay, Transwell invasion assay, Cell cycle detection |  | U6 |
| (Nizyaeva et al., 2018) | In situ hybridization | Not reported |  | Not reported |
| (Shen et al., 2018) | qRT-PCR | Adenovirus and plasmid construction, Western blotting, NO detection |  | U6 |
| (Timofeeva et al., 2018) | miRNA sequencing | yes (qRT-PCR) | same | SNORD68, miR-532-5p, cel-miR-39 |
|  | qRT-PCR | Not reported |  | SNORD68, miR-532-5p, cel-miR-39 |
| (Wang and Yan, 2018) | qRT-PCR | Cell transfection, Caspase-3 activity assay, Transwell invasion assay, Western blot analysis |  | U6 |
| (Wang et al., 2018a) | qRT-PCR | Immunohistochemistry, In-situ hybridization, Sequences and constructs, Cell culture and transfection, Cellular ATP measurement, Mitochondrial membrane potential, Cell apoptosis assay |  | U6 |
| (Wang et al., 2018b) | qRT-PCR | Cell transfection, Proliferation assay,Western blot analysis,Scratch assay,Transwell cell invasion assay |  | U6 |
| (Wang et al., 2018c) | miRNA microarray | yes (qRT-PCR, Transfection of cells with miRNA mimics, ELISA, Western blotting) | same | TNU44 |
| (Winger et al., 2018) | miRNA microarray | yes (qRT-PCR) | different | Not reported |
| (Zou et al., 2018) | qRT-PCR | Cell Proliferation Assay, Trophoblast Cells Infiltration Experiment, Western Blot Assay |  | U6 |
| (Awamleh et al., 2019) | Illumina Truseq RNA and smRNA library preparation kits | TaqMan fast advanced PCR master mix in conjunction with TaqMan miRNA expression assays, Target prediction and gene ontology |  | miR-191-5p |
| (Biró et al., 2019) | Universal RT miRNA PCR Assay | yes (qRT-PCR) | same | miR-103a |
| (Chen et al., 2019) | qRT-PCR | Not reported |  | U6 |
| (Devor et al., 2020) | miRNA microarray | yes (qRT-PCR) | same | RNU48 |
| (Dong et al., 2019) | qRT-PCR | Not reported |  | U6 |
| (Eghbal-Fard et al., 2019) | qRT-PCR | Not reported |  | U6 |
| (Hocaoglu et al., 2019) | qRT-PCR | Not reported |  | U6 |
| (Hromadnikova et al., 2019b) | qRT-PCR | Not reported |  | U58A and U38B |
| (Hromadnikova et al., 2019a) | miRNA microarray | yes (qRT-PCR) | same | Cel-miR-39 |
| (Hu et al., 2019) | miRNA microarray | yes (qRT-PCR) | same | U6 |
| (Huang et al., 2019) | qRT-PCR | Cell Culture and Treatment, Transfection, Cell Viability Assay, Cell Cycle Analysis, Apoptosis Detection Assay, Gelatin Zymography, Western Blotting Analysis |  | U6 |
| (Li et al., 2019) | qRT-PCR | Cell culture and treatments, Cell transfection, An RNA pull-down assay, Microarray analyses, CCK-8 cell proliferation assay, Transwell cell invasion assay, Scratch assay, Immunohistochemistry, Gelatin zymography assay |  | U6 |
| (Liu et al., 2019a) | qRT-PCR | Not reported |  | U6 |
| (Liu et al., 2019b) | qRT-PCR | Immuno-Histochemical Detection, Western Blotting, Cell Invasion Assay, Overexpression of MiR-34a and Transfection of ICN Expression Plasmids |  | U6 |
| (Liu et al., 2019c) | qRT-PCR | Microarray‐based analysis, ELISA, Immunohistochemistry, Cell treatment,Western blot analysis, Immunofuorescence assay, Scratch test, Transwell assay |  | U6 |
| (Ma et al., 2019) | qRT-PCR | Not reported |  | U6 |
| (Martinez-Fierro et al., 2019) | qRT-PCR | Not reported |  | Not reported |
| (Mei et al., 2019) | qRT-PCR | Transfection, Cell invasion assay, Immunofluorescence assay |  | U6 |
| (Nejad et al., 2019) | qRT-PCR | Not reported |  | miR-103 |
| (Pillay et al., 2019) | Nanostring ncounter system miRNA assay | Nanoparticle tracking analysis, Western blotting |  | miRNA controls (RPLP0, RPL19, B2M, GAPDH, andACTB) and spike-in miRNA (osa-miR422, osa-miR414,osa-miR254, osa-miR248 and osa-miR159a) included in the assay, which was finalormalizatsed to total miRNA count |
| (Sekar et al., 2019) | qRT-PCR | Not reported |  | Not reported |
| (Shi et al., 2019) | qRT-PCR | Cell culture and cell treatments, Cell proliferation assay, Cell apoptosis assay, Cell invasion assay, Western blotting |  | U6 |
| (Tang et al., 2019) | qRT-PCR | miR-424 target predictions |  | U6 |
| (Wang et al., 2019a) | qRT-PCR | MicroRNAs prediction, Transfection experiments |  | U6 |
| (Wang et al., 2019b) | qRT-PCR | Western blot analysis, Cell transfection, Transwell cell invasion assay, Scratch assay, RNA pull‐down assay, Immunohistochemical staining |  | U6 |
| (Wang et al., 2019e) | qRT-PCR | Cells culture and transfection, Cell Counting Kit‐8 assay, Enzyme‐linked immunosorbent assay, Western blot analysis, Immunoflorescence |  | miR-16a |
| (Wang et al., 2019c) | Immunohistochemistry (ICH) | yes (qRT-PCR, *In situ* hybridization, Western blotting, Cell culture and in vitro transfection, Cell migration and invasion assay, Network formation assay) | same | U6 |
| (Wang et al., 2019d) | qRT-PCR | Immunohistochemical analysis, Western blotting, Cell transfection, Predictions of miRNA targets |  | U6 |
| (Xiaobo (赵肖波) et al., 2019) | qRT-PCR | miRNA target predictions of target genes, Transfection experiments,Immunohistochemistry, Sequences and constructs, Western blotting, Transwell invasion assay |  | U6 |
| (Xie et al., 2019) | qRT-PCR | Biochemical testing, Transfection, Target gene prediction, MTT assa, Transweormalizion assay, Western blotting |  | U6 |
| (Xue et al., 2019) | miRNA microarray | yes (qRT-PCR, Cell transfection, Invasion and migration assay,Immunofluorescence, Western blotting) | same | U6 |
| (Yang et al., 2019a) | qRT-PCR | Not reported |  | U6 |
| (Yang and Guo, 2019) | qRT-PCR | Cell transfection, Knockdown of PDGFRA, Western blotting, Cell cicle assay,Cell apoptosis,Wound healing and transwell assays, Transwell invasion and migration assay, miRNA target prediction |  | U6 |
| (Yang and Meng, 2019) | qRT-PCR | CCK-8 assay, transwell migration and invasion assay, cell cycle distribution assay and apoptosis assay in HTR-8/Svneo cells transfected with miR-431 mimic, siR-ZEB1 |  | U6 |
| (Yang et al., 2019b) | qRT-PCR | Cell transfection, CCK-8 assay, Transwell invasion assay, Wound healing assay, Cell apoptosis and cell cycle analysis, Western blotting |  | U6 |
| (Yang et al., 2019c) | qRT-PCR | Plasmids, miRNAs, small interfering RNAs, and transfections, Western blot assay, Cell Counting Kit‐8 assay, Colony formation assay, Transwell invasion and migration assay, RNA immunoprecipitation assay |  | U6 |
| (Youssef and Marei, 2019) | qRT-PCR | Not reported |  | U6 |
| (Zhong et al., 2019) | miRNA microarray | yes (qRT-PCR, Function analysis of miRNAs) |  | U6 |
| Ayoub, 2019 | RT-PCR | Not reported |  | MiRNA *SNORD68* |
| Cao, 2019 | qRT-PCR (Tiangen, Beijing, China detection kit) | Not reported |  | U6 |
| Demirer, 2019 | RT-PCR (miScript Primer Assa–s – Qiagen, Valencia, CA, USA) | Not reported |  | U6 |
| Dong, 2019 | qRT-PCR (TB Green^TM^ Premix Ex Taq^TM^ K–t – Takara, Japan) | Not reported |  | U6 |
| Lip, 2019 | MicroRNA array | qRT-PCR (StepOnePlus™ Real-Time PCR System machi–e – Applied Biosystems) | same | hsa-miR-191-5p |
| Lv, 2019 | qRT-PCR with SYBR Green (Life Tech) | Not reported |  | U6 |
| Qian, 2019 | qRT-PCR with SYBR Green | Not reported |  | U6 |
| Xu, 2019 | In situ hybridizatioormaliURY LNA hybridizatioeenptimization Kit (FFPE) from Exiqon) | Not reported |  | NA |
| Yang, 2019 | qRT-PCR | Not reported |  | U6 |
| Yuan, 2019 | qRT-PCR reaction in a ABI 7500  RT-qPCR instrument using SYBR premix Ex Taq^TM^ II PCR Kit  (TaKaRa, Dalian, Liaoning, China) | Not reported |  | U6 |
| Zhang, 2019 | qRT-PCR | Not reported |  | GAPDH |
| Akgor, 2020 | qRT-PCR high-throughput BioMark Real-Time PCR system | Not reported |  | global meormalizationion method |
| Devor, 2020 | A-set TaqMan Low Density microRNA Arrays (TaqMan low density array (TLDA), Life Technologies) | Not reported |  | RNU48 |
| Dong, 2020 | miRNA‑specific TaqMan miRNA assay kit (Applied Biosystems; Thermo Fisher Scientific, Inc.). | qRT-PCR (FastStart Univepreeclampsial SYBR Green Master (Applied Biosystems; Thermo Fisher Scientific, Inc.)) | same | U6 |
| Fan, 2020 | qRT-PCR (SYBR Green plus kit (Roche, Basel, Switzerland)) | Not reported |  | U6 |
| Gong, 2020 | qRT-PCR | Not reported |  | U6 |
| Han, 2020 | qRT-PCR | Not reported |  | Not reported |
| Huang, 2020 | qRT-PCR (YBR-Green Master mix (Life Technologies, Carlsbad, CA, USA)) | Not reported |  | U6 |
| Jelena, 2020 | Taq-Man miRNA Reverse Transcription  Kit (Cat. Number: 4366596) and specific TaqMan MicroRNA Assays | ddPCR | same | / |
| Kim, 2020 | qRT-PCR was performed with a miScript SYBR Green PCR kit (#218073; Qiagen, Hilden, Germany) | Not reported |  | SNORD-95  miR-1290-39 |
| Li W, 2020 | SYBR Green Realtime PCR Master Mix (SinoBio, Shanghai, China) on a PCR Amplifier (Suzhou Molarray Co., Ltd., China) | Not reported |  | U6 |
| Li T, 2020 | qRT-PCR | Not reported |  | U6 |
| Li Q, 2020 | TB Green® Premix Ex Taq™ II kit (Takara, Dalian, China) with an Applied Biosystems 7500Fast (PerkinElmer,  Foster City, CA) | Not reported |  | U6 |
| Li H, 2020 | qRT-PCR | Not reported |  | U6 |
| Licini, 2020 | Total RNA Purification Kit (Norgen Biotek Corp., Thorold, Canada) | TaqMan Fast Universal PCR Master Mix and probes for miR-125b, RNU48, and cel-miR-39 (all from Applied Biosystem, Life Technologies) | same | RNU-48 |
| Ma, 2020 | qRT-PCR | Not reported |  | U6 |
| Mavreli, 2020 | Illumina NGS | qRT-PCR | different | U6 |
| Sheng, 2020 | qRT-PCR | Not reported |  | U6 |
| Song 2020 | qRT-PCR | Not reported |  | U6 |
| Tao, 2020 | qRT-PCR | Not reported |  | Not reported |
| Wang, 2020 | qRT-PCR | Not reported |  | U6 |
| Whigham, 2020 | Microarray (miScript SYBR green PCR kit (Qiagen)) | qRT-PCR |  | miR191  SNORD44  SNORD48 |
| Wu D, 2020 | qRT-PCR | Not reported |  | U6 |
| Wu G, 2020 | qRT-PCR | Not reported |  | U6 |
| Xueya, 2020 | qRT-PCR | Not reported |  | U6 |
| Yang, 2020 | qRT-PCR | Not reported |  | U6 |
| Zhao, 2020 | qRT-PC | Not reported |  | U6 |
| Zheng, 2020 | qRT-PCR | Not reported |  | U6 |
| Zhou, 2020 | qRT-PCR (miRcute miRNA qPCR Detection kit (SYBR Green; Tiangen Biotech Co., Ltd.)) | Not reported |  | U6 |
| Zhu, 2020 | qRT-PCR | Not reported |  | U6 |
| Ali, 2021 | qRT-PCR | Not reported |  | Three housekeeping genes were used for internal control |
| Brodowski, 2021 | small RNA sequencing (NEBNext® Multiplex Small RNA Library Prep  Kit for Illumina® 96 rxns, Index Primers 1–48 (E7560S, New England Biolabs, Frankfurt am Main, Germany).) | qRT-PCR (miScript II Master Mix (miScript II, SYBR Green Assay, Qiagen, Leipzig, Germany, 218160) on a Corbett Rotor Gene (Corbett Life Science, Sydney, Australia).) |  | 18S rRNA (RNA78S1) |
| Cai, 2021 | qRT-PCR | Not reported |  | U6 |
| Chu, 2021 | In situ hybridization | Not reported |  | / |
| Hayder, 2021 | qRT-PCR | Not reported |  | U6 |
| Jairajpuri, 2021 | qRT-PCR | Not reported |  | U6 |
| Jiu, 2021 | qRT-PCR | Not reported |  | U6 |
| Kamali-Simsek, 2021 | Droplet Digital PCR (ddPCR) was performed using QX200 ddPCR EvaGreen SuperMix (Bio-Rad, Hercules, CA, USA) in a QX200 Droplet Digital PCR System (Bio-Rad). | Not reported |  | U6 |
| Kolkova, 2021 | qRT-PCR | Not reported |  | Not reported |
| Liao, 2021 | qRT-PCR (SYBR_ Premix Ex TaqTM II (Takara) on an ABI 7900HT fast PCR real-time system (Applied Biosystems, Foster City, CA, USA)) | Not reported |  | U6 |
| Luizon, 2021 | The QIAGEN’s GeneGlobe Data Analysis Center (https://  geneglobe.qiagen.com/us/analyze/) online platform | Not reported |  | Not reported |
| Mao, 2021 | qRT-PCR (TaqMan® MicroRNA real-time PCR Assay reagents was carried out (Applied Biosystems; Foster City, CA, USA)) | Not reported |  | U6 |
| Martinez-Fierro, 2021 | TaqMan human microRNA array set v2.0 (Applied Biosystems, Foster City, CA, USA) | Not reported |  | Not reported |
| Peng, 2021 | qRT-PC | Not reported |  | U6 |
| Witvrouwen, 2021 | qRT-PCR (TaqMan miRNA primers (Thermo Fisher) and multiplex qPCR was done in a CFX96 thermal cycler (Bio-Rad)) | Not reported |  | spike-in Cel-miR-39 |
| Xu, 2021 | qRT-PC (SYBR-miR-39cycler (Bi  II (Perfect Real Time) kit (DRR081) on the ABI7500 quantitative PCR instrument  (ABI)) | Not reported |  | U6 |
| Yu, 2021 | qRT-PCR | Not reported |  | U6 |
| Zhao, 2021 | qRT-PCR | Not reported |  | U6 |
| Zhao, 2021 | qRT-PCR | Not reported |  | U6 |
| Zhu, 2021 | qRT-PCR | Not reported |  | U6 |
| Zolfaghari, 2021 | qRT-PCR | Not reported |  | Not reported |

Table S5. Major findings for more severe PE

| **miRNK** | **tissue** | **Up/Down** | **Sig.** | **Study** | **Ref.** |
| --- | --- | --- | --- | --- | --- |
| 182 | Placenta | Up in sPE | yes (p<0.10) | (Pineles et al., 2007) | 1 |
| 210 | Placenta  Placenta (villi)  Placenta (villi)  Placenta (eciduas-MSCs)  MPB (plasma)  Placenta (chorionic plate)  Placenta (basal plate)  MPB  Placenta (chorionic plate)  Placenta (basal plate)  MPB (plasma)  Placenta  Placenta (villi)  MPB (plasma) | Up in sPE  Up in sPE  Up in sPE  Down in sPE  Up in sPE  No difference in sPE  Up in sPE  NR  No difference in sPE  Up in sPE  Up in sPE  Up in sPE  Up in sPE  Up in sPE | yes (p<0.10)  yes  yes  no  yes  no  yes  NR  no  yes  yes  yes  yes  yes | (Pineles et al., 2007)  (Zhong et al., 2019)  (Muralimanoharan et al., 2012)  (Wang et al., 2012b)  (Zhang et al., 2012)  (Luo et al., 2014)  (Luo et al., 2014)  (Winger et al., 2015)  (Xu et al., 2014)  (Xu et al., 2014)  (Xu et al., 2014)  (Vashukova et al., 2016)  (Adel et al., 2017)  (Jairajpuri et al., 2021) | 1  3  17  19  21  39  39  44  45  45  45  74  82  Jairajpuri 2021 |
| 210-3p | MPB (plasma)  MPB (plasma)  Placenta (villi)  MPB (plasma)  Placenta | Up in sPE at deliverz  No difference in sPE 1 year after delivery  No difference in EOPE  No difference in EOPE  No difference in pre-term PE | Yes  No  No  No  No | (Murphy et al., 2015)  (Murphy et al., 2015)  (Awamleh et al., 2019)  (Jelena et al., 2020)  (Hayder et al., 2021) | 60  60  150  Jelena 2020  Hayder 2021 |
| 181a | Placenta (chorion)  UC-MSCs  Placenta(eciduas)  MPB (plasma)  MPB  Placenta (syncytiotrophoblast) | Up in sPE  Up in sLOPE  Up in sLOPE  Up in sPE  NR  Down in EOPE | yes  yes  yes  yes  NR  Yes | (Hu et al., 2009)  (Liu et al., 2012)  (Liu et al., 2012)  (Wu et al., 2012)  (Winger et al., 2015)  (Nizyaeva et al., 2018) | 2  16  16  20  44  139 |
| 181a-1,2 | Placenta (chorionic plate)  Placenta (basal plate) | No difference in sPE  No difference in sPE | No  No | (Xu et al., 2014)  (Xu et al., 2014) | 45  45 |
| 181a-5p | MPB (plasma)  MPB (plasma) | Up in sPE at delivery  No difference in sPE 1 year after | Yes  No | (Murphy et al., 2015)  (Murphy et al., 2015) | 60  60 |
| 195 | Placenta (chorion)  Placenta  MPB  Placenta (chorionic plate)  Placenta (basal plate)  Placenta  UC blood  MPB (plasma)  Placenta  MPB (plasma)  MPB (plasma) | Up in sPE  Down in sPE  NR  Down in sPE  No difference in sPE  Down in sPE  Down in sPE  No difference in sPE  Down in sPE  Down in sPE (15-18gw)  Down in sPE (35-39gw) | Yes  Yes  NR  Yes  No  Yes  Yes  NR  Yes  Yes  Yes | (Hu et al., 2009)  (Bai et al., 2012)  (Winger et al., 2015)  (Xu et al., 2014)  (Xu et al., 2014)  (Vashukova et al., 2016)  (Hromadnikova et al., 2017)  (Gunel et al., 2020)  (Gunel et al., 2020)  (Wang et al., 2018a)  (Wang et al., 2018a) | 2  12  44  45  45  74  92  127  127  143  143 |
| 195-5p | MPB (plasma)  MPB (whole blood) | No difference in sPE at delivery and 1 year after  No difference in sPE | No  No | (Murphy et al., 2015)  (Hromadnikova et al., 2016) | 60  66 |
| 222 | Placenta (chorion) | Up in sPE | yes | (Hu et al., 2009) | 2 |
| 222-3p | MPB (plasma) | Up in sPE at delivery  No difference 1 year after delivery | No  no | (Murphy et al., 2015)  (Murphy et al., 2015) | 60  60 |
| 16 | Placenta (chorion)  Placenta (deciduas-MSCs)  MPB  Placenta (deciduas MSCs)  MPB (plasma)  Placenta  MPB (whole blood)  MPB (serum)  MPB (plasma) | Up in sPE  Up in sPE  NR  Up in sPE  No difference in EOPE  Up in sPE  Down in sEOPE  Up in EOPE  Up in EOPE | Yes  Yes  NR  Yes  No  Yes  Yes  Yes  Yes | (Hu et al., 2009)  (Wang et al., 2012b)  (Winger et al., 2015)  (Zhao et al., 2014)  (Dong et al., 2019)  (Li et al., 2020)  (Whigham et al., 2020)  (Ali et al., 2021)  (Witvrouwen et al., 2021) | 2  19  44  46  154  Li T, 2020  Whigham 2020  Ali 2021  Witvrouwen 2021 |
| 16-5p | MPB (plasma) | No difference in sPE at delivery and 1 year after | no | (Murphy et al., 2015) | 60 |
| 29b | Placenta (chorion)  Placenta (eciduas-MSCs)  Placenta (chorion)  Placenta (eciduas MSCs)  MPB (plasma) | Up in sPE  Up in sPE  Up in sLOPE  Up in sPE  No difference in EOPE | yes  yes  yes  yes  No | (Hu et al., 2009)  (Wang et al., 2012b)  (Li et al., 2013b)  (Zhao et al., 2014)  (Witvrouwen et al., 2021) | 2  19  30  46  Witvrouwen 2021 |
| 29b-3p | MPB (plasma) | Up in sPE at delivery  No difference in sPE 1 year after | Yes  no | (Murphy et al., 2015)  (Murphy et al., 2015) | 60  60 |
| 26b | Placenta (chorion) | Up in sPE | yes | (Hu et al., 2009) | 2 |
| 26b-5p | MPB (plasma) | Up in sPE | No | (Luizon et al., 2021) | Luizon 2021 |
| 335 | Placenta (chorion) | Up in sPE | yes | (Hu et al., 2009) | 2 |
| 152 | Placenta (villi)  Placenta (eciduas-MSCs) | Up in sPE  Up in sPE | yes  yes | (Zhong et al., 2019)  (Wang et al., 2012b) | 3  19 |
| 411 | Placenta (villi)  Placenta (chorionic plate)  Placenta (basal plate) | Down in sPE  Down in sPE  Down in sPE | Yes  Yes  Yes | (Zhong et al., 2019)  (Xu et al., 2014)  (Xu et al., 2014) | 3  45  45 |
| 377 | Placenta (villi)  Placenta | Down in sPE  No difference in preterm PE | Yes  No | (Zhong et al., 2019)  (Yang et al., 2019a) | 3  186 |
| 519b | Placenta (villi) | Up in sPE | yes | (Zhong et al., 2019) | 3 |
| 18a | Placenta (villi)  Placenta (chorionic plate)  Placenta (basal plate)  MPB (plasma)  MPB (plasma)  Placenta  MPB (whole blood) | Down in sPE  Down in sPE  No difference in sPE  Down in sPE  Up in sPE  Up in sPE  Down in sEOPE | Yes  Yes  No  Yes  NR  NR  Yes | (Zhong et al., 2019)  (Xu et al., 2014)  (Xu et al., 2014)  (Xu et al., 2014)  (Yang et al., 2015)  (Yang et al., 2015)  (Whigham et al., 2020) | 3  45  45  45  64  64  Whigham 2020 |
| 18a-5p | MPB (plasma)  MPB (plasma)  MPB (plasma) | No difference in sPE at delivery  No difference in sPE 1 year after delivery  Up in sPE | No  No  No | (Murphy et al., 2015)  (Murphy et al., 2015)  (Luizon et al., 2021) | 60  60  Luizon 2021 |
| 363 | Placenta (villi)  Placenta (chorionic plate)  Placenta (basal plate)  Placenta (basal plate)  Placenta (chorionic plate)  MPB (whole blood) | Down in sPE  No difference in sPE  No difference in sPE  Down in sPE  Down in sPE  Down in sEOPE | Yes  No  No  Yes  No  Yes | (Zhong et al., 2019)  (Xu et al., 2014)  (Xu et al., 2014)  (Zhang et al., 2015a)  (Zhang et al., 2015a)  (Whigham et al., 2020) | 3  45  45  65  65  Whigham 2020 |
| 542-3p | Placenta (villi)  Placenta (chorionic plate)  Placenta (basal plate) | Down in sPE  No difference in sPE  No difference in sPE | Yes  No  No | (Zhong et al., 2019)  (Xu et al., 2014)  (Xu et al., 2014) | 3  45  45 |
| 155 | Placenta (chorion)  UC hUVECs  Placenta  MPB  Placenta (chorion)  Placenta (villi – syncytiotrophoblast)  MPB (plasma) | Up in sPE  Down in sPE  Up in sPE  NR  Up in sPE  Up in EOPE  No difference in EOPE | yes  NR  yes  NR  Yes  Yes  No | (Zhang et al., 2010)  (Cheng et al., 2011)  (Li et al., 2014b)  (Winger et al., 2015)  (Azizi et al., 2017)  (Nizyaeva et al., 2017)  (Witvrouwen et al., 2021) | 4  5  38  44  83  106  Witvrouwen 2021 |
| 155-5p | MPB (plasma)  MPB (plasma)  MPB (leukocytes) | Up in sPE at delivery  No difference in sPE 1 year after delivery  Not reported | Yes  No  NR | (Murphy et al., 2015)  (Murphy et al., 2015)  (Demirer et al., 2020) | 60  60  Demirer 2019 |
| 24 | Placenta  MPB (plasma) | NR  Up in sPE | NR  yes | (Xu et al., 2019)  (Huang et al., 2020) | 9  20 |
| 24-3p | MPB (plasma) | Up in sPE | No | (Luizon et al., 2021) | Luizon 2021 |
| 517-5p | Placenta | Down in sPE and EOPE | yes | (Hromadnikova et al., 2015b) | 53 |
| 517a | Placenta  Placenta  MPB (serum) | NR  Up in preterm PE  Up in sPE | NR  Yes  Yes | (Xu et al., 2019)  (Anton et al., 2015)  (Martinez-Fierro et al., 2018) | 9  49  136 |
| 517b | Placenta | Up in preterm PE | Yes | (Anton et al., 2015) | 49 |
| 519d | Placenta  MPB (plasma)  Placenta | NR  Up in sPE  Up in sPE | NR  NR  NR | (Xu et al., 2019)  (Yang et al., 2015)  (Yang et al., 2015) | 9  64  64 |
| 519-3p | Placenta  Placenta  MPB (plasma) | Up in sPE  Down in EOPE  Up in EOPE | Yes  Yes  Yes | (Ding et al., 2015)  (Timofeeva et al., 2018)  (Timofeeva et al., 2018) | 51  141  141 |
| 519a | Placenta  Placenta  MPB (plasma)  MPB (plasma)  MPB (plasma)  Placenta (syncytiotrophoblast) | NR  Down in sPE  Up in sPE  Up in sEOPE  No difference in sLOPE  Up in EOPE | NR  Yes  Yes  Yes  No  Yes | (Xu et al., 2019)  (Hromadnikova et al., 2015b)  (Miura et al., 2015)  (Miura et al., 2015)  (Miura et al., 2015)  (Nizyaeva et al., 2018) | 9  53  59  59  59  139 |
| 23a | Placenta | NR | NR | (Xu et al., 2019) | 9 |
| 103 | Placenta  MPB (plasma) | NR  Up in sPE | NR  yes | (Xu et al., 2019)  (Wu et al., 2012) | 9  20 |
| 103a-3p | MPB (whole blood)  MPB (plasma) | No difference in sPE  Up in sPE | No  No | (Hromadnikova et al., 2016)  (Luizon et al., 2021) | 66  Luizon 2021 |
| 29a | Placenta  MPB (plasma)  MPB (plasma)  Placenta | NR  No difference in sPE  Up in sPE  Up in sPE | NR  No  NR  NR | (Xu et al., 2019)  (Li et al., 2013a)  (Yang et al., 2015)  (Yang et al., 2015) | 9  29  64  64 |
| 29a-3p | MPB (plasma)  MPB  MPB (plasma) | No difference in sPE at delivery and 1 year after  Up in sPE  Up in sPE | No  Yes  No | (Murphy et al., 2015)  (Hromadnikova et al., 2019b)  (Luizon et al., 2021) | 60  158  Luizon 2021 |
| 517c | Placenta  Placenta  MPB (plasma  Placenta) | NR  Up in preterm PE  Up in sPE  Up in sPE | NR  Yes  NR  NR | (Xu et al., 2019)  (Anton et al., 2015)  (Yang et al., 2015)  (Yang et al., 2015) | 9  49  64  64 |
| 130a | Placenta  MPB (plasma)  Placenta | NR  Up in sPE  Up in sPE | NR  NR  NR | (Xu et al., 2019)  (Yang et al., 2015)  (Yang et al., 2015) | 9  64  64 |
| 130a-3p | MPB (plasma) | No difference in sPE at delivery and 1 year after | no | (Murphy et al., 2015) | 60 |
| 29c | Placenta | NR | NR | (Xu et al., 2019) | 9 |
| 223 | MPB (serum)  MPB  Placenta (chorionic plate)  Placenta (basal plate)  Placenta  Placenta | Up in sPE  NR  Down in sPE  Down in sPE  Down in sPE  Down in EOPE | Yes  NR  Yes  Yes  Yes  Yes | (Yang et al., 2011)  (Winger et al., 2015)  (Xu et al., 2014)  (Xu et al., 2014)  (Vashukova et al., 2016)  (Meng et al., 2017) | 11  44  45  45  74  105 |
| let-7d | MPB (serum)  Placenta (trophoblast cells) | Up in sPE  Up in sPE | Yes  Yes | (Yang et al., 2011)  (Dai and Cai, 2018) | 11  122 |
| let-7f-5p | MPB (plasma) | No difference in sPE at delivery and 1 year after | no | (Murphy et al., 2015) | 60 |
| let-7f | MPB (serum)  Placenta | Up in sPE  Down in sPE | Yes  Yes | (Yang et al., 2011)  (Vashukova et al., 2016) | 11  74 |
| 34c | Placenta (villi)  Placenta  Placenta | no difference  Up in sPE  Down in sPE | no  no  Yes | (Muralimanoharan et al., 2012)  (Wang et al., 2012a)  (Vashukova et al., 2016) | 17  18  74 |
| 129 | Placenta (villi) | no difference | no | (Muralimanoharan et al., 2012) | 17 |
| 328 | Placenta (villi) | no difference | no | (Muralimanoharan et al., 2012) | 17 |
| 17 | Placenta  Placenta (chorionic plate)  Placenta (basal plate)  MPB (plasma)  Placenta (syncytiotrophoblast)  Endothelium | Up in sPE  Down in sPE  No difference in sPE  No difference in sPE  NR  NR | Yes  No  No  No  NR  NR | (Wang et al., 2012a)  (Xu et al., 2014)  (Xu et al., 2014)  (Xu et al., 2014)  (Nizyaeva et al., 2018)  (Nizyaeva et al., 2018) | 18  45  45  45  139  139 |
| 17-3p | Placenta (chorionic plate)  Placenta (basal plate) | No difference in sPE  Up in sPE | No  Yes | (Xu et al., 2014)  (Xu et al., 2014) | 45  45 |
| 17-5p | MPB (plasma)  MPB (whole blood)  MPB  MPB (plasma) | No difference in sPE at delivery and 1 year after  No difference in sPE  Up in sPE  Up in sPE | No  No  Yes  No | (Murphy et al., 2015)  (Hromadnikova et al., 2016)  (Hromadnikova et al., 2019b)  (Luizon et al., 2021) | 60  66  158  Luizon 2021 |
| 20a | Placenta  Placena (chorionic plate)  Placenta (basal plate) | Up in sPE  No difference in sPE  No difference in sPE | Yes  No  No | (Wang et al., 2012a)  (Xu et al., 2014)  (Xu et al., 2014) | 18  45  45 |
| 20a-5p | MPB (plasma) | No difference in sPE at delivery and 1 year after | no | (Murphy et al., 2015) | 60 |
| 20b | Placenta  Placenta (basal plate)  Placenta (chorionic plate) | Up in sPE  Up in sPE  Up in sPE | Yes  Yes  Yes | (Wang et al., 2012a)  (Zhang et al., 2015a)  (Zhang et al., 2015a) | 18  65  65 |
| 20b-5p | MPB (plasma)  MPB | No difference in sPE at delivery and 1 year after  Up in sPE | No  Yes | (Murphy et al., 2015)  (Hromadnikova et al., 2019b) | 60  158 |
| 151-3p | Placenta | Down in sPE | no | (Wang et al., 2012a) | 18 |
| 151 | Placenta (chorionic plate)  Placenta (basal plate) | No difference in sPE  Up in sPE | No  Yes | (Xu et al., 2014)  (Xu et al., 2014) | 45  45 |
| 524-3p | Placenta | Up in sPE | yes | (Wang et al., 2012a) | 18 |
| 524-5p | Placenta | Down in sPE | yes |  | 53 |
| 524 | Placena (chorionic plate)  Placenta (basal plate)  Placenta | No difference in sPE  Up in sPE  Up in sPE | No  Yes  Yes | (Xu et al., 2014)  (Xu et al., 2014)  (Vashukova et al., 2016) | 45  45  74 |
| 1975 | Placenta | Up in sPE | no | (Wang et al., 2012a) | 18 |
| 30a | Placenta (eciduas MSCs)  Placenta (eciduas MSCs) | Up in sPE  Up in sPE | Yes  Yes | (Wang et al., 2012b)  (Zhao et al., 2014) | 19  46 |
| 30a-3p | Placenta (chorionic plate)  Placenta (basal plate) | No difference in sPE  Up in sPE | No  Yes | (Xu et al., 2014)  (Xu et al., 2014) | 45  45 |
| 26a | MPB (plasma) | Up in sPE | yes | (Wu et al., 2012) | 20 |
| 26a-5p | Placenta  UC blood  UC blood | Down in EOPE  Down in sPE  Down in EOPE | Yes  Yes  Yes | (Hromadnikova et al., 2015a)  (Hromadnikova et al., 2017)  (Hromadnikova et al., 2017) | 52  92  92 |
| 130b | MPB (plasma) | Up in sPE | yes | (Wu et al., 2012) | 20 |
| 342-3p | MPB (plasma) | Up in sPE | yes | (Wu et al., 2012) | 20 |
| 574-5p | MPB (plasma)  MPB (plasma) | Up in sPE  Up in EOPE | Yes  Yes | (Wu et al., 2012)  (Lip et al., 2020) | 20  Lip 2019 |
| 574-3p | MPB (whole blood)  UC blood  UC blood | No difference in sPE  Down in sPE  Down in EOPE | No  Yes  Yes | (Hromadnikova et al., 2016)  (Hromadnikova et al., 2017)  (Hromadnikova et al., 2017) | 66  92  92 |
| 141 | MPB (plasma) | No difference in sPE | no | (Li et al., 2013a) | 29 |
| 144 | MPB (plasma) | Down in sPE | yes | (Li et al., 2013a) | 29 |
| 221 | MPB (plasma)  MPB (serum)  Placenta (eciduas MSCs) | No difference in sPE  No difference in EOPE  Up in sPE | no  no  yes | (Li et al., 2013a)  (Luque et al., 2014)  (Zhao et al., 2014) | 29  40  46 |
| 125b-1-3p | Placenta (basal plate)  Placenta (chorionic plate) | Up in sPE  No difference in sPE | yes  no | (Li et al., 2014a)  (Li et al., 2014a) | 37  37 |
| 125-5p | MPB (whole blood)  MPB (whole blood) | Down in sPE  Down in EOPE | Yes  Yes | (Hromadnikova et al., 2016)  (Hromadnikova et al., 2016) | 66  66 |
| 192 | MPB (serum) | No difference in EOPE | no | (Luque et al., 2014) | 40 |
| 143 | MPB (serum) | No difference in EOPE | no | (Luque et al., 2014) | 40 |
| 143-3p | MPB (plasma) | Up in sPE | No | (Luizon et al., 2021) | Luizon 2021 |
| 125b | MPB (serum) | No difference in EOPE | no | (Luque et al., 2014) | 40 |
| 127 | MPB (serum) | No difference in EOPE | no | (Luque et al., 2014) | 40 |
| 942 | MPB (serum)  MPB (plasma) | No difference in EOPE  Up in sPE | No  Yes | (Luque et al., 2014)  (Singh et al., 2017) | 40  109 |
| 126 | MPB (serum)  MPB (plasma)  Placenta  Placenta  MPB (plasma) | No difference in EOPE  Up in sPE  Up in sPE  Up in sPE  No difference in EOPE | No  NR  NR  Yes  No | (Luque et al., 2014)  (Yang et al., 2015)  (Yang et al., 2015)  (Liu et al., 2021)  (Witvrouwen et al., 2021) | 40  64  64  Liu 2021  Witvrouwen 2021 |
| 126* | MPB (plasma)  Placenta | Up in sPE  Up in sPE | NR  NR | (Yang et al., 2015)  (Yang et al., 2015) | 64  64 |
| 126-3p | MPB (plasma)  MPB  MPB (plasma exosomes) | No difference in sPE at delivery and 1 year after  No difference in sPE  Up in EOPE | No  Yes  Yes | (Murphy et al., 2015)  (Hromadnikova et al., 2019b)  (Pillay et al., 2019) | 60  158  171 |
| 223-3p | Placenta  MPB (plasma exosomes)  MPB (plasma) | Down in EOPE  Up in EOPE  Up in sPE | Yes  Yes  No | (Weedon-Fekjær et al., 2014)  (Pillay et al., 2019)  (Luizon et al., 2021) | 43  171  Luizon 2021 |
| 224 | MPB (plasma)  Placenta | Up in sPE  Up in sPE | NR  NR | (Yang et al., 2015)  (Yang et al., 2015) | 64  64 |
| 224-5p | Placenta | Down in EOPE | yes | (Weedon-Fekjær et al., 2014) | 43 |
| 1301 | Placenta | Down in EOPE | yes | (Weedon-Fekjær et al., 2014) | 43 |
| 340-5p | MPB | NR | NR | (Winger et al., 2015) | 44 |
| 424 | MPB (whole blood) | No difference in sEOPE | No | (Whigham et al., 2020) | Whigham 2020 |
| 424-5p | MPB | NR | NR | (Winger et al., 2015) | 44 |
| 33a-5p | MPB | NR | NR | (Winger et al., 2015) | 44 |
| 7-5p | MPB | NR | NR | (Winger et al., 2015) | 44 |
| 1229 | MPB | NR | NR | (Winger et al., 2015) | 44 |
| 1267 | MPB | NR | NR | (Winger et al., 2015) | 44 |
| 671-3p | MPB | NR | NR | (Winger et al., 2015) | 44 |
| 1 | MPB  Placenta  Placenta | NR  No difference in EOPE  Down in sPE | NR  No  Yes | (Winger et al., 2015)  (Hromadnikova et al., 2015a)  (Vashukova et al., 2016) | 44  52  74 |
| 133b | MPB | NR | NR | (Winger et al., 2015) | 44 |
| 144-3p | MPB | NR | NR | (Winger et al., 2015) | 44 |
| 582-5p | MPB | NR | NR | (Winger et al., 2015) | 44 |
| 30e-3p | MPB | NR | NR | (Winger et al., 2015) | 44 |
| 30e-5p | MPB (plasma) | Up in sPE | No | (Luizon et al., 2021) | Luizon 2021 |
| 199a-5p | MPB  MPB (whole blood)  MPB (whole blood)  UC blood | NR  No difference in sPE  Down in EOPE  Down in sPE | NR  No  Yes  Yes | (Winger et al., 2015)  (Hromadnikova et al., 2016)  (Hromadnikova et al., 2016)  (Hromadnikova et al., 2017) | 44  66  66  92 |
| 199b-5p | MPB | NR | NR | (Winger et al., 2015) | 44 |
| 221-5p | MPB | NR | NR | (Winger et al., 2015) | 44 |
| 221-3p | MPB (whole blood)  MPB (whole blood)  UC blood  MPB (plasma)  MPB (plasma) | No difference in sPE  Down in EOPE  Down in sPE  No difference in sPE at delivery and 1 year after  Up in sPE | No  Yes  Yes  No  No | (Hromadnikova et al., 2016)  (Hromadnikova et al., 2016)  (Hromadnikova et al., 2017)  (Murphy et al., 2015)  (Luizon et al., 2021) | 66  66  92  60  Luizon 2021 |
| 575 | MPB | NR | NR | (Winger et al., 2015) | 44 |
| 301a-3p | MPB  MPB (plasma)  Placenta | NR  Up in sPE  Up in sPE | NR  NR  NR | (Winger et al., 2015)  (Yang et al., 2015)  (Yang et al., 2015) | 44  64  64 |
| 148a-3p | MPB | NR | NR | (Winger et al., 2015) | 44 |
| 193a-3p | MPB | NR | NR | (Winger et al., 2015) | 44 |
| 219-5p | MPB | NR | NR | (Winger et al., 2015) | 44 |
| 132 | MPB | NR | NR | (Winger et al., 2015) | 44 |
| 513a-5p | MPB | NR | NR | (Winger et al., 2015) | 44 |
| 1244 | MPB | NR | NR | (Winger et al., 2015) | 44 |
| 146a | MPB  Placenta (villi – syncytiotrophoblast)  Placenta (villi – syncytial knots) | NR  Down in EOPE  Down in EOPE | NR  No  No | (Winger et al., 2015)  (Nizyaeva et al., 2017)  (Nizyaeva et al., 2017) | 44  106  106 |
| 146-5p | MPB (whole blood)  MPB (whole blood)  MPB (plasma) | No difference in sPE  Down in EOPE  Up in sPE | No  Yes  No | (Hromadnikova et al., 2016)  (Hromadnikova et al., 2016)  (Luizon et al., 2021) | 66  66  Luizon 2021 |
| 196a | MPB | NR | NR | (Winger et al., 2015) | 44 |
| 218 | Placenta (chorionic plate)  Placenta (basal plate) | Down in sPE  Down in sPE | Yes  Yes | (Xu et al., 2014)  (Xu et al., 2014) | 45  45 |
| 218-5p | Placenta | Up in PE | Yes | (Yu et al., 2021) | Yu 2021 |
| 379 | Placenta (chorionic plate)  Placenta (basal plate) | Down in sPE  No difference in sPE | Yes  No | (Xu et al., 2014)  (Xu et al., 2014) | 45  45 |
| 518b | Placenta (chorionic plate)  Placenta (basal plate)  MPB (plasma)  MPB (plasma) | No difference in sPE  Up in sPE  Up in sPE, sEOPE, sLOPE  No difference | No  Yes  Yes  No | (Xu et al., 2014)  (Xu et al., 2014)  (Miura et al., 2015)  (Jelena et al., 2020) | 45  45  59  Jelena 2020 |
| 193b | Placenta (chorionic plate)  Placenta (basal plate)  Placenta | No difference in sPE  Up in sPE  Up in sPE | No  Yes  Yes | (Xu et al., 2014)  (Xu et al., 2014)  (Vashukova et al., 2016) | 45  45  74 |
| 638 | Placenta (chorionic plate)  Placenta (basal plate) | No difference in sPE  No difference in sPE | No  No | (Xu et al., 2014)  (Xu et al., 2014) | 45  45 |
| 515-3p | Placenta (chorionic plate)  Placenta (basal plate)  Placenta | No difference in sPE  No difference in sPE  Up in sPE | No  No  Yes | (Xu et al., 2014)  (Xu et al., 2014)  (Vashukova et al., 2016) | 45  45  74 |
| 515-5p | MPB (plasma)  Placenta | Up in sPE, sEOPE, sLOPE  Up in sPE | Yes  Yes | (Miura et al., 2015)  (Vashukova et al., 2016) | 59  74 |
| 525-3p | Placenta (chorionic plate)  Placenta (basal plate) | No difference in sPE  No difference in sPE | No  No | (Xu et al., 2014)  (Xu et al., 2014) | 45  45 |
| 525-5p | MPB (plasma) | Up in sPE, sEOPE, sLOPE | yes | (Miura et al., 2015) | 59 |
| 525 | Placenta | Down in sPE | yes | (Hromadnikova et al., 2015b) | 53 |
| 19a | Placenta (chorionic plate)  Placenta (basal plate) | No difference in sP  No difference in sPE | No  No | (Xu et al., 2014)  (Xu et al., 2014) | 45  45 |
| 19a-3p | MPB (plasma)  MPB (plasma) | No difference in sPE at delivery and 1 year after  Up in PE | No  No | (Murphy et al., 2015)  (Luizon et al., 2021) | 60  Luizon 2021 |
| 584 | Placenta (chorionic plate)  Placenta (basal plate) | No difference in sPE  No difference in sPE | No  No | (Xu et al., 2014)  (Xu et al., 2014) | 45  45 |
| 362 | Placenta (chorionic plate)  Placenta (basal plate) | No difference in sPE  No difference in sPE | No  No | (Xu et al., 2014)  (Xu et al., 2014) | 45  45 |
| 31 | Placenta (chorionic plate)  Placenta (basal plate)  Placenta  MPB (plasma) | No difference in sPE  No difference in sPE  Up in sPE  Down in EOPE | No  No  Yes  Yes | (Xu et al., 2014)  (Xu et al., 2014)  (Vashukova et al., 2016)  (Dong et al., 2019) | 45  45  74  154 |
| 590 | Placenta (chorionic plate)  Placenta (basal plate) | No difference in sPE  No difference in sPE | No  No | (Xu et al., 2014)  (Xu et al., 2014) | 45  45 |
| 296 | Placenta (chorionic plate)  Placenta (basal plate) | No difference in sPE  No difference in sPE | No  No | (Xu et al., 2014)  (Xu et al., 2014) | 45  45 |
| 296-3p | MPB (plasma) | No difference in sPE at delivery and 1 year after | no | (Murphy et al., 2015) | 60 |
| 519e-5p | Placenta (chorionic plate)  Placenta (basal plate)  Placenta | Down in sPE  No difference in sPE  Up in sPE | Yes  No  Yes | (Xu et al., 2014)  (Xu et al., 2014)  (Vashukova et al., 2016) | 45  45  74 |
| 214 | Placenta (chorionic plate)  Placenta (basal plate) | No difference in sPE  Up in sPE | No  Yes | (Xu et al., 2014)  (Xu et al., 2014) | 45  45 |
| 92a | Placenta (chorionic plate)  Placenta (basal plate)  MPB (plasma)  Placenta (basal plate)  Placenta (chorionic plate) | Down in sPE  No difference in sPE  Down in sPE  Down in sPE  Down in sPE | Yes  No  Yes  Yes  Yes | (Xu et al., 2014)  (Xu et al., 2014)  (Xu et al., 2014)  (Zhang et al., 2015a)  (Zhang et al., 2015a) | 45  45  45  65  65 |
| 92a-3p | MPB (plasma)  UC blood | No difference in sPE at delivery and 1 year after  No difference in sPE | No  No | (Murphy et al., 2015)  (Hromadnikova et al., 2017) | 60  92 |
| 19b | MPB (plasma)  Placenta (basal plate)  Placenta (chorionic plate) | Down in sPE  No difference in sPE  Down in sPE | Yes  No  Yes | (Xu et al., 2014)  (Zhang et al., 2015a)  (Zhang et al., 2015a) | 45  65  65 |
| 19b-3p | MPB (plasma) | Up in sPE | No | (Luizon et al., 2021) | Luizon 2021 |
| 136 | Placenta (decisua MSCs)  MPB (plasma exosomes) | Up in sPE  Up in EOPE | Yes  Yes | (Zhao et al., 2014)  (Motawi et al., 2018) | 46  137 |
| 495 | Placenta (decisua MSCs)  MPB (plasma exosomes) | Up in sPE  Up in sPE | Yes  Yes | (Zhao et al., 2014)  (Motawi et al., 2018) | 46  137 |
| 494 | Placenta (decisua MSCs)  MPB (plasma exosomes) | Up in sPE  Up in EOPE | Yes  Yes | (Zhao et al., 2014)  (Motawi et al., 2018) | 46  137 |
| 140-5p | Placenta (eciduas MSCs) | Up in sPE | Yes | (Zhao et al., 2014) | 46 |
| 100 | Placenta (eciduas MSCs) | Up in sPE | Yes | (Zhao et al., 2014) | 46 |
| 100-5p | MPB (whole blood)  MPB (whole blood) | Down in sPE  Down in EOPE | Yes  Yes | (Hromadnikova et al., 2016)  (Hromadnikova et al., 2016) | 66  66 |
| 1207-5p | Placenta (eciduas MSCs) | Up in sPE | Yes | (Zhao et al., 2014) | 46 |
| 130a | Placenta | Down in EOPE | yes | (Hromadnikova et al., 2015a) | 52 |
| 145-5p | Placenta  UC blood  UC blood | Down in EOPE  Down in sPE  Down in EOPE | Yes  Yes  Yes | (Hromadnikova et al., 2015a)  (Hromadnikova et al., 2017)  (Hromadnikova et al., 2017) | 52  92  92 |
| 499-5p | Placenta | Up in sPE and EOPE | yes | (Hromadnikova et al., 2015a) | 52 |
| 520a-5p | Placenta  MPB (plasma) | Down in sPE  Up in sPE, sEOPE, sLOPE | Yes  yes | (Hromadnikova et al., 2015b)  (Miura et al., 2015) | 53  59 |
| 520a | Placenta | Up in sPE | Yes | (Vashukova et al., 2016) | 74 |
| 520h | MPB (plasma) | Up in sPE, sEOPE, sLOPE | yes | (Miura et al., 2015) | 59 |
| 526b | MPB (plasma) | Up in sPE, sEOPE, sLOPE | yes | (Miura et al., 2015) | 59 |
| 1323 | MPB (plasma) | Up in sPE, sEOPE, sLOPE | yes | (Miura et al., 2015) | 59 |
| 516b | MPB (plasma) | Up in sPE, sEOPE, sLOPE | yes | (Miura et al., 2015) | 59 |
| 516a-5p | MPB (plasma)  Placenta | Up in sPE, sEOPE, sLOPE  Up in sPE | Yes  Yes | (Miura et al., 2015)  (Vashukova et al., 2016) | 59  74 |
| 98 | MPB (plasma)  MPB (plasma)  Placenta | Up in sPE at delivery  No difference in sPE 1 year after delivery  Down in sPE | Yes  No  Yes | (Murphy et al., 2015)  (Murphy et al., 2015)  (Vashukova et al., 2016) | 60  60  74 |
| 15b-5p | MPB (plasma)  MPB (plasma) | No difference in sPE and 1 year after  Up in sPE | No  No | (Murphy et al., 2015)  (Luizon et al., 2021) | 60  Luizon 2021 |
| 135b | MPB (plasma)  Placenta  Placenta | Up in sPE  Up in sPE  Down in sPE | NR  NR  Yes | (Yang et al., 2015)  (Yang et al., 2015)  (Vashukova et al., 2016) | 64  64  74 |
| 142-3p | MPB (plasma)  Placenta | Up in sPE  Up in sPE | NR  NR | (Yang et al., 2015)  (Yang et al., 2015) | 64  64 |
| 149 | MPB (plasma)  Placenta  MPB (whole blood) | Up in sPE  Up in sPE  Down in PE | NR  NR  Yes | (Yang et al., 2015)  (Yang et al., 2015)  (Whigham et al., 2020) | 64  64  Whigham 2020 |
| 188-5p | MPB (plasma)  Placenta | Up in sPE  Up in sPE | NR  NR | (Yang et al., 2015)  (Yang et al., 2015) | 64  64 |
| 18b | MPB (plasma)  Placenta  Placenta (basal plate)  Placenta (chorionic plate) | Up in sPE  Up in sPE  No difference in sPE  Down in sPE | NR  NR  Yes  no | (Yang et al., 2015)  (Yang et al., 2015)  (Zhang et al., 2015a)  (Zhang et al., 2015a) | 64  64  65  65 |
| 203 | MPB (plasma)  Placenta | Up in sPE  Up in sPE | NR  NR | (Yang et al., 2015)  (Yang et al., 2015) | 64  64 |
| 205 | MPB (plasma)  Placenta | Up in sPE  Up in sPE | NR  NR | (Yang et al., 2015)  (Yang et al., 2015) | 64  64 |
| 27a | MPB (plasma)  Placenta  Placenta  MPB (serum) | Up in sPE  Up in sPE  Up in sPE  Up in sPE | NR  NR  Yes  Yes | (Yang et al., 2015)  (Yang et al., 2015)  (Zheng et al., 2020)  (Zheng et al., 2020) | 64  64  Zheng W 2020  Zheng W 2020 |
| 27a-3p | MPB (plasma) | Up in sPE | Yes | (Luizon et al., 2021) | Luizon 2021 |
| 301-3p | MPB (plasma)  Placenta | Up in sPE  Up in sPE | NR  NR | (Yang et al., 2015)  (Yang et al., 2015) | 64  64 |
| 518a-3p | MPB (plasma)  Placenta  Placenta | Up in sPE  Up in sPE  Up in sPE | NR  NR  Yes | (Yang et al., 2015)  (Yang et al., 2015)  (Vashukova et al., 2016) | 64  64  74 |
| 518e | MPB (plasma)  Placenta  Placenta | Up in sPE  Up in sPE  Up in sPE | NR  NR  Yes | (Yang et al., 2015)  (Yang et al., 2015)  (Vashukova et al., 2016) | 64  64  74 |
| 93 | MPB (plasma)  Placenta | Up in sPE  Up in sPE | NR  NR | (Yang et al., 2015)  (Yang et al., 2015) | 64  64 |
| 106 | Placenta (basal plate)  Placenta (chorionic plate) | Up in sPE  No difference in sPE | Yes  No | (Zhang et al., 2015a)  (Zhang et al., 2015a) | 65  65 |
| 499a-5p | MPB (whole blood) | No difference in sPE | No | (Hromadnikova et al., 2016) | 66 |
| 518c | Placenta | Up in sPE | Yes | (Vashukova et al., 2016) | 74 |
| 532 | Placenta | Up in sPE | Yes | (Vashukova et al., 2016) | 74 |
| 518f-5p | Placenta | Up in sPE | Yes | (Vashukova et al., 2016) | 74 |
| 527 | Placenta | Up in sPE | Yes | (Vashukova et al., 2016) | 74 |
| 300 | Placenta  Placenta | Up in sPE  Up in EOPE | Yes  Yes | (Gao et al., 2017)  (Gao et al., 2017) | 87  87 |
| let-7b | MPB (plasma) | Down in sLOPE | Yes | (Gunel et al., 2017) | 89 |
| let-7f-1 | MPB (plasma) | Down in sLOPE | Yes | (Gunel et al., 2017) | 89 |
| 1183 | MPB (plasma) | Up in sLOPE | Yes | (Gunel et al., 2017) | 89 |
| 23c | MPB (plasma) | Down in sLOPE | Yes | (Gunel et al., 2017) | 89 |
| 425 | MPB (plasma) | Down in sLOPE | Yes | (Gunel et al., 2017) | 89 |
| 520g | MPB (serum)  MPB (serum)  MPB (serum) | Up in sPE 1^st^ trimester  No difference in sPE 2^nd^ trimetsre  No difference in sPE 3^rd^ trimester | Yes  No  No | (Jiang et al., 2017)  (Jiang et al., 2017)  (Jiang et al., 2017) | 96  96  96 |
| 137 | Placenta | Up in sPE | Yes | (Lu et al., 2017) | 101 |
| 22 | Placenta | Up in sEOPE  Down in sLOPE | Yes  No | (Shao et al., 2017)  (Shao et al., 2017) | 108  108 |
| 22-3p | MPB (plasma) | Up in PE | No | (Luizon et al., 2021) | Luizon 2021 |
| 202-3p | Placenta (villi) | Up in sPE | Yes | (Singh et al., 2017) | 109 |
| 299 | Placenta | Up in sPE | Yes | (Gao et al., 2018b) | 126 |
| 376c | MPB (plasma)  Placenta  Placenta | Down in sPE  Down in sPE  No difference in preterm PE | Yes  Yes  No | (Li et al., 2018)  (Li et al., 2018)  (Yang et al., 2019a) | 132  132  186 |
| 376c-3p | MPB (plasma) | Up in sPE | No | (Luizon et al., 2021) | Luizon 2021 |
| 518-5p | Placenta | Up in EOPE | Yes | (Lykoudi et al., 2018) | 135 |
| 518b | MPB (leukocytes) | Up in EOPE | NR | (Demirer et al., 2020) | Demirer 2019 |
| 518f | MPB (serum) | Up in sPE | Yes | (Martinez-Fierro et al., 2018) | 136 |
| 539-5p | Placenta | Down in EOPE | Yes | (Timofeeva et al., 2018) | 141 |
| 423-5p | Placenta  MPB (plasma)  MPB (plasma exosomes)  MPB (plasma exosomes)  MPB (plasma exosomes) | Down in EOPE  Up in EOPE  Up in EOPE (11-13gw)  Up in EOPE (24-26gw)  Up in EOPE (30-32gw) | Yes  Yes  Yes  No  Yes | (Timofeeva et al., 2018)  (Timofeeva et al., 2018)  (Timofeeva et al., 2018)  (Timofeeva et al., 2018)  (Timofeeva et al., 2018) | 141  141  141  141  141 |
| 629-5p | Placenta  MPB (plasma) | Down in EOPE  Up in EOPE | Yes  Yes | (Timofeeva et al., 2018)  (Timofeeva et al., 2018) | 141  141 |
| 532-5p | Placenta  MPB (plasma exosomes)  MPB (plasma exosomes)  MPB (plasma exosomes) | Down in EOPE  Up in EOPE (11-13gw)  Up in EOPE (24-26gw)  No difference in EOPE (30-32gw) | Yes  Yes  Yes  No | (Timofeeva et al., 2018)  (Timofeeva et al., 2018)  (Timofeeva et al., 2018)  (Timofeeva et al., 2018) | 141  141  141  141 |
| 519-3p | Placenta  MPB (plasma) | Down in EOPE  Up in EOPE | Yes  Yes | (Timofeeva et al., 2018)  (Timofeeva et al., 2018) | 141  141 |
| 127-3p | Placenta | Down in EOPE | Yes | (Timofeeva et al., 2018) | 141 |
| let-7c-5p | Placenta  MPB (plasma) | Down in EOPE  Up in EOPE | Yes  Yes | (Timofeeva et al., 2018)  (Timofeeva et al., 2018) | 141  141 |
| 892c-3p | Placenta | Down in EOPE | Yes | (Wang et al., 2018c) | 145 |
| 378c | Placenta | Down in EOPE | Yes | (Wang et al., 2018c) | 145 |
| 514b-3p | Placenta | Down in EOPE | Yes | (Wang et al., 2018c) | 145 |
| 663 | Placenta | Up in EOPE | Yes | (Wang et al., 2018c) | 145 |
| 134 | Placenta (basal plate)  Placenta (chorionic plate) | Up in sPE  No difference in sPE | Yes  No | (Zou et al., 2018)  (Zou et al., 2018) | 148  148 |
| 193b-3p | Placenta (villi) | No difference in EOPE | No | (Awamleh et al., 2019) | 150 |
| 193-5p | Placenta (villi) | Up in EOPE | Yes | (Awamleh et al., 2019) | 150 |
| 365a/b3p | Placenta (villi) | No difference in EOPE | No | (Awamleh et al., 2019) | 150 |
| 520a-3p | Placenta (villi) | Up in EOPE | Yes | (Awamleh et al., 2019) | 150 |
| 210-5p | Placenta (villi) | No difference in EOPE | No | (Awamleh et al., 2019) | 150 |
| 181a-2-3p | Placenta (villi) | No difference in EOPE | No | (Awamleh et al., 2019) | 150 |
| 33b-3p | Placenta (villi) | No difference in EOPE | No | (Awamleh et al., 2019) | 150 |
| 21 | MPB (plasma) | Down in EOPE | Yes | (Dong et al., 2019) | 154 |
| 21-3p | MPB (leukocytes) | Not reported | NR | (Demirer et al., 2020) | Demirer 2019 |
| 133a-3p | MPB | Up in sPE | Yes | (Hromadnikova et al., 2019b) | 158 |
| 130b-3p | MPB | No difference in sPE | No | (Hromadnikova et al., 2019b) | 158 |
| 490-3p | MPB (plasma exosomes) | Up in EOPE | Yes | (Pillay et al., 2019) | 171 |
| 874-3p | MPB (plasma exosomes) | Up in EOPE | Yes | (Pillay et al., 2019) | 171 |
| 190a-5p | MPB (plasma exosomes) | Up in EOPE | Yes | (Pillay et al., 2019) | 171 |
| 23a-3p | MPB (plasma exosomes) | Up in EOPE | Yes | (Pillay et al., 2019) | 171 |
| 324-3p | MPB (plasma exosomes) | Up in EOPE | Yes | (Pillay et al., 2019) | 171 |
| 431-5p | MPB (plasma exosomes) | Up in EOPE | Yes | (Pillay et al., 2019) | 171 |
| 758-5p | MPB (plasma exosomes) | Up in EOPE | Yes | (Pillay et al., 2019) | 171 |
| 504-5p | MPB (plasma exosomes) | Up in EOPE | Yes | (Pillay et al., 2019) | 171 |
| 2113 | MPB (plasma exosomes) | Down in EOPE | Yes | (Pillay et al., 2019) | 171 |
| 374-5p | MPB (plasma exosomes) | Down in EOPE | Yes | (Pillay et al., 2019) | 171 |
| 204-5p | MPB (plasma exosomes)  MPB (plasma) | Down in EOPE  Up in sPE | Yes  Yes | (Pillay et al., 2019)  (Luizon et al., 2021) | 171  Luizon 2021 |
| 139-5p | MPB (plasma exosomes)  Placenta | Down in EOPE  Down in sPE | Yes  Yes | (Pillay et al., 2019)  (Huang et al., 2020) | 171  Huang 2020 |
| 449c-5p | MPB (plasma exosomes) | Down in EOPE | Yes | (Pillay et al., 2019) | 171 |
| 1275 | MPB (plasma exosomes) | Down in EOPE | Yes | (Pillay et al., 2019) | 171 |
| 320d | MPB (plasma exosomes) | Down in EOPE | Yes | (Pillay et al., 2019) | 171 |
| 452-5p | MPB (plasma exosomes) | Down in EOPE | Yes | (Pillay et al., 2019) | 171 |
| 378b | MPB (plasma exosomes) | Down in EOPE | Yes | (Pillay et al., 2019) | 171 |
| 424 | Placenta | Down in sPE | Yes | (Tang et al., 2019) | 176 |
| 441 | Placenta | Down in preterm PE | Yes | (Yang et al., 2019a) | 186 |
| 1972 | MPB (plasma) | Up in EOPE | Yes | (Lip et al., 2020) | Lip 2019 |
| 4793-3p | MPB (plasma) | Up in EOPE | Yes | (Lip et al., 2020) | Lip 2019 |
| 483 | MPB (serum)  MPB (serum)  UCB  UCB  Placenta  Placenta | Down in mild EOPE  Down in severe EOPE  Down in mild EOPE  Down in severe EOPE  Down in mild EOPE  Down in severe EOPE | Yes  Yes  Yes  Yes  Yes  Yes |  | Han 2020  Han 2020  Han 2020  Han 2020  Han 2020  Han 2020 |
| 383 | Placenta | Down in sPE | Yes |  | Li T 2020 |
| 1283 | MPB (whole blood) | No difference in sEOPE | No | (Whigham et al., 2020) | Whigham 2020 |
| 200a | Placenta | Up in sPE | Yes |  | Wu G, 2020 |
| 320a | MPB (serum)  MPB (serum)  MPB (serum) | Up in EOPE  Up in mEOPE  Up in sEOPE | Yes  Yes  Yes |  | Liao, 2021  Liao 2021  Liao 2021 |
| 106b-5p | MPB (plasma) | Up in sPE | No | (Luizon et al., 2021) | Luizon 2021 |
| 15a-5p | MPB (plasma) | Up in sPE | No | (Luizon et al., 2021) | Luizon 2021 |
| 128-3p | MPB (plasma) | Up in sPE | No | (Luizon et al., 2021) | Luizon 2021 |
| 200b-3p | MPB (plasma) | Up in sPE | No | (Luizon et al., 2021) | Luizon 2021 |
| 34a-5p | MPB (plasma) | Up in sPE | No | (Luizon et al., 2021) | Luizon 2021 |
| 200c | MPB (plasma) | Up in EOPE | Yes | (Witvrouwen et al., 2021) | Witvrouwen 2021 |

Table S6. Major findings for less severe PE

| **miRNK** | **tissue** | **Up/Down** | **Sig.** | **Ref.** |
| --- | --- | --- | --- | --- |
| 210 | Placenta (villi)  MPB (plasma)  MPB  Placenta (villi) | Down in mPE  Up in mPE  NR  Up in mPE | Yes  Yes  NR  Yes | 3  21  44  82 |
| 210-3p | MPB (plasma)  MPB (plasma)  MPB (plasma)  MPB (plasma)  Placenta | No difference in mPE at delivery No difference in mPE 1 year after delivery  No difference in mPE  No difference in LOPE  Up in term-PE | No  No  No  No  Yes | 60  60  Jelena 2020  Jelena 2020  Hayder 2021 |
| 152 | Placenta (villi) | Down in mPE | Yes | 3 |
| 411 | Placenta (villi) | Down in mPE | Yes | 3 |
| 377 | Placenta (villi)  Placenta | Down in mPE  No difference in term PE | Yes  No | 3  186 |
| 519b | Placenta (villi) | Down in mPE | Yes | 3 |
| 18a | Placenta (villi)  MPB (plasma)  Placenta  MPB (whole blood)  Placenta | Down in mPE  Up in mPE  Up in mPE  Down in PE 36GW  No difference term PE | Yes  NR  NR  Yes  No | 3  64  64  Whigham 2020  Whigham 2020 |
| 18b | MPB (plasma)  Placenta | Up in mPE  Up in mPE | NR  NR | 64  64 |
| 18a-5p | MPB (plasma) | No difference in mPE at delivery No difference in mPE 1 year after delivery | No  No | 60  60 |
| 363 | Placenta (villi)  MPB (whole blood)  Placenta | Down in mPE  Down in PE 36GW  Down in term PE | Yes  Yes  Yes | 3  Whigham 2020  Whigham 2020 |
| 542-3p | Placenta (villi) | Down in mPE | Yes | 3 |
| 24 | Placenta | NR | NR | 9 |
| 517a | Placenta | NR | NR | 9 |
| 517-5p | Placenta  Placenta | Down in mPE  Down in LOPE | Yes  Yes | 53  53 |
| 519a | Placenta  Placenta | NR  Down in LOPE | NR  Yes | 9  53 |
| 519d | Placenta  Placenta  MPB (plasma)  Placenta | NR  Down in LOPE  Up in mPE  Up in mPE | NR  yes  NR  NR | 9  53  64  64 |
| 517c | Placenta  Placenta  MPB (plasma)  Placenta | NR  No difference in term PE  Up in mPE  Up in mPE | NR  No  NR  NR | 9  49  64  64 |
| 517c-3p | MPB (plasma exosomes) | Down in LOPE | Yes | 159 |
| 517a/b | Placenta | Up in term PE | Yes | 49 |
| 521 | Placenta | NR | NR | 9 |
| 23a | Placenta | NR | NR | 9 |
| 103 | Placenta | NR | NR | 9 |
| 520g | Placenta | NR | NR | 9 |
| 520a-5p | Placenta  Placenta | Down in mPE  Down in LOPE | Yes  Yes | 53  53 |
| 520h | Placenta  Placenta | Down in mPE  Down in LOPE | Yes  Yes | 53  53 |
| 451 | Placenta | NR | NR | 9 |
| 451a | MPB (plasma exosomes) | Up in LOPE | Yes | 159 |
| let-7d | MPB (serum) | Up in mPE | Yes | 11 |
| let-7f | MPB (serum) | Up in mPE | Yes | 11 |
| let-7f-5p | MPB (plasma)  MPB (plasma) | No difference in mPE at delivery  No difference in mPE 1 year after delivery | No  No | 60  60 |
| 223 | MPB (serum)  MPB | Up in mPE  NR | Yes  NR | 11  44 |
| 141 | MPB (plasma) | Up in mPE | Yes | 29 |
| 144 | MPB (plasma) | Down in mPE | Yes | 29 |
| 144-3p | MPB | NR | NR | 44 |
| 221 | MPB (plasma) | No difference in mPE | No | 29 |
| 221-3p | MPB (plasma)  MPB (plasma)  MPB (whole blood)  MPB (whole blood)  UCB | No difference in mPE at delivery  No difference in mPE 1 year after delivery  No difference in mPE  No difference in LOPE  No difference in mPE | No  No  No  No  No | 60  60  66  66  92 |
| 221-5p | MPB | NR | NR | 44 |
| 29a | MPB (plasma) | Up in mPE | Yes | 29 |
| 29a-3p | MPB (plasma)  MPB (plasma)  MPB | No difference in mPE at delivery No difference in mPE 1 year after delivery  No difference in mPE | No  No  No | 60  60  158 |
| 223-3p | Placenta | No difference in LOPE | No | 43 |
| 224-5p | Placenta | No difference in LOPE | No | 43 |
| 224 | MPB (plasma)  Placenta | Up in mPE  Up in mPE | NR  NR | 64  64 |
| 1301 | Placenta | No difference in LOPE | No | 43 |
| 340-5p | MPB | NR | NR | 44 |
| 424 | Placenta | No difference in term PE | No | Whigham 2020 |
| 424-5p | MPB | NR | NR | 44 |
| 33a-5p | MPB | NR | NR | 44 |
| 7-5p | MPB | NR | NR | 44 |
| 1229 | MPB | NR | NR | 44 |
| 1267 | MPB | NR | NR | 44 |
| 671-3p | MPB | NR | NR | 44 |
| 1 | MPB | NR | NR | 44 |
| 1-3p | Placenta | Up in LOPE | Yes | 52 |
| 133b | MPB | NR | NR | 44 |
| 582-5p | MPB | NR | NR | 44 |
| 30e-3p | MPB | NR | NR | 44 |
| 199a-5p | MPB  MPB (whole blood)  MPB (whole blood)  UCB | NR  No difference in mPE  No difference in LOPE  No difference in mPE | NR  No  No  No | 44  66  66  92 |
| 199b-5p | MPB | NR | NR | 44 |
| 575 | MPB | NR | NR | 44 |
| 301a-3p | MPB  MPB (plasma)  Placenta | NR  Up in mPE  Up in mPE | NR  NR  NR | 44  64  64 |
| 148a-3p | MPB | NR | NR | 44 |
| 193a-3p | MPB | NR | NR | 44 |
| 219-5p | MPB | NR | NR | 44 |
| 132 | MPB | NR | NR | 44 |
| 513a-5p | MPB | NR | NR | 44 |
| 1244 | MPB | NR | NR | 44 |
| 16 | MPB  MPB (plasma)  MPB (plasma)  Placenta  MPB (serum) | NR  No difference in LOPE  No difference in LOPE  No difference in term PE  Up in LOPE | NR  No  No  No  Yes | 44  154  Dong 2019  Whigham 2020  Ali 2021 |
| 16-5p | MPB (plasma) | No difference in mPE at delivery No difference in mPE 1 year after delivery | No  No | 60  60 |
| 146a | MPB  Placenta (syncytiotrophoblast)  Placenta (syncytial knots) | NR  Down in LOPE  Down in LOPE | NR  Yes  Yes | 44  106  106 |
| 146a-5p | MPB (whole blood)  MPB (whole blood) | No difference in mPE  No difference in LOPE | No  No | 66  66 |
| 155 | MPB  Placenta (syncytiotrophoblast) | NR  Down in LOPE | NR  No | 44  106 |
| 155-5p | MPB (plasma) | No difference in mPE at delivery No difference in mPE 1 year after delivery | No  No | 60  60 |
| 181a | MPB | NR | NR | 44 |
| 181a-5p | MPB (plasma) | No difference in mPE at delivery No difference in mPE 1 year after delivery | No  No | 60  60 |
| 196a | MPB | NR | NR | 44 |
| 26a-5p | Placenta  UCB  UCB  MPB (plasma exosomes) | No difference in LOPE  No difference in mPE  No difference in LOPE  Down in LOPE | No  No  No  Yes | 52  92  92  159 |
| 103a-3p | Placenta  MPB (whole blood) | No difference in LOPE  No difference in mPE | No  No | 52  66 |
| 145-5p | Placenta  UCB  UCB | No difference in LOPE  No difference in mPE  No difference in LOPE | No  No  No | 52  92  92 |
| 499-5p | Placenta  Placenta | Up in mPE  Up in LOPE | Yes  Yes | 52  52 |
| 499a-5p | MPB (whole blood) | No difference in mPE | No | 66 |
| 515-5p | Placenta | Down in LOPE | Yes | 53 |
| 518-5p | Placenta | Up in LOPE | Yes | 135 |
| 518b | Placenta  MPB (plasma)  MPB (plasma) | Down in LOPE  Up in mPE  No difference in LOPE | Yes  Yes  No | 53  Jelena 2020  Jelena 2020 |
| 518f-5p | Placenta | Down in LOPE | Yes | 53 |
| 518f | MPB (serum) | Up in mPE (20gw) | Yes | 136 |
| 524-5p | Placenta  Placenta | Down in mPE  Down in LOPE | Yes  Yes | 53  53 |
| 525 | Placenta  Placenta | Down in mPE  Down in LOPE | Yes  Yes | 53  53 |
| 98-5p | MPB (plasma) | No difference in mPE at delivery No difference in mPE 1 year after delivery | No  No | 60  60 |
| 222-3p | MPB (plasma) | No difference in mPE at delivery No difference in mPE 1 year after delivery | No  No | 60  60 |
| 126 | MPB (plasma)  Placenta | Up in mPE  Up in mPE | NR  NR | 64  64 |
| 126* | MPB (plasma)  Placenta | Up in mPE  Up in mPE | NR  NR | 64  64 |
| 126-3p | MPB (plasma)  MPB (plasma)  MPB | No difference in mPE at delivery No difference in mPE 1 year after delivery  No difference in mPE | No  No  No | 60  60  158 |
| 130a-3p | MPB (plasma) | No difference in mPE at delivery No difference in mPE 1 year after delivery | No  No | 60  60 |
| 130a | MPB (plasma)  Placenta | Up in mPE  Up in mPE | NR  NR | 64  64 |
| 17 | Placenta (sincytiothrophoblast) Endothelium | NR  NR | NR  NR | 139  139 |
| 17-5p | MPB (plasma)  MPB (plasma)  MPB (whole blood)  MPB | No difference in mPE at delivery No difference in mPE 1 year after delivery  No difference in mPE  No difference in mPE | No  No  No  No | 60  60  66  158 |
| 19a-3p | MPB (plasma) | No difference in mPE at delivery No difference in mPE 1 year after delivery | No  No | 60  60 |
| 19a-3p | MPB (plasma) | No difference in mPE at delivery No difference in mPE 1 year after delivery | No  No | 60  60 |
| 29a | MPB (plasma)  Placenta | Up in mPE  Up in mPE | NR  NR | 64  64 |
| 92a-3p | MPB (plasma)  MPB (plasma)  UCB | No difference in mPE at delivery No difference in mPE 1 year after delivery  Up in mPE | No  No  Yes | 60  60  92 |
| 20a-5p | MPB (plasma) | No difference in mPE at delivery No difference in mPE 1 year after delivery | No  No | 60  60 |
| 20b-5p | MPB (plasma)  MPB (plasma)  MPB | No difference in mPE at delivery No difference in mPE 1 year after delivery  No difference in mPE | No  No  No | 60  60  158 |
| 15b-5p | MPB (plasma) | No difference in mPE at delivery No difference in mPE 1 year after delivery | No  No | 60  60 |
| 296-3p | MPB (plasma) | No difference in mPE at delivery No difference in mPE 1 year after delivery | No  No | 60  60 |
| 195-5p | MPB (plasma)  MPB (plasma)  MPB (whole blood)  UCB | No difference in mPE at delivery No difference in mPE 1 year after delivery  No difference in mPE  No difference in mPE | No  No  No  No | 60  60  66  92 |
| 29b-3p | MPB (plasma) | No difference in mPE at delivery No difference in mPE 1 year after delivery | No  No | 60  60 |
| 130a | MPB (plasma)  Placenta | Up in mPE  Up in mPE | NR  NR | 64  64 |
| 135b | MPB (plasma)  Placenta | Up in mPE  Up in mPE | NR  NR | 64  64 |
| 142-3p | MPB (plasma)  Placenta | Up in mPE  Up in mPE | NR  NR | 64  64 |
| 149 | MPB (plasma)  Placenta  Placenta | Up in mPE  Up in mPE  Down in term PE | NR  NR  Yes | 64  64  Whigham 2020 |
| 188-5p | MPB (plasma)  Placenta | Up in mPE  Up in mPE | NR  NR | 64  64 |
| 203 | MPB (plasma)  Placenta | Up in mPE  Up in mPE | NR  NR | 64  64 |
| 205 | MPB (plasma)  Placenta | Up in mPE  Up in mPE | NR  NR | 64  64 |
| 224 | MPB (plasma)  Placenta | Up in mPE  Up in mPE | NR  NR | 64  64 |
| 27a | MPB (plasma)  Placenta | Up in mPE  Up in mPE | NR  NR | 64  64 |
| 518a-3p | MPB (plasma)  Placenta | Up in mPE  Up in mPE | NR  NR | 64  64 |
| 518e | MPB (plasma)  Placenta | Up in mPE  Up in mPE | NR  NR | 64  64 |
| 93 | MPB (plasma)  Placenta | Up in mPE  Up in mPE | NR  NR | 64  64 |
| 100-5p | MPB (whole blood)  MPB (whole blood) | Down in mPE  Down in LOPE | Yes  Yes | 66  66 |
| 125-5p | MPB (whole blood)  MPB (whole blood) | Down in mPE  Down in LOPE | Yes  Yes | 66  66 |
| 574-3p | MPB (whole blood)  UCB  UCB | No difference in mPE  No difference in mPE  No difference in mPE | No  No  No | 66  92  92 |
| 300 | Placenta (villi)  Placenta (villi) | Up in mPE  Up in LOPE | Yes  Yes | 87  87 |
| 137 | Placenta | Up in mPE | Yes | 101 |
| 218-5p | Placenta (trophoblast cells) | Down in LOPE | Yes | 119 |
| 376c | MPB (plasma)  Placenta  Placenta  MPB (plasma)  MPB (plasma)  MPB (plasma) | Down in mPE  Down in mPE  Down in term PE  No difference in LOPE in 1^st^ trimester  No difference in LOPE in 2^nd^ trimester  No difference in LOPE in 3^rd^ trimester | Yes  Yes  Yes  No  No  No | 132  132  186  Devor 2020  Devor 2020  Devor 2020 |
| 520d-3p | MPB (serum) | Up in mPE (16gw and 20gw) | Yes | 136 |
| 136 | MPB (plasma exosomes) | Up in LOPE | Yes | 137 |
| 494 | MPB (plasma exosomes) | Up in LOPE | Yes | 137 |
| 495 | MPB (plasma exosomes) | Up in LOPE | Yes | 137 |
| 663 | Placenta | No difference in LOPE | No | 145 |
| 31 | MPB (plasma)  MPB (plasma) | No difference in LOPE  No difference in LOPE | No  No | 154  Dong 2019 |
| 21 | MPB (plasma)  MPB (plasma) | Down in LOPE  Down in LOPE | Yes  Yes | 154  Dong 2019 |
| 133-3p | MPB | No difference in mPE | No | 158 |
| 130b-3p | MPB | No difference in mPE | No | 158 |
| 202-3p | MPB (plasma exosomes) | Up in LOPE | Yes | 159 |
| 297 | MPB (plasma exosomes) | Up in LOPE | Yes | 159 |
| 375 | MPB (plasma exosomes) | Down in LOPE | Yes | 159 |
| 488-3p | MPB (plasma exosomes) | Down in LOPE | Yes | 159 |
| 504-5p | MPB (plasma exosomes) | Down in LOPE | Yes | 159 |
| 2113 | MPB (plasma exosomes) | Down in LOPE | Yes | 159 |
| 374-5p | MPB (plasma exosomes) | Down in LOPE | Yes | 159 |
| 379-5p | MPB (plasma exosomes) | Down in LOPE | Yes | 159 |
| 376b-5p | MPB (plasma exosomes) | Down in LOPE | Yes | 159 |
| 150-5p | MPB (plasma exosomes) | Down in LOPE | Yes | 159 |
| 122 | MPB (plasma exosomes) | Up in LOPE | Yes | 159 |
| 3605 | MPB (plasma exosomes) | Down in LOPE | Yes | 159 |
| 134 | MPB (plasma)  MPB (plasma)  MPB (plasma) | Up in LOPE in 1^st^ trimester  No difference in LOPE in 2^nd^ trimester  No difference in LOPE in 3^rd^ trimester | Yes  No  No | Devor 2020  Devor 2020  Devor 2020 |
| 196b | MPB (plasma)  MPB (plasma)  MPB (plasma) | No difference Up in LOPE in 1^st^ trimester  No difference in LOPE in 2^nd^ trimester  Down in LOPE in 3^rd^ trimester | No  No  Yes | Devor 2020  Devor 2020  Devor 2020 |
| 302c | MPB (plasma)  MPB (plasma)  MPB (plasma) | No difference in LOPE in 1^st^ trimester  No difference in LOPE in 2^nd^ trimester  Up in LOPE in 3^rd^ trimester | No  No  Yes | Devor 2020  Devor 2020  Devor 2020 |
| 346 | MPB (plasma)  MPB (plasma)  MPB (plasma) | No difference in LOPE in 1^st^ trimester  No difference in LOPE in 2^nd^ trimester  Up in LOPE in 3^rd^ trimester | No  No  Yes | Devor 2020  Devor 2020  Devor 2020 |
| 486-3p | MPB (plasma)  MPB (plasma)  MPB (plasma) | Up in LOPE in 1^st^ trimester  No difference in LOPE in 2^nd^ trimester  No difference in LOPE in 3^rd^ trimester | Yes  No  No | Devor 2020  Devor 2020  Devor 2020 |
| 590-5p | MPB (plasma)  MPB (plasma)  MPB (plasma) | Up in LOPE in 1^st^ trimester  No difference in LOPE in 2^nd^ trimester  No difference in LOPE in 3^rd^ trimester | Yes  No  No | Devor 2020  Devor 2020  Devor 2020 |
| 618 | MPB (plasma)  MPB (plasma)  MPB (plasma) | No difference in LOPE in 1^st^ trimester  No difference in LOPE in 2^nd^ trimester  Up in LOPE in 3^rd^ trimester | No  No  Yes | Devor 2020  Devor 2020  Devor 2020 |
| 1283 | MPB (whole blood)  Placenta | Down in PE 36GW  No difference in term PE | Yes  Yes | Whigham 2020  Whigham 2020 |
| 200a | Placenta | Up in mPE | Yes | Wu G, 2020 |

Table S7. Major findings for not specified PE

| **miRNK** | **tissue** | **Up/Down** | **Sig.** | **Ref.** |
| --- | --- | --- | --- | --- |
| 518c | Placenta  Placenta | Down in PE  Up in PE | Yes  Yes | 7  14 |
| 1 | Placenta  MPB (serum)  MPB  MPB | Down in PE  No difference in PE  No difference vs. preconceptional  No difference vs. 1^st^ trimester control | Yes  No  No  No | 7  57  63  63 |
| 1-3p | MPB | No difference | No | 158 |
| 103 | Placenta  Placenta | Down in PE  NR | Yes  NR | 7  9 |
| 103a-3p | MPB (whole blood) | No difference | No | 66 |
| 15a | Placenta | Down in PE | Yes | 7 |
| 15a-3p | MPB | Down in PE | Yes | 76 |
| 584 | Placenta  Placenta  MPB (serum) | Down in PE  Up in PE  No difference | Yes  Yes  No | 7  55  57 |
| 584-5p | MPB (plasma exosomes) | Up in PE | Yes | 110 |
| 324-5p | Placenta | Down in PE | Yes | 7 |
| 200a | MPB (plasma)  MPB (plasma)  Placenta | Up in PE  Up in PE  Up in PE | Yes  Yes  Yes | 177  Cao 2019  Cao 2019 |
| 200b | Placenta  MPB (plasma) | Down in PE  No difference | Yes  No | 7  177 |
| 210 | Placenta  MPB (plasma)  Placenta (villi)  Placenta  MPB (serum)  Placenta  Placenta  MPB (serum)  MPB (serum)  MPB  MPB  Placenta (chorionic plate)  MPB (serum)  Urine  MPB (plasma)  Placenta  MPB (serum)  MPB (buffy coat)  MPB (plasma)  Placenta  Placenta  Placenta  MPB (serum)  MPB (plasma)  MPB (plasma) | Up in PE  NR  NR  Up in PE  Up in PE  Up in PE  Up in PE  Up in PE  Up in 1^st^, 2^nd^ and 3^rd^ trimester  No difference vs. preconceptional  No difference vs. 1^st^ trimester control  Up in PE  Up in PE  No difference  Up in PE  Up in PE  NR  NR  Up in PE  Up in PE  Up in PE  Down in PE  Up in PE  Down in PE  Up in mPE | Yes  NR  NR  Yes  Yes  Yes  Yes  NR  Yes  No  No  Yes  Yes  No  Yes  Yes  NR  NR  Yes  Yes  Yes  Yes  Yes  Yes  Yes | 7  8  10  14  22  23  36  41  57  63  63  81  86  86  95  98  128  146  151  151  152  180  191  Akgor 2020  Jairajpuri 2021 |
| 210-3p | MPB (plasma)  MPB (plasma)  MPB (serum)  MPB (plasma) | No difference at delivery  No difference 1 year after delivery  Up in PE  Up in PE | No  No  Yes  Yes | 60  60  70  170 |
| 210-5p | MPB (serum)  MPB (plasma) | Up in PE  Up in PE | Yes  No | 70  Kolkova 2021 |
| 154 | Placenta | Down in PE | Yes | 7 |
| 153 | MPB (plasma) | NR | NR | 8 |
| 24 | Placenta | NR | NR | 9 |
| 24-3p | MPB (plasma) | Down in PE | Yes | Akgor 2020 |
| 24-1-3p | MPB (serum) | No difference | No | 70 |
| 521 | Placenta | NR | NR | 9 |
| 29b | Placenta  MPB | NR  Up in PE | NR  Yes | 9  Dong 2020 |
| 29b-3p | MPB (plasma)  MPB (plasma) | No difference at delivery  No difference 1 year after delivery | No  No | 60  60 |
| 515 | Placenta | NR | NR | 9 |
| 515-5p | Placenta | Down in PE | Yes | 53 |
| 424 | Placenta  MPB (whole blood)  MPB (whole blood) | NR  Down in PE 36GW  No difference 28GW | NR  Yes  No | 9  Whigham 2020  Whigham 2020 |
| 424-5p | MPB  MPB | No difference in PE vs. preconceptual  Down in PE vs. 1^st^ trimester control | No  Yes | 63  63 |
| 143 | Placenta | NR | NR | 9 |
| 143-3p | MPB | No difference | No | 158 |
|  |  |  |  |  |
| 145 | Placenta  Placenta  Placenta (villi) | NR  Down in PE  Down in PE | NR  Yes  Yes | 9  91  121 |
| 145-5p | Placenta (trophoblast debris)  Placenta | Up in PE  Down in PE | Yes  Yes | 113  Lv 2019 |
| 23a | Placenta  MPB (plasma)  Placenta  Placenta | NR  No difference  Up in PE  Up in PE | NR  No  Yes  Yes | 9  48  99  Fan 2020 |
| 23a-3p | MPB (plasma exosomes) | Down in PE | Yes | 110 |
| 29c | Placenta | NR | NR | 9 |
| 15b | Placenta (villi) | NR | NR | 10 |
| 15b-5p | MPB (plasma)  MPB (plasma) | No difference at delivery  No difference 1 year after delivery | No  No | 60  60 |
| 181a | Placenta (villi)  MPB  MPB  MPB (serum)  MPB (serum)  Placenta | NR  No difference vs. preconceptional  No difference vs. 1^st^ trimester control  No difference  No difference  Up in PE | NR  No  No  No  No  Yes | 10  63  63  70  130  130 |
| 181a-5p | MPB (plasma)  MPB (plasma)  Placenta  MPB  Placenta | Up at delivery  No difference 1 year after delivery  Up in PE  No difference  Up in PE | Yes  No  Yes  No  Yes | 60  60  144  158  161 |
| 181a-3p | MPB (serum) | No difference | No | 70 |
| 200c | Placenta (villi)  Placenta | NR  Up in PE | NR  Yes | 10  67 |
| 296-3p | Placenta (villi)  Placenta  MPB (plasma)  MPB (plasma) | NR  Up in PE  Up at delivery  No difference 1 year after delivery | NR  Yes  Yes  No | 10  24  60  60 |
| 296-5p | Placenta | Up in PE | Yes | 24 |
| 377 | Placenta (villi)  MPB (serum)  MPB (plasma) | NR  No difference  No difference | NR  No  No | 10  57  186 |
| 483-5p | Placenta (villi) | NR | NR | 10 |
| 493 | Placenta (villi) | NR | NR | 10 |
| 516-5p | MPB (plasma)  MPB (plasma) | NR  Up in PE | NR  Yes | 13  27 |
| 516b-5p | Placenta (chorionic plate)  MPB (plasma exosomes) | Up in PE  No difference | Yes  No | 81  159 |
| 517 | MPB (plasma)  MPB (plasma) | NR  Up in PE | NR  Yes | 13  27 |
| 517-5p | Placenta  MPB (plasma exosomes) | Down in PE  Down in PE | Yes  Yes | 53  159 |
| 518b | MPB (plasma)  Placenta  Placenta  MPB (plasma exosomes)  MPB (leukocytes) | NR  No difference  Down in PE  No difference  Up in PE | NR  No  Yes  No  Yes | 13  36  53  159  Demirer 2019 |
| 520a | MPB (plasma)  MPB (plasma)  MPB (serum) | NR  Up in PE  Up in PE | NR  Yes  NR | 13  27  41 |
| 520a-5p | Placenta  Placenta  Placenta (chorionic plate)  MPB (plasma exosomes) | Down in PE  Down in PE  Up in PE  Down in PE | Yes  Yes  Yes  Yes | 26  53  81  159 |
| 520c-3p | Placenta | Down in PE | Yes | 166 |
| 520h | MPB (plasma)  Placenta  MPB (plasma exosomes) | NR  Down in PE  No difference | NR  Yes  No | 13  53  159 |
| 525 | MPB (plasma)  MPB (plasma)  Placenta | NR  Up in PE  Down in PE | NR  Yes  Yes | 13  27  53 |
| 525-5p | Placenta  MPB (plasma exosomes) | Up in PE  Down in PE | Yes  Yes | 14  159 |
| 526-5p | Placenta (trophoblast debris) | Up in PE | Yes | 113 |
| 526a | MPB (plasma)  MPB (plasma)  Placenta | NR  Up in PE  Down in PE | NR  Yes  Yes | 13  27  53 |
| 16 | MPB (plasma)  MPB  MPB  MPB (plasma)  Placenta  Placenta  MPB  MPB (whole blood)  MPB (whole blood)  MPB (serum)  Placenta | NR  Up in PE vs. preconceptional  Down in PE vs. 1^st^ trimester control  No difference  No difference  Up in PE  No difference  Down in PE 36GW  No difference 28GW  Up in PE  Up in PE | NR  Yes  Yes  No  No  Yes  No  Yes  No  Yes  Yes | 13  63  63  151  151  Yuan 2019  Dong 2020  Whigham 2020  Whigham 2020  Ali 2021  Zhao 2021 |
| 16-5p | MPB (plasma)  MPB (plasma)  MPB (leukocytes)  MPB (plasma) | No difference at delivery  No difference 1 year after delivery  Down in PE  Down in PE | No  No  Yes  No | 60  60  157  Kolkova 2021 |
| 16-2-3p | MPB (serum) | No difference | No | 70 |
| let-7a-5p | MPB (plasma exosomes) | Up in PE | Yes | 110 |
| let-7d | MPB (plasma)  Placenta | NR  Up in PE | NR  Yes | 13  Xu 2021 |
| 193b | Placenta | Up in PE | Yes | 14 |
| 144 | Placenta  MPB (serum)  Placenta  Placenta | Up in PE  Down in PE  Down in PE  Up in PE | Yes  NR  Yes  Yes | 14  41  115  Zhu 2020 |
| 144-3p | MPB  MPB  MPB (serum)  MPB (plasma exosomes)  Placenta | No difference vs. preconceptional  No difference vs. 1^st^ trimester control  No difference  Down in PE  Down in PE | No  No  Yes  Yes  Yes | 63  63  70  110  160 |
| 18a | Placenta  MPB (plasma) | Up in PE  Down in PE | Yes  Yes | 14  95 |
| 18a-5p | MPB (plasma)  MPB (plasma) | No difference at delivery  No difference 1 year after delivery | No  No | 60  60 |
| 185 | Placenta | Up in PE | Yes | 14 |
| 19a | Placenta | Up in PE | Yes | 14 |
| 19a-3p | MPB (plasma)  MPB (plasma)  Placenta  MPB (serum) | No difference at delivery  No difference 1 year after delivery  Down in PE  Down in PE | No  No  Yes  Yes | 60  60  178  178 |
| 590-5p | Placenta  MPB (plasma exosomes) | Up in PE  Up in PE | Yes  Yes | 14  153 |
| 142-3p | Placenta  Placenta  MPB (plasma)  Placenta | Up in PE  Up in PE  No difference  Up in PE | Yes  Yes  No  Yes | 14  164  Akgor 2020  Mao 2021 |
| 451 | Placenta | Up in PE | Yes | 14 |
| 451a | MPB  MPB (plasma exosomes) | Up in PE  Down in PE | Yes  Yes | 76  110 |
| 22 | Placenta | Up in PE | Yes | 14 |
| 526b | Placenta  Placenta | Up in PE  No difference | Yes  No | 14  36 |
| 520-3p | Placenta | Up in PE | Yes | 14 |
| 10b | Placenta | Up in PE | Yes | 14 |
| 10b-3p | Placenta | Down in PE | Yes | 163 |
| 20a | Placenta  Placenta  Placenta | Up in PE  Up in PE  Up in PE | Yes Yes  Yes | 14  42  67 |
| 20a-5p | MPB (plasma)  MPB (plasma)  MPB | No difference at delivery  No difference 1 year after delivery  No difference | No  No  No | 60  60  158 |
| 518f | Placenta | Up in PE | Yes | 14 |
| 518f-5p | Placenta | Down in PE | Yes | 53 |
| 146b-5p | Placenta | Up in PE | Yes | 14 |
| 517c | Placenta  MPB (plasma)  Placenta | Up in PE  No difference  Up in PE | Yes  No  Yes | 14  151  151 |
| 517c-3p | MPB (plasma) | Up in PE | Yes | 170 |
| 519e | Placenta | Up in PE | Yes | 14 |
| 126 | Placenta  Placenta  Placenta  Subcutaneous adipose tissue  MPB | Up in PE  Down in PE  Down in PE  Down in PE  No difference | Yes  Yes  Yes  Yes  No | 14  31  35  Xu 2019  Dong 2020 |
| 126-3p | MPB (plasma)  MPB (plasma)  MPB  Placenta | No difference at delivery  No difference 1 year after delivery  No difference  Down in PE | No  No  No  Yes | 60  60  158  Chu 2021 |
| 182 | Placenta  MPB (serum)  MPB (serum) | Up in PE  No difference in 1^st^ and 2^nd^ trimester  Up in 3^rd^ trimester | Yes  No  Yes | 23  57  57 |
| 182-5p | Placenta | Up in PE | Yes | 123 |
| 30a-3p | Placenta  Placenta  Placenta | No difference  Up in PE  No difference | No  Yes  No | 23  138  144 |
| 30a | UC veins  UC-MSCs | Up in PE  Up in PE | Yes  Yes | 54  54 |
| 30c-5p | MPB (plasma exosomes) | Up in PE | Yes | 110 |
| 92b | Placenta | Up in PE | Yes | 24 |
| 197 | Placenta | Up in PE | Yes | 24 |
| 197-3p | MPB (plasma) | Down in PE | Yes | Akgor 2020 |
| 342 | Placenta | Up in PE | Yes | 187 |
| 342-3p | Placenta  MPB (serum)  MPB (plasma) | Up in PE  No difference  No difference | Yes  No  No | 24  70  Akgor 2020 |
| 26a-5p | MPB (plasma exosomes)  MPB (serum)  Placenta | Up in PE  No difference  Up in PE | Yes  No  Yes | 110  70  190 |
| 26b | Placenta | Up in PE | Yes | 24 |
| 26b-5p | Placenta | No difference | No | 160 |
| 25 | Placenta | Up in PE | Yes | 24 |
| 376c | Placenta  MPB (plasma)  MPB (plasma exosomes)  MPB (plasma) | Down in PE  Down in PE  Up in PE  Down in PE | Yes  Yes  Yes  Yes | 25  25  153  186 |
| 149 | Placenta  MPB (whole blood)  MPB (whole blood) | Down in PE  Down in PE 36GW  No difference 28GW | Yes  Yes  No | 26 Whigham 2020  Whigham 2020 |
| 149-5p | Placenta | Down in PE | Yes | 182 |
| 106a | Placenta  Placenta | Up in PE  Down in PE | Yes  Yes | 28  Zhao 2020 |
| 106b | MPB (mononuclear cells) | Up in PE | Yes | 155 |
| 19b | Placenta  MPB (plasma) | Up in PE  Down in PE | Yes  Yes | 28  95 |
| 125b | MPB (plasma)  Placenta (chorionic plate)  Placenta (basal plate)  MPB (plasma)  MPB (plasma) | No difference  Up in PE  Up in PE  Up in PE  Up in PE | No  Yes  Yes  Yes  Yes | 32  79  79  79  Licini, 2020 |
| 125b-5p | MPB (serum)  Urine  MPB (plasma)  MPB (plasma) | Down in PE  No difference  No difference  Up in PE | Yes  No  No  Yes | 86  86  Akgor 2020  Li Q 2020 |
| 125b-2-3p | MPB (plasma exosomes) | Down in PE | Yes | 110 |
| 146a | MPB (plasma)  MPB  MPB | No difference  No difference vs. preconceptional  No difference vs. 1^st^ trimester control | No  No  No | 32  63  63 |
| 146a-5p | MPB (whole blood)  MPB  Placenta | No difference  No difference  Up in PE | No  No  Yes | 66  158  Peng 2021 |
| 196b | MPB (plasma)  MPB (plasma)  MPB (plasma exosomes) | Up in PE  No difference  Up in PE | Yes  No  Yes | 32  48  153 |
| 181b | Placenta | Down in PE | Yes | 33 |
| 34a | Placenta  Placenta  Placenta  Placenta | Down in PE  Up in PE  Up in PE  Up in PE | Yes  Yes  Yes  Yes | 34  62  90 |
| 34a-5p | Placenta  MPB (serum) | Up in PE  Up in PE | Yes  Yes | 185  185 |
| 455-5p | Placenta  Placenta (trophoblast debris) | Down in PE  Up in PE | Yes  Yes | 36  113 |
| 455-3p | Placenta | Down in PE | Yes | 36 |
| 517a | Placenta | No difference | No | 36 |
| 1233 | MPB (serum) | Up in PE | NR | 41 |
| 1233-3p | MPB (serum) | Up in PE | Yes | 70 |
| 1233-5p | MPB (serum) | No difference | No | 70 |
| 101 | Placenta | Down in PE | Yes | 47 |
| 206 | MPB (plasma)  Placenta  Myometrium  MPB (serum)  MPB (plasma) | Up in PE  Up in PE  NR  Up in PE  Up in PE | Yes  Yes  NR  Yes  Yes | 48  48  48  167  Sheng 2020 |
| 502 | MPB (plasma) | No difference | No | 48 |
| 503 | MPB (plasma) | No difference | No | 48 |
| 758 | MPB (plasma) | No difference | No | 48 |
| 494 | Placenta (deciduas MSCs) | Up in PE | Yes | 50 |
| 499-5p | Placenta  MPB (whole blood) | Up in PE  No difference | Yes  No | 52  66 |
| 499a-5p | MPB | No difference | No | 158 |
| 519a | Placenta | Down in PE | yes | 53 |
| 519d | Placenta  Placenta | Down in PE  Down in PE | Yes  Yes | 53  Cai 2021 |
| 524-5p | Placenta | Down in PE | Yes | 53 |
| 335 | Placenta | Up in PE | Yes | 55 |
| 335-5p | MPB (plasma exosomes) | Down in PE | Yes | 110 |
| 155 | Placenta  MPB  MPB  MPB (serum)  Urine  MPB (plasma)  MPB (serum)  Placenta  MPB (serum)  MPB (serum exosomes)  MPB (serum)  MPB (serum) | Up in PE  No difference vs. preconceptional  No difference vs. 1^st^ trimester control  Up in PE  No difference  Up in PE  Up in PE  Up in PE  NR  Up in PE  Up in PE  Down in PE | Yes  No  No  Yes  No  Yes  Yes  Yes  NR  Yes  Yes  Yes | 56  63  63  86  86  95  117  117  128  140  191  Ayoub 2019 |
| 155-5p | MPB (plasma)  MPB (plasma)  MPB (serum)  MPB (leukocytes)  MPB (leukocytes)  MPB (serum)  MPB (plasma) | No difference at delivery  No difference 1 year after delivery  No difference  Down in PE  No difference  Up in PE  Up in PE | No  No  No  Yes  No  Yes  Yes | 60  60  70  157  Demirer 2019  Kim 2020  Kolkova 2021 |
| 122 | Placenta | Up in PE | Yes | 56 |
| 122-5p | MPB  Placenta | Up in PE  Up in PE | Yes  Yes | 76  160 |
| 21 | Placenta  MPB (plasma)  Placenta (chorionic plate)  Placenta (basal plate)  Placenta  MPB (serum) | Up in PE  Up in PE  Down in PE  Down in PE  Up in PE  Up in PE | Yes  Yes  Yes  Yes  Yes  Yes | 56  95  134  134  Zhou 2020  Zhou 2020 |
| 21-3p | MPB (leukocytes)  MPB (leukocytes) | Down in PE  No difference | Yes  No | 157  Demirer 2019 |
| 21-5p | MPB (plasma) | Up in PE | Yes | Kolkova 2021 |
| 152 | MPB (serum)  MPB (plasma) | Up in 1^st^, 2^nd^ and 3^rd^ trimester PE  No difference | Yes  No | 57  Akgor |
| 183 | MPB (serum) | Up in 1^st^, 2^nd^ and 3^rd^ trimester PE | Yes | 57 |
| 328 | MPB (serum)  MPB (plasma) | No difference  Down in PE | No  Yes | 57  Akgor 2020 |
| 363 | MPB (serum)  MPB (whole blood)  MPB (whole blood) | No difference  Down in PE 36GW  Down in PE 28GW | No  Yes  Yes | 57  Whigham 2020  Whigham 2020 |
| 500 | MPB (serum) | No difference | No | 57 |
| let-7f-5p | MPB (plasma)  MPB (plasma) | No difference at delivery  No difference 1 year after delivery | No  No | 60  60 |
| let-7f-1-3p | Placenta (hDMSC) | No difference in PE | No | Kamali-Simsek 2021 |
| 98-5p | MPB (plasma)  MPB (plasma) | No difference at delivery  No difference 1 year after delivery | No  No | 60  60 |
| 221-3p | MPB (plasma)  MPB (plasma)  MPB (whole blood)  MPB (plasma exosomes)  MPB  Placenta | No difference at delivery  Down 1 year after delivery  No difference  Up in PE  No difference  Down in PE | No  Yes  No  Yes  No  Yes | 60  60  66  110  158  189 |
| 221-5p | MPB  MPB | No difference vs. preconceptional  No difference vs. 1^st^ trimester control | No  No | 63  63 |
| 222 | MPB (serum) | Down in PE | Yes | 130 |
| 222-3p | MPB (plasma)  MPB (plasma) | No difference at delivery  No difference 1 year after delivery | No  No | 60  60 |
| 130a | MPB (plasma)  MPB (plasma) | No difference at delivery  No difference 1 year after delivery | No  No | 60  60 |
| 17-5p | MPB (plasma)  MPB (plasma)  MPB (whole blood)  MPB (plasma exosomes)  MPB  MPB (plasma) | No difference at delivery  No difference 1 year after delivery  No difference  Up in PE  Up in PE  Up in PE | No  No  No  Yes  Yes  Yes | 60  60  66  110  158  Akgor 2020 |
| 29a | MPB (serum) | Up in PE | Yes | 130 |
| 29a-3p | MPB (plasma)  MPB (plasma)  MPB (serum)  MPB  MPB (plasma) | No difference at delivery  No difference 1 year after delivery  No difference  No difference  Down in PE | No  No  No  No  Yes | 60  60  70  158  Akgor 2020 |
| 92-3p | MPB (plasma)  MPB (plasma) | No difference at delivery  No difference 1 year after delivery | No  No | 60  60 |
| 20b-5p | MPB (plasma)  MPB (plasma)  MPB | No difference at delivery  No difference 1 year after delivery  No difference | No  No  No | 60  60  158 |
| 20b | Placenta  Placenta  MPB | Up in PE  Up in PE  Up in PE | Yes  Yes  Yes | 67  97  97 |
| 195-5p | MPB (plasma)  MPB (plasma)  MPB (whole blood)  MPB (plasma) | No difference at delivery  No difference 1 year after delivery  No difference  Up in PE | No  No  No  Yes | 60  60  66  72 |
| 33a | MPB (buffy coat) | NR | NR | 146 |
| 33a-5p | MPB  MPB | No difference vs. preconceptional  No difference vs. 1^st^ trimester control | No  No | 63  63 |
| 7-5p | MPB  MPB | Up in PE vs. preconceptional  No difference vs. 1^st^ trimester control | Yes  No | 63  63 |
| 1229 | MPB  MPB | No difference vs. preconceptional  Down in PE vs. 1^st^ trimester control | No  Yes | 63  63 |
| 1267 | MPB  MPB  MPB (buffy coat) | No difference vs. preconceptional  Down in PE vs. 1^st^ trimester control  NR | No  Yes  NR | 63  63  146 |
| 671-3p | MPB  MPB | No difference vs. preconceptional  No difference vs. 1^st^ trimester control | No  No | 63  63 |
| 133 | MPB (serum)  MPB (serum) | Up in PE  Up in PE | Yes  Yes | 167  Zhang 2019 |
| 133a-3p | MPB | Up in PE | Yes | 158 |
| 133b | MPB  MPB  Placenta | No difference vs. preconceptional  No difference vs. 1^st^ trimester control  Down in PE | No  No  Yes | 63  63  93 |
| 582 | MPB (buffy coat) | NR | NR | 146 |
| 582-5p | MPB  MPB | Up in PE vs. preconceptional  No difference vs. 1^st^ trimester control | Yes  No | 63  63 |
| 582-3p | MPB (plasma)  Fetal cord blood (plasma)  Placenta | Down in PE  Down in PE  Down in PE | Yes  Yes  Yes | 111  111  111 |
| 30e-3p | MPB  MPB | No difference vs. preconceptional  Down in PE vs. 1^st^ trimester control | No  Yes | 63  63 |
| 199a-5p | MPB  MPB  MPB (whole blood)  MPB  Placenta | No difference vs. preconceptional  No difference vs. 1^st^ trimester control  No difference  No difference  Up in PE | No  No  No  No  Yes | 63  63  66  158  169 |
| 199a-3p | MPB (plasma exosomes) | Up in PE | Yes | 110 |
| 199b-5p | MPB  MPB | No difference vs. preconceptional  Down in PE vs. 1^st^ trimester control | No  No | 63  63 |
| 575 | MPB  MPB  MPB (buffy coat) | No difference vs. preconceptional  No difference vs. 1^st^ trimester control  NR | No  No  NR | 63  63  146 |
| 301a-3p | MPB  MPB | No difference vs. preconceptional  Down in PE vs. 1^st^ trimester control | No  Yes | 63  63 |
| 148a-3p | MPB  MPB  Placenta (chorionic plate) | Up in PE vs. preconceptional  Down in PE vs. 1^st^ trimester control  Up in PE | Yes  Yes  Yes | 63  63  81 |
| 148a | Placenta | Down in PE | Yes | 103 |
| 193-3p | MPB  MPB  Placenta (chorionic plate) | No difference vs. preconceptional  No difference vs. 1^st^ trimester control  Up in PE | No  No  Yes | 63  63  81 |
| 219-5p | MPB  MPB | No difference vs. preconceptional  No difference vs. 1^st^ trimester control | No  No | 63  63 |
| 132 | MPB  MPB  Placenta | No difference vs. preconceptional  No difference vs. 1^st^ trimester control  Down in PE | No  No  Yes | 63  63  Wang 2020 |
| 132-3p | MPB (plasma) | Down in PE | Yes | Akgor 2020 |
| 513-5p | MPB  MPB | No difference vs. preconceptional  No difference vs. 1^st^ trimester control | No  No | 63  63 |
| 1244 | MPB  MPB | No difference vs. preconceptional  No difference vs. 1^st^ trimester control | No  No | 63  63 |
| 196a | MPB  MPB  MPB (buffy coat) | No difference vs. preconceptional  No difference vs. 1^st^ trimester control  NR | No  No  NR | 63  63  146 |
| 223 | MPB  MPB | Up in PE vs. preconceptional  Down in PE vs. 1^st^ trimester control | Yes  Yes | 63  63 |
| 100-5p | MPB (whole blood) | Down in PE | Yes | 66 |
| 125-5p | MPB (whole blood) | Down in PE | Yes | 66 |
| 125a | UCB (exosomes)  MPB (plasma exosomes)  Placenta | Up in PE  Up in PE  Up in PE | Yes  Yes  Yes | Xueya 2020  Xueya 2020  Xueya 2020 |
| 125a-5p | MPB (serum)  Urine | No difference  No difference | No  No | 86  86 |
| 574-3p | MPB (whole blood) | No difference | No | 66 |
| 574-5p | MPB (serum) | Up in PE | Yes | 70 |
| 130b-3p | MPB (serum) | No difference | No | 70 |
| 130b-5p | MPB (serum) | No difference | No | 70 |
| 124-3p | MPB (serum)  Placenta | No difference  Up in PE | No  Yes | 70  Tao 2020 |
| 141 | Placenta (villi)  MPB (plasma) | Up in PE  Up in PE | Yes  Yes | 71  177 |
| 141-3p | MPB (plasma exosomes)  MPB (plasma)  Placenta | Up in PE  Up in PE  Up in PE | Yes  Yes  Yes | 110  Cao 2019  Cao 2019 |
| 141-5p | Placenta | Down in PE | Yes | 181 |
| 885-5p | MPB (plasma) | Up in PE | Yes | 73 |
| 203 | Maternal subcutaneous fat tissue endothelial cells  Placenta | Up in PE  Up in PE | NR  Yes | 75  133 |
| 203a-3p | Placental monocytes  MPB (serum exosomes) | Down in PE  Down in PE | Yes  Yes | Ma 2020  Ma 2020 |
| 31 | MPB | Down in PE | Yes | 76 |
| 31-5p | Placenta (chorionic plate)  MPB (serum)  MPB (serum)  Placenta | Up in PE  Up in PE  Up in PE  Up in PE | Yes  Yes  Yes  Yes | 81  131  Kim 2020  Li 2020 |
| 365a-3p | Placenta (chorionic plate) | Up in PE | Yes | 81 |
| 27a-5p | Placenta (chorionic plate) | Up in PE | Yes | 81 |
| 135b-5p | Placenta (chorionic plate) | Down in PE | Yes | 81 |
| 136-3p | Placenta (chorionic plate) | Down in PE | Yes | 81 |
| 218 | Placenta (trophoblast cells) | Up in PE | Yes | 85 |
| 218-5p | Placenta (chorionic plate)  Placenta (basal plate)  MPB (plasma) | Down in PE  Down in PE  MPB (plasma) | Yes  Yes  Yes | 119  119  Akgor 2020 |
| 300 | MPB (plasma) | Up in PE | Yes | 87 |
| 942 | MPB (plasma) | Up in PE | Yes | 94 |
| 215 | MPB (plasma)  Placenta | Up in PE  Up in PE | Yes  Yes | 95  Yang 2019 |
| 650 | MPB (plasma) | Up in PE | Yes | 95 |
| 495 | UC tissue  UC-MSCs | Up in PE  Up in PE | Yes  Yes | 100  100 |
| 486-3p | MPB (plasma exosomes) | Up in PE | Yes | 153 |
| 486-5p | MPB (plasma) | No difference | No | Akgor 2020 |
| 486-1-5p | MPB (plasma exosomes) | Up in PE | Yes | 107 |
| 486-2-5p | MPB (plasma exosomes) | Up in PE | Yes | 107 |
| 192-5p | MPB (plasma exosomes) | Down in PE | Yes | 110 |
| 542 | MPB (plasma exosomes) | Down in PE | Yes | 110 |
| 205-5p | MPB (plasma exosomes) | Down in PE | Yes | 110 |
| 208-3p | MPB (plasma exosomes) | Down in PE | Yes | 110 |
| 518-5p | Placenta | Up in PE | Yes | 135 |
| 518a | MPB (plasma exosomes) | Down in PE | Yes | 110 |
| 744-5p | MPB (plasma exosomes) | Up in PE | Yes | 110 |
| 6724-5p | MPB (plasma exosomes) | Up in PE | Yes | 110 |
| 346 | MPB (plasma)  Fetal cord blood (plasma)  Placenta  MPB (plasma exosomes) | Up in PE  Down in PE  Down in PE  Up in PE | Yes  Yes  Yes  Yes | 111  111  111  153 |
| 18b | Placenta  Placenta  UCMSC | Down in PE  Down in PE  Down in PE | Yes  Yes  Yes | 112  Yang 2020  Yang 2020 |
| 615-3p | Placenta (trophoblast debris) | Up in PE | Yes | 113 |
| 1247-5p | Placenta (trophoblast debris) | Up in PE | Yes | 113 |
| 370 | Placenta | Down in PE | Yes | 116 |
| 4421 | Placenta | Up in PE | Yes | 125 |
| 423-5p | MPB (plasma) | Up in PE | Yes | 128 |
| 454 | Placenta  Placenta | Down in PE  Down in PE | Yes  Yes | 142  175 |
| 362-3p | Placenta | Up in PE | Yes | 144 |
| 134 | Placenta (chorionic plate)  Placenta (basal plate)  MPB (plasma exosomes)  MPB | No difference  Up in PE  Up in PE  No difference | No  Yes  Yes  No | 148  148  153  Dong 2020 |
| 302c | MPB (plasma exosomes) | Up in PE | Yes | 153 |
| 618 | MPB (plasma exosomes) | Up in PE | Yes | 153 |
| 326 | MPB (mononuclear cells)  MPB (mononuclear cells) | Up in PE  Up in PE | Yes  Yes | 155  Zolfaghari 2021 |
| 337-3p | Placenta | Up in PE | Yes | 160 |
| 187-3p | Placenta | Up in PE | Yes | 160 |
| 628-3p | MPB (serum)  MPB (serum)  MPB (serum) | Up in PE vs. 12gw control  No difference vs. 16gw control  No difference vs. 20gw control | Yes  No  No | 168  168  168 |
| 628-5p | MPB (serum)  MPB (serum)  MPB (serum) | No difference vs. 12gw control  Up in PE vs. 16gw control  Up in PE vs. 20gw control | No  Yes  Yes | 168  168  168 |
| 510 | MPB | Up in PE | Yes | 174 |
| 429 | MPB (plasma) | No difference | No | 177 |
| 548c-5p | Placenta  MPB (serum exosomes) | Down in PE  Down in PE | Yes  Yes | 179  179 |
| 320a | Placenta  MPB (plasma) | Down in PE  Up in PE | Yes  Yes | 183 |
| 411 | MPB (plasma) | Down in PE | Yes | 186 |
| 431 | Placenta | Up in PE | Yes | 188 |
| 1304-5p | MPB (plasma) | Up in PE | Yes | 192 |
| 5002-5p | MPB (plasma) | Up in PE | Yes | 192 |
| 188-3p | MPB (plasma) | Down in PE | Yes | 192 |
| 211-5p | MPB (plasma) | Down in PE | Yes | 192 |
| Hiv1-miR-TAR-3p | MPB (plasma) | Down in PE | Yes | 192 |
| 4432 | MPB (plasma) | Down in PE | Yes | 192 |
| 4498 | MPB (plasma) | Down in PE | Yes | 192 |
| 30b | Placenta (villi) | Up in PE | Yes | Qian 2019 |
| 128 | MPB (plasma) | No difference | No | Akgor 2020 |
| 302b-3p | MPB (plasma) | Up in PE | Yes | Akgor 2020 |
| 191-5p | MPB (plasma) | Up in PE | Yes | Akgor 2020 |
| 191-3p | Placenta (hDMSC) | Up in PE | No | Kamali-Simsek 2021 |
| 375 | MPB (plasma) | Down in PE | Yes | Akgor 2020 |
| 139-5p | MPB (plasma) | No difference | No | Akgor 2020 |
| 214-3p | MPB (plasma)  MPB (serum) | No difference  Up in PE | No  Yes | Akgor 2020  Kim 2020 |
| 214-5p | Placenta  MPB (ECFC) | Up in PE  Down in PE | Yes  Yes | Gong 2020  Brodowski 2021 |
| 646 | MPB  UC blood | Up in PE  Up in PE | Yes  Yes | Dong 2020  Dong 2020 |
| 34b | MPB | Up in PE | Yes | Dong 2020 |
| 302d | MPB | Up in PE | Yes | Dong 2020 |
| 147a | MPB | No difference | No | Dong 2020 |
| 140 | MPB | No difference | No | Dong 2020 |
| 107 | MPB | No difference | No | Dong 2020 |
| 507 | Placenta | Up in PE | Yes | Li H, 2020 |
| 23b-5p | MPB (plasma) | Down in PE | Yes | Mavreli 2020 |
| 99b | MPB (plasma) | Down in PE | Yes | Mavreli 2020 |
| 655-3p | Placenta | Up in PE | Yes | Song 2020 |
| 135a | Placenta | Up in PE | Yes | Wu 2020 |
| 1270 | UCB (ECFC) | Down in PE | Yes | Brodowski 2021 |
| 2467-5p | UCB (ECFC) | Up in PE | Yes | Brodowski 2021 |
| 3177-5p | MPB (ECFC) | Up in PE | Yes | Brodowski 2021 |
| let-7b-3p | Placenta (hDMSC) | Up in PE | No | Kamali-Simsek 2021 |
| 425-3p | Placenta (hDMSC) | No difference in PE | No | Kamali-Simsek 2021 |
| 550a-5p | Placenta (hDMSC) | Up in PE | Yes | Kamali-Simsek 2021 |
| 33b-3p | Placenta (hDMSC) | Up in PE | Yes | Kamali-Simsek 2021 |
| 135-3p | Placenta | Down in PE | Yes | Zhao 2021 |
| 135-5p | Placenta | Down in PE | Yes | Zhao 2021 |
| 27b-3p | MPB (serum) | Up in PE | Yes | Zhu 2021 |

# Supplementary Figures


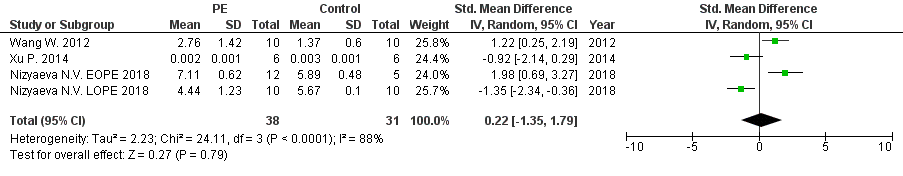


**Supplementary Figure S1.** Meta-analysis of differences in expression level of miRNA-17 in placenta between women with vs. without preeclampsia (SMD=0.22, 95%CI=-1.35-1.79, p=0.790).


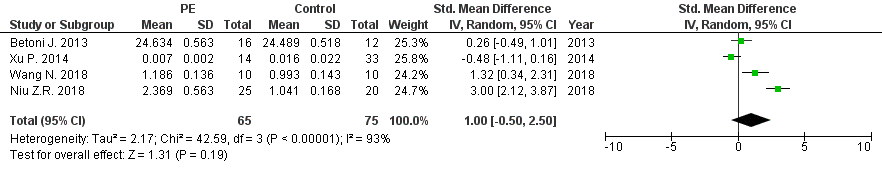


**Supplementary Figure S2.** Meta-analysis of differences in expression level of miRNA-30-3p in placenta between women with vs. without preeclampsia (SMD=1.00, 95%CI=-0.50-2.50, p=0.190).


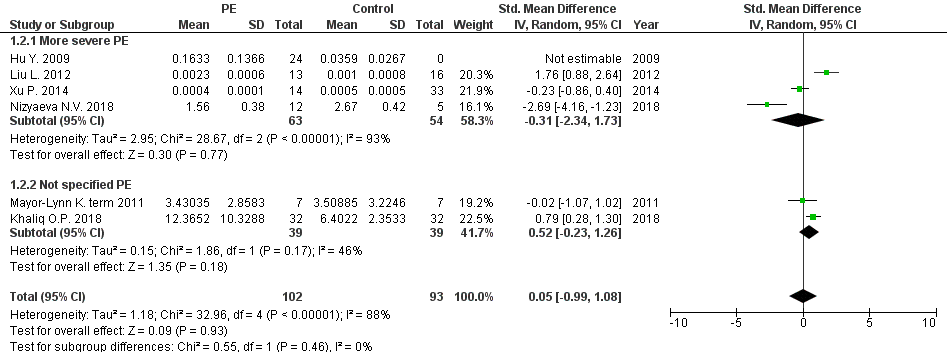


**Supplementary Figure S3.** Meta-analysis of differences in expression level of miRNA-181a in placenta between women with vs. without preeclampsia (SMD=0.05, 95%CI=-0.99-1.08, p=0.930).


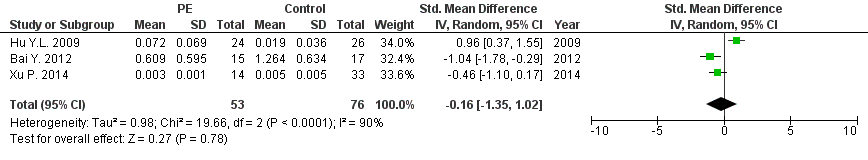


**Supplementary Figure S4.** Meta-analysis of differences in expression level of miRNA-195 in placenta between women with vs. without preeclampsia (SMD=-0.16, 95%CI=-1.35-1.02, p=0.780).


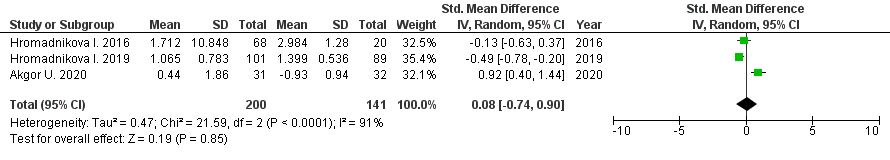


**Supplementary Figure S5.** Meta-analysis of differences in expression level of miRNA-17 in peripheral blood between women with vs. without preeclampsia (SMD=0.08, 95%CI=-0.74-0.90, p=0.850).


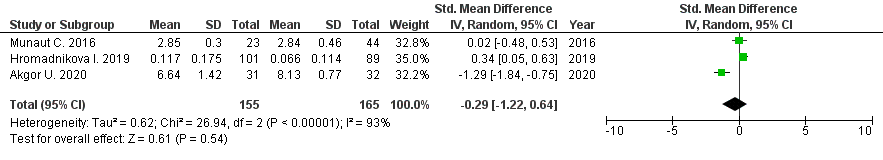


**Supplementary Figure S6.** Meta-analysis of differences in expression level of miRNA-29a-3p in peripheral blood between women with vs. without preeclampsia (SMD=-0.29, 95%CI=-1.22-0.64, p=0.540).


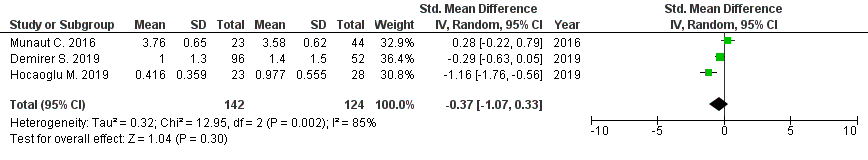


**Supplementary Figure S7.** Meta-analysis of differences in expression level of miRNA-155-5p in peripheral blood between women with vs. without preeclampsia (SMD=-0.37, 95%CI=-1.07-0.33, p=0.300).


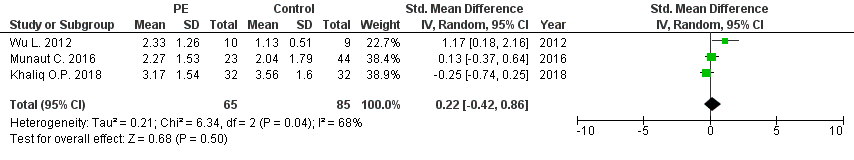


**Supplementary Figure S8.** Meta-analysis of differences in expression level of miRNA-181a in peripheral blood between women with vs. without preeclampsia (SMD=0.22, 95%CI=-0.42-0.86, p=0.500).


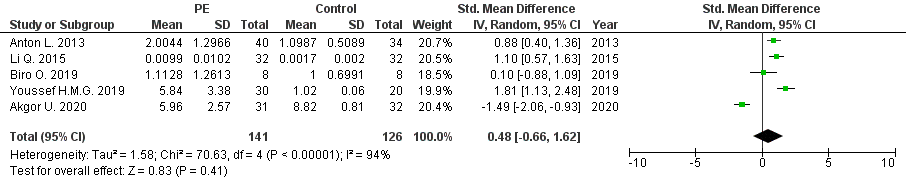


**Supplementary Figure S9.** Meta-analysis of differences in expression level of miRNA-210 in peripheral blood between women with vs. without preeclampsia (SMD=0.48, 95%CI=-0.66-1.62, p=0.410).


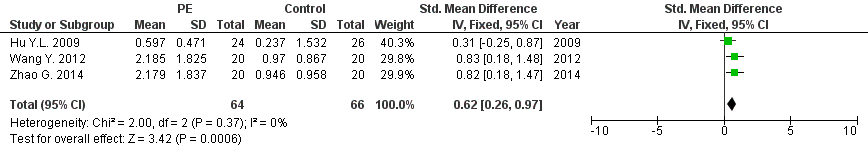


**Supplementary Figure S10.** Meta-analysis of differences in expression level of miRNA-16 in placenta between women with vs. without preeclampsia (sensitivity analysis). Sensitivity analysis after excluding study with not-specified type of PE showed the same result as the first analysis (SMD=0.62 (95%CI=0.26-0.97); p=0.001).

**
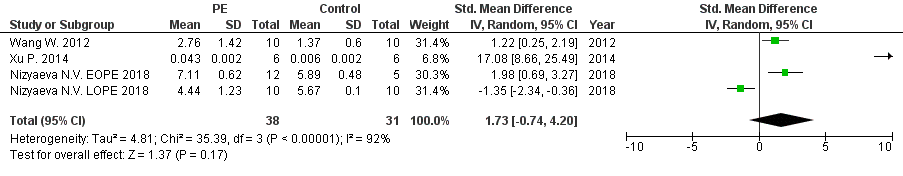
**

**Supplementary Figure S11.** Meta-analysis of differences in expression level of miRNA-17 in placenta between women with vs. without preeclampsia (sensitivity analysis). Sensitivity analysis after exchanging expression data obtained from chorionic plate with data obtained from basal plate, showed the same result as the first analysis (SMD=1.73 (95%CI=-0.74-4.20); p=0.170).


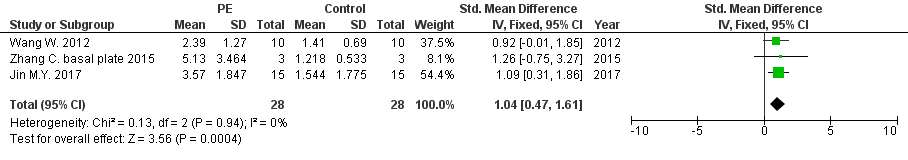


**Supplementary Figure S12.** Meta-analysis of differences in expression level of miRNA-20b in placenta between women with vs. without preeclampsia (sensitivity analysis). Sensitivity analysis after exchanging expression data obtained from chorionic plate with data obtained from basal plate, showed the same result as the first analysis (SMD=1.04 (95%CI=0.47-1.61); p<0.001).


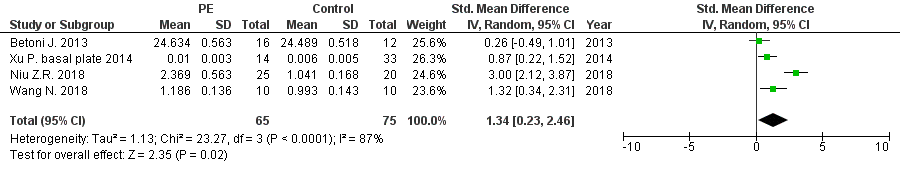


**Supplementary Figure S13.** Meta-analysis of differences in expression level of miRNA-30a-3p in placenta between women with vs. without preeclampsia (sensitivity analysis). Sensitivity analysis after exchanging expression data obtained from chorionic plate with data obtained from basal plate, showed the same result as the first analysis (SMD=1.34 (95%CI=0.23-2.46); p=0.020).


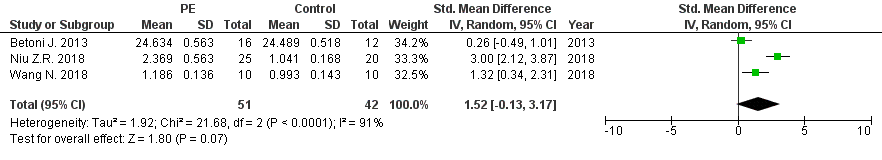


**Supplementary Figure S14.** Meta-analysis of differences in expression level of miRNA-30a-3p in placenta between women with vs. without preeclampsia (sensitivity analysis). Sensitivity analysis was performed by excluding study analyzing more severe form of PE. In this situation result changed. The expression level of miRNK-30a-3pin placenta in women with not-specified form of PE wasn`t significantly different (SMD=1.52 (95%CI=-0.13-3.17); p=0.070)compared to women without PE.


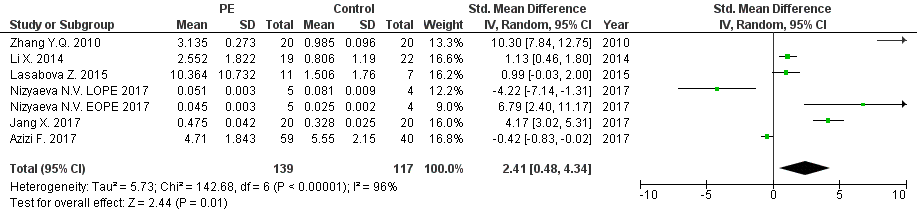


**Supplementary Figure S15.** Meta-analysis of differences in expression level of miRNA-155 in placenta between women with vs. without preeclampsia (sensitivity analysis). Sensitivity analysis after including data for women with PE and mild proteinuria (instead of severe proteinuria in first analysis) showed the same result as the first analysis (SMD=2.65 (95%CI=0.60-4.70); p=0.010).


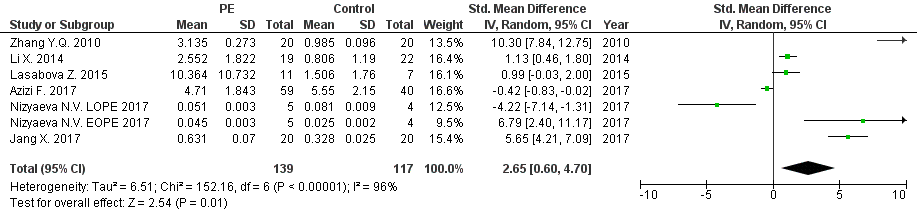


**Supplementary Figure S16.** Meta-analysis of differences in expression level of miRNA-155 in placenta between women with vs. without preeclampsia (sensitivity analysis). Sensitivity analysis after including data for women with PE and moderate proteinuria (instead of severe proteinuria in first analysis) showed the same result as the first analysis (SMD=2.41 (95%CI=0.48-4.34); p=0.010).


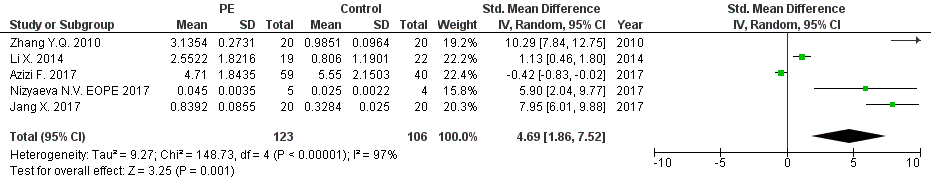


**Supplementary Figure S17.** Meta-analysis of differences in expression level of miRNA-155 in placenta between women with vs. without preeclampsia (sensitivity analysis). significantly higher expression level of miRNA-155 in placenta was in sensitivity analysis after excluding studies evaluating LOPE and not-specified form of PE (SMD=4.69 (95%CI=1.86-7.52); p=0.001).


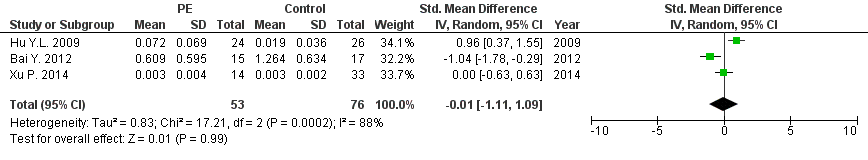


**Supplementary Figure S18.** Meta-analysis of differences in expression level of miRNA-195 in placenta between women with vs. without preeclampsia (sensitivity analysis). Sensitivity analysis after exchanging expression data obtained from chorionic plate with data obtained from basal plate, showed the same result as the first analysis (SMD=-0.01 (95%CI=1.11-1.09); p=0.990).


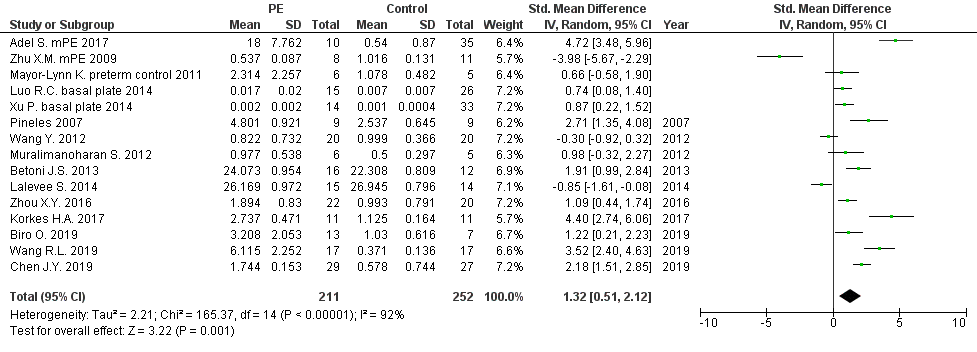


**Supplementary Figure S19.** Meta-analysis of differences in expression level of miRNA-210 in placenta between women with vs. without preeclampsia (sensitivity analysis). Sensitivity analysis after exchanging expression data obtained from chorionic plate with data obtained from basal plate, after using data for preterm instead for term controls , and after using mPE instead of sPE data showed the same result as the first analysis (SMD=1.32 (95%CI=0.51-2.12); p=0.001).


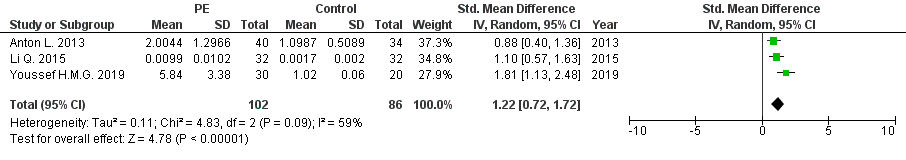


**Supplementary Figure S20.** Meta-analysis of differences in expression level of miRNA-210 in peripheral blood between women with vs. without preeclampsia (sensitivity analysis). Sensitivity analysis after excluding study that analyzed more severe form of PE showed the same result as the first analysis (SMD=1.22 (95%CI=0.72-1.72); p=0.001).


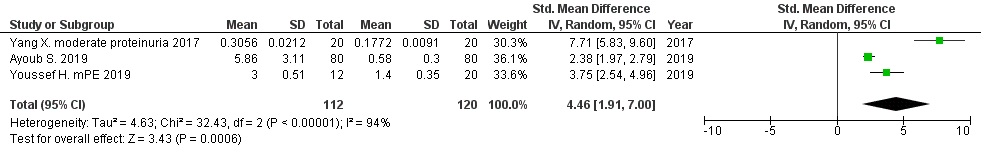


**Supplementary Figure S21.** Meta-analysis of differences in expression level of miRNA-155 in maternal peripheral blood between women with vs. without preeclampsia (sensitivity analysis). Sensitivity analysis after including data for women with PE and moderate proteinuria (instead of mild proteinuria in first analysis) (SMD=4.46 (95%CI=1.91-7.00); p=0<0.001).


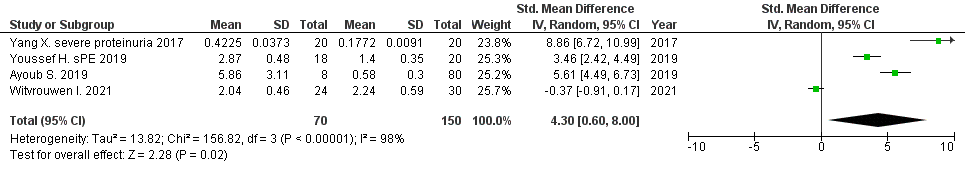


**Supplementary Figure S22.** Meta-analysis of differences in expression level of miRNA-155 in maternal peripheral blood between women with vs. without preeclampsia (sensitivity analysis). Sensitivity analysis after including data for women with PE and severe proteinuria (instead of mild proteinuria in first analysis) (SMD=4.30 (95%CI=0.60-8.00); p=0.020).

References

Adel, S., Mansour, A., Louka, M., Matboli, M., Elmekkawi, S. F., and Swelam, N. (2017). Evaluation of MicroRNA-210 and Protein tyrosine phosphatase, non-receptor type 2 in Pre-eclampsia. *Gene* 596, 105–109. doi:10.1016/j.gene.2016.10.014.

Akehurst, C., Small, H. Y., Sharafetdinova, L., Forrest, R., Beattie, W., Brown, C. E., et al. (2015). Differential expression of microRNA-206 and its target genes in preeclampsia. *J. Hypertens.* 33, 2068–2074. doi:10.1097/HJH.0000000000000656.

Ali, Z., Zafar, U., Zaki, S., Ahmad, S., Khaliq, S., and Lone, K. P. (2021). Expression levels of MiRNA-16, SURVIVIN and TP53 in Preeclamptic and Normotensive women. *J. Pak. Med. Assoc.* 71, 2208–2213. doi:10.47391/JPMA.1171.

Anton, L., Olarerin-George, A. O., Hogenesch, J. B., and Elovitz, M. A. (2015). Placental expression of miR-517a/b and miR-517c contributes to trophoblast dysfunction and preeclampsia. *PLoS One* 10, e0122707. doi:10.1371/journal.pone.0122707.

Anton, L., Olarerin-George, A. O., Schwartz, N., Srinivas, S., Bastek, J., Hogenesch, J. B., et al. (2013). MiR-210 inhibits trophoblast invasion and is a serum biomarker for preeclampsia. *Am. J. Pathol.* 183, 1437–1445. doi:10.1016/j.ajpath.2013.07.021.

Awamleh, Z., Gloor, G. B., and Han, V. K. M. (2019). Placental microRNAs in pregnancies with early onset intrauterine growth restriction and preeclampsia: Potential impact on gene expression and pathophysiology. *BMC Med. Genomics* 12, 91. doi:10.1186/s12920-019-0548-x.

Azizi, F., Saleh Gargari, S., Shahmirzadi, S. A., Dodange, F., Amiri, V., Mirfakhraie, R., et al. (2017). Evaluation of Placental mir-155-5p and Long Non-coding RNA sONE Expression in Patients with Severe Pre-eclampsia. *Int J Mol Cell Med* 6, 22–30.

Bai, Y., Yang, W., Yang, H. xia, Liao, Q., Ye, G., Fu, G., et al. (2012). Downregulated miR-195 detected in Preeclamptic Placenta affects trophoblast cell invasion via modulating ActRIIA expression. *PLoS One* 7, e38875. doi:10.1371/journal.pone.0038875.

Betoni, J. S., Derr, K., Pahl, M. C., Rogers, L., Muller, C. L., Packard, R. E., et al. (2013). MicroRNA analysis in placentas from patients with preeclampsia: Comparison of new and published results. *Hypertens. Pregnancy* 32, 321–339. doi:10.3109/10641955.2013.807819.

Biró, O., Fóthi, Á., Alasztics, B., Nagy, B., Orbán, T. I., and Rigó, J. (2019). Circulating exosomal and Argonaute-bound microRNAs in preeclampsia. *Gene* 692, 138–144. doi:10.1016/j.gene.2019.01.012.

Brkić, J., Dunk, C., O’Brien, J., Fu, G., Nadeem, L., Wang, Y. ling, et al. (2018). MicroRNA-218-5p Promotes Endovascular Trophoblast Differentiation and Spiral Artery Remodeling. *Mol. Ther.* 26, 2189–2205. doi:10.1016/j.ymthe.2018.07.009.

Campos, C. B., Marques, T. M., Pereira, R. W., and Sandrim, V. C. (2014). Reduced circulating miR-196b levels is associated with preeclampsia. *Pregnancy Hypertens.* 4, 11–13. doi:10.1016/j.preghy.2013.10.002.

Chen, J., Zhao, L., Wang, D., Xu, Y., Gao, H., Tan, W., et al. (2019). Contribution of regulatory T cells to immune tolerance and association of microRNA-210 and Foxp3 in preeclampsia. *Mol. Med. Rep.* 19, 1150–1158. doi:10.3892/mmr.2018.9733.

Chen, S., Zhao, G., Miao, H., Tang, R., Song, Y., Hu, Y., et al. (2015). MicroRNA-494 inhibits the growth and angiogenesis-regulating potential of mesenchymal stem cells. *FEBS Lett.* 589, 710–717. doi:10.1016/j.febslet.2015.01.038.

Chen, Y. S., Shen, L., Mai, R. Q., and Wang, Y. (2014). Levels of microRNA‑181b and plasminogen activator inhibitor‑1 are associated with hypertensive disorders complicating pregnancy. *Exp. Ther. Med.* 8, 1523–1527. doi:10.3892/etm.2014.1946.

Cheng, W., Liu, T., Jiang, F., Liu, C., Zhao, X., Gao, Y., et al. (2011). microRNA-155 regulates angiotensin II type 1 receptor expression in umbilical vein endothelial cells from severely pre-eclamptic pregnant women. *Int. J. Mol. Med.* 27, 393–399. doi:10.3892/ijmm.2011.598.

Chi, Z., and Zhang, M. (2018). Exploration of the regulation and control mechanisms of mir-145 in trophoblast cell proliferation and invasion. *Exp. Ther. Med.* 16, 5298–5304. doi:10.3892/etm.2018.6890.

Choi, S. Y., Yun, J., Lee, O. J., Han, H. S., Yeo, M. K., Lee, M. A., et al. (2013). MicroRNA expression profiles in placenta with severe preeclampsia using a PNA-based microarray. *Placenta* 34, 799–804. doi:10.1016/j.placenta.2013.06.006.

Dai, X., and Cai, Y. (2018). Down-regulation of microRNA let-7d inhibits the proliferation and invasion of trophoblast cells in preeclampsia. *J. Cell. Biochem.* 119, 1141–1151. doi:10.1002/jcb.26282.

Demirer, S., Hocaoglu, M., Turgut, A., Karateke, A., and Komurcu-Bayrak, E. (2020). Expression profiles of candidate microRNAs in the peripheral blood leukocytes of patients with early- and late-onset preeclampsia versus normal pregnancies. *Pregnancy Hypertens.* 19, 239–245. doi:10.1016/j.preghy.2019.11.003.

Devor, E., Santillan, D., Scroggins, S., Warrier, A., and Santillan, M. (2020). Trimester-specific plasma exosome microRNA expression profiles in preeclampsia. *J. Matern. Neonatal Med.* 33, 3116–3124. doi:10.1080/14767058.2019.1569614.

Ding, J., Huang, F., Wu, G., Han, T., Xu, F., Weng, D., et al. (2015). MiR-519d-3p suppresses invasion and migration of trophoblast cells via targeting MMP-2. *PLoS One* 10, e0120321. doi:10.1371/journal.pone.0120321.

Dong, K., Zhang, X., Ma, L., Gao, N., Tang, H., Jian, F., et al. (2019). Downregulations of circulating miR-31 and miR-21 are associated with preeclampsia. *Pregnancy Hypertens.* 17, 59–63. doi:10.1016/j.preghy.2019.05.013.

Doridot, L., Houry, D., Gaillard, H., Chelbi, S. T., Barbaux, S., and Vaiman, D. (2014). miR-34A expression, epigenetic regulation, and function in human placental diseases. *Epigenetics* 9, 142–151. doi:10.4161/epi.26196.

Eghbal-Fard, S., Yousefi, M., Heydarlou, H., Ahmadi, M., Taghavi, S., Movasaghpour, A., et al. (2019). The imbalance of Th17/Treg axis involved in the pathogenesis of preeclampsia. *J. Cell. Physiol.* 234, 5106–5116. doi:10.1002/jcp.27315.

Enquobahrie, D. A., Abetew, D. F., Sorensen, T. K., Willoughby, D., Chidambaram, K., and Williams, M. A. (2011). Placental microRNA expression in pregnancies complicated by preeclampsia. *Am. J. Obstet. Gynecol.* 204, 178.e12-178.e21. doi:10.1016/j.ajog.2010.09.004.

Fang, M., Du, H., Han, B., Xia, G., Shi, X., Zhang, F., et al. (2017). Hypoxia-inducible microRNA-218 inhibits trophoblast invasion by targeting LASP1: Implications for preeclampsia development. *Int. J. Biochem. Cell Biol.* 87, 95–103. doi:10.1016/j.biocel.2017.04.005.

Fang, Y., Huang, Z., Tan, W., Zhang, Q., L, W., and Wu, J. (2018). High expression of miR-182-5p promotes preeclampsia progression. *Eur. Rev. Med. Pharmacol. Sci.* 22, 6583–6590. doi:10.26355/eurrev_201810_16132.

Fu, G., Ye, G., Nadeem, L., Ji, L., Manchanda, T., Wang, Y., et al. (2013). MicroRNA-376c impairs transforming growth factor-β and nodal signaling to promote trophoblast cell proliferation and invasion. *Hypertens. (Dallas, Tex. 1979)* 61, 864–872. doi:10.1161/HYPERTENSIONAHA.111.203489.

Gan, L., Liu, Z., Wei, M., Chen, Y., Yang, X., Chen, L., et al. (2017). MIR-210 and miR-155 as potential diagnostic markers for pre-eclampsia pregnancies. *Med. (United States)* 96, e7515. doi:10.1097/MD.0000000000007515.

Gao, S., Wang, Y., Han, S., and Zhang, Q. (2017). Up-regulated microRNA-300 in maternal whole peripheral blood and placenta associated with pregnancy-induced hypertension and preeclampsia. *Int J Clin Exp Pathol* 10, 4232–4242.

Gao, X., Li, H., and Wei, J.-X. (2018a). MiR-4421 regulates the progression of preeclampsia by regulating CYP11B2. *Eur Rev Med Pharmacol Sci* 22, 1533–1540. doi:10.26355/eurrev_201803_14557.

Gao, Y., She, R., Wang, Q., Li, Y., and Zhang, H. (2018b). Up-regulation of miR-299 suppressed the invasion and migration of HTR-8/SVneo trophoblast cells partly via targeting HDAC2 in pre-eclampsia. *Biomed. Pharmacother.* 97, 1222–1228. doi:10.1016/j.biopha.2017.11.053.

Gunel, T., Hosseini, M. K., Gumusoglu, E., Kisakesen, H. I., Benian, A., and Aydinli, K. (2017). Expression profiling of maternal plasma and placenta microRNAs in preeclamptic pregnancies by microarray technology. *Placenta* 52, 77–85. doi:10.1016/j.placenta.2017.02.019.

Gunel, T., Kamali, N., Hosseini, M. K., Gumusoglu, E., Benian, A., and Aydinli, K. (2020). Regulatory effect of miR-195 in the placental dysfunction of preeclampsia. *J. Matern. Neonatal Med.* 33, 901–908. doi:10.1080/14767058.2018.1508439.

Gunel, T., Zeybek, Y. G., Akçakaya, P., Kalelioğlu, I., Benian, A., Ermis, H., et al. (2011). Serum microRNA expression in pregnancies with preeclampsia. *Genet. Mol. Res.* 10, 4034–4040. doi:10.4238/2011.November.8.5.

Guo, L., Liu, Y., Guo, Y., Yang, Y., and Chen, B. (2018). MicroRNA-423–5p inhibits the progression of trophoblast cells via targeting IGF2BP1. *Placenta* 74, 1–8. doi:10.1016/j.placenta.2018.12.003.

Guo, L., Tsai, S. Q., Hardison, N. E., James, A. H., Motsinger-Reif, A. A., Thames, B., et al. (2013). Differentially expressed microRNAs and affected biological pathways revealed by modulated modularity clustering (MMC) analysis of human preeclamptic and IUGR placentas. *Placenta* 34, 599–605. doi:10.1016/j.placenta.2013.04.007.

Guo, L., Yang, Q., Lu, J., Li, H., Ge, Q., Gu, W., et al. (2011). A comprehensive survey of miRNA repertoire and 3′ addition events in the placentas of patients with pre-eclampsia from high-throughput sequencing. *PLoS One* 6, e21072. doi:10.1371/journal.pone.0021072.

Guo, M., Zhao, X., Yuan, X., and Li, P. (2017). Elevated microRNA-34a contributes to trophoblast cell apoptosis in preeclampsia by targeting BCL-2. *J. Hum. Hypertens.* 31, 815–820. doi:10.1038/jhh.2017.65.

Han, L., Zhao, Y., Luo, Q. Q., Liu, X. X., Lu, S. S., and Zou, L. (2017). The significance of miR-145 in the prediction of preeclampsia. *Bratislava Med. J.* 118, 523–528. doi:10.4149/BLL_2017_101.

Hayder, H., Fu, G., Nadeem, L., O’brien, J. A., Lye, S. J., and Peng, C. (2021). Overexpression of mir-210-3p impairs extravillous trophoblast functions associated with uterine spiral artery remodeling. *Int. J. Mol. Sci.* 22, 3961. doi:10.3390/ijms22083961.

Hocaoglu, M., Demirer, S., Senturk, H., Turgut, A., and Komurcu-Bayrak, E. (2019). Differential expression of candidate circulating microRNAs in maternal blood leukocytes of the patients with preeclampsia and gestational diabetes mellitus. *Pregnancy Hypertens.* 17, 5–11. doi:10.1016/j.preghy.2019.04.004.

Hong, F., Li, Y., and Xu, Y. (2014). Decreased placental miR-126 expression and vascular endothelial growth factor levels in patients with pre-eclampsia. *J. Int. Med. Res.* 42, 1243–1251. doi:10.1177/0300060514540627.

Hromadnikova, I., Dvorakova, L., Kotlabova, K., and Krofta, L. (2019a). The prediction of gestational hypertension, preeclampsia and fetal growth restriction via the first trimester screening of plasma exosomal C19MC microRNAs. *Int. J. Mol. Sci.* 20, 2972. doi:10.3390/ijms20122972.

Hromadnikova, I., Kotlabova, K., Doucha, J., Dlouha, K., and Krofta, L. (2012). Absolute and relative quantification of placenta-specific microRNAs in maternal circulation with placental insufficiency - Related complications. *J. Mol. Diagnostics* 14, 160–167. doi:10.1016/j.jmoldx.2011.11.003.

Hromadnikova, I., Kotlabova, K., Dvorakova, L., and Krofta, L. (2019b). Postpartum profiling of microRNAs involved in pathogenesis of cardiovascular/cerebrovascular diseases in women exposed to pregnancy-related complications. *Int. J. Cardiol.* 291, 158–167. doi:10.1016/j.ijcard.2019.05.036.

Hromadnikova, I., Kotlabova, K., Hympanova, L., and Krofta, L. (2015a). Cardiovascular and cerebrovascular disease associated microRNAS are dysregulated in placental tissues affected with gestational hypertension, preeclampsia and intrauterine growth restriction. *PLoS One* 10, e0138383. doi:10.1371/journal.pone.0138383.

Hromadnikova, I., Kotlabova, K., Hympanova, L., and Krofta, L. (2016). Gestational hypertension, preeclampsia and intrauterine growth restriction induce dysregulation of cardiovascular and cerebrovascular disease associated microRNAs in maternal whole peripheral blood. *Thromb. Res.* 137, 126–140. doi:10.1016/j.thromres.2015.11.032.

Hromadnikova, I., Kotlabova, K., Ivankova, K., Vedmetskaya, Y., and Krofta, L. (2017). Profiling of cardiovascular and cerebrovascular disease associated microRNA expression in umbilical cord blood in gestational hypertension, preeclampsia and fetal growth restriction. *Int. J. Cardiol.* 249, 402–409. doi:10.1016/j.ijcard.2017.07.045.

Hromadnikova, I., Kotlabova, K., Ondrackova, M., Kestlerova, A., Novotna, V., Hympanova, L., et al. (2013). Circulating C19MC MicroRNAs in preeclampsia, gestational hypertension, and fetal growth restriction. *Mediators Inflamm.* 2013, 186041. doi:10.1155/2013/186041.

Hromadnikova, I., Kotlabova, K., Ondrackova, M., Pirkova, P., Kestlerova, A., Novotna, V., et al. (2015b). Expression profile of C19MC microRNAs in placental tissue in pregnancy-related complications. *DNA Cell Biol.* 34, 437–457. doi:10.1089/dna.2014.2687.

Hu, E., Ding, L., Miao, H., Liu, F., Liu, D., Dou, H., et al. (2015). MiR-30a attenuates immunosuppressive functions of IL-1β-elicited mesenchymal stem cells via targeting TAB3. *FEBS Lett.* 589, 3899–3907. doi:10.1016/j.febslet.2015.11.001.

Hu, S., Li, J., Tung, M., Li, Q., Chen, Y., Lu, H., et al. (2019). MicroRNA-144-3p may participate in the pathogenesis of preeclampsia by targeting Cox-2. *Mol. Med. Rep.* 19, 4655–4662. doi:10.3892/mmr.2019.10150.

Hu, T. X., Guo, X., Wang, G., Gao, L., He, P., Xia, Y., et al. (2017). MiR133b is involved in endogenous hydrogen sulfide suppression of sFlt-1 production in human placenta. *Placenta* 52, 33–40. doi:10.1016/j.placenta.2017.02.012.

Hu, T. X., Wang, G., Guo, X. J., Sun, Q. Q., He, P., Gu, H., et al. (2016). MiR 20a,-20b and -200c are involved in hydrogen sulfide stimulation of VEGF production in human placental trophoblasts. *Placenta* 39, 101–110. doi:10.1016/j.placenta.2016.01.019.

Hu, Y., Li, P., Hao, S., Liu, L., Zhao, J., and Hou, Y. (2009). Differential expression of microRNAs in the placentae of Chinese patients with severe pre-eclampsia. *Clin. Chem. Lab. Med.* 47, 923–929. doi:10.1515/CCLM.2009.228.

Huang, J., Zheng, L., Kong, H., Wang, F., Su, Y., and Xin, H. (2020). miR-139-5p promotes the proliferation and invasion of trophoblast cells by targeting sFlt-1 in preeclampsia. *Placenta* 92, 37–43. doi:10.1016/j.placenta.2020.02.003.

Huang, X., Wu, L., Zhang, G., Tang, R., and Zhou, X. (2019). Elevated MicroRNA-181a-5p Contributes to Trophoblast Dysfunction and Preeclampsia. *Reprod. Sci.* 26, 1121–1129. doi:10.1177/1933719118808916.

Jairajpuri, D. S., Malalla, Z. H., Mahmood, N., and Almawi, W. Y. (2017). Circulating microRNA expression as predictor of preeclampsia and its severity. *Gene* 627, 543–548. doi:10.1016/j.gene.2017.07.010.

Jairajpuri, D. S., Malalla, Z. H., Sarray, S., and Mahmood, N. (2021). Analysis of differential expression of hypoxia-inducible microRNA-210 gene targets in mild and severe preeclamptic patients. *Non-coding RNA Res.* 6, 51–57. doi:10.1016/j.ncrna.2021.03.001.

Jelena, M., Sopić, M., Joksić, I., Zmrzljak, U. P., Karadžov-Orlić, N., Košir, R., et al. (2020). Placenta-specific plasma miR518b is a potential biomarker for preeclampsia. *Clin. Biochem.* 79, 28–33. doi:10.1016/j.clinbiochem.2020.02.012.

Jiang, F., Li, J., Wu, G., Miao, Z., Lu, L., Ren, G., et al. (2015). Upregulation of microRNA-335 and microRNA-584 contributes to the pathogenesis of severe preeclampsia through downregulation of endothelial nitric oxide synthase. *Mol. Med. Rep.* 12, 5383–5390. doi:10.3892/mmr.2015.4018.

Jiang, L., Long, A., Tan, L., Hong, M., Wu, J., Cai, L., et al. (2017). Elevated microRNA-520g in pre-eclampsia inhibits migration and invasion of trophoblasts. *Placenta* 51, 70–75. doi:10.1016/j.placenta.2017.02.001.

Jin, M., Li, H., Xu, H., Huo, G., and Yao, Y. (2017). MicroRNA-20b inhibits trophoblast cell migration and invasion by targeting MMP-2. *Int J Clin Exp Pathol* 10, 10901–10909.

Khaliq, O. P., Murugesan, S., Moodley, J., and Mackraj, I. (2018). Differential expression of miRNAs are associated with the insulin signaling pathway in preeclampsia and gestational hypertension. *Clin. Exp. Hypertens.* 40, 744–751. doi:10.1080/10641963.2018.1431257.

Kim, S., Lee, K. S., Choi, S., Kim, J., Lee, D. K., Park, M., et al. (2018). NF-B–responsive miRNA-31-5p elicits endothelial dysfunction associated with preeclampsia via downregulation of endothelial nitric-oxide synthase. *J. Biol. Chem.* 293, 18989–19000. doi:10.1074/jbc.RA118.005197.

Korkes, H. A., De Oliveira, L., Sass, N., Salahuddin, S., Karumanchi, S. A., and Rajakumar, A. (2017). Relationship between hypoxia and downstream pathogenic pathways in preeclampsia. *Hypertens. Pregnancy* 36, 145–150. doi:10.1080/10641955.2016.1259627.

Kumar, P., Luo, Y., Tudela, C., Alexander, J. M., and Mendelson, C. R. (2013). The c-Myc-Regulated MicroRNA-17∼92 (miR-17∼92) and miR-106a∼363 Clusters Target hCYP19A1 and hGCM1 To Inhibit Human Trophoblast Differentiation. *Mol. Cell. Biol.* 33, 1782–1796. doi:10.1128/MCB.01228-12.

Lalevée, S., Lapaire, O., and Bühler, M. (2014). MiR455 is linked to hypoxia signaling and is deregulated in preeclampsia. *Cell Death Dis.* 5, e1408. doi:10.1038/cddis.2014.368.

Lasabová, Z., Vážan, M., Zibolenová, J., Švecová, I., and Lasabová, A. Z. (2015). Overexpression of miR-21 and miR-122 in preeclamptic placentas. *Neuroendocr. Lett* 36, 26859593–360715.

Lázár, L., Nagy, B., Molvarec, A., Szarka, A., and Rigó, J. (2012). Role of hsa-miR-325 in the etiopathology of preeclampsia. *Mol. Med. Rep.* 6, 597–600. doi:10.3892/mmr.2012.954.

Li, H., Ge, Q., Guo, L., and Lu, Z. (2013a). Maternal plasma miRNAs expression in preeclamptic pregnancies. *Biomed Res. Int.* 2013, 970265. doi:10.1155/2013/970265.

Li, J., Du, J., Wang, Z., Wang, C., Bai, J., and Zhang, S. (2018). Expression of miR‑376 in blood of pregnant women with preeclampsia and its effect on 25‑hydroxyvitamin D. *Exp. Ther. Med.* 16, 1701–1706. doi:10.3892/etm.2018.6394.

Li, L., Hou, A., Gao, X., Zhang, J., Zhang, L., Wang, J., et al. (2017a). Lentivirus-mediated miR-23a overexpression induces trophoblast cell apoptosis through inhibiting X-linked inhibitor of apoptosis. *Biomed. Pharmacother.* 94, 412–417. doi:10.1016/j.biopha.2017.07.082.

Li, P., Guo, W., Du, L., Zhao, J., Wang, Y., Liu, L., et al. (2013b). MicroRNA-29b contributes to pre-eclampsia through its effects on apoptosis, invasion and angiogenesis of trophoblast cells. *Clin. Sci.* 124, 27–40. doi:10.1042/CS20120121.

LI, Q., LONG, A., JIANG, L., CAI, L., XIE, L., GU, J., et al. (2015). Quantification of preeclampsia-related microRNAs in maternal serum. *Biomed. Reports* 3, 792–796. doi:10.3892/br.2015.524.

Li, Q., Pan, Z., Wang, X., Gao, Z., Ren, C., and Yang, W. (2014a). MiR-125b-1-3p inhibits trophoblast cell invasion by targeting sphingosine-1-phosphate receptor 1 in preeclampsia. *Biochem. Biophys. Res. Commun.* 453, 57–63. doi:10.1016/j.bbrc.2014.09.059.

Li, R., Wang, N., Xue, M., Long, W., Cheng, C., Mi, C., et al. (2019). A potential regulatory network among WDR86-AS1, miR-10b-3p, and LITAF is possibly involved in preeclampsia pathogenesis. *Cell. Signal.* 55, 40–52. doi:10.1016/j.cellsig.2018.12.006.

Li, T., Zhou, B., He, Y., Liu, J., and Li, Y. (2020). Expression and clinical diagnostic value of miR-383 in patients with severe preeclampsia. *Cell. Mol. Biol.* 66, 92–100. doi:10.14715/cmb/2020.66.3.14.

Li, X., Li, C., Dong, X., and Gou, W. (2014b). MicroRNA-155 inhibits migration of trophoblast cells and contributes to the pathogenesis of severe preeclampsia by regulating endothelial nitric oxide synthase. *Mol. Med. Rep.* 10, 550–554. doi:10.3892/mmr.2014.2214.

Li, X., Song, Y., Liu, D., Zhao, J., Xu, J., Ren, J., et al. (2017b). MiR-495 Promotes Senescence of Mesenchymal Stem Cells by Targeting Bmi-1. *Cell. Physiol. Biochem.* 42, 780–796. doi:10.1159/000478069.

Lip, S. V., Boekschoten, M. V., Hooiveld, G. J., van Pampus, M. G., Scherjon, S. A., Plösch, T., et al. (2020). Early-onset preeclampsia, plasma microRNAs, and endothelial cell function. *Am. J. Obstet. Gynecol.* 222, 497.e1-497.e12. doi:10.1016/j.ajog.2019.11.1286.

Liu, B., Liu, L., Cui, S., Qi, Y., and Wang, T. (2021). Expression and significance of microRNA-126 and VCAM-1 in placental tissues of women with early-onset preeclampsia. *J. Obstet. Gynaecol. Res.* 47, 2042–2050. doi:10.1111/jog.14732.

Liu, E., Liu, Z., Zhou, Y., Chen, M., Wang, L., and Li, J. (2019a). MicroRNA‑142‑3p inhibits trophoblast cell migration and invasion by disrupting the TGF‑β1/Smad3 signaling pathway. *Mol. Med. Rep.* 49, 3775–3782. doi:10.3892/mmr.2019.9997.

Liu, F., Wu, K., Wu, W., Chen, Y., Wu, H., Wang, H., et al. (2018). MiR-203 contributes to pre-eclampsia via inhibition of VEGFA expression. *Mol. Med. Rep.* 17, 5627–5634. doi:10.3892/mmr.2018.8558.

Liu, J., Zhang, L., Zhang, F., Luan, T., Yin, Z., Rui, C., et al. (2019b). Influence of miR-34a on preeclampsia through the Notch signaling pathway. *Eur Rev Med Pharmacol Sci* 23, 923–931. doi:10.26355/eurrev_201902_16978.

Liu, L., Wang, Y., Fan, H., Zhao, X., Liu, D., Hu, Y., et al. (2012). MicroRNA-181a regulates local immune balance by inhibiting proliferation and immunosuppressive properties of mesenchymal stem cells. *Stem Cells* 30, 1756–1770. doi:10.1002/stem.1156.

Liu, Z., Zhao, X., Shan, H. Y., Gao, H., and Wang, P. (2019c). microRNA-520c-3p suppresses NLRP3 inflammasome activation and inflammatory cascade in preeclampsia by downregulating NLRP3. *Inflamm. Res.* 68, 643–654. doi:10.1007/s00011-019-01246-8.

Lou, C.-X., Zhou, X.-T., Tian, Q.-C., Xie, H.-Q., and Zhang, J.-Y. (2018). Low expression of microRNA-21 inhibits trophoblast cell infiltration through targeting PTEN. *Eur Rev Med Pharmacol Sci* 22, 6181–6189. doi:10.26355/eurrev_201810_16023.

Lu, T. M., Lu, W., and Zhao, L. J. (2017). MicroRNA-137 Affects Proliferation and Migration of Placenta Trophoblast Cells in Preeclampsia by Targeting ERRα. *Reprod. Sci.* 24, 85–96. doi:10.1177/1933719116650754.

Luizon, M. R., Conceição, I. M. C. A., Viana-Mattioli, S., Caldeira-Dias, M., Cavalli, R. C., and Sandrim, V. C. (2021). Circulating MicroRNAs in the Second Trimester From Pregnant Women Who Subsequently Developed Preeclampsia: Potential Candidates as Predictive Biomarkers and Pathway Analysis for Target Genes of miR-204-5p. *Front. Physiol.* 12, 678184. doi:10.3389/fphys.2021.678184.

Luo, R., Shao, X., Xu, P., Liu, Y., Wang, Y., Zhao, Y., et al. (2014). MicroRNA-210 Contributes to Preeclampsia by Downregulating Potassium Channel. *Hypertension* 64, 839–45. doi:10.1161/HYPERTENSIONAHA.114.03530.

Luo, S., Cao, N., Tang, Y., and Gu, W. (2017a). Identification of key microRNAs and genes in preeclampsia by bioinformatics analysis. *PLoS One* 12, e0178549. doi:10.1371/journal.pone.0178549.

Luo, S., Li, H., Cao, N., Tang, Y., and Gu, W. (2017b). MicroRNA-148a affects functions of placental trophoblast cells in preeclampsia by regulating HLA-G. *Int J Clin Exp Pathol* 10, 5205–5212.

Luque, A., Farwati, A., Crovetto, F., Crispi, F., Figueras, F., Gratacós, E., et al. (2014). Usefulness of circulating microRNAs for the prediction of early preeclampsia at first-trimester of pregnancy. *Sci. Rep.* 4, 4882. doi:10.1038/srep04882.

Lykoudi, A., Kolialexi, A., Lambrou, G. I., Braoudaki, M., Siristatidis, C., Papaioanou, G. K., et al. (2018). Dysregulated placental microRNAs in Early and Late onset Preeclampsia. *Placenta* 61, 24–32. doi:10.1016/j.placenta.2017.11.005.

Ma, R., Lu, Y., Dou, C., and Gu, Q. (2019). Clinical significance of miR-133a and miR-206 in pregnant women with preeclampsia and correlation with pregnancy outcomes. *Int J Clin Exp Med* 12, 7383–7391.

Martinez-Fierro, M. L., Carrillo-Arriaga, J. G., Luevano, M., Lugo-Trampe, A., Delgado-Enciso, I., Rodriguez-Sanchez, I. P., et al. (2019). Serum levels of miR-628-3p and miR-628-5p during the early pregnancy are increased in women who subsequently develop preeclampsia. *Pregnancy Hypertens.* 16, 120–125. doi:10.1016/j.preghy.2019.03.012.

Martinez-Fierro, M. L., Garza-Veloz, I., Gutierrez-Arteaga, C., Delgado-Enciso, I., Barbosa-Cisneros, O. Y., Flores-Morales, V., et al. (2018). Circulating levels of specific members of chromosome 19 microRNA cluster are associated with preeclampsia development. *Arch. Gynecol. Obstet.* 297, 365–371. doi:10.1007/s00404-017-4611-6.

Mayor-Lynn, K., Toloubeydokhti, T., Cruz, A. C., and Chegini, N. (2011). Expression profile of microRNAs and mRNAs in human placentas from pregnancies complicated by preeclampsia and preterm labor. *Reprod. Sci.* 18, 46–56. doi:10.1177/1933719110374115.

Mei, Z., Huang, B., Zhang, Y., Qian, X., Mo, Y., and Deng, N. (2019). Histone deacetylase 6 negatively regulated microRNA-199a-5p induces the occurrence of preeclampsia by targeting VEGFA in vitro. *Biomed. Pharmacother.* 114, 108805. doi:10.1016/j.biopha.2019.108805.

Meng, H.-X., Xu, L.-N., Jing, G., Qian, L., and Qi, M.-G. (2017). MiR-223 promotes trophoblast cell survival and invasion by targeting STAT3 in preeclampsia. *Int J Clin Exp Med* 10, 6577–6585.

Miura, K., Higashijima, A., Murakami, Y., Tsukamoto, O., Hasegawa, Y., Abe, S., et al. (2015). Circulating chromosome 19 miRNA cluster microRNAs in pregnant women with severe pre-eclampsia. *J. Obstet. Gynaecol. Res.* 41, 1526–1532. doi:10.1111/jog.12749.

Motawi, T. M. k., Sabry, D., Maurice, N. W., and Rizk, S. M. (2018). Role of mesenchymal stem cells exosomes derived microRNAs; miR-136, miR-494 and miR-495 in pre-eclampsia diagnosis and evaluation. *Arch. Biochem. Biophys.* 659, 13–21. doi:10.1016/j.abb.2018.09.023.

Munaut, C., Tebache, L., Blacher, S., Noël, A., Nisolle, M., and Chantraine, F. (2016). Dysregulated circulating miRNAs in preeclampsia. *Biomed. Reports* 5, 686–692. doi:0.3892/br.2016.779.

Muralimanoharan, S., Maloyan, A., Mele, J., Guo, C., Myatt, L. G., and Myatt, L. (2012). MIR-210 modulates mitochondrial respiration in placenta with preeclampsia. *Placenta* 33, 816–823. doi:10.1016/j.placenta.2012.07.002.

Murphy, M. S. Q., Casselman, R. C., Tayade, C., and Smith, G. N. (2015). Differential expression of plasma microRNA in preeclamptic patients at delivery and 1 year postpartum. *Am. J. Obstet. Gynecol.* 213, 367.e1-367.e9. doi:10.1016/j.ajog.2015.05.013.

Nejad, R. M. A., Saeidi, K., Gharbi, S., Salari, Z., and Saleh-Gohari, N. (2019). Quantification of circulating miR-517c-3p and miR-210-3p levels in preeclampsia. *Pregnancy Hypertens.* 16, 75–78. doi:10.1016/j.preghy.2019.03.004.

Niu, Z. ru, Han, T., Sun, X. luan, Luan, L. xia, Gou, W. li, and Zhu, X. ming (2018). MicroRNA-30a-3p is overexpressed in the placentas of patients with preeclampsia and affects trophoblast invasion and apoptosis by its effects on IGF-1. *Am. J. Obstet. Gynecol.* 218, 249.e1-249.e12. doi:10.1016/j.ajog.2017.11.568.

Nizyaeva, N. V., Kulikova, G. V., Nagovitsyna, M. N., Kan, N. E., Prozorovskaya, K. N., Shchegolev, A. I., et al. (2017). Expression of MicroRNA-146a and MicroRNA-155 in Placental Villi in Early- and Late-Onset Preeclampsia. *Bull. Exp. Biol. Med.* 163, 394–399. doi:10.1007/s10517-017-3812-0.

Nizyaeva, N. V., Kulikova, G. V., Nagovitsyna, M. N., Kan, N. E., Prozorovskaya, K. N., and Shchegolev, A. I. (2018). Change in OncomicroRNA Expression in the Placenta during Preeclampsia. *Bull. Exp. Biol. Med.* 165, 793–797. doi:10.1007/s10517-018-4267-7.

Ospina-Prieto, S., Chaiwangyen, W., Herrmann, J., Groten, T., Schleussner, E., Markert, U. R., et al. (2016). MicroRNA-141 is upregulated in preeclamptic placentae and regulates trophoblast invasion and intercellular communication. *Transl. Res.* 172, 61–72. doi:10.1016/j.trsl.2016.02.012.

Pillay, P., Vatish, M., Duarte, R., Moodley, J., and Mackraj, I. (2019). Exosomal microRNA profiling in early and late onset preeclamptic pregnant women reflects pathophysiology. *Int. J. Nanomedicine* 14, 5637–5657. doi:10.2147/IJN.S208865.

Pineles, B. L., Romero, R., Montenegro, D., Tarca, A. L., Han, Y. M., Kim, Y. M., et al. (2007). Distinct subsets of microRNAs are expressed differentially in the human placentas of patients with preeclampsia. *Am. J. Obstet. Gynecol.* 196, 261.e1-261.e6. doi:10.1016/j.ajog.2007.01.008.

Salomon, C., Guanzon, D., Scholz-Romero, K., Longo, S., Correa, P., Illanes, S. E., et al. (2017). Placental exosomes as early biomarker of preeclampsia: Potential role of exosomalmicrornas across gestation. *J. Clin. Endocrinol. Metab.* 102, 3182–3194. doi:10.1210/jc.2017-00672.

Sandrim, V. C., Dias, M. C., Bovolato, A. L. de C., Tanus-Santos, J. E., Deffune, E., and Cavalli, R. C. (2016a). Plasma from pre-eclamptic patients induces the expression of the anti-angiogenic miR-195-5p in endothelial cells. *J. Cell. Mol. Med.* 20, 1198–1200. doi:10.1111/jcmm.12767.

Sandrim, V. C., Luizon, M. R., Palei, A. C., Tanus-Santos, J. E., and Cavalli, R. C. (2016b). Circulating microRNA expression profiles in pre-eclampsia: evidence of increased miR-885-5p levels. *BJOG* 123, 2120–2128. doi:10.1111/1471-0528.13903.

Sekar, D., Lakshmanan, G., Mani, P., and Biruntha, M. (2019). Methylation-dependent circulating microRNA 510 in preeclampsia patients. *Hypertens. Res.* 42, 1647–1648. doi:10.1038/s41440-019-0269-8.

Shao, X., Liu, Y., Liu, M., Wang, Y., Yan, L., Wang, H., et al. (2017). Testosterone Represses Estrogen Signaling by Upregulating miR-22: A Mechanism for Imbalanced Steroid Hormone Production in Preeclampsia. *Hypertension* 69, 721–730. doi:10.1161/HYPERTENSIONAHA.116.08468.

Shen, L., Li, Y., Li, R., Diao, Z., Yany, M., Wu, M., et al. (2018). Placenta-associated serum exosomal miR-155 derived from patients with preeclampsia inhibits eNOS expression in human umbilical vein endothelial cells. *Int. J. Mol. Med.* 41, 1731–1739. doi:10.3892/ijmm.2018.3367.

Shi, Z., She, K., Li, H., Yuan, X., Han, X., and Wang, Y. (2019). MicroRNA-454 contributes to sustaining the proliferation and invasion of trophoblast cells through inhibiting Nodal/ALK7 signaling in pre-eclampsia. *Chem. Biol. Interact.* 298, 8–14. doi:10.1016/j.cbi.2018.10.012.

Singh, K., Williams, J., Brown, J., Wang, E. T., Lee, B., Gonzalez, T. L., et al. (2017). Up-regulation of microRNA-202-3p in first trimester placenta of pregnancies destined to develop severe preeclampsia, a pilot study. *Pregnancy Hypertens.* 10, 7–9. doi:10.1016/j.preghy.2017.04.002.

Sun, M., Chen, H., Liu, J., Tong, C., and Meng, T. (2015). MicroRNA-34a inhibits human trophoblast cell invasion by targeting MYC. *BMC Cell Biol.* 16, 21. doi:10.1186/s12860-015-0068-2.

Takizawa, T., Ishibashi, O., Ohkuchi, A., Moksed Ali, M., Kurashina, R., Luo, S. S., et al. (2012). Hydroxysteroid (17-β) dehydrogenase 1 is dysregulated by miR-210 and miR-518c that are aberrantly expressed in preeclamptic placentas: A novel marker for predicting preeclampsia. *Hypertension* 59, 265–273. doi:10.1161/HYPERTENSIONAHA.111.180232.

Tang, Q., Gui, J., Wu, X., and Wu, W. (2019). Downregulation of miR-424 in placenta is associated with severe preeclampsia. *Pregnancy Hypertens.* 17, 109–112. doi:10.1016/j.preghy.2019.05.017.

Timofeeva, A. V., Gusar, V. A., Kan, N. E., Prozorovskaya, K. N., Karapetyan, A. O., Bayev, O. R., et al. (2018). Identification of potential early biomarkers of preeclampsia. *Placenta* 61, 61–71. doi:10.1016/j.placenta.2017.11.011.

Truong, G., Guanzon, D., Kinhal, V., Elfeky, O., Lai, A., Longo, S., et al. (2017). Oxygen tension regulates the miRNA profile and bioactivity of exosomes released from extravillous trophoblast cells-Liquid biopsies for monitoring complications of pregnancy. *PLoS One* 12, e0174514. doi:10.1371/journal.pone.0174514.

Tsai, P. Y., Li, S. H., Chen, W. N., Tsai, H. L., and Su, M. T. (2017). Differential miR-346 and miR-582-3p expression in association with selected maternal and fetal complications. *Int. J. Mol. Sci.* 18, 1570. doi:10.3390/ijms18071570.

Ura, B., Feriotto, G., Monasta, L., Bilel, S., Zweyer, M., and Celeghini, C. (2014). Potential role of circulating microRNAs as early markers of preeclampsia. *Taiwan. J. Obstet. Gynecol.* 53, 232–234. doi:10.1016/j.tjog.2014.03.001.

Vashukova, E. S., Glotov, A. S., Fedotov, P. V., Efimova, O. A., Pakin, V. S., Mozgovaya, E. V., et al. (2016). Placental microRNA expression in pregnancies complicated by superimposed pre‑eclampsia on chronic hypertension. *Mol. Med. Rep.* 14, 22–32. doi:10.3892/mmr.2016.5268.

Wang, C. Y., Tsai, P. Y., Chen, T. Y., Tsai, H. L., Kuo, P. L., and Su, M. T. (2019a). Elevated miR-200a and miR-141 inhibit endocrine gland-derived vascular endothelial growth factor expression and ciliogenesis in preeclampsia. *J. Physiol.* 597, 3069–3083. doi:10.1113/JP277704.

Wang, F., and Yan, J. (2018). MicroRNA-454 is involved in regulating trophoblast cell proliferation, apoptosis, and invasion in preeclampsia by modulating the expression of ephrin receptor B4. *Biomed. Pharmacother.* 107, 746–753. doi:10.1016/j.biopha.2018.08.055.

Wang, H., Zhang, L., Guo, X., Bai, Y., Li, Y. X., Sha, J., et al. (2018a). MiR-195 modulates oxidative stress-induced apoptosis and mitochondrial energy production in human trophoblasts via flavin adenine dinucleotide-dependent oxidoreductase domain-containing protein 1 and pyruvate dehydrogenase phosphatase regulatory subunit. *J. Hypertens.* 36, 306–318. doi:10.1097/HJH.0000000000001529.

Wang, N., Feng, Y., Xu, J., Zou, J., Chen, M., He, Y., et al. (2018b). miR-362-3p regulates cell proliferation, migration and invasion of trophoblastic cells under hypoxia through targeting Pax3. *Biomed. Pharmacother.* 99, 462–468. doi:10.1016/j.biopha.2018.01.089.

Wang, N., Li, R., and Xue, M. (2019b). Potential regulatory network in the PSG10P/miR-19a-3p/IL1RAP pathway is possibly involved in preeclampsia pathogenesis. *J. Cell. Mol. Med.* 23, 852–864. doi:10.1111/jcmm.13985.

Wang, R., Liu, W., Liu, X., Liu, X., Tao, H., Wu, D., et al. (2019c). MicroRNA-210 regulates human trophoblast cell line HTR-8/SVneo function by attenuating Notch1 expression: Implications for the role of microRNA-210 in pre-eclampsia. *Mol. Reprod. Dev.* 86, 896–907. doi:10.1002/mrd.23154.

Wang, S., Wang, X., Weng, Z., Zhang, S., Ning, H., and Li, B. (2017). Expression and role of microRNA 18b and hypoxia inducible factor‑1α in placental tissues of preeclampsia patients. *Exp. Ther. Med.* 14, 4554–4560. doi:10.3892/etm.2017.5067.

Wang, W., Feng, L., Zhang, H., Hachy, S., Satohisa, S., Laurent, L. C., et al. (2012a). Preeclampsia up-regulates angiogenesis-associated microRNA (i.e., miR-17, -20a, and -20b) that target ephrin-B2 and EPHB4 in human placenta. *J. Clin. Endocrinol. Metab.* 97, E1051-9. doi:10.1210/jc.2011-3131.

Wang, Y., Cheng, K., Zhou, W., Liu, H., Yang, T., Hou, P., et al. (2019d). miR-141-5p regulate ATF2 via effecting MAPK1/ERK2 signaling to promote preeclampsia. *Biomed. Pharmacother.* 115, 108953. doi:10.1016/j.biopha.2019.108953.

Wang, Y., Dong, Q., Gu, Y., and Groome, L. J. (2016a). Up-regulation of miR-203 expression induces endothelial inflammatory response: Potential role in preeclampsia. *Am. J. Reprod. Immunol.* 76, 482–490. doi:10.1111/aji.12589.

Wang, Y., Fan, H., Zhao, G., Liu, D., Du, L., Wang, Z., et al. (2012b). MiR-16 inhibits the proliferation and angiogenesis-regulating potential of mesenchymal stem cells in severe pre-eclampsia. *FEBS J.* 279, 4510–4524. doi:10.1111/febs.12037.

Wang, Y., Lumbers, E. R., Arthurs, A. L., De Meaultsart, C. C., Mathe, A., Avery-Kiejda, K. A., et al. (2018c). Regulation of the human placental (pro)renin receptor-prorenin-angiotensin system by microRNAs. *Mol. Hum. Reprod.* 24, 453–464. doi:10.1093/molehr/gay031.

Wang, Y., Yang, X., Yang, Y., Wang, W., Zhao, M., Liu, H., et al. (2016b). High-throughput deep screening and identification of four peripheral leucocyte microRNAs as novel potential combination biomarkers for preeclampsia. *J. Perinatol.* 36, 263–267. doi:10.1038/jp.2015.192.

Wang, Y., Zhang, Y., Wang, H., Wang, J., Zhang, Y., Wang, Y., et al. (2014). Aberrantly up-regulated miR-20a in pre-eclampsic placenta compromised the proliferative and invasive behaviors of trophoblast cells by targeting forkhead box protein A1. *Int. J. Biol. Sci.* 10, 973–982. doi:10.7150/ijbs.9088.

Wang, Z., Wang, P., Wang, Z., Qin, Z., Xiu, X., Xu, D., et al. (2019e). MiRNA-548c-5p downregulates inflammatory response in preeclampsia via targeting PTPRO. *J. Cell. Physiol.* 234, 11149–11155. doi:10.1002/jcp.27758.

Weedon-Fekjær, M. S., Sheng, Y., Sugulle, M., Johnsen, G. M., Herse, F., Redman, C. W., et al. (2014). Placental miR-1301 is dysregulated in early-onset preeclampsia and inversely correlated with maternal circulating leptin. *Placenta* 35, 709–717. doi:10.1016/j.placenta.2014.07.002.

Wei, J., Blenkiron, C., Tsai, P., James, J. L., Chen, Q., Stone, P. R., et al. (2017). Placental trophoblast debris mediated feto-maternal signalling via small RNA delivery: Implications for preeclampsia. *Sci. Rep.* 7, 14681. doi:10.1038/s41598-017-14180-8.

Whigham, C. A., MacDonald, T. M., Walker, S. P., Hiscock, R., Hannan, N. J., Pritchard, N., et al. (2020). MicroRNAs 363 and 149 are differentially expressed in the maternal circulation preceding a diagnosis of preeclampsia. *Sci. Rep.* 10, 18077. doi:10.1038/s41598-020-73783-w.

Winger, E. E., Reed, J. L., and Ji, X. (2014). First Trimester Pbmc Microrna Predicts Adverse Pregnancy Outcome. *Am. J. Reprod. Immunol.* 72, 515–526. doi:10.1111/aji.12287.

Winger, E. E., Reed, J. L., and Ji, X. (2015). First-trimester maternal cell microRNA is a superior pregnancy marker to immunological testing for predicting adverse pregnancy outcome. *J. Reprod. Immunol.* 110, 22–35. doi:10.1016/j.jri.2015.03.005.

Winger, E. E., Reed, J. L., Ji, X., and Nicolaides, K. (2018). Peripheral blood cell microRNA quantification during the first trimester predicts preeclampsia: Proof of concept. *PLoS One* 13, e0190654. doi:10.1371/journal.pone.0190654.

Witvrouwen, I., Mannaerts, D., Ratajczak, J., Boeren, E., Faes, E., van Craenenbroeck, A. H., et al. (2021). MicroRNAs targeting VEGF are related to vascular dysfunction in preeclampsia. *Biosci. Rep.* 41, BSR20210874. doi:10.1042/BSR20210874.

Wu, L., Zhou, H., Lin, H., Qi, J., Zhu, C., Gao, Z., et al. (2012). Circulating microRNAs are elevated in plasma from severe preeclamptic pregnancies. *Reproduction* 143, 389–397. doi:10.1530/REP-11-0304.

Xiao, J., Tao, T., Yin, Y., Zhao, L., Yang, L., and Hu, L. (2017). miR-144 may regulate the proliferation, migration and invasion of trophoblastic cells through targeting PTEN in preeclampsia. *Biomed. Pharmacother.* 94, 341–353. doi:10.1016/j.biopha.2017.07.130.

Xiaobo (赵肖波), Z., Qizhi, H., Zhiping, W., and Tao, D. (2019). Down-regulated miR-149-5p contributes to preeclampsia via modulating endoglin expression. *Pregnancy Hypertens.* 15, 201–208. doi:10.1016/j.preghy.2019.01.002.

Xie, N., Jia, Z., and Li, L. (2019). MIR-320a upregulation contributes to the development of Preeclampsia by inhibiting the growth and invasion of trophoblast cells by targeting interleukin 4. *Mol. Med. Rep.* 20, 3256–3264. doi:10.3892/mmr.2019.10574.

Xu, H., and Zhang, X. (2017). Abnormal expression of microRNA-370 regulates Endoglin expression in preeclampsia. *Int J Clin Exp Med* 10, 4943–4949.

Xu, J., Gu, Y., Lewis, D. F., Cooper, D. B., McCathran, C. E., and Wang, Y. (2019). Downregulation of vitamin D receptor and miR-126-3p expression contributes to increased endothelial inflammatory response in preeclampsia. *Am. J. Reprod. Immunol.* 82, e13172. doi:10.1111/aji.13172.

Xu, P., Zhao, Y., Liu, M., Wang, Y., Wang, H., Li, Y., et al. (2014). Variations of MicroRNAs in Human Placentas and Plasma From Preeclamptic Pregnancy. *Hypertension* 63, 1276–84. doi:10.1161/HYPERTENSIONAHA.113.02647.

Xue, F., Yang, J., Li, Q., and Zhou, H. (2019). Down-regulation of microRNA-34a-5p promotes trophoblast cell migration and invasion via targetting Smad4. *Biosci. Rep.* 39, BSR20181631. doi:10.1042/BSR20181631.

Yan, T., Liu, Y., Cui, K., Hu, B., Wang, F., and Zou, L. (2013). MicroRNA-126 regulates EPCs function: Implications for a role of miR-126 in preeclampsia. *J. Cell. Biochem.* 114, 2148–2159. doi:10.1002/jcb.24563.

Yang, H. L., Zhang, H. Z., Meng, F. R., Han, S. Y., and Zhang, M. (2019a). Differential expression of microRNA-411 and 376c is associated with hypertension in pregnancy. *Brazilian J. Med. Biol. Res.* 52, e7546. doi:10.1590/1414-431X20197546.

Yang, Q., Lu, J., Wang, S., Li, H., Ge, Q., and Lu, Z. (2011). Application of next-generation sequencing technology to profile the circulating microRNAs in the serum of preeclampsia versus normal pregnant women. *Clin. Chim. Acta* 412, 2167–2173. doi:10.1016/j.cca.2011.07.029.

Yang, S., Li, H., Ge, Q., Guo, L., and Chen, F. (2015). Deregulated microRNA species in the plasma and placenta of patients with preeclampsia. *Mol. Med. Rep.* 12, 527–534. doi:10.3892/mmr.2015.3414.

Yang, W., Wang, A., Zhao, C., Li, Q., Pan, Z., Han, X., et al. (2016). MiR-125b enhances IL-8 production in early-onset severe preeclampsia by targeting sphingosine-1-phosphate lyase 1. *PLoS One* 11, e0166940. doi:10.1371/journal.pone.0166940.

Yang, X., and Guo, F. (2019). MiR-342-3p suppresses cell migration and invasion in preeclampsia by targeting platelet-derived growth factor receptor α. *Mol. Med. Rep.* 20, 1772–1780. doi:10.3892/mmr.2019.10372.

Yang, X., and Meng, T. (2019). MicroRNA-431 affects trophoblast migration and invasion by targeting ZEB1 in preeclampsia. *Gene* 683, 225–232. doi:10.1016/j.gene.2018.10.015.

Yang, X., Zhang, J., and Ding, Y. (2017). Association of microRNA-155, interleukin 17A, and proteinuria in preeclampsia. *Med. (United States)* 96, e6509. doi:10.1097/MD.0000000000006509.

Yang, Y., Li, H., Ma, Y., Zhu, X., Zhang, S., and Li, J. (2019b). MiR-221-3p is down-regulated in preeclampsia and affects trophoblast growth, invasion and migration partly via targeting thrombospondin 2. *Biomed. Pharmacother.* 109, 127–134. doi:10.1016/j.biopha.2018.10.009.

Yang, Y., Xi, L., Ma, Y., Zhu, X., Chen, R., Luan, L., et al. (2019c). The lncRNA small nucleolar RNA host gene 5 regulates trophoblast cell proliferation, invasion, and migration via modulating miR-26a-5p/N-cadherin axis. *J. Cell. Biochem.* 120, 3173–3184. doi:10.1002/jcb.27583.

Youssef, H. M. G., and Marei, E. S. (2019). Association of MicroRNA-210 and MicroRNA-155 with severity of preeclampsia. *Pregnancy Hypertens.* 17, 49–53. doi:10.1016/j.preghy.2019.05.010.

Yu, Z., Zhang, Y., Zheng, H., Gao, Q., and Wang, H. (2021). LncRNA SNHG16 regulates trophoblast functions by the miR-218-5p/LASP1 axis. *J. Mol. Histol.* 52, 1021–1033. doi:10.1007/s10735-021-09985-x.

Zhang, C., Li, Q., Ren, N., Li, C., Wang, X., Xie, M., et al. (2015a). Placental miR-106a-363 cluster is dysregulated in preeclamptic placenta. *Placenta* 36, 250–252. doi:10.1016/j.placenta.2014.11.020.

Zhang, C., Li, Q., Ren, N., Li, C., Wang, X., Xie, M., et al. (2015b). Placental miR-106a∼363 cluster is dysregulated in preeclamptic placenta. *Placenta* 36, 250–252. doi:10.1016/j.placenta.2014.11.020.

Zhang, Y., Diao, Z., Su, L., Sun, H., Li, R., Cui, H., et al. (2010). MicroRNA-155 contributes to preeclampsia by down-regulating CYR61. *Am. J. Obstet. Gynecol.* 202, 466.e1-466.e7. doi:10.1016/j.ajog.2010.01.057.

Zhang, Y., Fei, M., Xue, G., Zhou, Q., Jia, Y., Li, L., et al. (2012). Elevated levels of hypoxia-inducible microRNA-210 in pre-eclampsia: New insights into molecular mechanisms for the disease. *J. Cell. Mol. Med.* 16, 249–259. doi:10.1111/j.1582-4934.2011.01291.x.

Zhang, Y., Huang, G., Zhang, Y., Yang, H., Long, Y., Liang, Q., et al. (2017). MiR-942 decreased before 20 weeks gestation in women with preeclampsia and was associated with the pathophysiology of preeclampsia in vitro. *Clin. Exp. Hypertens.* 39, 108–113. doi:10.1080/10641963.2016.1210619.

Zhao, G., Zhou, X., Chen, S., Miao, H., Fan, H., Wang, Z., et al. (2014). Differential expression of microRNAs in decidua-derived mesenchymal stem cells from patients with pre-eclampsia. *J. Biomed. Sci.* 21, 81. doi:10.1186/s12929-014-0081-3.

Zheng, W., Chen, A., Yang, H., and Hong, L. (2020). MicroRNA‑27a inhibits trophoblast cell migration and invasion by targeting SMAD2: Potential role in preeclampsia. *Exp. Ther. Med.* 20, 2262–2269. doi:10.3892/etm.2020.8924.

Zhong, Y., Zhu, F., and Ding, Y. (2019). Differential microRNA expression profile in the plasma of preeclampsia and normal pregnancies. *Exp. Ther. Med.* 18, 826–32. doi:0.3892/etm.2019.7637.

Zhou, X., Li, Q., Xu, J., Zhang, X., Zhang, H., Xiang, Y., et al. (2016). The aberrantly expressed miR-193b-3p contributes to preeclampsia through regulating transforming growth factor-β signaling. *Sci. Rep.* 6, 19910. doi:10.1038/srep19910.

Zhu, X. ming, Han, T., Sargent, I. L., Yin, G. wu, and Yao, Y. qing (2009). Differential expression profile of microRNAs in human placentas from preeclamptic pregnancies vs normal pregnancies. *Am. J. Obstet. Gynecol.* 200, 661.e1-661.e7. doi:10.1016/j.ajog.2008.12.045.

Zou, A.-X., Chen, B., Li, Q.-X., and Liang, Y.-C. (2018). MiR-134 regulates the progression of preeclampsia. *Eur. Rev. Med. Pharmacol. Sci.* 22, 2199–2206. doi:10.26355/eurrev_201803_14557.

Zou, Y., Jiang, Z., Yu, X., Zhang, Y., Sun, M., Wang, W., et al. (2014). MiR-101 regulates apoptosis of trophoblast HTR-8/SVneo cells by targeting endoplasmic reticulum (ER) protein 44 during preeclampsia. *J. Hum. Hypertens.* 28, 610–616. doi:10.1038/jhh.2014.35.
